# Copper(II)-Mediated Synthesis of Indolequinones from Bromoquinones and Enamines

**DOI:** 10.1002/ejoc.201201597

**Published:** 2013-02-20

**Authors:** Martyn Inman, Christopher J Moody

**Affiliations:** [a] School of Chemistry, University of NottinghamUniversity Park, Nottingham NG7 2RD, UK Fax: +44-115-951-3564, E-mail: martyn.inman@nottingham.ac.ukc.j.moody@nottingham.ac.uk Homepage: http://www.nottingham.ac.uk/∼pczcm3/

**Keywords:** Synthetic methods, Cycliza­tion, Nitrogen heterocycles, Quinones, Copper

## Abstract

The reaction of enamines and bromoquinones in the presence of copper(II) acetate and potassium carbonate results in a regiospecific synthesis of indolequinones. The reaction is broad in scope and scalable and provides a route to the core structure that is present in several biologically interesting natural and synthetic compounds.

## Introduction

**Figure 1:**
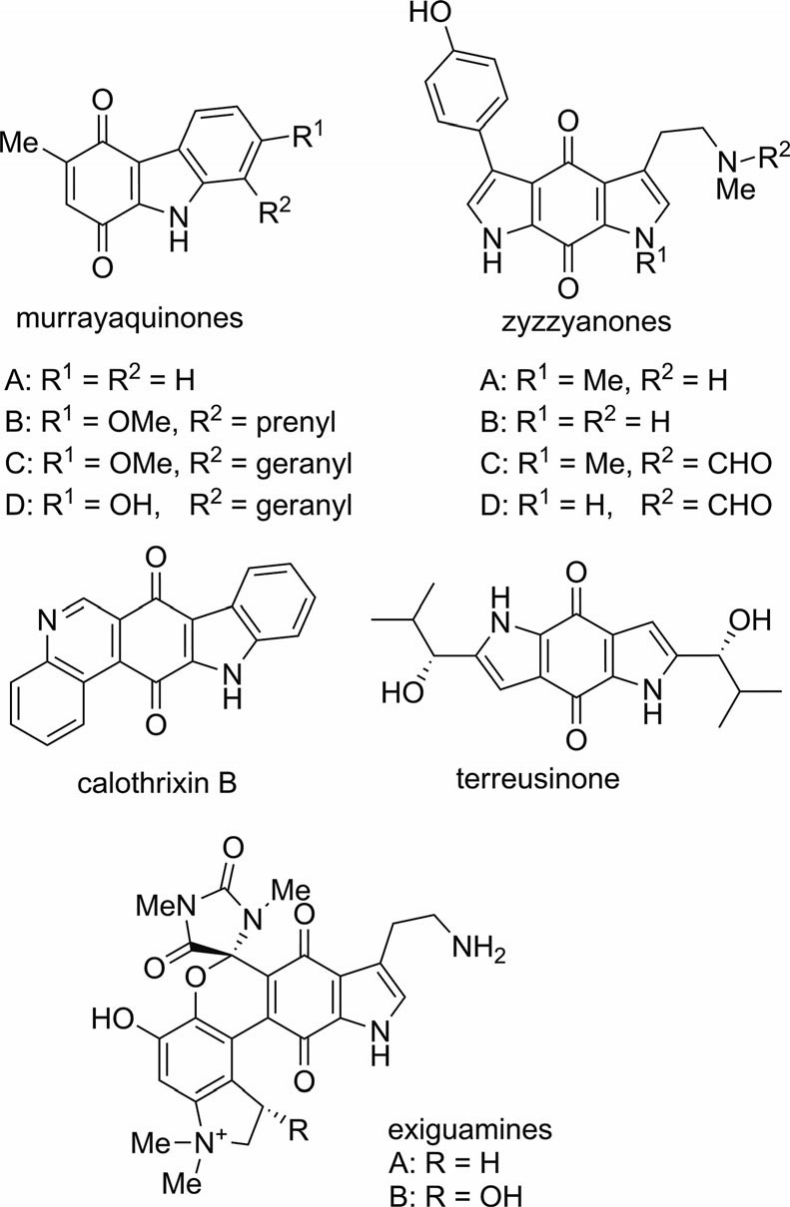
Naturally occurring indolequinones.

**Figure 2:**
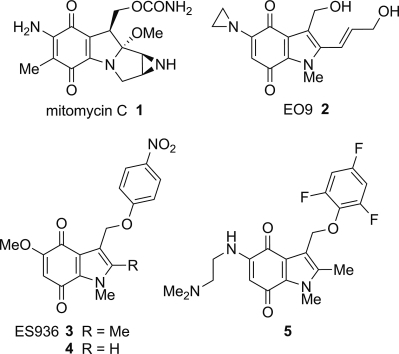
Bioactive indolequinones.

The indolequinone motif constitutes the core structure in a number of classes of natural products including the murrayaquinones, zyzzyanones, and exiguamines as well as the natural products calothrixin B and terreusinone (see Figure 1).^1^ Mitomycin C (**1**), the most important of the indolequinone natural products, is used clinically for the treatment of several solid tumors,^2^ and its synthetic analogue EO9 (**2**) is currently in clinical trials for the treatment of bladder cancer.^3^ The indolequinone structures ES936 (**3**)^4^ and **5**^5^ have found use as potent and selective inhibitors of the human quinone reductases NQO1 and NQO2, respectively. Related indolequinones such as **4**^6^ show great promise for treatment against pancreatic cancer (see Figure 2). Furthermore, the indolequinone core has been used in the design of bioreductive prodrugs of various active agents such as 5-fluorodeoxyuridine, camptothecin, and naloxone.^7^ Hence, there is significant interest in the development of improved methods for the synthesis of indolequinones.

Our continuing study of the chemistry and biology of indolequinones required a synthetic route that would provide rapid access to a range of structures with a sufficiently broad substrate scope, which would then allow us to study the effect of each substituent in turn. We now report in full detail our development of such a route through an oxidative copper(II)-mediated reaction of bromoquinones and enamines.

**Scheme 1:**
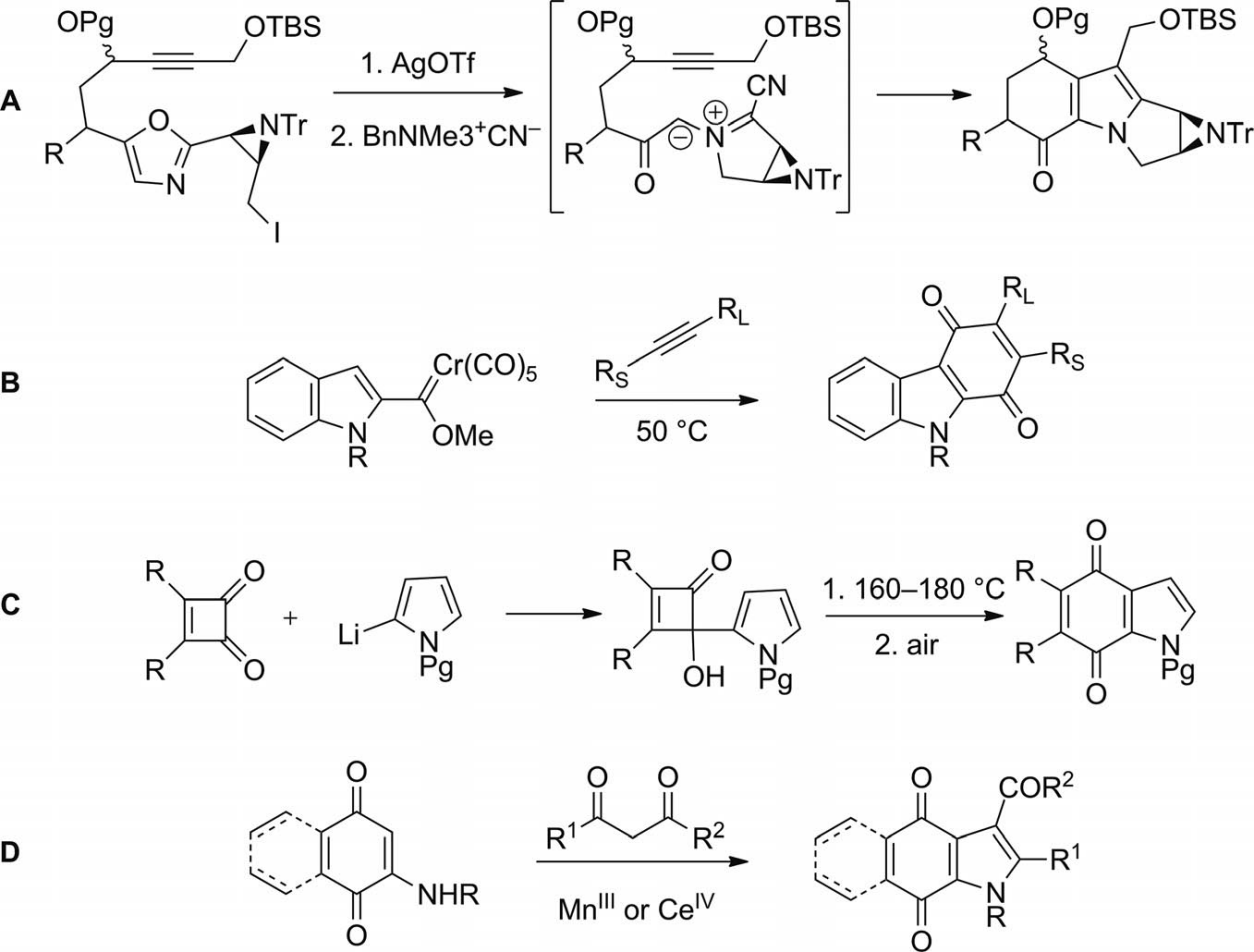
Approaches to the synthesis of indolequinones (TBS = *tert*-butyldimethylsilyl, Tr = triphenylmethyl, Pg = protecting group).

Traditionally, indolequinones have been synthesized by the oxidation of indole derivatives, which usually contain at least one hydroxy, methoxy, or amino group at the 4- and 7-positions.^8^ Although such methods are usually effective, they require access to highly substituted indole precursors that typically require an overall laborious synthetic route.^9^ The subsequent oxidation can also be problematic with regard to regiochemistry and functional group tolerance. Alternative approaches have been developed, which include intramolecular cycloadditions of alkynes with azomethine ylides (see Scheme 1, A),^10^ the reaction of Fischer carbenes with alkynes (see Scheme 1, B),^11^ the addition of lithiated pyrroles to cyclobutenediones (see Scheme 1, C),^12^ and the cerium(IV)- or manganese(III)-mediated oxidative cyclizations of aminoquinones with 1,3-dicarbonyl compounds (Scheme 1, D).^13^ Palladium-catalyzed Hegedus,^14^ Castro,^15^ and Mori-Ban^16^ indole syntheses have also been performed with aminoquinone derivatives to give indolequinones in moderate yields.

## Results and Discussion

**Scheme 2:**
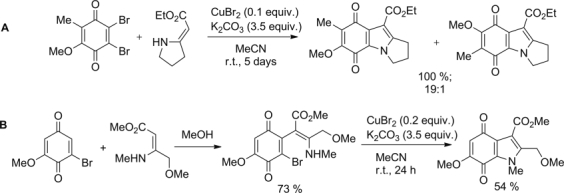
Reactions of bromoquinones with enamines.^19^

As part of their approach to the synthesis of mitosenes, Luly and Rapoport reported that the reaction of 2,3-dibromobenzoquinones with vinylogous carbamates in the presence of copper(II) bromide gave indolequinones that contained esters at the 3-position (see Scheme 2, A).^17^ Although it proceeded in excellent yield, the reaction took five days to reach completion and gave a mixture of regioisomers, which could only be separated by preparative MPLC. This issue of regioselectivity was resolved by Murphy's development of a similar reaction that used monobromoquinones as the starting materials and air as a terminal oxidant. The bromine substituent in the starting material indicated the position of attachment for the nitrogen atom (see Scheme 2, B).^18^ This reaction was applied to the total syntheses of EO9 and murrayaquinone A,^19^ but the improved regioselectivity came at the expense of generally low yields and a poor scope of substrates, as evidenced by the wide range of conditions that were employed. In our search for methods to synthesize indolequinones, we were attracted by the convergent nature of this reaction and the ready availability of the requisite starting materials. Hence, we sought to develop a set of reaction conditions that would give reliably good yields for a wide range of substrates.^20^,^21^

With this objective, a series of optimization experiments were performed, using the reaction of 2-bromo-6-methoxy-1,4-benzoquinone (**6a**) with methyl (methylamino)crotonate (**7a**, see Table 1). A breakthrough came with the discovery that relatively forcing conditions ― 3 equiv. of Cu(OAc)_2_**·**H_2_O at 140 °C in *N*,*N*-dimethylformamide (DMF) ― gave a satisfactory result (see Table 1, Entry 7). Further optimization of the reaction conditions revealed that 1.5 equiv. of Cu(OAc)_2_**·**H_2_O and 3 equiv. of K_2_CO_3_ in acetonitrile at reflux under air gave a yield of 89 % (see Table 1, Entry 12). The reaction could be repeated on a large scale (25 mmol) without a significant reduction in the yield, but isolation of the product by recrystallization afforded a diminished yield of 65 %.

**Table 1:** Optimization of the synthesis of indolequinone 8

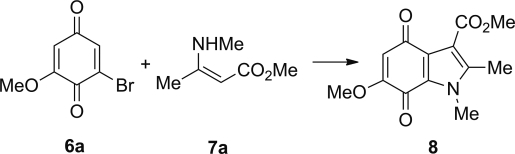						
Entry	Oxidant [equiv.]	Solvent	Base [equiv.]	*t* [h]	*T* [°C]	Yield [%]
1	CuBr_2_ (0.2)	MeOH	K_2_CO_3_ (3.5)	2	r.t.	39
2	CuBr_2_ (1)	MeOH	K_2_CO_3_ (3.5)	2	r.t.	22
3	CuBr_2_ (0.2)	MeOH	NEt_3_ (3.5)	2	r.t.	0
4	–	MeCN	–	20	r.t.	32
5	CuBr_2_ (0.2)	MeCN	K_2_CO_3_ (3.5)	24	r.t.	41
6	CuBr_2_ (1.5)	MeCN	K_2_CO_3_ (3.5)	2.5	80	46
7	Cu(OAc)_2_**·**H_2_O (3)	DMF	K_2_CO_3_ (3)	0.17	140	62
8	Cu(OAc)_2_**·**H_2_O (3)	DMF	K_2_CO_3_ (3)	0.25	100	63
9	Cu(OAc)_2_**·**H_2_O (3)	MeCN	K_2_CO_3_ (3)	2.5	80	78
10	Cu(OAc)_2_**·**H_2_O (0.2)	MeCN	K_2_CO_3_ (3)	3.5	80	60
11	Cu(OAc)_2_**·**H_2_O (1.5)	MeCN	K_2_CO_3_ (3)	3.5	80	62[a]
12	Cu(OAc)_2_**·**H_2_O (1.5)	MeCN	K_2_CO_3_ (3)	3.5	80	89
13	–	MeCN	K_2_CO_3_ (3)	4.5	80	46

[a] Reaction was carried out under argon.

**Scheme 3:**
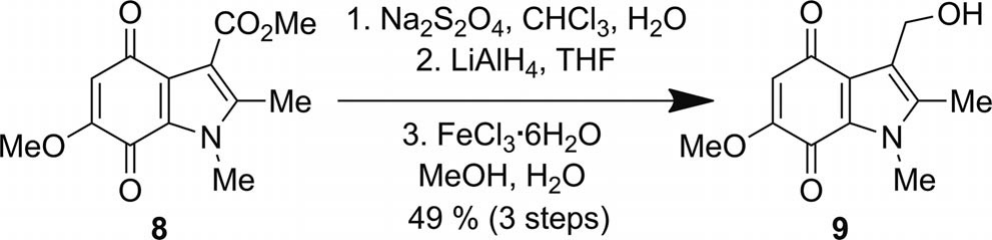
Conversion of indolequinone **8** into known compound **9**.

The regiochemistry of the reaction was confirmed through the reduction of the product with sodium dithionite followed by lithium aluminium hydride and then reoxidation to the known quinone **9** by treatment with iron(III) chloride (see Scheme 3). Comparison of the ^13^C NMR spectrum of **9** to the known 5-methoxy- and 6-methoxyindolequinones^22^ provided clear evidence that the regioselectivity matched that reported by Murphy.

**Scheme 4:**
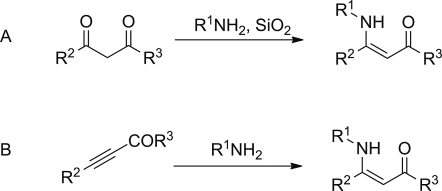
Synthesis of enamines.

With an optimized set of conditions in hand, a variety of bromoquinones and enamines were synthesized to examine the substrate scope. Enamines **7a**–**7w** were readily available in excellent yield either by the condensation of primary amines with β-keto esters or symmetrical 1,3-diketones, or by the 1,4-addition of primary amines to electron-deficient alkynes (see Scheme 4 and Table 2).

**Table 2:** Synthesis of enamines (Boc = *tert*-butoxycarbonyl, PMB = *para*-methoxybenzyl, TBDPS = *tert*-butyldiphenylsilyl)

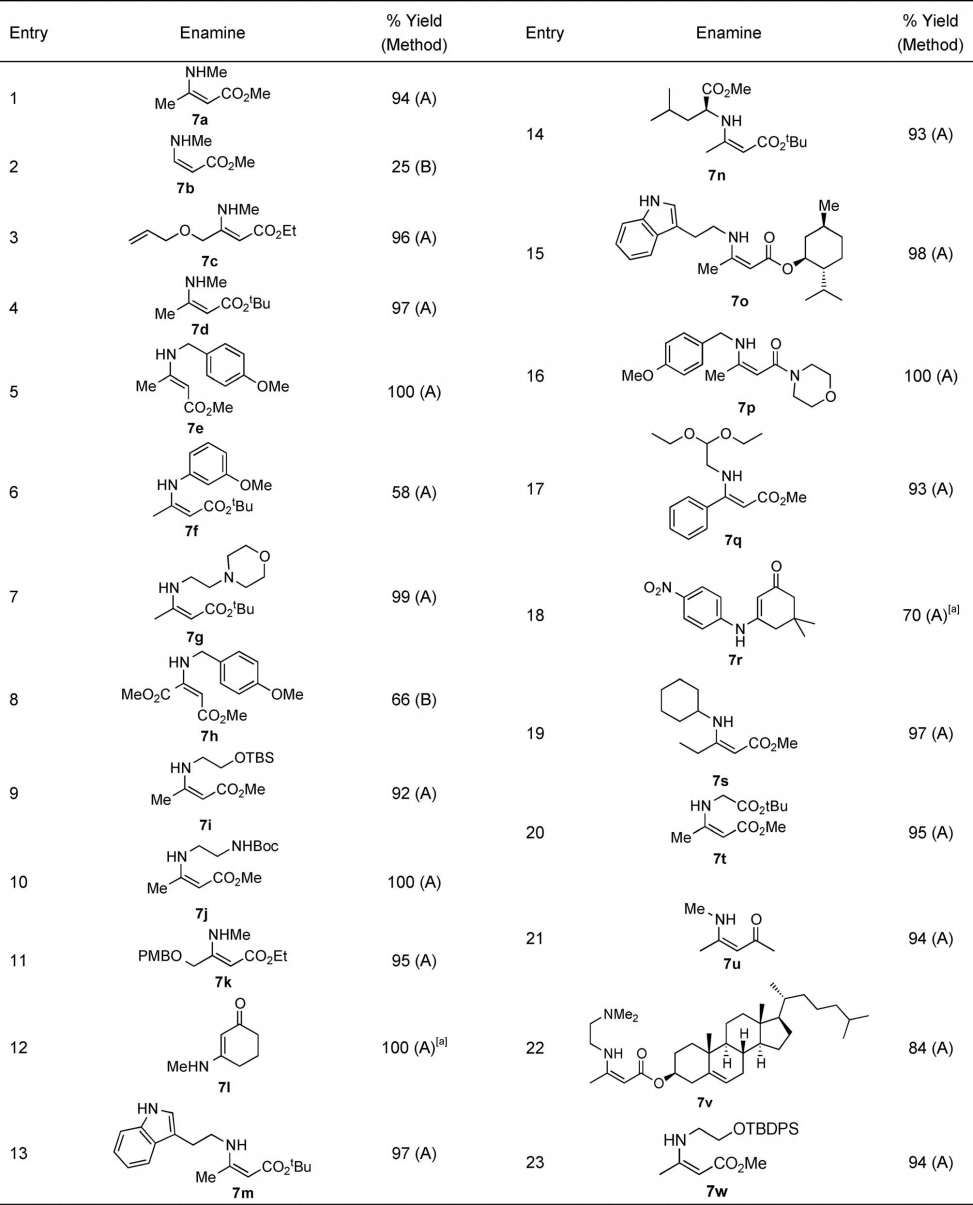

**Scheme 5:**
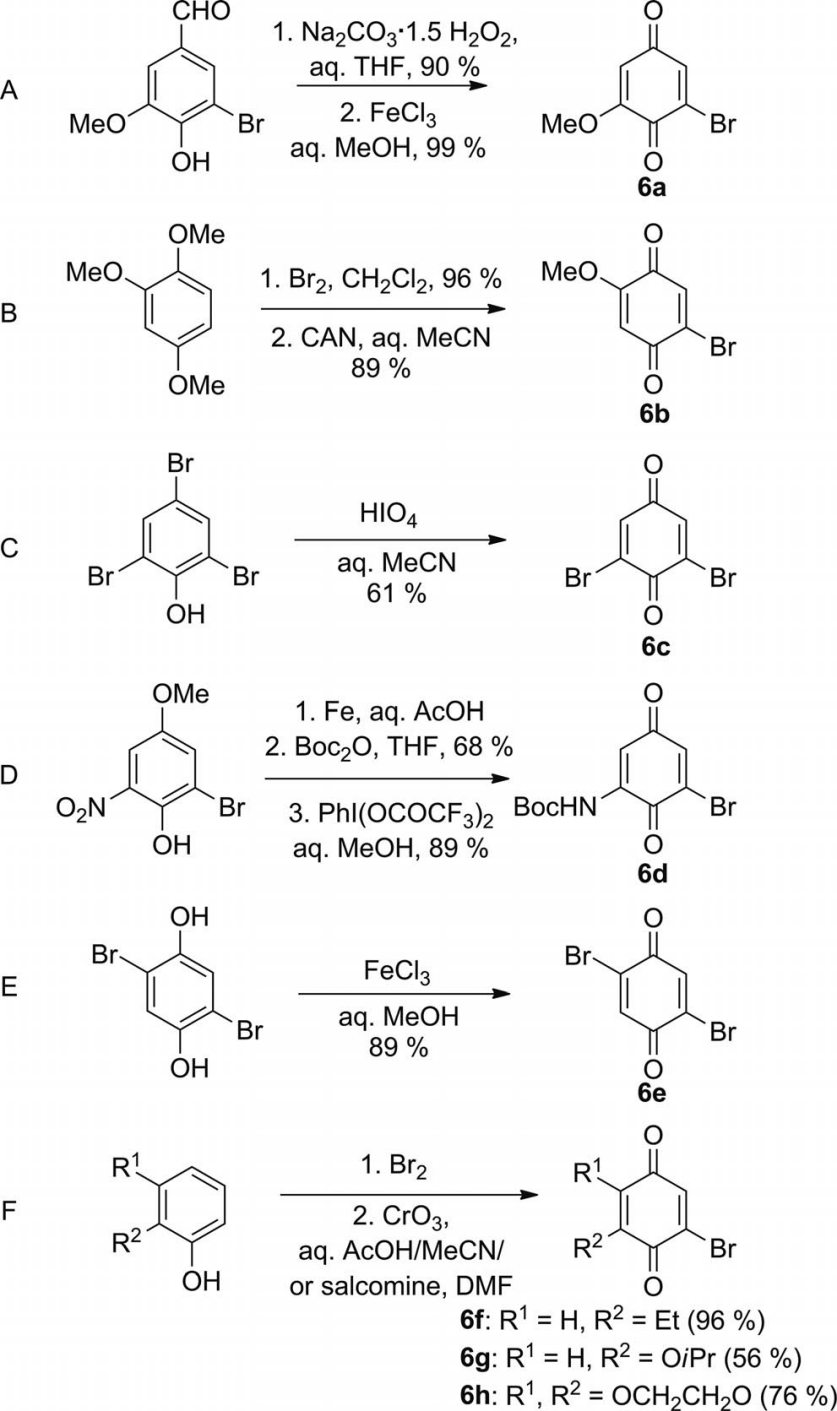
Syntheses of bromoquinones (THF = tetrahydrofuran, CAN = cerium(IV) ammonium nitrate).

Although commercially unavailable, the bromoquinone substrates **6b**–**6h** were typically synthesized in good yield from the phenolic precursors through bromination and oxidation reactions (see Scheme 5, B–F). However, 2-methoxy-6-bromobenzoquinone (**6a**) was prepared through a Dakin oxidation of 5-bromovanillin with sodium percarbonate, which was followed by an oxidation with iron(III) chloride (see Scheme 5, A).

**Scheme 6:**
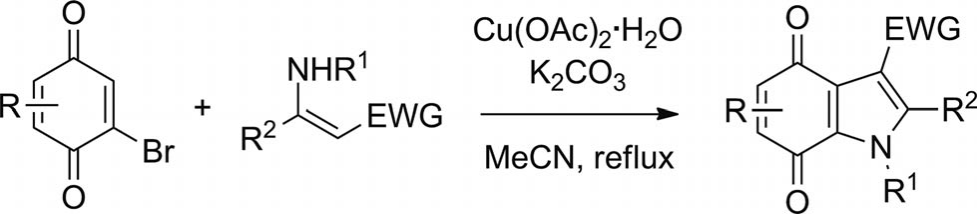
Synthesis of indolequinones (EWG = electron-withdrawing group).

A wide variety of functional and protecting groups were tolerated in the annulation reaction (see Scheme 6). These include methyl, ethyl, *tert*-butyl, menthyl, and cholesteryl esters, cyclic and acyclic ketones, amides, TBS, TBDPS, allyl, and PMB ethers, basic tertiary amines, Boc- and PMB-protected amines, nitro groups, and acetals (see Table 3). The N-substituent could be an alkyl or aryl group, but all attempts to synthesize *N*-unsubstituted indolequinones by using methyl aminocrotonate were unsuccessful, as were attempts to deprotect the corresponding PMB-protected indolequinones. Attempts to synthesize *N*-acyl indolequinones from enamides were also unsuccessful.

**Table 3:** Synthesis of indolequinones

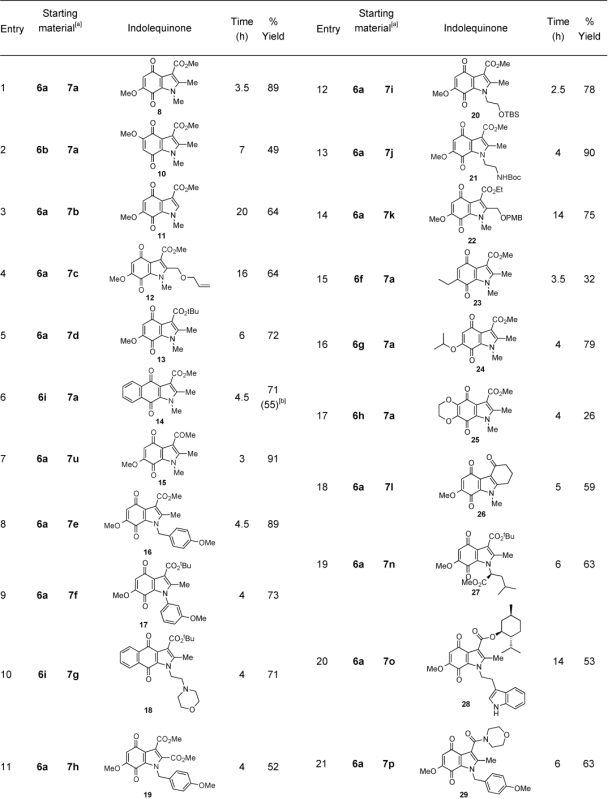
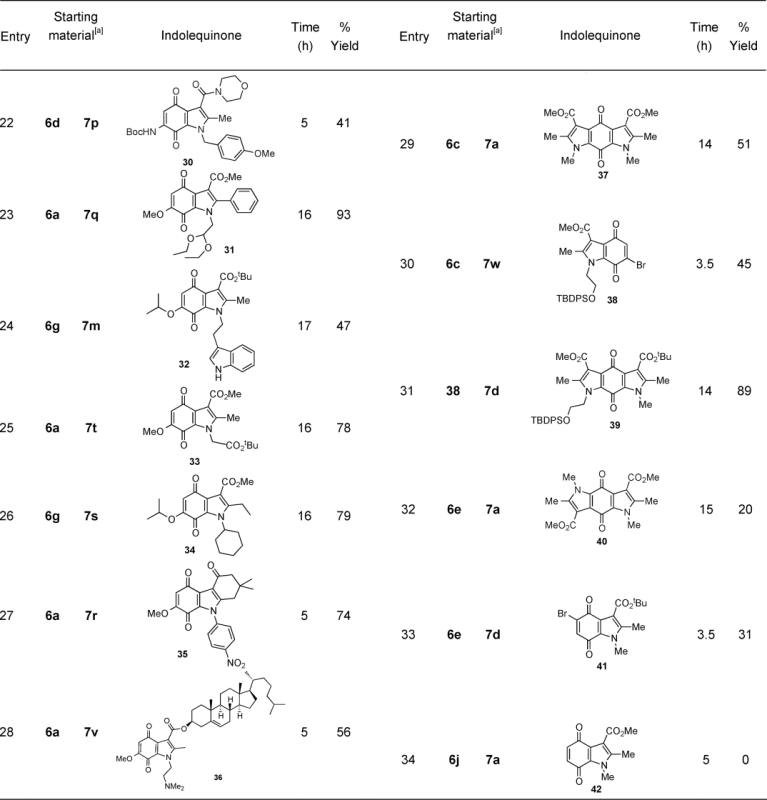

[a] Enamine (1 equiv.) was used with the exceptions of Entry 2 (4 equiv.), Entries 29, 31, and 32 (2 equiv.), and Entry 30 (0.5 equiv.).

[b] Chloroquinone was used instead of bromoquinone. Compound **6i** is 2-bromo-1,4-naphthoquinone.

The scope for substrates with substituents at the 5- and 6-positions of the quinone was slightly more limited. 2-Bromo-1,4-benzoquinone (**6j**) failed to give the expected 5,6-unsubstituted product, but instead the reaction resulted in decomposition (see Table 3, Entry 34). Strong electron-donating substituents such as alkoxy groups at either the 5- or 6-position gave the best results, but alkyl and carbamate substituents did not perform as well. Presumably, an electron-donating group is required to suppress an undesired nucleophilic attack at these positions. Optimum results were obtained with an electron-donating substituent at the 6-position rather than the 5-position, and this is consistent with the proposed mechanism (see Scheme 8).

Employing 2,6-dibromobenzoquinone (**6c**) as a substrate afforded symmetrical (see Table 3, Entry 29) and unsymmetrical (see Table 3, Entry 31) pyrroloindolequinones in moderate yields and in one and two steps, respectively. The corresponding reactions of 2,5-dibromobenzoquinone (**6e**) proved much less satisfactory, presumably for reasons to the poor performance of 2-bromo-5-methoxybenzoquinone (**6b**). Unsymmetrical pyrroloindolequinones could not be synthesized from 2,5-dibromo-1,4-benzoquinone in detectable quantities, and the symmetrical dimethyl ester **40** was obtained in only 20 % yield in comparison to 51 % yield for the corresponding compound **37** derived from the 2,6-dibromoquinone.

**Scheme 7:**
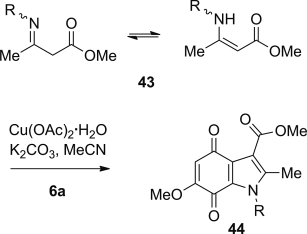
Synthesis of 1-amino- and 1-alkoxyindolequinones.

Next, the scope of the reaction was extended to include the synthesis of indolequinones that contain heteroatom substituents at the 1-position. The reaction of β-keto esters with 1,1-dialkylhydrazines gave inseparable mixtures of *E* and *Z* hydrazones with *E* and *Z* enehydrazines **43a**–**43b**. The reaction of these mixtures with bromoquinones under the optimized conditions gave the corresponding 1-aminoindolequinones **44a**–**44b** in moderate yields (see Scheme 7 and Table 4). Similarly, oxime ether **43c**, which was derived from the reaction of methoxylamine hydrochloride with methyl acetoacetate, gave the corresponding 1-methoxyindolequinone **44c** in only 16 % yield when sodium *tert*-butoxide was used instead of potassium carbonate. Varying the copper salt, base, and solvent failed to improve this yield.

**Table 4:** Synthesis of 1-amino- and 1-alkoxyindolequinones

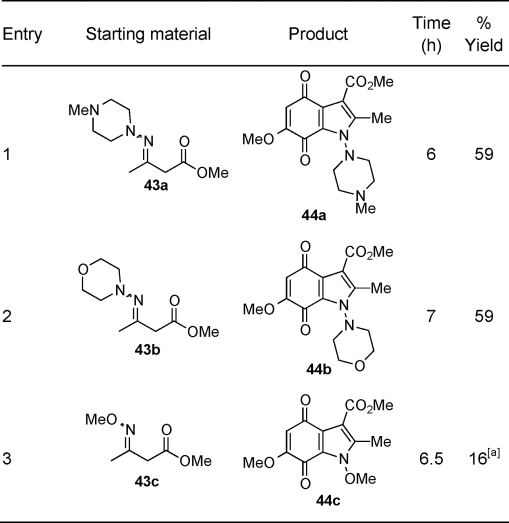

[a] Sodium *tert*-butoxide was used in place of potassium carbonate.

The relatively low cost of Cu(OAc)_2_**·**H_2_O compensates for the need to use stoichiometric quantities ― indeed, the HPLC-grade acetonitrile used as a solvent represents the most expensive reagent. The reaction did proceed with catalytic amounts of Cu(OAc)_2_ under air or with 1.5 equiv. of Cu(OAc)_2_ under argon, albeit in lower yields in both cases. This indicates that either air or Cu^II^ could act as the terminal oxidant, but the optimum conditions utilized both. Surprisingly, the reaction also proceeds under air in the absence of added copper salts, although with a diminished yield. The termination of the reaction prior to completion allowed the isolation of intermediate enamine **7**, which is indicative of a reaction mechanism that begins with a nucleophilic attack by the enamine on the more electrophilic C-3 of the quinone, which has no bromine atom.^23^ Oxidation of the resulting hydroquinone to **45** is followed by a C–N bond formation with the loss of HBr to deliver the product. The precise mechanism by which this occurs is unclear, although it has been attributed to the activation of the C–Br bond through the Lewis acidity of the copper salt.^19^ Although an Ullmann–Goldberg-type reaction cannot be entirely discounted, it seems unlikely given the success of the reaction without the addition of Cu(OAc)_2_. Using a chloroquinone in place of a bromoquinone gives the same reaction, but with a significant decrease in the rate of the C–N bond formation and, consequently, a lower yield. This finding could be consistent with either the Lewis acid activation hypothesis or an Ullmann–Goldberg reaction.

**Scheme 8:**
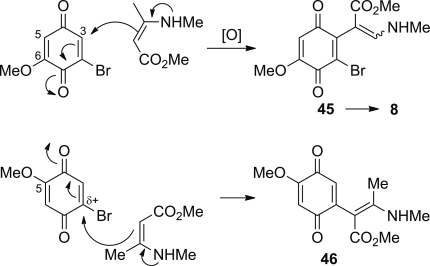
Mechanistic considerations.

5-Alkoxy substituents on the quinone generally resulted in lower yields, which was most likely the result of their deactivating influence on the 3-position. In these cases, the formation of side product **46** indicates competitive nucleophilic addition at the 2-position followed by loss of bromide to give the 2-aminovinyl quinone, which is unable to cyclize to the indolequinone under the reaction conditions (see Scheme 8).

## Conclusions

In summary, we report a versatile and practical synthesis of indolequinones by using the reaction of bromoquinones with enamines in the presence of copper(II) acetate and potassium carbonate. This method, which tolerates a wide range of functional groups, is effective on a gram scale and can be extended to the syntheses of pyrroloindolequinones, *N*-aminoquinones, and *N*-alkoxyquinones.

## Experimental Section

**General Methods:** Commercially available reagents were used throughout without purification, with the exception of tetrahydrofuran and dichloromethane, which were freshly distilled from sodium/benzophenone and calcium hydride, respectively. Light petroleum refers to the fraction with a boiling range of 40–60 °C, and “ether” refers to diethyl ether. Thin-layer chromatography was carried out with aluminum foil backed plates and visualized under UV light (at 254 and 360 nm) or by using vanillin or permanganate stains. Chromatography was carried out with silica gel, and the eluent is specified. Fully characterized compounds are chromatographically homogeneous. Infrared spectra were recorded with a FTIR spectrometer in the range 4000–600 cm^–1^ and chloroform as the solvent or as solids in attenuated total reflectance (ATR) mode. The NMR spectroscopic data were recorded at 300, 400, and 500 MHz for ^1^H NMR and at 75, 100, and 125 MHz for ^13^C NMR. The chemical shifts are reported in ppm and are referenced to the residual proton in the deuterated solvent as the internal standard. The coupling constants (*J*) are reported in hertz (Hz). In the ^13^C NMR spectra, the signals corresponding to the CH, CH_2_, or Me groups, as assigned from the DEPT spectra, are noted. All other signals correspond to quaternary carbons. High and low resolution mass spectra were recorded with a time-of-flight mass spectrometer.

**General Procedure for the Synthesis of Enamines 7:** A mixture of the primary amine (1.0 equiv.), the β-keto ester (1.0 equiv.), and silica gel (0.01–0.1 g/mmol) was stirred at room temperature overnight and then diluted with dichloromethane. The resulting solution was filtered, and the filtrate was concentrated to give the enamine, which was used without further purification.

**General Procedure for the Synthesis of Indolequinones:** A solution of enamine **7** (1.0–4.0 equiv.) in acetonitrile (5–10 mL/mmol) was added to a mixture of bromoquinone **6** (1.0 equiv.), copper(II) acetate monohydrate (1.5–2.0 equiv.), and potassium carbonate (3.0 equiv.). The resulting mixture was stirred, heated at reflux for the indicated time, cooled to room temperature, and then diluted with dichloromethane (20 mL/mmol). The resulting mixture was filtered through Celite, and the filtrate was concentrated in vacuo. Column chromatography of the residue gave the indolequinone.

**Methyl 6-Methoxy-1,2-dimethyl-4,7-dioxo-4,7-dihydro-1*H*-indole-3-carboxylate (8):** Prepared by following the general procedure, 2-bromo-6-methoxy-1,4-benzoquinone (**6a**, 0.109 g, 0.5 mmol), methyl 3-(methylamino)but-2-enoate (**7a**, 0.065 g, 0.5 mmol), copper(II) acetate monohydrate (0.150 g, 0.75 mmol), and potassium carbonate (0.207 g, 1.5 mmol) were stirred and heated at reflux in acetonitrile (5 mL) for 3.5 h. Column chromatography (ethyl acetate/light petroleum, 1:1) gave **8** (0.117 g, 89 %) as a yellow solid; m.p. 209–211 °C. IR (CHCl_3_): 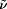
_max_ = 3620, 3007, 2976, 1447, 1248, 1046 cm^–1^. ^1^H NMR (400 MHz, CDCl_3_): *δ* = 5.71 (s, 1 H), 3.93 (s, 3 H), 3.91 (s, 3 H), 3.82 (s, 3 H), 2.45 (s, 3 H) ppm. ^13^C NMR (75 MHz, CDCl_3_): *δ* = 181.3, 172.3, 164.7, 158.7, 142.6, 127.8, 124.5, 112.9, 107.5 (CH), 56.6 (Me), 52.0 (Me), 32.8 (Me), 10.9 (Me) ppm. HRMS (ESI): calcd. for C_13_H_13_NO_5_Na [M + Na]^+^ 286.0686; found 286.0676.

**Supporting Information** (see footnote on the first page of this article): Full experimental details and copies of the ^1^H and ^13^C NMR spectra.

## Supplementary material

As a service to our authors and readers, this journal provides supporting information supplied by the authors. Such materials are peer reviewed and may be re-organized for online delivery, but are not copy-edited or typeset. Technical support issues arising from supporting information (other than missing files) should be addressed to the authors.
