# Supplementary material for: Copper(II)-Mediated Synthesis of Indolequinones from Bromoquinones and Enamines
Source: European J Org Chem. 2013 Feb 20;2013(11):2179–87. doi: 10.1002/ejoc.201201597 (PMC3659408; doi:10.1002/ejoc.201201597)

Eur. J. Org. Chem. **2013** · © WILEY-VCH Verlag GmbH & Co. KGaA, 69451 Weinheim, 2013 · ISSN 1434–193X

SUPPORTING INFORMATION

DOI: 10.1002/ejoc.201201597
Title: Copper(II)-Mediated Synthesis of Indolequinones from Bromoquinones and Enamines
Author(s): Martyn Inman,* Christopher J. Moody

Contents

General experimental details S2

Preparation of enamines S2

Preparation of bromoquinones S17

Preparation of indolequinones S24

References S46

Copies of ^{1}H and ^{13}C NMR spectra S48

General experimental details

Commercially available reagents were used throughout without purification, except tetrahydrofuran and dichloromethane, which were freshly distilled from sodium/benzophenone and calcium hydride respectively. Light petroleum refers to the fraction with bp 40-60 °C and ether refers to diethyl ether. Thin layer chromatography was carried out on aluminum foil backed plates, visualized under UV light (at 254 and/or 360 nm) or by vanillin or permanganate stains. Chromatography was carried out using silica gel, with the eluent specified. Fully characterized compounds are chromatographically homogeneous. Infrared spectra were recorded on an FTIR spectrometer, in the range 4000-600 cm^{-1} using chloroform as solvent. NMR spectra were recorded at 300, 400 and 500 MHz (^{1}H frequencies, corresponding ^{13}C frequencies 75, 100 and 125 MHz). Chemical shifts are quoted in ppm and are referenced to residual H in the deuterated solvent as the internal standard. *J* values are recorded in Hz. In the ^{13}C spectra, signals corresponding to CH, CH_2, or Me groups, as assigned from DEPT, are noted; all others are quaternary C. High and low resolution mass spectra were recorded on a time-of-flight mass spectrometer.

Preparation of Enamines

***(Z)*-Methyl 3-(methylamino)but-2-enoate (7a)**

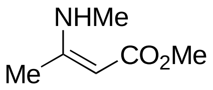

A solution of methylamine (25% w/v in water; 14.8 mL, 120 mmol) was added in a single portion to a stirred suspension of silica gel (1.0 g) and methyl acetoacetate (11.5 g, 100 mmol) at room temperature, and the resulting mixture was stirred at room temperature for 15 h. The mixture was extracted with dichloromethane (3 × 25 mL), and the combined organic phases were dried ($MgSO_4$), filtered and concentrated *in vacuo* to give the *title compound* as an off-white solid (12.18 g, 94%); mp 58-60 °C (lit.,[1] mp 63-66 °C); (Found: $M+Na^+$, 152.0681. $C_6H_{11}NO_2Na$ requires 152.0682); δ_H (400 MHz; $CDCl_3$) 8.50 (1H, br s, NH), 4.51 (1H, s, CH), 3.66 (3H, s, OMe), 2.95 (3H, d, *J* 5.2, NMe), 1.96 (3H, s, Me); δ_C (75 MHz; $CDCl_3$) 171.0, 162.9, 81.5 (CH), 50.0 (Me), 30.0 (Me), 19.2 (Me); *m/z* (ESI) 152 ($M+Na^+$, 100%).

(*E/Z*)-Methyl 3-(methylamino)propenoate (7b)

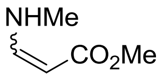

Methyl propiolate (8.40 g, 100 mmol) was added to a mixture of methylamine hydrochloride (6.75 g, 100 mmol) and potassium carbonate (13.80 g, 100 mmol) in acetonitrile (50 mL) at room temperature, and the resulting mixture was stirred at reflux for 24 h, cooled, filtered and concentrated *in vacuo*. Vacuum distillation (2 mbar, vapour temperature 35-40 °C) gave the *title compound* as a colorless oil (2.86 g, 25%, 8:1 *Z*:*E*); (lit.,[2] bp 90-93 °C at 10 mbar); (Found: M+Na$^+$, 138.0492. $C_5H_9NO_2Na$ requires 138.0525); δ_H (400 MHz; $CDCl_3$) *Z* isomer: 7.65 (1H, br s, NH), 6.58 (1H, dd, *J* 13.2, 8.0, CH), 4.47 (1H, d, *J* 8.0, CH), 3.64 (3H, s, OMe), 2.96 (3H, d, *J* 5.2, NMe); *E* isomer: 7.60 (1H, m, CH), 4.70 (1H, d, *J* 13.2, CH), 4.61 (1H, br s, NH), 3.66 (3H, s, OMe), 2.76 (3H, d, *J* 5.2, NMe); δ_C (75 MHz; $CDCl_3$) mixture: 171.2, 170.0, 153.5 (CH), 85.1 (CH), 81.3 (CH), 50.5 (Me), 50.1 (Me), 34.9 (Me); *m/z* (ESI) 138 (M+Na$^+$, 100%).

***(E/Z)*-Ethyl 4-allyloxy-3-(methylamino)but-2-enoate (7c)**

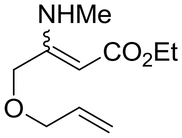

(a) A solution of allyl alcohol (3.3 mL, 47 mmol) in THF (17 mL) was added to a stirred suspension of sodium hydride (60% dispersion in oil; 3.8 g, 94 mmol) in THF (33 mL) at 0 °C, and the resulting mixture was stirred at 0 °C for 1 h, then at reflux for 1 h, then cooled to 0 °C. A solution of ethyl 4-chloroacetoacetate (4.94 mL, 36.7 mmol) in THF (17 mL) was added dropwise over 2 min, and the resulting mixture was allowed to warm to room temperature with stirring over 16 h. The mixture was acidified to pH 2 by dropwise addition of hydrochloric acid (1 M), and water (50 mL) and ethyl acetate (50 mL) were added. The aqueous phase was further extracted with ethyl acetate (3 × 50 mL) and the combined organic phases were dried ($MgSO_4$), filtered and concentrated *in vacuo*. Column chromatography of the residue eluting with ethyl acetate and light petroleum (1:4) gave ethyl 4-allyloxy-3-oxobutanoate[3] (5.32 g, 78%) as a pale yellow liquid (Found: M+Na$^+$, 209.0798. $C_9H_{14}O_4Na$ requires 209.0784); ν_{max} ($CHCl_3$)/cm^{-1} 2986, 1720, 1659; δ_H (400 MHz; $CDCl_3$) 5.94-5.87 (1H, m, CH), 5.31 (1H, d, *J* 17.2, CH), 5.24 (1H, d, *J* 10.4, CH), 4.20 (2H, q, *J* 7.2, OCH_2), 4.13 (2H, s, CH_2), 4.05 (2H, d, *J* 5.2, OCH_2), 3.54 (2H, s, CH_2), 1.29 (3H, t, *J* 7.2, $CH_2C\underline{H}_3$); δ_C (75 MHz; $CDCl_3$) 201.9, 167.0, 133.5 (CH), 118.1 (CH_2), 74.7 (CH_2), 72.4 (CH_2), 61.4 (CH_2), 46.0 (CH_2), 14.1 (Me); *m/z* (ESI) 395 (2M+Na$^+$, 100%), 209 (M+Na$^+$, 30).

(b) A solution of methylamine (25% w/v in H_2O; 1.86 mL, 15 mmol) was added in a single portion to a stirred suspension of silica gel (1.0 g) and ethyl 4-allyloxy-3-oxobutanoate (2.79 g, 15 mmol) at room temperature, and the resulting mixture was stirred at room temperature for 15 h. The mixture was diluted with dichloromethane (20 mL), dried ($MgSO_4$), filtered and concentrated *in vacuo* to give the *title compound* as a pale brown oil (2.88 g, 96%; 1.5:1 *E*:*Z*); (Found: M+Na$^+$, 222.1106. $C_{10}H_{17}NO_3Na$ requires 222.1101); ν_{max} ($CHCl_3$)/cm^{-1} 3430, 3008, 2984, 1674, 1656, 1592, 1153; δ_H (400 MHz; $CDCl_3$) *E* isomer: 5.96-5.92 (m, 1H, CH), 5.74 (1H, br s, NH), 5.31 (1H, d, *J* 17.2, CH), 5.23 (1H, d, *J* 10.4, CH), 4.78 (2H, s, OCH_2), 4.52 (1H, s, CH), 4.14-4.02 (m, 4H, 2 × OCH_2), 2.77 (3H, d, *J* 5.2, NMe), 1.27 (3H, t, *J* 7.0, CH_3); *Z* isomer: 8.25 (1H, br s, NH), 5.96-5.92 (m, 1H, CH), 5.31 (1H, d, *J* 17.2, CH), 5.23 (1H, d, *J* 10.4, CH), 4.66 (1H, s, CH), 4.14-4.02 (m, 6H, 3 × CH_2), 2.97 (3H, d, *J* 5.2, NMe), 1.27 (3H, t, *J* 7.0, CH_3); δ_C (75 MHz; $CDCl_3$) mixture: 170.7, 168.9, 160.7, 160.2, 134.0 (CH), 133.9 (CH), 117.8 (CH_2), 117.7 (CH_2), 82.9 (CH), 79.1 (CH), 72.2 (CH_2), 71.1 (CH_2), 68.8 (CH_2), 68.3 (CH_2), 58.6 (CH_2), 58.4 (CH_2), 29.6 (Me), 29.5 (Me), 14.7 (Me), 14.6 (Me); *m/z* (ESI) 421 (2M+Na$^+$, 100%), 222 (M+Na$^+$, 55).

***(Z)-tert*-Butyl 3-(methylamino)but-2-enoate (7d)**

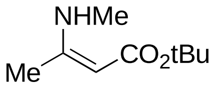

A solution of methylamine (25% w/v in water; 3.72 mL, 30 mmol) was added in a single portion to a stirred suspension of silica gel (0.3 g) and *tert*-butyl acetoacetate (3.95 g, 25 mmol) at room temperature, and the resulting mixture was stirred at room temperature for 15 h. The mixture was extracted with dichloromethane (3 × 15 mL), and the combined organic phases were dried ($MgSO_4$), filtered and concentrated *in vacuo* to give the *title compound* as a pale yellow oil (4.15 g, 97%); (Found: M+H$^+$, 172.1342. $C_9H_{18}NO_2$ requires 172.1332); ν_{max} ($CHCl_3$)/cm^{-1} 3306, 3005, 2980, 2931, 1595, 1293; δ_H (400 MHz; $CDCl_3$) 8.45 (1H, br s, NH), 4.41 (1H, s, CH), 2.89 (3H, d, *J* 4.8, NMe), 1.89 (3H, s, Me), 1.47 (9H, s, *t*Bu); δ_C (75 MHz; $CDCl_3$) 170.9, 162.2, 83.5 (CH), 77.7, 29.5 (Me), 28.6 (Me), 19.0 (Me); *m/z* (ESI) 365 (2M+Na$^+$, 100%), 172 (M+H$^+$, 79).

***(Z)*-Methyl 3-(4-methoxybenzylamino)but-2-enoate (7e)**

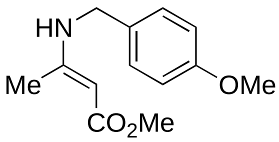

4-Methoxybenzylamine (1.37 g, 10 mmol) was added in a single portion to a stirred suspension of methyl acetoacetate (1.15 g, 10 mmol) and silica gel (0.1 g) at room temperature, and the resulting mixture was stirred at room temperature for 13 h. The mixture was diluted with dichloromethane (10 mL), filtered and concentrated *in vacuo* to give the *title compound* as a colorless liquid (2.35 g, 100%); (Found: $M+Na^+$, 258.1098. $C_{13}H_{17}NO_3Na$ requires 258.1101); ν_{max} ($CHCl_3$)/cm^{-1} 3297, 3009, 2951, 1649, 1605, 1514, 1248, 1174; δ_H (400 MHz; $CDCl_3$) 8.88 (1H, br s, NH), 7.20 (2H, d, *J* 8.4, ArH), 6.88 (2H, d, *J* 8.4, ArH), 4.53 (1H, s, CH), 4.37 (2H, d, *J* 6.4, CH_2), 3.81 (3H, s, OMe), 3.64 (3H, s, OMe), 1.94 (3H, s, Me); δ_C (75 MHz; $CDCl_3$) 170.9, 161.9, 158.9, 130.6, 128.2 (CH), 114.2 (CH), 82.6 (CH), 55.3 (Me), 50.0 (Me), 46.3 (CH_2), 19.4 (Me); *m/z* (ESI) 493 ($2M+Na^+$, 100%), 258 ($M+Na^+$, 15).

***(Z)-tert*-Butyl 3-(3-methoxyphenylamino)but-2-enoate (7f)**

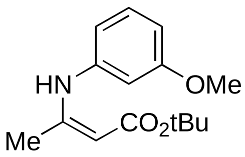

Zinc perchlorate hexahydrate (0.186 g, 0.5 mmol) and then $MgSO_4$ (0.360 g, 3 mmol) were added as single portions to a stirred solution of *m*-anisidine (1.23 g, 10 mmol) and *tert*-butyl acetoacetate (1.58 g, 10 mmol) in dichloromethane (2.5 mL) at room temperature. The resulting mixture was stirred at room temperature for 16 h, diluted with dichloromethane (20 mL), filtered through Celite and concentrated *in vacuo*. Column chromatography of the residue eluting with ethyl acetate and light petroleum (1:19) gave the *title compound* as a colorless oil (1.51 g, 58%); (Found: $M+H^+$, 264.1592. $C_{15}H_{22}NO_3$ requires 264.1594); ν_{max} ($CHCl_3$)/cm^{-1} 3268, 3008, 2980, 1713, 1643, 1590, 1286; δ_H (400 MHz; $CDCl_3$) 10.37 (1H, br s, NH), 7.20 (1H, t, *J* 8.1, ArH), 6.70-6.64 (3H, m, ArH), 4.64 (1H, s, CH), 3.80 (3H, s, OMe), 2.01 (3H, s, Me), 1.52 (9H, s, *t*Bu); δ_C (75 MHz; $CDCl_3$) 170.4, 160.2, 158.0, 140.7, 129.6 (CH), 116.6 (CH), 110.2 (CH), 109.9 (CH), 88.1 (CH), 82.0, 55.3 (Me), 28.6 (Me), 20.4 (Me); *m/z* (ESI) 264 ($M+H^+$, 100%).

***(Z)-tert*-Butyl 3-(2-morpholinoethylamino)but-2-enoate (7g)**

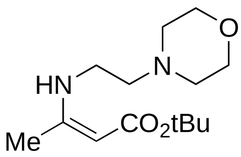

2-Morpholin-4-yl-ethylamine (0.65 g, 5 mmol) was added in a single portion to a stirred suspension of silica gel (0.10 g) and *tert*-butyl acetoacetate (0.79 g, 5 mmol) at room temperature, and the resulting mixture was stirred at room temperature for 18 h. The mixture was diluted with dichloromethane (10 mL), dried ($MgSO_4$), filtered and concentrated *in vacuo* to give the *title compound* as a colorless solid (1.34 g, 99%); mp 63-65 °C; (Found: $M+H^+$, 271.2005. $C_{14}H_{27}N_2O_3$ requires 271.2022); ν_{max} ($CHCl_3$)/cm^{-1} 3008, 2977, 2942, 1642, 1604, 1280, 1153; δ_H (400 MHz; $CDCl_3$) 8.56 (1H, br s, NH), 4.40 (1H, s, CH), 3.74-3.72 (4H, m, OCH_2), 3.31 (2H, q, *J* 6.7, NCH_2), 2.54 (2H, t, *J* 6.7, NCH_2), 2.50-2.48 (4H, m, NCH_2), 1.90 (3H, s, Me), 1.47 (9H, s *t*Bu); δ_C (75 MHz; $CDCl_3$) 170.5, 168.9, 84.4 (CH), 77.9, 66.9 (CH_2), 58.5 (CH_2), 53.7 (CH_2), 40.2 (CH_2), 28.5 (Me), 19.5 (Me); *m/z* (ESI) 271 ($M+H^+$, 100%).

(Z)-Dimethyl 2-(4-methoxybenzylamino)fumarate (7h)

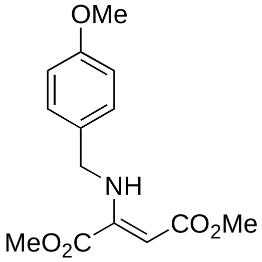

4-Methoxybenzylamine (1.37 g, 10 mmol) was added in a single portion to a stirred solution of dimethyl acetylenedicarboxylate (1.42 g) in methanol (5 mL), and the resulting mixture was stirred at room temperature for 10 min then concentrated *in vacuo*. Column chromatography of the residue eluting with ethyl acetate and light petroleum (1:2.5) gave the *title compound* as a pale yellow solid (1.83 g, 66%); mp 31-33 °C; (Found: $M+Na^+$, 302.0990. $C_{14}H_{17}NO_5Na$ requires 302.0999); ν_{max} ($CHCl_3$)/cm^{-1} 3423, 3305, 3011, 2954, 2911, 2839, 1736, 1662, 1607, 1514; δ_H (400 MHz; $CDCl_3$) 8.32 (1H, br s, NH), 7.21 (2H, d, *J* 8.4, ArH), 6.88 (2H, d, *J* 8.4, ArH), 5.17 (1H, s, CH), 4.50 (2H, d, *J* 6.0, CH_2), 3.80 (6H, s, 2 × OMe), 3.68 (3H, s, OMe); δ_C (75 MHz; $CDCl_3$) 170.5, 164.2, 159.0, 151.2, 130.8, 128.8 (CH), 114.1 (CH), 87.8 (CH), 55.3 (Me), 52.7 (Me), 50.8 (Me), 48.1 (CH_2); *m/z* (ESI) 302 ($M+Na^+$, 100%).

***(Z)*-Methyl 3-(2-(*tert*-butyldimethylsiloxy)ethylamino)but-2-enoate (7i)**

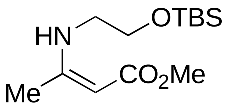

(a) A solution of *tert*-butyldimethylchlorosilane (3.15 g, 21 mmol) in dichloromethane (10 mL) was added dropwise over 3 min to a stirred solution of ethanolamine (1.22 g, 20 mmol) and imidazole (2.72 g, 40 mmol) in dichloromethane (20 mL) at room temperature, and the resulting mixture was stirred at room temperature for 1 h. Water (20 mL) was added, and the phases separated. The aqueous phase was extracted with dichloromethane (2 × 20 mL), and the combined organic phases were dried ($MgSO_4$), filtered and concentrated *in vacuo* to give 2-(*tert*-butyldimethylsiloxy)ethylamine[4] (3.50 g, 100%) as a pale yellow oil (Found: M+Na$^+$, 176.1471. $C_8H_{22}NOSiNa$ requires 176.1465); ν_{max} ($CHCl_3$)/cm^{-1} 2955, 2934, 2858, 1472, 1257; δ_H (400 MHz; $CDCl_3$) 3.64 (2H, t, *J* 5.0, CH_2), 3.05 (2H, br s, NH_2), 2.80 (2H, t, *J* 5.0, CH_2), 0.90 (9H, s, *t*Bu), 0.06 (6H, s, $SiMe_2$); δ_C (75 MHz; $CDCl_3$) 64.7 (CH_2), 44.1 (CH_2), 25.9 (Me), 18.3, -3.4 (Me); *m/z* (ESI) 176 (M+H$^+$, 100%).

(b) 2-(*tert*-Butyldimethylsilyloxy)ethylamine (1.75 g, 10 mmol) was added as a single portion to a stirred suspension of methyl acetoacetate (1.15 g, 10 mmol) and silica gel (1.0 g), and the resulting mixture was stirred at room temperature for 13 h, diluted with dichloromethane (10 mL), filtered and concentrated *in vacuo* to give the *title compound* as a pale yellow oil (2.51 g, 92%); (Found: M+Na$^+$, 296.1641. $C_{13}H_{27}NO_3SiNa$ requires 296.1652); ν_{max} ($CHCl_3$)/cm^{-1} 3008, 2954, 2931, 2859, 1650, 1605; δ_H (400 MHz; $CDCl_3$) 8.62 (1H, br s, NH), 4.46 (1H, s, CH), 3.70 (2H, t, *J* 5.8, OCH_2), 3.62 (3H, s, OMe), 3.34 (2H, q, *J* 5.8, NCH_2), 1.95 (3H, s, Me), 0.90 (9H, s, *t*Bu), 0.07 (6H, s, $SiMe_2$); δ_C (75 MHz; $CDCl_3$) 170.7, 161.9, 82.1 (CH), 63.0 (CH_2), 49.9 (Me), 45.2 (CH_2), 25.9 (Me), 19.5 (Me), 18.0, -3.0 (Me); *m/z* (ESI) 296 (M+Na$^+$, 100%).

(Z)-Methyl 3-(2-(*tert*-butoxycarbonylamino)ethylamino)but-2-enoate (7j)

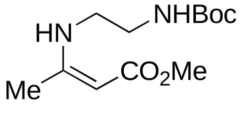

(a) A solution of di-*tert*-butyl dicarbonate (6.3 g, 29 mmol) in 1,4-dioxane (10 mL) was added dropwise over 30 min to a stirred solution of ethylenediamine (10.3 g, 172 mmol) in 1,4-dioxane (5 mL) at room temperature, and the resulting solution was stirred at room temperature for 22 h, then concentrated *in vacuo*. The residue was dissolved in water (100 mL), filtered and the filtrate was extracted with dichloromethane (3 × 50 mL). The combined organic phases were dried ($MgSO_4$), filtered and concentrated *in vacuo* to give *tert*-butyl 2-

aminoethylcarbamate[5] (1.98 g, 43%) as a pale yellow oil; ν_{max} ($CHCl_3$)/cm^{-1} 3631, 3482, 3011, 2945, 2839, 1602, 1240, 1016; δ_H (400 MHz; $CDCl_3$) 4.95 (1H, br s, NH), 3.18-3.15 (2H, m, CH_2), 2.81-2.79 (2H, m, CH_2), 1.44 (9H, s, ^{t}Bu); δ_C (75 MHz; $CDCl_3$) 156.2, 79.2, 43.4 (CH_2), 41.9 (CH_2), 28.4 (Me).

(b) *tert*-Butyl 2-aminoethylcarbamate (1.60 g, 10 mmol) was added as a single portion to a stirred suspension of methyl acetoacetate (1.15 g, 10 mmol) and silica gel (1.0 g), and the resulting mixture was stirred at room temperature for 16 h, diluted with dichloromethane (10 mL), filtered and concentrated *in vacuo* to give the *title compound* as a pale yellow oil (2.58 g, 100%); (Found: M+Na$^+$, 281.1460. $C_{12}H_{22}N_2O_4Na$ requires 281.1477); ν_{max} ($CHCl_3$)/cm^{-1} 3458, 3008, 2982, 1709, 1648, 1606, 1506; δ_H (400 MHz; $CDCl_3$) 8.58 (1H, br s, NH), 4.87 (1H, br s, NH), 4.48 (1H, s, CH), 3.61 (3H, s, OMe), 3.35-3.32 (2H, m, CH_2), 3.26-3.23 (2H, m, CH_2), 1.92 (3H, s, Me), 1.44 (9H, s, *t*Bu); δ_C (75 MHz; $CDCl_3$) 170.9, 162.0, 155.9, 82.7 (CH), 79.6, 50.0 (Me), 41.3 (CH_2), 39.0 (CH_2), 28.3 (Me), 19.3 (Me); *m/z* (ESI) 539 (2M+Na$^+$, 100%), 281 (M+Na$^+$, 31).

(E/Z)-Ethyl 4-(4-methoxybenzyloxy)-3-(methylamino)but-2-enoate (7k)

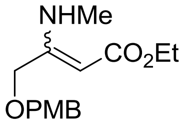

(a) A solution of 4-methoxybenzyl alcohol (5.52 g, 40 mmol) in toluene (10 mL) was added over 5 min to a stirred suspension of NaH (60% on mineral oil; 1.92 g, 48 mmol) in toluene (30 mL) at room temperature. The resulting mixture was stirred for 1.5 h, then ethyl 4-chloroacetoacetate (3.28 g, 20 mmol) was added over 5 min. The resulting mixture was stirred at room temperature for 14 h, quenched by addition of hydrochloric acid (1 M; 10 mL) and diluted with water (30 mL). The aqueous phase was extracted with ethyl acetate (2 × 20 mL), and the combined organic phases were dried ($MgSO_4$), filtered and concentrated *in vacuo*. Chromatography of the residue eluting with light petroleum and ethyl acetate (7:1) gave ethyl 4-(4-methoxybenzyloxy)-3-oxobutanoate as a pale yellow oil (2.46 g, 46%); (Found: M+Na$^+$, 2890.1031. $C_{14}H_{18}O_5Na$ requires 289.1046); ν_{max} ($CHCl_3$)/cm^{-1} 3011, 2939, 1722, 1614, 1514; δ_H (400 MHz; $CDCl_3$) 7.27 (2H, d, *J* 7.6, ArH), 6.90 (2H, d, *J* 7.6, ArH), 4.53 (2H, s, CH_2), 4.19 (2H, q, *J* 7.1, OCH_2), 4.12 (2H, s, CH_2), 3.82 (3H, s, OMe), 3.52 (2H, s, CH_2), 1.28 (3H, t, *J* 7.1, $CH_2C\underline{H}_3$); (75 MHz; $CDCl_3$) 201.8, 167.0, 159.6, 129.6 (CH) 129.0, 114.0 (CH), 74.5

(CH_2), 73.1 (CH_2), 61.4 (CH_2), 55.2 (Me), 46.1 (CH_2), 14.1 (Me); *m/z* (ESI) 555 ($2M+Na^+$, 100%), 289 ($M+Na^+$, 15).

(b) A solution of methylamine (25% w/v in water; 0.59 mL, 4.7 mmol) was added in a single portion to a stirred suspension of silica gel (0.5 g) and **e**thyl 4-(4-methoxybenzyloxy)-3-oxobutanoate (1.142 g, 4.3 mmol) at room temperature, and the resulting mixture was stirred at room temperature for 15 h. The mixture was diluted with dichloromethane (10 mL), dried ($MgSO_4$), filtered and concentrated *in vacuo* to give the *title compound* as a pale yellow oil (1.143 g, 95%, 1:1 *E*:*Z*); (Found: $M+Na^+$, 302.1358. $C_{15}H_{21}NO_4Na$ requires 302.1363); ν_{max} ($CHCl_3$)/cm^{-1} 3429, 3011, 2869, 1654, 1612, 1590, 1515 1251; δ_H (400 MHz; $CDCl_3$) *E* isomer: 7.28 (2H, d, *J* 7.6, ArH), 6.91 (2H, d, *J* 7.6, ArH), 5.72 (1H, br s, NH), 4.82 (2H, s, CH_2), 4.51 (1H, s, CH), 4.14-4.07 (4H, m, 2 × CH_2), 3.83 (3H, s, OMe), 2.75 (3H, d, *J* 4.8, NMe), 1.27 (3H, m, $CH_2C\underline{H}_3$); *Z* isomer: 8.25 (1H, br s, NH), 7.28 (2H, d, *J* 7.6, ArH), 6.91 (2H, d, *J* 7.6, ArH), 4.68 (1H, s, CH), 4.14-4.07 (6H, m, 3 × CH_2), 3.83 (3H, s, OMe), 2.97 (3H, d, *J* 5.2, NMe), 1.27 (3H, m, $CH_2C\underline{H}_3$); δ_C (75 MHz; $CDCl_3$) mixture: 170.7, 168.7, 160.7, 160.3, 159.5, 159.4, 129.6 (CH), 129.5, 128.6 113.9 (CH), 83.1 (CH), 79.1 (CH), 73.2 (CH_2), 71.9 (CH_2), 68.6 (CH_2), 68.3 (CH_2), 58.6 (CH_2), 58.4 (CH_2), 46.1 (Me), 29.7 (Me), 29.6 (Me), 14.7 (Me), 14.1 (Me); *m/z* (ESI) 581 ($2M+Na^+$, 100%), 302 ($M+Na^+$, 38), 280 ($M+H^+$, 14).

3-(Methylamino)cyclohex-2-enone (7l)

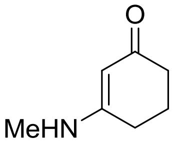

A mixture of methylamine (25 % aq; 1.24 mL, 10 mmol), 1,3-cyclohexanedione (1.12 g, 10 mmol) and toluene (20 mL) was stirred at reflux under Dean-Stark conditions for 6 h and concentrated *in vacuo* to give the *title compound* in sufficiently pure form for the next step as an orange solid (1.25 g, 100 %); mp 66-68 °C (lit.,[6] mp 68-69 °C); ν_{max} ($CHCl_3$)/cm^{-1} 3453, 3001, 1587, 1524, 1193; δ_H (400 MHz; $CDCl_3$) 5.43 (1H, br s, NH), 5.07 (1H, s, CH), 2.78 (3H, d, *J* 4.9, Me), 2.36 (2H, t, *J* 6.1, CH_2), 2.30 (2H, t, *J* 6.1, CH_2), 1.95 (2H, pent, *J* 6.1, CH_2); δ_C (75 MHz; $CDCl_3$) 197.3, 166.2, 96.0, 46.2 (CH_2), 29.5 (Me), 29.4 (CH_2), 8.7 (CH_2).

(Z)-*tert*-Butyl 3-(2-(1H-indol-3-yl)ethylamino)but-2-enoate (7m)

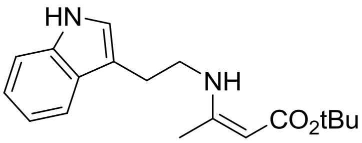

Silica gel (0.5 g) was added to a stirred solution of tryptamine (0.800 g, 5.0 mmol) and *tert*-butyl acetoacetate (0.790 g, 5.0 mmol) in dichloromethane (50 mL). The resulting mixture was stirred for 16 h at room temperature, filtered and concentrated *in vacuo* to give the *title compound* as a pale brown oil (1.450 g, 97 %); (Found: M+H$^+$, 301.1900. $C_{18}H_{25}N_2O_2$ requires 301.1911); δ_H (400 MHz; $CDCl_3$) 8.69 (1H, br s, NH), 8.06 (1H, br s, NH), 7.60 (1H, d, *J* 7.8, ArH), 7.39 (1H, d, *J* 8.1, ArH), 7.23 (1H, dd, *J* 8.1, 7.1, ArH), 7.15 (1H, dd, *J* 7.8, 7.1, ArH), 7.08 (1H, d, *J* 2.2, CH), 4.40 (1H, s, CH), 3.51 (2H, dt, *J* 7.0, 6.4, CH_2N), 3.04 (2H, t, *J* 6.4, CH_2), 1.85 (3H, s, Me), 1.51 (9H, s, tBu); δ_C (75 MHz; $CDCl_3$) 170.3, 161.1, 136.3, 127.2, 122.2 (CH), 122.1 (CH), 119.5 (CH), 118.6 (CH), 112.9, 111.2 (CH), 83.8 (CH), 77.8, 43.7 (CH_2), 28.7 (Me), 26.8 (CH_2), 19.3 (Me); *m/z* (ESI) 301 (M+H$^+$, 86 %), 245 (M-tBu+H$^+$, 100 %).

(S)-(E,Z)-Methyl 2-(4-*tert*-butoxy-4-oxobut-2-en-2-ylamino)-4-methylpentanoate (7n)

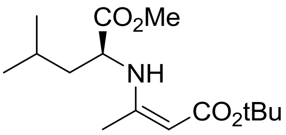

Triethylamine (1.01 g, 10 mmol) was added to a stirred suspension of *tert*-butyl acetoacetate (1.580 g, 10 mmol) and (S)-leucine methyl ester hydrochloride (1.815 g, 10 mmol) in dichloromethane. The resulting suspension was stirred at room temperature for 16 h, filtered through a pad of silica and concentrated *in vacuo* to give the *title compound* as a colorless oil (2.64 g, 93 %); (Found: M+Na$^+$, 308.1823. $C_{15}H_{27}NO_4Na$ requires 308.1838); ν_{max} ($CHCl_3$)/cm^{-1} 3287, 2962, 2873, 1737, 1613, 1438; δ_H (400 MHz; $CDCl_3$) 8.56 (1H, br d, *J* 9.0, NH), 4.47 (1H, s, CH), 4.12 (1H, ddd, *J* 7.1, 9.0, 7.5, CH); 3.75 (3H, s, OMe), 1.87 (3H, s, Me), 1.81-1.36 (3H, m, CH_2 + CH), 1.48 (9H, s, tBu), 0.99 (3H, d, *J* 6.4, Me), 0.94 (3H, d, *J* 6.4, Me); δ_C (75 MHz; $CDCl_3$) 173.4, 170.1, 159.2, 86.6 (CH), 78.2, 54.5 (CH), 52.3 (Me), 42.1 (CH_2), 28.6 (Me), 24.6 (CH), 22.7 (Me), 21.9 (Me), 19.5 (Me); *m/z* (ESI) 308 (M+Na$^+$, 100 %).

(±)-(Z)-Menthyl 3-(2-(1H-indol-3-yl)ethylamino)but-2-enoate (7o)

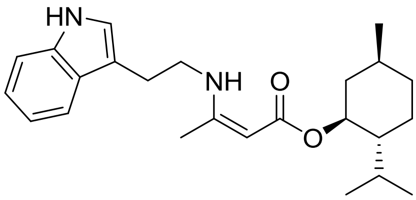

(a) 2,2,6-Trimethyl-4*H*-1,3-dioxin-4-one (2.19 mL, 16.5 mmol) was added to a stirred solution of (±)-menthol (2.34 g, 15 mmol) in xylenes (10 mL) at room temperature, and the resulting mixture was stirred at reflux for 3 h. The solvent was removed *in vacuo*, and column chromatography eluting with light petroleum and ethyl acetate (1:0 then 14:1) gave (±)-menthyl acetoacetate[7] as a pale orange liquid (3.57 g, 99 %); δ_H (400 MHz; $CDCl_3$) 4.77 (1H, dtt, *J* 10.9, 4.4, 2.4, CH), 3.45 (2H, s, CH_2), 2.29 (3H, s, Me), 2.07-1.88 (3H, m, 3 × CH), 1.73-1.69 (2H, m, CH_2), 1.52 (1H, qqd, *J* 7.0, 6.4, 2.6, CH), 1.40 (1H, tdd, *J* 12.1, 11.1, 3.0, CH), 1.13-0.97 (2H, m, 2 CH), 0.93 (3H, d, *J* 6.4, Me), 0.92 (3H, d, *J* 7.0, Me), 0.79 (3H, d, *J* 6.9, Me); δ_C (75 MHz; $CDCl_3$) 200.7, 166.7, 75.5 (CH), 50.6 (CH_2), 46.9 (CH), 40.7 (CH_2), 34.2 (CH_2), 31.4 (CH), 30.1 (Me), 26.1 (CH), 23.3 (CH_2), 22.0 (Me), 20.7 (Me), 16.2 (Me).

(b) Silica gel (0.5 g) was added to a stirred solution of tryptamine (0.800 g, 5.0 mmol) and menthyl acetoacetate (1.200 g, 5.0 mmol) in dichloromethane (50 mL). The resulting mixture was stirred for 16 h at room temperature, filtered and concentrated *in vacuo* to give the *title compound* as a pale brown solid (1.880 g, 98 %); mp 119-121 °C; (Found: $M+H^+$, 383.2712. $C_{24}H_{35}N_2O_2$ requires 383.2693); ν_{max} ($CHCl_3$)/cm^{-1} 3481, 3011, 2958, 2958, 2870, 1638, 1603, 1174; δ_H (400 MHz; $CDCl_3$) 8.73 (1H, br s, NH), 8.05 (1H, br s, NH), 7.60 (1H, dd, *J* 7.5, 0.8, ArH), 7.39 (1H, dt, *J* 8.0, 1.0, ArH), 7.23 (1H, ddd, *J* 7.5, 7.1, 1.0, ArH), 7.15 (1H, ddd, *J* 8.0, 7.1, 0.8, ArH), 7.10 (1H, d, *J* 3.4, ArH), 4.66 (1H, dt, *J* 10.9, 4.4, CH), 4.44 (1H, s, CH), 3.53 (2H, td, *J* 7.1, 6.1, NCH_2), 3.05 (2H, t, *J* 7.1, CH_2), 2.07-1.92 (2H, m, 2 CH), 1.87 (3H, s, Me), 1.74-1.65 (2H, m, CH_2), 1.57-1.47 (1H, m, CH), 1.41-1.33 (1H, m, CH), 1.15-0.87 (3H, m, 3 CH), 0.93 (3H, d, *J* 3.2, Me), 0.90 (3H, d, *J* 3.7, Me), 0.81 (3H, d, *J* 6.9, Me); δ_C (75 MHz; $CDCl_3$) 170.3, 161.6, 136.3, 127.2, 122.2 (CH), 122.1 (CH), 119.5 (CH), 118.5 (CH), 112.8, 111.2 (CH), 82.5 (CH), 71.6 (CH), 47.3 (CH), 43.7 (CH_2), 41.6 (CH_2), 34.5 (CH_2), 31.5 (CH), 26.7 (CH_2), 26.2 (CH), 23.7 (CH_2), 22.1 (Me), 20.8 (Me), 19.4 (Me), 16.6 (Me); *m/z* (ESI) 383 ($M+H^+$, 100 %)

(Z)-3-(4-Methoxybenzylamino)-1-morpholinobut-2-en-1-one (7p)

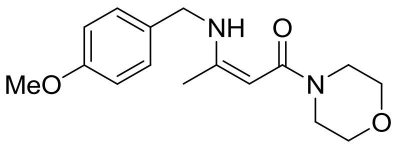

(a) 2,2,6-Trimethyl-4*H*-1,3-dioxin-4-one (1.99 mL, 15 mmol) was added to a stirred solution of morpholine (1.30 mL, 15 mmol) in toluene (10 mL) at room temperature, and the resulting mixture was stirred at reflux for 2.5 h. The solvent was removed *in vacuo*, and column chromatography eluting with light petroleum and ethyl acetate (2:1) gave 1-morpholinobutane-1,3-dione[7] as a pale yellow solid, as a mixture of keto and enol forms (1.142 g, 45 %); mp 48-50 °C (lit.,[7] viscous oil); ν_{max} ($CHCl_3$)/cm^{-1} 3011, 2863, 1723, 1641, 1440; δ_H (400 MHz; $CDCl_3$) 3.71-3.64 (6H, m, 2 CH_2 + 2CH), 3.58 (2H, s, CH_2), 3.43 (2H, dd, *J* 5.2, 4.6, 2CH); 2.30 (3H, s, Me); δ_C (75 MHz; $CDCl_3$) 202.1, 175.6, 170.8, 165.0, 86.2 (CH), 66.7 (CH_2), 66.6 (CH_2), 49.9 (CH_2), 46.8 (CH_2), 42.2 (CH_2), 30.3 (Me), 22.0 (Me).

(b) Silica gel (0.5 g) was added to a stirred solution of 4-methoxybenzylamine (0.685 g, 5.0 mmol) and 1-morpholinobutane-1,3-dione (0.855 g, 5.0 mmol) in dichloromethane (10 mL). The resulting mixture was stirred for 15 h at room temperature, filtered and concentrated *in vacuo* to give the *title compound* as a pale yellow oil (1.440 g, 100 %); (Found: $M+H^+$, 291.1697. $C_{16}H_{23}N_2O_3$ requires 291.1703); ν_{max} ($CHCl_3$)/cm^{-1} 3010, 2861, 1607, 1513, 1246; δ_H (400 MHz; $CDCl_3$) 9.89 (1H, br s, NH), 7.22 (2H, d, *J* 8.5, ArH), 6.88 (2H, d, *J* 8.5, ArH), 4.64 (1H, s, CH), 4.38 (2H, d, *J* 6.3, CH_2), 3.81 (3H, s, OMe), 3.69 (4H, t, *J* 5.0, CH_2), 3.52 (4H, t, *J* 5.0, CH_2), 1.96 (3H, s, Me); δ_C (75 MHz; $CDCl_3$) 170.1, 160.3, 158.8, 131.1, 128.0 (CH), 114.1 (CH), 81.2 (CH), 66.7 (CH_2), 55.3 (Me), 46.1 (CH_2), 43.4 (CH_2), 20.0 (Me); *m/z* (ESI) 291 ($M+H^+$, 100 %).

(Z)-Methyl 3-(2,2-diethoxyethylamino)-3-phenylpropenoate (7q)

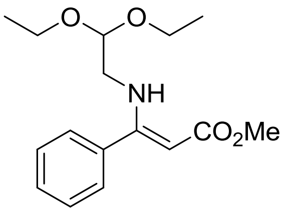

Methyl phenylpropiolate (0.800 g, 5.0 mmol) was added as as single portion to a stirred solution of aminoacetaldehyde diethyl acetal (0.665 g, 5.0 mmol) in ethanol (10 mL) and the resulting mixture was stirred at reflux for 6 h and concentrated *in vacuo*. Column chromatography eluting with light petroleum and ethyl acetate (24:1) gave the *title compound* as a colorless oil (1.364 g, 93 %); (Found: $M+Na^+$, 316.1507. $C_{16}H_{23}NO_4Na$ requires

316.1519); ν_{max} ($CHCl_3$)/cm^{-1} 3295, 3011, 2981, 1651, 1611, 1595, 1482, 1305; δ_H (400 MHz; $CDCl_3$) 8.61 (1H, br s, NH), 7.42-7.29 (5H, m, 5 ArH), 4.66 (1H, s, CH), 4.46 (1H, t, *J* 5.6, CH), 3.71 (3H, s, OMe), 3.67 (2H, dt, *J* 9.3, 7.0, 2 CH), 3.52 (2H, dt, *J* 9.3, 7.0, 2 CH), 3.20 (2H, dd, *J* 6.2, 5.6, NCH_2), 1.22 (6H, t, *J* 7.0, Me); δ_C (75 MHz; $CDCl_3$) 170.4, 164.5, 136.2, 129.2 (CH), 128.4 (CH), 127.9 (CH), 101.8 (CH), 85.8 (CH), 62.8 (CH_2), 50.3 (Me), 47.2 (CH_2), 15.3 (Me); *m/z* (ESI) 316 (M+Na$^{+,}$ 100 %).

5,5-Dimethyl-3-(4-nitrophenylamino)cyclohex-2-enone (7r)[8]

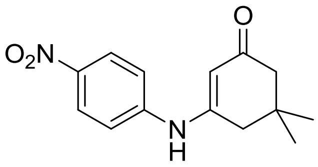

A mixture of 4-nitroaniline (1.38 g, 10 mmol), dimedone (1.40 g, 10 mmol), 4-toluenesulfonic acid monohydrate (0.095 g, 0.5 mmol) and toluene (30 mL) was stirred at reflux under Dean-Stark conditions for 6 h, cooled to room temperature and filtered. The solid was recrystallised from acetonitrile (approx. 130 mL) to give the *title compound* as a bright green-yellow solid (1.83 g, 70 %); mp 240-242 °C; ν_{max} (DMSO-d_6)/cm^{-1} 3691, 2964, 1590, 1507, 1343; δ_H (400 MHz; $CDCl_3$) 9.33 (1H, br s, NH), 8.22 (2H, d, *J* 9.2, ArH), 7.38 (2H, d, *J* 9.2, ArH), 5.68 (1H, s, CH), 2.45 (2H, s, CH_2), 2.14 (2H, s, CH_2), 1.04 (6H, s, Me); δ_C (75 MHz; DMSO-d_6) 197.1, 157.9, 146.8, 142.1, 125.8 (CH), 120.8 (CH), 101.7 (CH), 50.6 (CH_2), 42.6 (CH_2), 32.7, 28.3 (Me).

(Z)-Methyl 3-(cyclohexylamino)pent-2-enoate (7s)

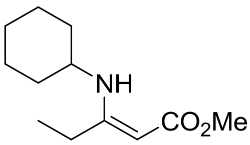

Silica gel (1.0 g) was added to a stirred solution of cyclohexylamine (0.99 g, 10.0 mmol) and methyl propionylacetate (1.30 g, 10.0 mmol) in dichloromethane (10 mL). The resulting mixture was stirred for 15 h at room temperature, filtered and concentrated *in vacuo* to give the *title compound* as a colorless oil (2.04 g, 97 %); (Found: M+Na$^+$, 234.1474. $C_{12}H_{21}NO_2Na$ requires 234.1465); ν_{max} ($CHCl_3$)/cm^{-1} 3281, 3011, 2980, 2937, 2857, 1643, 1601, 1274; δ_H (400 MHz; $CDCl_3$) 8.66 (1H, br s, NH), 4.46 (1H, s, CH), 3.65 (3H, s, OMe), 3.39-3.31 (1H, m, CH), 2.26 (2H, q, *J* 7.5, CH_2), 1.91-1.76 (4H, m, 2 CH_2), 1.62-1.57 (1H, m, CH), 1.38-1.27 (5H, m, 2 CH_2 + CH), 1.17 (3H, t, *J* 7.5, Me); δ_C (75 MHz; $CDCl_3$) 171.3, 166.4, 79.3 (CH),

50.9 (CH), 49.9 (Me), 34.5 (CH_2), 25.4 (CH_2), 25.2 (CH_2), 24.7 (CH_2), 12.8 (Me); *m/z* (ESI) 212 ($M+Na^+$, 100 %).

(Z)-Methyl 3-(2-*tert*-butoxy-2-oxoethylamino)but-2-enoate (7t)

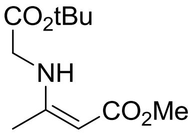

Merthyl acetoacetate (1.16 g, 10.0 mmol) was added to a stirred suspension of glycine *tert*-butyl ester hydrochloride (1.67 g, 10.0 mmol) and triethylamine (1.01 g, 10 mmol) in dichloromethane (10 mL). The resulting mixture was stirred at room temperature for 16 h, washed with water (20 mL), dried ($MgSO_4$), filtered and concentrated to give the *title compound* as a colorless solid (2.18 g, 95 %); mp 40-42 °C; (Found: $M+Na^+$, 252.1210. $C_{11}H_{19}NO_4Na$ requires 252.1206); ν_{max} ($CHCl_3$)/cm^{-1} 2983, 1741, 1655, 1606, 1150; δ_H (400 MHz; $CDCl_3$) 8.80 (1H, br s, NH), 4.55 (1H, s, CH), 3.88 (2H, d, *J* 6.1, CH_2), 3.63 (3H, s, OMe), 1.88 (3H, s, Me), 1.48 (9H, s, *t*Bu); δ_C (75 MHz; $CDCl_3$) 170.6, 168.8, 160.7, 83.8 (CH), 82.3, 52.3 (Me), 45.4 (CH_2), 28.0 (Me), 19.3 (Me); *m/z* (ESI) 252 ($M+Na^+$, 100 %).

(E)-4-(Methylamino)pent-3-en-2-one (7u)

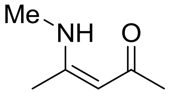

Silica gel (1.0 g) was added to a stirred solution of methylamine (25 % aq; 1.36 mL, 11 mmol) and pentane-1,3-dione (1.00 g, 10.0 mmol). The resulting mixture was stirred for 17 h at room temperature and diluted with dichloromethane (10 mL). The organic phase was collected, dried ($MgSO_4$), filtered and concentrated *in vacuo* to give the *title compound* as a yellow solid (1.06 g, 94 %); mp 38-40 °C (lit.,[9] mp 37-38 °C); ν_{max} ($CHCl_3$)/cm^{-1} 3007, 2826, 1612, 1566, 1528, 1303; δ_H (400 MHz; $CDCl_3$) 10.72 (1H, br s, NH), 4.99 (1H, s, CH), 2.93 (3H, d, *J* 5.4, Me), 2.00 (3H, s, Me), 1.92 (3H, s, Me); δ_C (75 MHz; $CDCl_3$) 194.7, 164.1, 95.1 (CH), 29.43 (Me), 28.7 (Me), 18.7 (Me).

Cholesteryl 3-(2-(dimethylamino)ethylamino)but-2-enoate (7v)

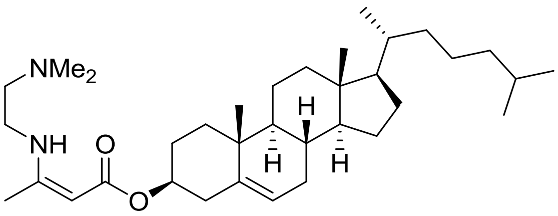

(a) 2,2,6-Trimethyl-4*H*-1,3-dioxin-4-one (2.19 mL, 16.5 mmol) was added to a stirred solution of cholesterol (5.80 g, 15 mmol) in xylenes (10 mL), and the resulting mixture was stirred at reflux for 2.5 h, cooled to room temperature and concentrated. Column chromatography eluting with ethyl acetate and light petroleum (1:19) gave cholesteryl acetoacetate as a colorless solid (7.01 g, 99 %); mp 91-93 ^oC (lit.,[7] mp 92-93 ^oC); (Found: $M+Na^+$, 493.3663. $C_{31}H_{50}O_3Na$ requires 493.3652); δ_H (400 MHz; $CDCl_3$) 5.41 (1H, d, *J* 4.7, CH), 4.74-4.66 (1H, m, CH), 3.45 (2H, s, CH_2), 2.38-2.35 (2H, m), 2.29 (3H, s, Me), 2.07-1.11 (26H, m), 1.04 (3H, s, Me), 0.94 (3H, d, *J* 6.7, Me), 0.90 (3H, d, *J* 6.6, Me), 0.89 (3H, d, *J* 6.7, Me), 0.70 (3H, s, Me); δ_C (75 MHz; $CDCl_3$) 200.7, 166.5, 139.6, 122.9 (CH), 75.1 (CH), 56.7 (CH), 56.1 (CH), 50.5 (CH_2), 50.0 (CH), 42.3, 39.7 (CH_2), 39.5 (CH_2), 37.9 (CH_2), 36.9 (CH_2), 36.6, 36.2 (CH_2), 35.8 (CH), 31.9 (CH_2), 31.8 (CH), 30.1 (Me), 28.2 (CH_2), 28.0 (CH), 27.6 (CH_2), 24.3 (CH_2), 23.8 (CH_2), 22.8 (Me), 22.6 (Me), 21.0 (CH_2), 19.3 (Me), 18.7 (Me), 11.9 (Me); *m/z* (ESI) 493 ($M+Na^+$, 14 %), 369 ($M-[MeCOCH_2CO_2H]^+$, 100).

(b) Silica gel (0.5 g) was added to a stirred solution of N,N-dimethylethylenediamine (0.264 g, 3.0 mmol) and cholesteryl acetoacetate (1.410 g, 3.0 mmol) in dichloromethane (10 mL). The resulting mixture was stirred for 15 h at room temperature, filtered and concentrated *in vacuo.* Column chromatography eluting with ethyl acetate and methanol (14:1) gave the *title compound* as a colorless solid (1.360 g, 84 %); mp 104-106 °C; (Found: $M+H^+$, 541.4757. $C_{35}H_{61}N_2O_2$ requires 541.4728); ν_{max} ($CHCl_3$)/cm^{-1} 2950, 2869, 1641, 1602, 1175; δ_H (400 MHz; $CDCl_3$) 8.60 (1H, br s, NH), 5.37 (1H, d, *J* 5.0, CH), 4.67-4.59 (1H, m, CH), 4.45 (1H, s, CH), 3.31 (2H, dt, *J* 6.6, 5.8, CH_2), 2.48 (2H, t, *J* 6.6, CH_2), 2.34-2.31 (2H, m), 2.28 (6H, s, NMe_2), 2.05-1.98 (2H, m), 1.94 (3H, s, Me), 1.90-1.83 (3H, m), 1.64-1.33 (11H, m), 1.21-0.98 (10H, m), 1.03 (3H, s, Me), 0.93 (3H, d, *J* 6.5, Me), 0.89 (3H, d, *J* 6.7, Me), 0.88 (3H, d, *J* 6.6, Me), 0.69 (3H, s, Me); δ_C (75 MHz; $CDCl_3$) 169.9, 161.3, 140.3, 122.1 (CH), 82.8 (CH), 71.5 (CH), 59.2 (CH_2), 56.7 (CH), 56.2 (CH), 50.1 (CH), 45.6 (Me), 42.3, 41.1 (CH_2), 39.8 (CH_2), 39.5 (CH_2), 38.7 (CH_2), 37.2 (CH_2), 36.6, 36.2 (CH_2), 35.8 (CH), 31.9 (CH_2), 31.8 (CH), 28.3 (CH_2), 28.2 (CH_2), 28.0 (CH), 24.3 (CH_2), 23.8 (CH_2), 22.8 (Me), 22.6 (Me), 21.0 (CH_2), 19.6 (Me), 19.4 (Me), 18.7 (Me), 11.9 (Me); *m/z* (ESI) 541 ($M+H^+$, 100 %).

(Z)-Methyl 3-(2-(*tert*-butyldiphenylsilyloxy)ethylamino)but-2-enoate (7w)

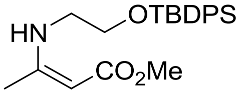

(a) A solution of *tert*-butyl(chloro)diphenylsilane (2.877 g, 10.5 mmol) in dichloromethane (5 mL) was added over 3 minutes to a stirred solution of ethanolamine (0.61 g, 10 mmol) and imidazole (1.36 g, 20 mmol) in dichloromethane (10 mL) at room temperature. The resulting mixture was stirred at room temperature for 3 h, washed with water (20 mL), and the aqueous phase was extracted with dichloromethane (2 x 15 mL). The combined organic phases were dried ($MgSO_4$), filtered and concentrated to give 2-(*tert*-butyldiphenylsilyloxy)ethylamine[10] as a colorless oil (2.99 g, 100 %); (Found: $M+H^+$, 300.1757. $C_{18}H_{26}NOSi$ requires: 300.1778); δ_H (400 MHz; $CDCl_3$) 7.70-7.68 (4H, m, ArH), 7.45-7.41 (6H, m, ArH), 3.71 (2H, t, *J* 5.3, CH_2O), 2.84 (2H, t, *J* 5.3, CH_2N), 2.46 (2H, br s, NH_2), 1.09 (9H, s, *t*Bu); δ_C (75 MHz; $CDCl_3$) 135.6 (CH), 133.6, 129.7 (CH), 127.7 (CH), 65.9 (CH_2), 44.1 (CH_2), 26.9 (Me), 19.3; *m/z* (ESI) 300 ($M+H^+$, 38 %), 222 ($M-Ph^+$, 100).

(b) Silica gel (0.3 g) was added to a stirred solution of 2-(*tert*-butyldiphenylsilyloxy)ethylamine (2.99 g, 10 mmol) and methyl acetoacetate (1.16 g, 10.0 mmol). The resulting mixture was stirred for 16 h at room, filtered and concentrated *in vacuo* to give the *title compound* as a yellow oil (3.94 g, 94 %); (Found: $M+H^+$, 398.2134. $C_{23}H_{32}NO_3Si$ requires: 398.2146); ν_{max} ($CHCl_3$)/cm^{-1} 3293, 3011, 2952, 1650, 1605, 1505, 1177; δ_H (400 MHz; $CDCl_3$) 8.74 (1H, br s, NH), 7.68 (4H, dd, *J* 8.0, 1.5, ArH), 7.45-7.39 (6H, m, ArH), 4.49 (1H, s, CH), 3.72 (2H, t, *J* 5.8, CH_2O), 3.66 (3H, s, Me), 3.38 (2H, dt, *J* 6.0, 5.8, CH_2N), 1.90 (3H, s, Me), 1.08 (9H, 2, *t*Bu); δ_C (75 MHz; $CDCl_3$) 170.7, 161.9, 135.6 (CH), 133.2, 129.8 (CH), 127.8 (CH), 82.3 (CH), 63.7 (CH_2), 49.9 (Me), 45.0 (Me), 26.8 (Me), 26.7 (Me), 19.1; *m/z* (ESI) 398 ($M+H^+$, 100 %).

Preparation of Bromoquinonones

2-Bromo-6-methoxy-1,4-benzoquinone (6a)

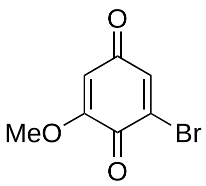

(a) Sodium percarbonate (8.24 g, 52.5 mmol) was added as a single portion to a stirred solution of 5-bromovanillin (11.55 g, 50 mmol) in THF (150 mL) and water (60 mL). The resulting mixture was stirred at room temperature for 5 h, concentrated *in vacuo* to a solid, dissolved in acetone (200 mL), filtered and concentrated *in vacuo*. Chromatography of the residue eluting with light petroleum and ethyl acetate (2:1) gave 2-bromo-6-methoxy-1,4-hydroquinone[11] as a pale grey solid (9.90 g, 90%); mp 139-141 °C; ν_{max} ($CHCl_3$)/cm^{-1} 3597, 3537, 3011, 1612, 1600, 1501; δ_H (300 MHz; acetone-d_6) 8.07 (1H, s, OH), 7.50 (1H, s, OH), 6.59 (1H, d, *J* 2.8, ArH), 6.50 (1H, d, *J* 2.8, ArH), 3.83 (3H, s, OMe); δ_C (75 MHz; acetone-d_6); 150.7, 148.7, 137.2, 110.0 (CH), 108.1, 99.7 (CH), 55.6 (Me).

(b) A solution of iron(III) chloride hexahydrate (61.02 g, 226.0 mmol) in water (300 mL) was added as a single portion to a stirred solution of 2-bromo-6-methoxy-1,4-hydroquinone (9.90 g, 45.2 mmol) in methanol (40 mL). The resulting mixture was stirred at room temperature for 5 h, and the precipitate was collected by filtration and dried *in vacuo* to give the *title compound* as an orange-brown solid (9.76 g, 99%); mp 160-162 °C (lit.,[12] mp 162-163 °C); ν_{max} ($CHCl_3$)/cm^{-1} 3045, 2940, 1696, 1643, 1590; δ_H (300 MHz; $CDCl_3$ 7.22 (1H, d, *J* 2.2, H-3), 5.98 (1H, d, *J* 2.2, H-5), 3.87 (3H, s, OMe); δ_C (75 MHz; $CDCl_3$) 184.6, 174.5, 158.3, 138.5 (CH), 134.3, 107.6 (CH), 56.8 (Me).

2-Bromo-5-methoxy-1,4-benzoquinone (6b)

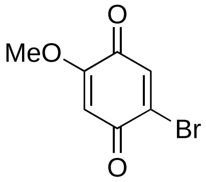

(a) A solution of bromine (1.59 mL, 30.9 mmol) in dichloromethane (50 mL) was added dropwise at 0 °C to a stirred solution of 1,2,4-trimethoxybenzene (4.945 g, 29.4 mmol) in dichloromethane (200 mL) over 1 h. The resulting solution was stirred at 0 °C for 2 min, washed sequentially with saturated aqueous sodium thiosulfate solution (100 mL), saturated aqueous sodium hydrogen carbonate solution (100 mL) and brine (100 mL), dried ($MgSO_4$), filtered and concentrated *in vacuo* to give 1-bromo-2,4,5-trimethoxybenzene as a colorless oil which solidified on standing at room temperature (6.94 g, 96%); mp 52-54 °C (lit.,[13] mp 53-54 °C); δ_H (300 MHz; $CDCl_3$) 7.05 (1H, s, ArH), 6.58 (1H, s, ArH), 3.90 (3H, s, OMe), 3.88 (3H, s, OMe), 3.85 (3H, s, OMe); δ_C (75 MHz; $CDCl_3$) 150.3, 149.1, 143.8, 116.5 (CH),

101.9, 98.9 (CH), 57.2 (Me), 567 (Me), 56.3 (Me); *m/z* (ESI) 271/269 ($M+Na^+$, 100/99.5%), 168 ($M-Br^+$, 90).

(b) A solution of ammonium cerium(IV) nitrate (20.55 g, 37.5 mmol) in water (100 mL) was added in one portion to a stirred solution of 1-bromo-2,4,5-trimethoxybenzene (3.71 g, 15 mmol) in acetonitrile (100 mL) at room temperature. The resulting mixture was stirred at room temperature for 16 h, concentrated to approximately half its volume *in vacuo* and extracted with dichloromethane (2 × 150 mL). The combined organic phases were dried ($MgSO_4$), filtered and concentrated *in vacuo* to give the *title compound* as an orange solid (2.90 g, 89%); mp 185-187 °C (lit.,[12] mp 192-194 °C) δ_H (300 MHz; $CDCl_3$) 7.27 (1H, s, Ar-H), 6.15 (1H, s, ArH), 3.88 (3H, s, OMe); δ_C (75 MHz; $CDCl_3$) 179.2, 159.0, 139.3, 135.8 (CH), 106.9 (CH), 56.7 (Me); 1 C unobserved; *m/z* (ESI) 227 (100), 217 (MH^+, 14%).

2,6-Dibromo-1,4-benzoquinone (6c)

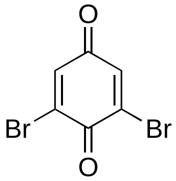

A solution of periodic acid (3.42 g, 15 mmol) in water (20 mL) was added as a single portion to a stirred solution of 2,4,6-tribromophenol (1.98 g, 6 mmol) in acetonitrile (80 mL), and the resulting mixture was stirred at 80 °C for 6 h then cooled to room temperature. The acetonitrile was removed *in vacuo* and the resulting aqueous suspension was extracted with ether (3 × 15 mL). The combined organic phases were dried ($MgSO_4$), filtered and concentrated *in vacuo*. Recrystallisation from ethanol gave the *title compound* as a yellow solid (0.98 g, 61%); mp 128-130 °C (lit.,[14] mp 130 °C); ν_{max} ($CHCl_3$)/cm^{-1} 3067, 1690, 1653, 1608, 1574, 1271; δ_H (300 MHz; $CDCl_3$ 7.35 (2H, s, CH); δ_C (75 MHz; $CDCl_3$) 182.4, 172.4, 138.2 (CH), 135.7.

2-Bromo-6-(*tert*-butoxycarbonylamino)-1,4-benzoquinone (6d)

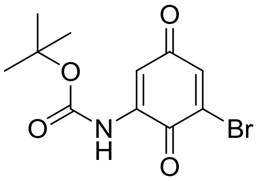

(a) To a mixture of 2-bromo-4-methoxy-6-nitrophenol (1.736 g, 7.0 mmol), acetic acid (15 mL), ethanol (12 mL) and water (3 mL) was added iron powder (2.94 g, 52.5 mmol). The

resulting mixture was stirred at reflux for 2.5 h, cooled to room temperature, diluted with water (75 mL) and saturated aqueous sodium bicarbonate solution (75 mL), filtered and the solid washed with dichloromethane (3 x 50 mL). The filtrate was partitioned, and the aqueous phase was extracted with dichloromethane (3 x 50 mL). The combined organic phases were dried ($MgSO_4$), filtered and concentrated to give the crude aniline as a purple solid. This residue was dissolved in THF (35 mL), and di-*tert*-butyl dicarbonate (1.831 g, 8.4 mmol) was added. The resulting mixture was stirred at reflux for 19 h, cooled to room temperature and concentrated. Column chromatography eluting with ethyl acetate and light petroleum (1:7) gave the Boc-protected aniline as a pale pink solid (1.522 g, 68 % over two steps); mp 91-93 °C; (Found: M+Na$^+$, 340.0133. $C_{12}H_{16}{}^{79}BrNO_4Na$ requires: 340.0155); δ_H (400 MHz; $CDCl_3$) 7.29 (1H, s, CH), 6.96 (1H, br s), 6.75 (1H, s, CH), 5.89 (1H, br s), 3.78 (3H, s, Me), 1.56 (9H, s, *t*Bu); δ_C (75 MHz; $CDCl_3$) 153.9, 153.0, 135.8, 127.7, 111.0 (CH), 110.0, 105.0 (CH), 81.3, 55.9 (Me), 28.3 (Me); ν_{max} ($CHCl_3$)/cm^{-1} 3691, 3536, 3011, 2982, 1725, 1597, 1523, 1465, 1157; *m/z* (ESI) 340/342 (M+Na$^+$, 100/98 %).

(b) To a suspension of the above Boc-protected aniline (1.59 g, 5.0 mmol), methanol (0.625 mL) and water (25 mL) was added bis(trifluoroacetoxy)iodobenzene (2.365 g, 5.5 mmol). The resulting mixture was stirred at room temperature for 17 h and extracted with dichloromethane (3 x 10 mL). The combined organic phases were dried ($MgSO_4$), filtered and concentrated. Column chromatography eluting with ethyl acetate and light petroleum (1:9) gave the *title compound* as an orange solid (1.03 g, 68 %); mp 83-85 °C; (Found: M+Na$^+$, 323.9813. $C_{11}H_{12}{}^{79}BrNO_4Na$ requires: 323.9842); δ_H (400 MHz; $CDCl_3$) 7.47 (1H, br s, NH), 7.29 (1H, d, *J* 1.6, CH), 7.22 (1H, d, *J* 1.6, CH), 1.54 (9H, s, *t*Bu); δ_C (75 MHz; $CDCl_3$) 184.8, 175.9, 150.8, 139.3 (CH), 139.1, 132.4, 112.5 (CH), 83.1, 28.2 (Me); ν_{max} ($CHCl_3$)/cm^{-1} 3385, 1739, 1680, 1641, 1504, 1148; *m/z* (ESI) 324/326 (M+Na$^+$, 100/97 %).

2,5-Dibromo-1,4-benzoquinone (6e)

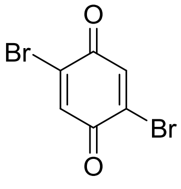

A solution of iron(III) chloride hexahydrate (4.11 g, 15 mmol) in water (15 mL) was added as a single portion to a stirred solution of 2,5-dibromo-1,4-hydroquinone in methanol (15 mL). The resulting mixture was stirred at room temperature for 2 h and the precipitate was collected by filtration and dried *in vacuo* to give the *title compound* as a yellow solid (0.710 g,

89 %); mp 186-188 °C (lit.,[15] mp 189 °C); δ_H (400 MHz; $CDCl_3$) 7.51 (2H, s, CH); δ_C (75 MHz; $CDCl_3$) 177.0, 137.8, 137.1 (CH).

2-Bromo-6-ethyl-1,4-benzoquinone (6f)

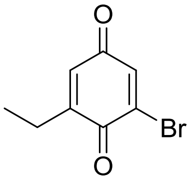

(a) Bromine (2.58 mL, 50 mmol) was added over 5 min to a stirred solution of 2-ethylphenol (3.05 g, 25 mmol) in acetic acid (36 mL) and water (4 mL). The mixture was stirred at room temperature for 1 h, diluted with water (200 mL) and extracted with dichloromethane (3 x 75 mL). The combined organic phases were washed with saturated sodium thiosulfate solution (75 mL) and brine (75 mL), dried ($MgSO_4$), filtered and concentrated to give 2,4-dibromo-6-ethylphenol[16] as a pale brown oil (6.687 g, 96 %); δ_H (400 MHz; $CDCl_3$) 7.46 (1H, d, *J* 2.4, ArH), 7.23 (1H, d, *J* 2.4, ArH), 5.56 (1H, s, OH), 2.69 (2H, q, *J* 7.5, CH_2), 1.24 (3H, t, *J* 7.5, Me); δ_C (75 MHz; $CDCl_3$) 149.4, 133.6, 131.6 (CH), 131.2 (CH), 112.3, 110.7, 23.6 (CH_2), 13.6 (Me); ν_{max} ($CHCl_3$)/cm^{-1} 3522, 2973, 2938, 1459, 1145.

(b) A solution of chromium(VI) oxide (2.591 g, 25.91 mmol) in water (7 mL) was added to a stirred solution of 2,4-dibromo-6-ethylphenol (6.595 g, 23.55 mmol) in acetic acid (12 mL), water (4 mL) and acetonitrile (12 mL), and the mixture was stirred at 60 °C for 1.5 h, cooled to room temperature, diluted with water (100 mL) and extracted with dichloromethane (3 x 50 mL). The combined organic phases were washed with brine (50 mL), dried ($MgSO_4$), filtered and concentrated to give the *title compound* as an orange oil (5.06 g, 100 %); (Found: $M+H^+$, 214.9642. $C_8H_8{}^{79}BrO_2$ requires: 214.9708); δ_H (400 MHz; $CDCl_3$) 7.26 (1H, d, *J* 3.2, CH), 6.61 (1H, dt, *J* 3.2, 1.6, CH), 2.55 (2H, qd, *J* 7.5, 1.6, CH_2), 1.19 (3H, t, *J* 7.5, Me); δ_C (75 MHz; $CDCl_3$) 185.1, 179.7, 150.8, 137.6 (CH), 137.6, 131.8 (CH), 23.1 (CH_2), 1.7 (Me); ν_{max} ($CHCl_3$)/cm^{-1} 3690, 3011, 2976, 1676, 1655, 1602; *m/z* (ESI) 215/217 ($M+H^+$, 39/39 %), 227 (100).

2-Bromo-6-isopropoxy-1,4-benzoquinone (6g)

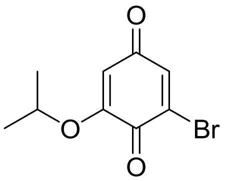

Bromine (2.58 mL, 50 mmol) was added dropwise over 10 min to a stirred solution of *tert*-butylamine (8.00 g, 100 mmol) in toluene (100 mL) at -30 °C. The mixture was stirred at -30 °C for 1 h, cooled to -60 °C, and a solution of 2-isopropoxyphenol (7.60 g, 50 mmol) in dichloromethane (20 mL) was added dropwise over 10 min. The mixture was stirred at room temperature for 16 h, washed with saturated sodium thiosulfate solution (100 mL) and brine (100 mL), dried ($MgSO_4$), filtered and concentrated. Column chromatography eluting with ethyl acetate and light petroleum (1:19) gave an inseparable 5:1 mixture of the *ortho*-brominated phenol and the starting material. A solution of this material and salcomine (1.635 g, 5 mmol) in DMF (200 mL) was stirred vigorously under air for 16 h, diluted with water (800 mL) and extracted with ethyl acetate (3 × 200 mL). The combined organic phases were washed with water (200 mL), dried ($MgSO_4$), filtered and concentrated. Column chromatography eluting with ethyl acetate and light petroleum (1:9) gave the *title compound* as a yellow solid (6.81 g, 56 % over two steps), mp 77-79 °C; (Found: M+Na^+, 266.9610. $C_9H_9{}^{79}BrO_3Na$ requires: 266.9633); δ_H (400 MHz; $CDCl_3$) 7.19 (1H, d, *J* 2.3, CH), 5.93 (1H, d, *J* 2.3, CH), 4.50 (1H, sep, *J* 6.1, OCH), 1.43 (6H, d, *J* 6.1, Me); δ_C (75 MHz; $CDCl_3$) 185.0, 174.8, 156.5, 138.2 (CH), 134.4, 108.1 (CH), 73.3 (CH), 21.1 (Me); ν_{max} ($CHCl_3$)/cm^{-1} 2987, 1693, 1640, 1583; *m/z* (ESI) 267/269 (M+Na^+, 72/75 %), 203/205 (M-$C_3H_5{}^+$, 93/100).

6-bromo-2,3-dihydrobenzo[*b*][1,4]dioxine-5,8-dione (6h)

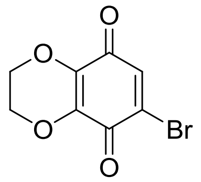

(a) To a flask containing 2,3-dihydroxybenzaldehyde (6.90 g, 50 mmol), potassium carbonate (20.70 g, 150 mmol) and copper(II) oxide (0.20 g, 2.5 mmol) was added a solution of 1,2-dibromoethane (10.34 g, 55 mmol) in DMF (100 mL), and the resulting suspension was stirred at reflux for 14 h, then cooled to room temperature. Water (500 mL) was added, and the mixture was extracted with ethyl acetate (4 × 200 mL). The combined organic phases were washed with water (200 mL), dried ($MgSO_4$), filtered and concentrated. Column chromatography eluting with ethyl acetate and light petroleum (1:5) gave 2,3-dihydrobenzo[*b*][1,4]dioxine-5-carbaldehyde as a colorless solid (4.17 g, 51 %); mp 58-60 ^oC; (Found: M+Na^+, 165.0545. $C_9H_9O_3$ requires: 165.0546); δ_H (400 MHz; $CDCl_3$) 10.39 (1H, s, CHO), 7.41 (1H, dd, *J* 7.9, 1.6, ArH), 7.12 (1H, dd, *J* 7.6, 1.6, ArH), 6.93 (1H, dd, *J* 7.9, 7.6, ArH), 4.42-4.33 (4H, m, 2 x CH_2); δ_C (75 MHz; $CDCl_3$) 189.0 (CH), 146.5, 144.1, 125.1,

123.1 (CH), 121.0 (CH), 120.9 (CH), 64.6 (CH_2), 63.9 (CH_2); *m/z* (ESI) 187 ($M+Na^+$, 85 %), 165 ($M+H^+$, 100).

(b) 3-Chloroperoxybenzoic acid (70 %; 8.45 g, 34.26 mmol) was added portionwise over 1 min to a stirred solution of the above aldehyde (4.68 g, 28.55 mmol) in dichloromethane (50 mL) at room temperature, and the resulting mixture was stirred at reflux for 14 h, cooled to room temperature and washed with saturated aqueous sodium bicarbonate (3 × 30 mL), dried ($MgSO_4$), filtered and concentrated to give the crude formate ester. This was dissolved in methanol (20 mL), and a solution of potassium hydroxide (4.12 g, 73.6 mmol) in water (40 mL) was added at room temperature. The mixture was stirred at room temperature for 2.5 h, cooled to 0 ^oC and acidified to pH 1 with hydrochloric acid (1M), then extracted with ether (3 × 40 mL). The combined organic phases were washed with brine (40 mL), dried ($MgSO_4$), filtered and concentrated. Flash column chromatography eluting with ethyl acetate and light petroleum (1:7) gave 2,3-dihydrobenzo[*b*][1,4]dioxin-5-ol as a pale yellow oil (2.14 g, 84 %); δ_H (400 MHz; $CDCl_3$) 6.75 (1H, t, *J* 8.2, ArH), 6.55 (1H, dd, *J* 8.2, 1.5, ArH), 6.48 (1H, dd, *J* 8.2, 1.5, ArH), 5.41 (1H, br s, OH), 4.35-4.29 (4H, m, 2 × CH_2); δ_C (75 MHz; $CDCl_3$) 145.4, 143.9, 131.3, 120.8 (CH), 108.9 (CH), 107.6 (CH), 64.7 (CH_2), 64.4 (CH_2); ν_{max} ($CHCl_3$)/cm^{-1} 3552, 2011, 2987, 2933, 2883, 1606, 1499, 1489.

(c) A solution of bromine (2.716 mL, 52.63 mmol) in dichloromethane (20 mL) was added dropwise over 20 min to a stirred solution of the above phenol (3.902 g, 25.67 mmol) in dichloromethane (60 mL) at 0 ^oC. The resulting mixture was stirred at 0 ^oC for 1 h, washed with saturated aqueous sodium thiosulfate (2 × 50 mL) and brine (50 mL), dried ($MgSO_4$), filtered and concentrated to give 6,8-dibromo-2,3-dihydrobenzo[b][1,4]dioxin-5-ol as a colorless solid (7.588 g, 95 %); %); mp 77-79 °C; (Found: $M+Na^+$, 330.8565. $C_8H_6{}^{79}Br_2O_3Na$ requires: 330.8581); δ_H (400 MHz; $CDCl_3$) 7.25 (1H, s, ArH), 5.60 (1H, br s, OH), 4.40-4.34 (4H, m, 2 × CH_2); δ_C (75 MHz; $CDCl_3$) 142.0, 140.5, 132.7, 126.2 (CH), 101.2, 100.2, 64.8 (CH_2), 64.5 (CH_2); ν_{max} ($CHCl_3$)/cm^{-1} 3533, 3011, 2993, 1466, 1446; *m/z* (ESI) 331/333/335 ($M+Na^+$, 54/100/52 %).

(d) A solution of chromium(VI) oxide (2.308 g, 23.08 mmol) in water (5 mL) was added as a single portion to a stirred solution of the above dibromophenol (6.504 g, 20.98 mmol) in acetonitrile (10 mL) and acetic acid (10 mL) at room temperature. The resulting mixture was stirred at 60 ^oC for 5 h, cooled to room temperature, diluted with water (100 mL) and extracted with dichloromethane (3 × 40 mL). The combined organic phases were dried ($MgSO_4$), filtered and concentrated to give the *title compound* as a red solid (4.11 g, 80 %);

mp 115-117 °C (dec); (Found: M+Na$^+$, 266.9268. $C_8H_5{}^{79}BrO_4Na$ requires: 266.9263); δ_H (400 MHz; $CDCl_3$) 7.12 (1H, s, CH), 4.38 (4H, br s, 2 × CH_2); δ_C (75 MHz; $CDCl_3$) 178.6, 173.6, 139.2, 138.8, 134.9 (CH), 134.6, 65.0 (CH_2), 64.9 (CH_2); ν_{max} ($CHCl_3$)/cm^{-1} 3006, 2949, 1678, 1666, 1641, 1586; *m/z* (ESI) 267/269 (M+Na$^+$, 90/98 %), 187 (100).

Preparation of indolequinones

General Procedure for the Synthesis of Indolequinones

A solution of enamine (0.5-4.0 equiv) in acetonitrile (5-10 mL/mmol) was added to a mixture of bromoquinone (1.0 equiv), copper(II) acetate monohydrate (1.5 equiv) and potassium carbonate (3.0 equiv). The resulting mixture was stirred at reflux for the indicated time, cooled to room temperature and diluted with dichloromethane (20 mL/mmol), filtered through Celite and concentrated *in vacuo*. Column chromatography of the residue gave the indolequinone.

Methyl 6-methoxy-1,2-dimethyl-4,7-dioxo-4,7-dihydro-1*H*-indole-3-carboxylate (8)

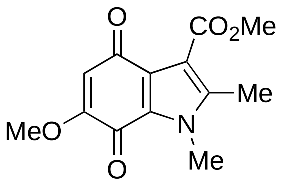

Prepared by the general procedure from quinone **6a** (0.109 g, 0.5 mmol), enamine **7a** (0.065 g, 0.5 mmol), copper(II) acetate monohydrate (0.150 g, 0.75 mmol) and potassium carbonate (0.207 g, 1.5 mmol) stirred at reflux in acetonitrile (5 mL) for 3.5 h. Column chromatography eluting with ethyl acetate and light petroleum (1:1) gave the *title compound* as a yellow solid (0.117 g, 89%); mp 209-211 °C; (Found: M+Na$^+$, 286.0676. $C_{13}H_{13}NO_5Na$ requires 286.0686); ν_{max} ($CHCl_3$)/cm^{-1} 3620, 3007, 2976, 1447, 1248, 1046; δ_H (400 MHz; $CDCl_3$) 5.71 (1H, s, 5-H), 3.93 (3H, s, Me), 3.91 (3H, s, Me), 3.82 (3H, s, Me), 2.45 (3H, s, Me); δ_C (75 MHz; $CDCl_3$) 181.3, 172.3, 164.7, 158.7, 142.6, 127.8, 124.5, 112.9, 107.5 (CH), 56.6 (Me), 52.0 (Me), 32.8 (Me), 10.9 (Me); *m/z* (ESI) 549 (2M+Na$^+$, 100), 286 (M+Na$^+$, 50).

Methyl 5-methoxy-1,2-dimethyl-4,7-dioxo-4,7-dihydro-1*H*-indole-3-carboxylate (10)

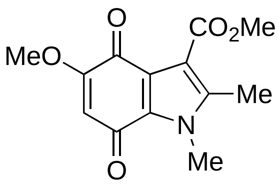

Prepared by the general procedure from quinone **6b** (0.109 g, 0.5 mmol), enamine **7a** (0.260 g, 2.0 mmol), copper(II) acetate monohydrate (0.150 g, 0.75 mmol) and potassium carbonate (0.207 g, 1.5 mmol) stirred at reflux in acetonitrile (5 mL) for 7 h. Column chromatography eluting with ethyl acetate and dichloromethane (1:14) gave the *title compound* as an orange solid (0.064 g, 49%); mp 212-214 °C; (Found: M+Na$^+$, 286.0685. $C_{13}H_{13}NO_5Na$ requires 286.0686); ν_{max} ($CHCl_3$)/cm^{-1} 3012, 1687, 1642, 1608, 1239; δ_H (400 MHz; $CDCl_3$) 5.67 (1H, s, 6-H), 3.95 (3H, s, Me), 3.92 (3H, s, Me), 3.84 (3H, s, Me), 2.48 (3H, s, Me); δ_C (75 MHz; $CDCl_3$) 179.1, 175.5, 164.8, 160.1, 141.6, 129.5, 121.7, 112.5, 105.9 (CH), 56.6 (Me), 51.8 (Me), 32.5 (Me), 10.7 (Me); *m/z* (ESI) 549 (2M+Na$^+$, 100%), 286 (M+Na$^+$, 47).

Methyl 6-methoxy-1-methyl-4,7-dioxo-4,7-dihydro-1*H*-indole-3-carboxylate (11)

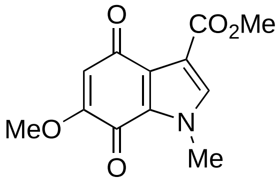

Prepared by the general procedure from quinone **6a** (0.109 g, 0.5 mmol), enamine **7b** (0.058 g, 0.5 mmol), copper(II) acetate monohydrate (0.150 g, 0.75 mmol) and potassium carbonate (0.207 g, 1.5 mmol) stirred at reflux in acetonitrile (5 mL) for 20 h. Column chromatography eluting with ethyl acetate and dichloromethane (1:14) gave the *title compound* as an orange solid (0.080 g, 64%); mp 229-231 °C; (Found: M+Na$^+$, 272.0529. $C_{12}H_{11}NO_5Na$ requires 272.0529); ν_{max} ($CHCl_3$)/cm^{-1} 3002, 2953, 1734, 1671, 1655, 1610; δ_H (400 MHz; $CDCl_3$) 7.45 (1H, s, 2-H), 5.83 (1H, s, 5-H), 4.03 (3H, s, Me), 3.90 (3H, s, Me), 3.84 (3H, s, Me); δ_C (75 MHz; $CDCl_3$) 180.9, 173.1, 162.6, 158.3, 135.8 (CH), 129.7, 124.9, 114.5, 108.7 (CH), 56.6 (Me), 52.0 (Me), 37.3 (Me); *m/z* (ESI) 521 (2M+Na$^+$, 98%), 272 (M+Na$^+$, 100).

Ethyl 2-(allyloxymethyl)-6-methoxy-1-methyl-4,7-dioxo-4,7-dihydro-1*H*-indole-3-carboxylate (12)

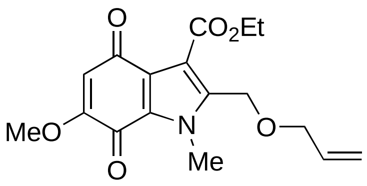

Prepared by the general procedure from quinone **6a** (1.09 g, 5.0 mmol), enamine **7c** (1.00 g, 5.0 mmol), copper(II) acetate monohydrate (1.50 g, 7.5 mmol) and potassium carbonate (2.07 g, 15.0 mmol) stirred at reflux in acetonitrile (50 mL) for 16 h. Column chromatography eluting with ethyl acetate and dichloromethane (1:13) gave the *title compound* as an orange solid (1.07 g, 64%); mp 161-163 °C; (Found: M+Na$^+$, 356.1112. $C_{17}H_{19}NO_6Na$ requires 356.1105); ν_{max} ($CHCl_3$)/cm^{-1} 3008, 2985, 2940, 1707, 1672, 1654, 1609; δ_H (400 MHz; $CDCl_3$) 5.92-5.85 (1H, m, CH), 5.75 (1H, s, 5-H), 5.28 (1H, d, *J* 17.2, CH), 5.21 (1H, d, *J* 10.8, CH), 4.71 (2H, s, OCH_2), 4.38 (2H, q, *J* 7.0, OCH_2), 4.04 (3H, s, Me), 4.02 (2H, d, *J* 5.6, OCH_2), 3.82 (3H, s, Me), 1.40 (3H, t, *J* 7.0, $CH_2C\underline{H}_3$); δ_C (75 MHz; $CDCl_3$) 181.0, 172.8, 163.8, 158.8, 139.8, 133.8 (CH), 128.7, 123.8, 118.2 (CH_2), 115.4, 107.9 (CH), 71.2 (CH_2), 61.4 (CH_2), 60.3 (CH_2), 56.6 (Me), 33.5 (Me), 14.1 (Me); *m/z* (ESI) 689 (2M+Na$^+$, 100%), 356 (M+Na$^+$, 75).

***tert*-Butyl 6-methoxy-1,2-dimethyl-4,7-dioxo-4,7-dihydro-1*H*-indole-3-carboxylate (13)**

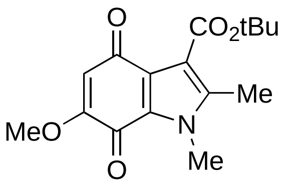

Prepared by the general procedure from quinone **6a** (0.217 g, 1.0 mmol), enamine **7d** (0.173 g, 1.0 mmol), copper(II) acetate monohydrate (0.299 g, 1.5 mmol) and potassium carbonate (0.414 g, 3.0 mmol) stirred at reflux in acetonitrile (10 mL) for 6 h. Column chromatography eluting with ethyl acetate and dichloromethane (1:19) gave the *title compound* as a bright yellow solid (0.221 g, 72%); mp 154-156 °C; (Found: M+Na$^+$, 328.1147. $C_{16}H_{19}NO_5Na$ requires 328.1155); ν_{max} ($CHCl_3$)/cm^{-1} 3008, 2981, 1708, 1651, 1609, 1151; δ_H (400 MHz; $CDCl_3$) 5.70 (1H, s, 5-H), 3.93 (3H, s, Me), 3.82 (3H, s, Me), 2.43 (3H, s, Me), 1.62 (9H, s, *t*Bu); δ_C (75 MHz; $CDCl_3$) 181.2, 172.3, 163.6, 158.8, 141.6, 127.4, 124.4, 115.3, 107.3 (CH), 81.8, 56.5 (Me), 32.7 (Me), 28.1 (Me), 10.8 (Me); *m/z* (ESI) 633 (2M+Na$^+$, 100%), 328 (M+Na$^+$, 43).

Methyl 1,2-dimethyl-4,9-dioxo-4,9-dihydro-1*H*-benzo[*f*]indole-3-carboxylate (14)

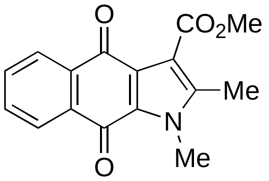

Prepared by the general procedure from quinone **6i** (0.237 g, 1.0 mmol), enamine **7a** (0.129 g, 1.0 mmol), copper(II) acetate monohydrate (0.299 g, 1.5 mmol) and potassium carbonate (0.414 g, 3.0 mmol) stirred at reflux in acetonitrile (10 mL) for 4.5 h. Column chromatography eluting with ethyl acetate and dichloromethane (1:19) gave the *title compound* as a bright yellow solid (0.201 g, 71%); mp 135-137 °C; (Found: $M+Na^+$, 306.0729. $C_{16}H_{13}NO_4Na$ requires 306.0737); ν_{max} ($CHCl_3$)/cm^{-1} 3008, 2954, 1711, 1655, 1505, 1471, 1277; δ_H (400 MHz; $CDCl_3$) 8.17-8.13 (2H, m, ArH), 7.70-7.68 (2H, m, ArH), 4.06 (3H, s, Me), 3.98 (3H, s, Me), 2.49 (3H, s, Me); δ_C (75 MHz; $CDCl_3$) 179.5, 176.5, 165.1, 142.5, 133.8, 133.3 (CH), 133.1, 133.0 (CH), 130.6, 126.8 (CH), 126.2 (CH), 125.6, 113.4, 52.1 (Me), 33.0 (Me), 11.0 (Me); *m/z* (ESI) 589 ($2M+Na^+$, 100%), 306 ($M+Na^+$, 35).

3-Acetyl-6-methoxy-1,2-dimethyl-1*H*-indole-4,7-dione (15)

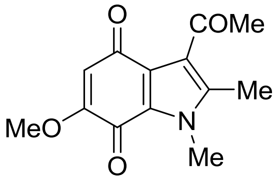

Prepared by the general procedure from quinone **6a** (0.217 g, 1.0 mmol), enamine **7u** (0.113 g, 1.0 mmol), copper(II) acetate monohydrate (0.399 g, 2.0 mmol) and potassium carbonate (0.414 g, 3.0 mmol) stirred at reflux in acetonitrile (20 mL) for 3 h. Column chromatography eluting with ethyl acetate and dichloromethane (1:19) gave the *title compound* as an orange solid (0.226 g, 91 %); mp 220-222 ^oC; (Found: $M+Na^+$, 270.0743. $C_{13}H_{13}NO_4Na$ requires 270.0737); ν_{max} ($CHCl_3$)/cm^{-1} 3011, 1650, 1610, 1186; δ_H (400 MHz; $CDCl_3$) 5.74 (1H, s, CH), 3.96 (3H, s, Me), 3.86 (3H, s, Me), 2.68 (3H, s, Me), 2.41 (3H, s, Me); δ_C (75 MHz; $CDCl_3$) 198.9, 182.7, 172.3, 159.0, 142.1, 127.2, 123.8, 122.1, 107.2 (CH), 56.7 (Me), 32.7 (Me), 31.6 (Me), 10.9 (Me); *m/z* (ESI) 270 ($M+Na^+$, 100 %), 248 ($M+H^+$, 18).

Methyl 6-methoxy-1-(4-methoxybenzyl)-2-methyl-4,7-dioxo-4,7-dihydro-1*H*-indole-3-carboxylate (16)

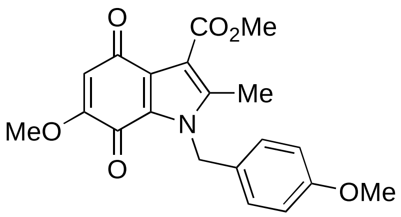

Prepared by the general procedure from quinone **6a** (0.217 g, 1.0 mmol), enamine **7e** (0.235 g, 1.0 mmol), copper(II) acetate monohydrate (0.299 g, 1.5 mmol) and potassium carbonate (0.414 g, 3.0 mmol) stirred at reflux in acetonitrile (10 mL) for 4.5 h. Column chromatography eluting with ethyl acetate and dichloromethane (1:12) gave the *title compound* as a yellow solid (0.306 g, 89%); mp 174-176 °C; (Found: M+Na$^+$, 392.1110. $C_{20}H_{19}NO_6Na$ requires 392.1105); ν_{max} ($CHCl_3$)/cm^{-1} 3011, 2954, 1714, 1651, 1611, 1514, 1247; δ_H (400 MHz; $CDCl_3$) 7.04 (2H, d, *J* 8.0, ArH), 6.85 (2H, d, *J* 8.0, ArH), 5.74 (1H, s, 5-H), 5.60 (2H, s, CH_2), 3.93 (3H, s, Me), 3.82 (3H, s, Me), 3.79 (3H, s, Me), 2.44 (3H, s, Me); δ_C (75 MHz; $CDCl_3$) 181.4, 172.1, 164.8, 159.3, 158.8, 142.6, 128.0 (CH), 127.4, 127.3, 125.0, 114.3 (CH), 113.4, 107.4 (CH), 56.6 (Me), 55.3 (Me), 52.1 (Me), 48.3 (CH_2), 11.2 (Me); *m/z* (ESI) 761 (2M+Na$^+$, 92%), 392 (M+Na$^+$, 100).

***tert*-Butyl 6-methoxy-1-(3-methoxyphenyl)-2-methyl-4,7-dioxo-4,7-dihydro-1*H*-indole-3-carboxylate (17)**

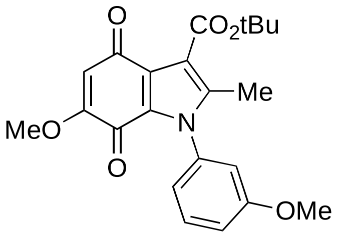

Prepared by the general procedure from quinone **6a** (0.217 g, 1.0 mmol), enamiine **7f** (0.263 g, 1.0 mmol), copper(II) acetate monohydrate (0.299 g, 1.5 mmol) and potassium carbonate (0.414 g, 3.0 mmol) stirred at reflux in acetonitrile (10 mL) for 4 h. Column chromatography eluting with ethyl acetate and dichloromethane (1:13) gave the *title compound* as an orange solid (0.272 g, 73%); mp 205-206 °C; (Found: M+Na$^+$, 420.1419. $C_{22}H_{23}NO_6Na$ requires 420.1418); ν_{max} ($CHCl_3$)/cm^{-1} 3071, 3057, 1672, 1651, 1609, 1497, 1112; δ_H (400 MHz; $CDCl_3$) 7.41 (1H, dd, *J* 8.4, 7.8, ArH), 7.07 (1H, d, *J* 8.4, ArH), 6.79 (1H, d, *J* 7.8, ArH), 6.73 (1H, s, ArH), 5.73 (1H, s, 5-H), 3.82 (3H, s, OMe), 3.76 (3H, s, OMe), 2.22 (3H, s, Me), 1.64 (9H, s, *t*Bu); δ_C (75 MHz; $CDCl_3$) 181.5, 170.7, 163.6, 160.4, 158.8, 142.2, 137.4, 130.2 (CH),

128.3, 124.6, 119.2 (CH), 115.3 (CH), 112.9 (CH), 107.3 (CH), 81.9, 56.4 (Me), 55.5 (Me), 28.2 (Me), 11.5 (Me); one C unobserved; *m/z* (ESI) 817 ($2M+Na^+$, 100%, 420 (67).

***tert*-Butyl 2-methyl-1-(2-morpholinoethyl)-4,9-dioxo-4,9-dihydro-1*H*-benzo[*f*]indole-3-carboxylate (18)**

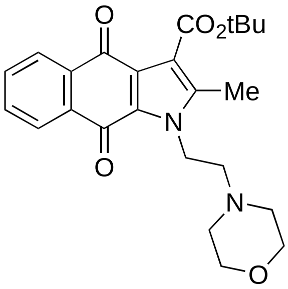

Prepared by the general procedure from quinone **6i** (0.237 g, 1.0 mmol), enamine **7g** (0.270 g, 1.0 mmol), copper(II) acetate monohydrate (0.299 g, 1.5 mmol) and potassium carbonate (0.414 g, 3.0 mmol) stirred at reflux in acetonitrile (10 mL) for 4 h. Column chromatography eluting with ethyl acetate and dichloromethane (1:1) gave the *title compound* as an orange oil (0.303 g, 71%); (Found: $M+H^+$, 425.2081. $C_{24}H_{29}N_2O_5$ requires 425.2071); ν_{max} ($CHCl_3$)/cm^{-1} 3011, 2978, 1714, 1852, 1283, 1118; δ_H (400 MHz; $CDCl_3$) 8.15-8.08 (2H, m, ArH), 7.68-7.66 (2H, m, ArH), 4.56 (2H, t, *J* 6.6, CH_2), 3.70 (4H, t, *J* 4.7, CH_2), 2.71 (2H, t, *J* 6.6, CH_2), 2.57 (4H, t, *J* 4.7, CH_2), 2.50 (3H, s, Me), 1.66 (9H, s, *t*Bu); δ_C (75 MHz; $CDCl_3$) 179.4, 176.0, 164.0, 141.1, 133.9, 133.3, 133.2 (CH), 132.9 (CH), 129.8, 126.7 (CH), 126.2 (CH), 125.8, 116.0, 81.9, 67.0 (CH_2), 58.4 (CH_2), 54.0 (CH_2), 53.6 (CH_2), 28.3 (Me), 10.8 (Me); *m/z* (ESI) 425 ($M+H^+$, 100%).

Dimethyl 6-methoxy-1-(4-methoxybenzyl)-4,7-dioxo-4,7-dihydro-1*H*-indole-2,3-dicarboxylate (19)

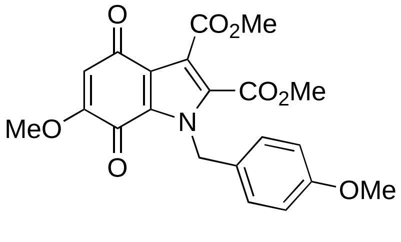

Prepared by the general procedure from quinone **6a** (0.217 g, 1.0 mmol), enaimne **7h** (0.279 g, 1.0 mmol), copper(II) acetate monohydrate (0.299 g, 1.5 mmol) and potassium carbonate (0.414 g, 3.0 mmol) stirred at reflux in acetonitrile (10 mL) for 4 h. Column chromatography eluting with ethyl acetate and dichloromethane (24:1) gave the *title compound* as a bright

yellow solid (0.216 g, 52%); mp 202-204 °C; (Found: M+Na$^+$, 436.1010. $C_{21}H_{19}NO_8Na$ requires 436.1003); ν_{max} ($CHCl_3$)/cm^{-1} 3011, 2955, 1729, 1653, 1604, 1242; δ_H (400 MHz; $CDCl_3$) 7.19 (2H, d, *J* 8.0, ArH), 6.82 (2H, d, *J* 8.0, ArH), 6.05 (2H, s, CH_2), 5.86 (1H, s, 5-H), 3.98 (3H, s, Me), 3.89 (3H, s, Me), 3.85 (3H, s, Me), 3.78 (3H, s, Me); δ_C (75 MHz; $CDCl_3$) 180.6, 172.6, 164.7, 160.1, 159.7, 159.3, 132.0, 129.0 (CH), 128.3, 126.5, 123.5, 121.8, 114.0 (CH), 107.8 (CH), 56.9 (Me), 55.3 (Me), 53.0 (Me), 52.8 (Me), 49.5 (CH_2); *m/z* (ESI) 436 (M+Na$^+$, 100%).

Methyl 1-(2-(*tert*-butyldimethylsiloxy)ethyl)-6-methoxy-2-methyl-4,7-dioxo-4,7-dihydro-1*H*-indole-3-carboxylate (20)

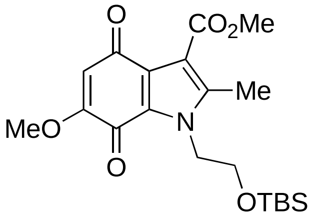

Prepared by the general procedure from quinone **6a** (0.217 g, 1.0 mmol), enamine **7i** (0.273 g, 1.0 mmol), copper(II) acetate monohydrate (0.299 g, 1.5 mmol) and potassium carbonate (0.414 g, 3.0 mmol) stirred at reflux in acetonitrile (10 mL) for 2.5 h. Column chromatography eluting with dichloromethane gave the *title compound* as an orange solid (0.316 g, 78%); mp 133-135 °C; (Found: M+Na$^+$, 430.1650. $C_{20}H_{29}NO_6SiNa$ requires 430.1656); ν_{max} ($CHCl_3$)/cm^{-1} 3011, 2955, 1717, 1651, 1610, 1240; δ_H (400 MHz; $CDCl_3$) 5.73 (1H, s, 5-H), 4.46 (2H, t, *J* 4.6, CH_2), 3.95-3.91 (5H, m, OMe + CH_2), 3.83 (3H, s, OMe), 2.51 (3H, s, Me), 0.82 (9H, s, *t*Bu), -0.07 (6H, s, $SiMe_2$); δ_C (75 MHz; $CDCl_3$) 181.5, 172.0, 164.8, 158.7, 143.9, 127.1, 125.1, 113.0, 107.4 (CH), 62.3 (CH_2), 56.6 (Me), 52.0 (Me), 48.0 (CH_2), 25.7 (Me), 18.1, 11.5 (Me); -5.7; *m/z* (ESI) 430 (M+Na$^+$, 100%).

Methyl 1-(2-(*tert*-butoxycarbonylamino)ethyl)-6-methoxy-2-methyl-4,7-dioxo-4,7-dihydro-1*H*-indole-3-carboxylate (21)

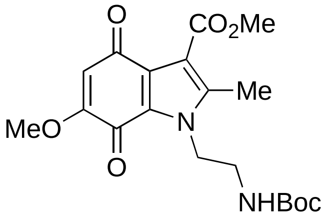

Prepared by the general procedure from quinone **6a** (0.217 g, 1.0 mmol), enamine **7j** (0.258 g, 1.0 mmol), copper(II) acetate monohydrate (0.299 g, 1.5 mmol) and potassium carbonate

(0.414 g, 3.0 mmol) stirred at reflux in acetonitrile (10 mL) for 4 h. Column chromatography eluting with dichloromethane then ethyl acetate and dichloromethane (1:14) gave the *title compound* as a yellow solid (0.354 g, 90%); mp 186-188 °C; (Found: M+Na$^+$, 415.1492. $C_{19}H_{24}N_2O_7Na$ requires 415.1476); ν_{max} ($CHCl_3$)/cm^{-1} 3460, 3011, 2981, 1710, 1652, 1610, 1503, 1244; δ_H (400 MHz; $CDCl_3$) 5.73 (1H, s, 5-H), 4.78 (1H, br s, NH), 4.43-4.40 (2H, m, CH_2), 3.92 (3H, s, OMe), 3.83 (3H, s, OMe), 3.47 (2H, q, *J* 6.0, CH_2), 2.49 (3H, s, Me), 1.42 (9H, s, *t*Bu); δ_C (75 MHz; $CDCl_3$) 181.3, 172.1, 164.7, 158.7, 155.9, 143.2, 127.3, 125.2, 113.2, 107.4 (CH), 79.9, 56.6 (Me), 52.0 (Me), 45.2 (CH_2), 40.4 (CH_2), 28.3 (Me), 10.8 (Me); *m/z* (ESI) 807 (2M+Na$^+$, 100%), 415 (M+Na$^+$, 93).

Ethyl 6-methoxy-2-(4-methoxybenzyloxy)methyl-1-methyl-4,7-dioxo-4,7-dihydro-1*H*-indole-3-carboxylate (22)

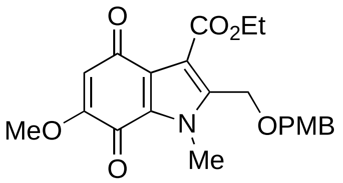

Prepared by the general procedure from quinone **6a** (1.085 g, 5.0 mmol), enamine **7k** (1.395 g, 5.0 mmol), copper(II) acetate monohydrate (1.995 g, 10 mmol) and potassium carbonate (2.070 g, 15 mmol) stirred at reflux in acetonitrile (50 mL) for 14 h. Column chromatography eluting with dichloromethane and ethyl acetate (19:1 then 9:1) gave the *title compound* as a bright yellow solid (1.539 g, 75%); mp 148-150 °C; (Found: M+Na$^+$, 436.1378. $C_{22}H_{23}NO_7Na$ requires 436.1367); ν_{max} ($CHCl_3$)/cm^{-1} 3053, 2939, 1670, 1652, 1610, 1249; δ_H (400 MHz; $CDCl_3$) 7.24 (2H, d, *J* 7.2, ArH), 6.89 (2H, d, *J* 7.2, ArH), 5.77 (1H, s, 5-H), 4.72 (2H, s, OCH_2), 4.49 (2H, s, OCH_2), 4.37 (2H, q, *J* 7.1, OCH_2), 4.01 (3H, s, Me), 3.83 (3H, s, Me), 3.82 (3H, s, Me), 1.39 (3H, t, *J* 7.1, $CH_2C\underline{H}_3$); δ_C (75 MHz; $CDCl_3$) 181.1, 172.8, 163.8, 159.5, 158.8, 141.6, 139.9, 129.6 (CH), 129.3, 123.9, 115.4, 113.9 (CH), 107.9 (CH), 72.2 (CH_2), 61.4 (CH_2), 60.2 (CH_2), 56.6 (Me), 55.3 (Me), 33.6 (Me), 14.4 (Me); *m/z* (ESI) 849 (2M+Na$^+$, 94%), 436 (M+Na$^+$, 100).

Methyl 6-ethyl-1,2-dimethyl-4,7-dioxo-4,7-dihydro-1*H*-indole-3-carboxylate (23)

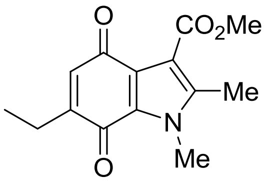

Prepared by the general procedure from quinone **6f** (0.645 g, 3.0 mmol), enamine **7a** (0.387 g, 3.0 mol), copper(II) acetate monohydrate (1.197 g, 6.0 mmol) and potassium carbonate (1.242 g, 9.0 mmol) stirred at reflux in acetonitrile (30 mL) for 3.5 h. Column chromatography eluting with ethyl acetate and dichloromethane (1:49 then 1:29) gave the *title compound* as an orange solid (0.208 g, 32 %), mp 71-73 °C; (Found: M+Na$^+$, 284.0874. $C_{14}H_{15}NO_4Na$ requires: 284.0899); ν_{max} (CHCl$_3$)/cm^{-1} 3011, 2654, 1710, 1650; δ_H (400 MHz; CDCl$_3$) 6.38 (1H, d, *J* 1.6, CH), 3.96 (3H, s, Me), 3.93 (3H, s, Me), 2.49 (2H, qd, *J* 7.4, 1.6, CH$_2$), 2.48 (3H, s, Me), 1.17 (3H, t, *J* 7.4, Me); δ_C (75 MHz; CDCl$_3$) 181.2, 178.6, 164.8, 149.4, 141.9, 132.7 (CH), 129.5, 123.8, 112.1, 51.9 (Me), 32.6 (Me), 21.7 (CH$_2$), 12.3 (Me), 10.9 (Me); *m/z* (ESI) 284 (M+Na$^+$, 100 %).

Methyl 6-isopropoxy-1,2-dimethyl-4,7-dioxo-4,7-dihydro-1*H*-indole-3-carboxylate (24)

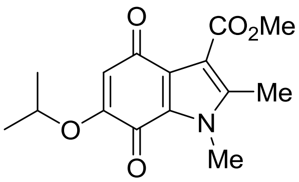

Prepared by the general procedure from quinone **6g** (0.735 g, 3.0 mmol), enamine **7a** (0.387 g, 3.0 mol), copper(II) acetate monohydrate (1.197 g, 6.0 mmol) and potassium carbonate (1.242 g, 9.0 mmol) stirred at reflux in acetonitrile (30 mL) for 4 h. Column chromatography eluting with ethyl acetate and dichloromethane (1:29) gave the *title compound* as a yellow solid (0.690 g, 79 %); mp 177-179 °C; (Found: M+Na$^+$, 314.0994. $C_{15}H_{17}NO_5Na$ requires: 314.0999); δ_H (400 MHz; CDCl$_3$) 5.69 (1H, s, CH), 4.46 (1H, sep, *J* 6.0, CH), 3.94 (3H, s, Me), 3.93 (3H, s, Me), 2.46 (3H, s, Me), 1.42 (6H, d, *J* 6.0, Me); δ_C (75 MHz; CDCl$_3$) 181.8, 172.9, 164.8, 156.7, 142.4, 128.0, 124.3, 112.7, 108.1 (CH), 72.3 (CH), 52.0 (Me), 32.7 (Me), 21.1 (Me), 10.9 (Me); ν_{max} (CHCl$_3$)/cm^{-1} 3011, 1713, 1667, 1648, 1603, 1470; *m/z* (ESI) 314 (M+Na$^+$, 100 %), 605 (2M+Na$^+$, 95).

Methyl 6,7-dimethyl-5,9-dioxo-3,5,6,9-tetrahydro-2*H*-[1,4]dioxino[2,3-*f*]indole-8-carboxylate (25)

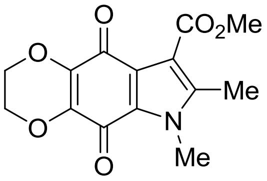

Prepared by the general procedure from quinone **6h** (0.245 g, 1.0 mmol), enamine **7a** (0.129 g, 1.0 mol), copper(II) acetate monohydrate (0.399 g, 2.0 mmol) and potassium carbonate (0.414 g, 3.0 mmol) stirred at reflux in acetonitrile (10 mL) for 4 h. Column chromatography eluting with ethyl acetate and dichloromethane (1:7) gave the *title compound* as a pink solid (0.075 g, 26 %); mp 236-238 ^{o}C; (Found: M+Na^{+}, 314.0635. $C_{14}H_{13}NO_6Na$ requires 314.0635); δ_H (400 MHz; $CDCl_3$) 4.33 (4H, s, 2 x CH_2), 3.94 (3H, s, Me), 3.92 (3H, s, Me), 2.46 (3H, s, Me); δ_C (75 MHz; $CDCl_3$) 175.1, 172.7, 164.7, 141.8, 139.0, 137.8, 127.0, 121.2, 113.0, 64.7 (CH_2), 64.6 (CH_2), 51.9 (Me), 32.8 (Me), 10.7 (Me); ν_{max} ($CHCl_3$)/cm^{-1} 3002, 1708, 1655, 1626, 1602; *m/z* (ESI) 314 (M+Na^{+}, 100 %).

2-Methoxy-9-methyl-7,8-dihydro-1*H*-carbazole-1,4,5(6*H*,9*H*)-trione (26)

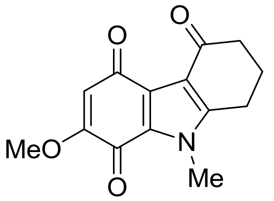

Prepared by the general procedure from quinone **6a** (0.313 g, 2.5 mmol), enamine **7l** (0.543 g, 2.5 mmol), copper(II) acetate (0.998 g, 5.0 mmol) and potassium carbonate (1.035 g, 7.5 mmol) in acetonitrile (25 mL) at reflux for 5 h. Column chromatography eluting with methanol and dichloromethane (1:19) gave the *title compound* as a yellow-orange solid (0.385 g, 59 %), mp 212-214 °C (dec.); (Found: M+H^{+}, 260.0902. $C_{14}H_{14}NO_4$ requires: 260.0923); δ_H (400 MHz; $CDCl_3$) 5.85 (1H, s, CH), 3.98 (3H, s, Me), 3.84 (3H, s, Me), 5.86 (2H, t, *J* 6.3, CH_2), 2.62 (2H, t, *J* 6.5, CH_2), 2.22 (2H, tt, *J* 6.5, 6.3, CH_2); δ_C (75 MHz; $CDCl_3$) 191.3, 180.9, 173.4, 158.1, 150.9, 129.8, 123.9, 118.5, 108.2 (CH), 56.6 (Me), 39.1 (CH_2), 33.2 (Me), 22.3 (CH_2), 22.0 (CH_2); ν_{max} ($CHCl_3$)/cm^{-1} 3690, 3004, 1602; *m/z* (ESI) 282 (M+Na^{+}, 100 %), 260 (M+H^{+}, 25).

(S)-*tert*-Butyl 6-methoxy-1-(1-methoxy-4-methyl-1-oxopentan-2-yl)-2-methyl-4,7-dioxo-4,7-dihydro-1*H*-indole-3-carboxylate (27)

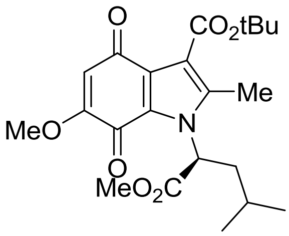

Prepared by the general procedure from quinone **6a** (0.217 g, 1.0 mmol), enamine **7n** (0.285 g, 1.0 mmol), copper(II) acetate monohydrate (0.399 g, 2.0 mmol) and potassium carbonate (0.414 g, 3.0 mmol) stirred at reflux in acetonitrile (10 mL) for 6 h. Column chromatography eluting with ethyl acetate and dichloromethane (1:12) gave the *title compound* as a yellow solid (0.262 g, 63 %); mp 93-95 °C; (Found: M+Na$^+$, 442.1848. $C_{22}H_{29}NO_7Na$ requires 442.1836); ν_{max} ($CHCl_3$)/cm^{-1} 2959, 1745, 1699, 1652, 1610, 1247; δ_H (400 MHz; $CDCl_3$) 5.73 (1H, s, CH), 4.84 (1H, br s, CH), 3.83 (3H, s, Me), 3.76 (3H, s, Me), 2.50-2.38 (5H, m, Me + CH_2), 1.93-1.68 (1H, m, CH), 1.63 (9H, s, *t*Bu), 0.98 (3H, d, *J* 6.5, Me), 0.85 (3H, d, *J* 6.6, Me); δ_C (75 MHz; $CDCl_3$) 188.6, 181.3, 163.6, 158.9, 141.8, 126.8, 107.1 (CH), 82.0, 56.6 (Me), 52.9 (Me), 40.3 (CH_2), 28.1 (Me), 25.3 (CH), 22.9 (Me), 22.1 (Me), 11.4 (Me); four C unobserved; *m/z* (ESI) 442 (M+Na$^+$, 100 %).

(±)-Menthyl 1-(2-(1*H*-indol-3-yl)ethyl)-6-methoxy-2-methyl-4,7-dioxo-4,7-dihydro-1*H*-indole-3-carboxylate (28)

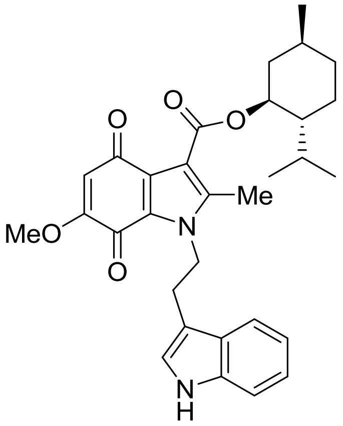

Prepared by the general procedure from quinone **6a** (0.217 g, 1.0 mmol), enamine **7o** (0.382 g, 1.0 mmol), copper(II) acetate monohydrate (0.399 g, 2.0 mmol) and potassium carbonate (0.414 g, 3.0 mmol) stirred at reflux in acetonitrile (10 mL) for 14 h. Column chromatography eluting with ethyl acetate and dichloromethane (1:19) gave the *title compound* as a yellow solid (0.267 g, 53 %); mp 275-277 °C; (Found: M+Na$^+$, 539.2530. $C_{31}H_{36}N_2O_5Na$ requires 539.2516); ν_{max} ($CHCl_3$)/cm^{-1} 3606, 3479, 3009, 2960, 1711, 1651, 1604, 1242; δ_H (400 MHz; $CDCl_3$) 8.10 (1H, br s, NH), 7.73 (1H, d, *J* 7.8, ArH), 7.41 (1H, d, *J* 8.0, ArH), 7.25 (1H, dd, *J* 7.8, 6.9, ArH), 7.19 (1H, dd, *J* 8.0, 6.9, ArH), 7.03 (1H, d, *J* 3.3, ArH), 5.78 (1H, s, CH), 5.00 (1H, td, *J* 10.9, 4.4, CH), 4.58 (2H, t, *J* 7.7, CH_2), 3.87 (3H, s, Me), 3.20 (2H, t, *J* 7.7, CH_2), 2.24-2.19 (1H, m, CH), 2.22 (3H, s, Me), 2.08-2.00 (1H, m, CH), 1.77-1.70 (2H, m, CH_2),1.58-1.46 (2H, m, CH_2),1.30-1.08 (3H, m, 3 CH),0.98 (3H, d, *J* 6.7, Me), 0.94 (3H, d, *J*

7.1, Me), 0.85 (3H, d, *J* 6.9, Me); δ_C (75 MHz; $CDCl_3$) 181.4, 171.9, 164.2, 158.9, 141.4, 136.2, 127.1, 127.0, 124.8, 122.6 (CH), 122.4 (CH), 119.9 (CH), 118.5 (CH), 114.3, 111.6, 111.3 (CH), 107.3 (CH), 75.1 (CH), 56.6 (Me), 47.0 (CH), 46.9 (CH_2), 40.6 (CH_2), 34.3 (CH_2), 31.6 (CH), 26.3 (CH_2), 26.0 (CH), 23.2 (CH_2), 22.1 (Me), 20.9 (Me), 16.1 (Me), 10.5 (Me); *m/z* (ESI) 539 ($M+Na^+$, 100 %), 517 ($M+H^+$, 30).

6-Methoxy-1-(4-methoxybenzyl)-2-methyl-3-(morpholine-4-carbonyl)-1*H*-indole-4,7-dione (29)

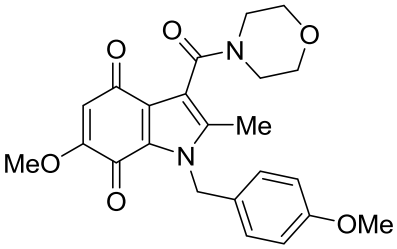

Prepared by the general procedure from quinone **6a** (0.217 g, 1.0 mmol), enamine **7p** (0.290 g, 1.0 mmol), copper(II) acetate monohydrate (0.399 g, 2.0 mmol) and potassium carbonate (0.414 g, 3.0 mmol) stirred at reflux in acetonitrile (10 mL) for 6 h. Column chromatography eluting with ethyl acetate gave the *title compound* as a yellow solid (0.262 g, 63 %); mp 161-163 °C; (Found: $M+Na^+$, 447.1537. $C_{23}H_{24}N_2O_6Na$ requires 447.1537); ν_{max} ($CHCl_3$)/cm^{-1} 3011, 1646, 1605, 1514, 1240; δ_H (400 MHz; $CDCl_3$) 7.11 (2H, d, *J* 8.7, ArH), 6.86 (2H, d, *J* 8.7, ArH), 5.70 (1H, s, CH), 5.58 (1H, d, *J* 15.2, CH), 5.53 (1H, d, *J* 15.3, CH), 3.95-3.80 (3H, m, 3 CH), 3.83 (3H, s, Me), 3.80 (3H, s, Me), 3.75-3.69 (2H, m, 2 CH), 3.54-3.48 (1H, m, CH), 3.41-3.36 (1H, m, CH), 3.30-3.28 (1H, m, CH), 2.29 (3H, s, Me); δ_C (75 MHz; $CDCl_3$) 182.3, 171.5, 164.2, 160.0, 159.4, 138.6, 128.4 (CH), 127.4, 126.4, 123.6, 116.6, 114.3 (CH), 106.4 (CH), 66.7 (CH_2), 56.7 (Me), 55.3 (Me), 48.5 (CH_2), 47.2 (CH_2), 42.3 (CH_2), 10.8 (Me); *m/z* (ESI) 447 ($M+Na^+$, 100 %), 425 (63).

***tert*-Butyl 1-(4-methoxybenzyl)-2-methyl-3-(morpholine-4-carbonyl)-4,7-dioxo-4,7-dihydro-1*H*-indol-6-ylcarbamate (30)**

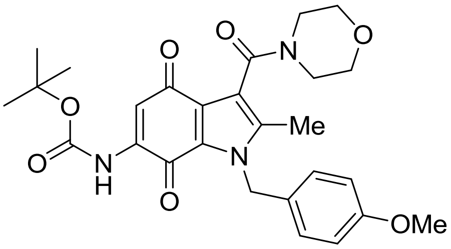

Prepared by the general procedure from quinone **6d** (0.302 g, 1.0 mmol), enamine **7p** (0.285 g, 1.0 mmol), copper(II) acetate monohydrate (0.399 g, 2.0 mmol) and potassium carbonate (0.414 g, 3.0 mmol) stirred at reflux in acetonitrile (10 mL) for 6 h. Column chromatography eluting with ethyl acetate and dichloromethane (1:1) gave the *title compound* as a yellow solid (0.208 g, 41 %); mp 208-210 °C; (Found: M+Na$^+$, 532.2068. $C_{27}H_{31}N_3O_7Na$ requires 532.2054); ν_{max} ($CHCl_3$)/cm^{-1} 3370, 3011, 1734, 1642, 1613, 1500; δ_H (400 MHz; $CDCl_3$) 7.64 (1H, br s, NH), 7.06 (2H, d, *J* 8.6, ArH), 7.02 (1H, s, CH), 6.88 (2H, d, *J* 8.6, ArH), 5.57 (1H, d, *J* 15.4, CH), 5.52 (1H, d, *J* 15.4, CH), 3.94-3.72 (5H, m, 3 CH), 3.81 (3H, s, Me), 3.51-3.47 (1H, m, CH), 3.43-3.38 (1H, m, CH), 3.29-3.22 (1H, m, CH), 2.29 (3H, s, Me), 1.53 (9H, s, *t*Bu); δ_C (75 MHz; $CDCl_3$) 182.9, 171.5, 164.0, 159.4, 151.3, 140.2, 139.4, 127.9 (CH), 127.3, 125.3, 124.0, 117.1, 114.0 (CH), 111.4 (CH), 82.3, 66.7 (CH_2), 55.3 (Me), 48.5 (CH_2), 47.1 (CH_2), 42.3 (CH_2), 28.1 (Me), 10.7 (Me); *m/z* (ESI) 532 (M+Na$^+$, 100 %), 510 (M+H$^+$, 74).

Methyl 1-(2,2-diethoxyethyl)-6-methoxy-4,7-dioxo-2-phenyl-4,7-dihydro-1*H*-indole-3-carboxylate (31)

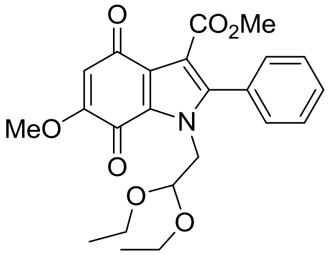

Prepared by the general procedure from quinone **6a** (0.217 g, 1.0 mmol), enamine **7q** (0.293 g, 1.0 mmol), copper(II) acetate monohydrate (0.399 g, 2.0 mmol) and potassium carbonate (0.414 g, 3.0 mmol) stirred at reflux in acetonitrile (10 mL) for 16 h. Column chromatography eluting with ethyl acetate and dichloromethane (1:19) gave the *title compound* as an orange solid (0.398 g, 93 %); mp 117-119 °C; (Found: M+Na$^+$, 450.1537. $C_{23}H_{25}NO_7Na$ requires 450.1523); ν_{max} ($CHCl_3$)/cm^{-1} 3009, 2980, 1726, 1652, 1606, 1240; δ_H (400 MHz; $CDCl_3$) 7.47-7-44 (5H, m, 5 ArH), 5.83 (1H, s, CH), 4.70 (1H, t, *J* 5.4, CH), 4.34 (2H, d, *J* 5.4, CH_2), 3.88 (3H, s, Me), 3.71 (3H, s, Me), 3.65 (2H, dq, *J* 9.4, 7.1, 2 CH), 3.46 (2H, dt, *J* 9.4, 7.1, 2H, 2 CH), 1.13 (6H, t, *J* 7.1, Me); δ_C (75 MHz; $CDCl_3$) 181.5, 172.4, 164.4, 159.2, 144.9, 130.7 (CH), 129.6 (CH), 128.4, 128.3 (CH), 127.2, 125.0, 115.2, 107.5 (CH), 101.3 (CH), 64.1 (CH_2), 56.8 (Me), 52.2 (Me), 48.9 (CH_2), 15.2 (Me); *m/z* (ESI) 450 (M+Na$^+$, 100 %).

tert-Butyl 1-(2-(1*H*-indol-3-yl)ethyl)-6-isopropoxy-2-methyl-4,7-dioxo-4,7-dihydro-1*H*-indole-3-carboxylate (32)

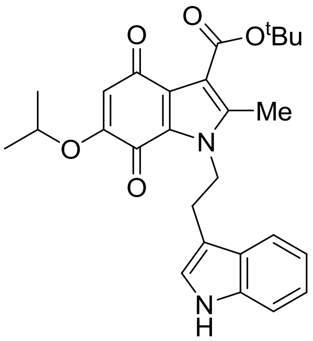

Prepared by the general procedure from quinone **6g** (0.245 g, 1.0 mmol), enamine **7m** (0.300 g, 1.0 mmol), copper(II) acetate monohydrate (0.399 g, 2.0 mmol) and potassium carbonate (0.414 g, 3.0 mmol) stirred at reflux in acetonitrile (10 mL) for 17 h. Column chromatography eluting with ethyl acetate and dichloromethane (1:19) gave the *title compound* as an orange solid (0.218 g, 47 %); mp 168-170 °C; (Found: M+Na$^+$, 485.2066. $C_{27}H_{30}N_2O_5Na$ requires 485.2047); ν_{max} ($CHCl_3$)/cm^{-1} 3479, 3008, 1647, 1602, 1242; δ_H (400 MHz; $CDCl_3$) 8.09 (1H, br s, NH), 7.71 (1H, d, *J* 7.8, ArH), 7.40 (1H, d, *J* 8.0, ArH), 7.25 (1H, dd, *J* 7.5, 7.8, ArH), 7.19 (1H, dd, *J* 7.5, 8.0, ArH), 7.01 (1H, d, *J* 2.3, ArH), 5.72 (1H, s, CH), 4.56 (2H, t, *J* 7.5, CH_2), 4.50 (1H, sep, *J* 6.1, CH), 3.20 (2H, t, *J* 7.5, CH_2), 2.20 (3H, s, Me), 1.63 (9H, s, *t*Bu), 1.46 (6H, d, *J* 6.1, Me); δ_C (75 MHz; $CDCl_3$) 181.9, 172.4, 163.8, 156.9, 141.2, 136.2, 127.1, 127.0, 124.5, 122.6 (CH), 122.3 (CH), 119.8 (CH), 118.5 (CH), 115.2, 111.7, 111.3 (CH), 108.0 (CH), 81.7, 72.3 (CH), 46.7 (CH_2), 28.1 (Me), 26.3 (CH_2), 21.2 (Me), 10.4 (Me); *m/z* (ESI) 485 (M+Na$^+$, 100 %), 407 (M-*t*Bu+H$^+$, 36).

Methyl 1-(2-*tert*-butoxy-2-oxoethyl)-6-methoxy-2-methyl-4,7-dioxo-4,7-dihydro-1*H*-indole-3-carboxylate (33)

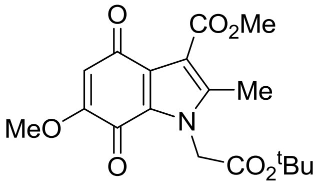

Prepared by the general procedure from quinone **6a** (1.085 g, 5.0 mmol), enamine **7t** (1.145 g, 5.0 mmol), copper(II) acetate monohydrate (1.995 g, 10.0 mmol) and potassium carbonate (2.070 g, 15.0 mmol) stirred at reflux in acetonitrile (50 mL) for 16 h. Column chromatography eluting with ethyl acetate and dichloromethane (1:14) gave the *title compound* as an orange solid (1.421 g, 78 %); mp 176-178 °C; (Found: M+H$^+$, 364.1380.

$C_{18}H_{22}NO_7$ requires 364.1391); ν_{max} ($CHCl_3$)/cm^{-1} 3011, 2984, 1745, 1654, 1609, 1240; δ_H (400 MHz; $CDCl_3$) 5.76 (1H, s, CH), 5.07 (2H, s, CH_2), 3.94 (3H, s, OMe), 3.83 (3H, s, OMe), 2.44 (3H, s, Me), 1.51 (9H, s, *t*Bu); δ_C (75 MHz; $CDCl_3$) 181.2, 172.4, 165.9, 164.5, 158.5, 142.8, 127.9, 124.9, 113.1, 107.8 (CH), 83.6, 56.6 (Me), 52.0 (Me), 47.4 (CH_2), 28.0 (Me), 10.7 (Me); *m/z* (ESI) 386 ($M+Na^+$, 100 %), 365 ($M+H^+$, 36).

Methyl 1-cyclohexyl-2-ethyl-6-isopropoxy-4,7-dioxo-4,7-dihydro-1*H*-indole-3-carboxylate (34)

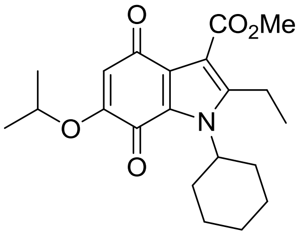

Prepared by the general procedure from quinone **6g** (0.245 g, 1.0 mmol), enamine **7s** (0.211 g, 1.0 mmol), copper(II) acetate monohydrate (0.399 g, 2.0 mmol) and potassium carbonate (0.414 g, 3.0 mmol) stirred at reflux in acetonitrile (10 mL) for 16 h. Column chromatography eluting with ethyl acetate and dichloromethane (0:1 then 1:99) gave the *title compound* as an orange solid (0.296 g, 79 %); mp 150-152 °C; (Found: $M+Na^+$, 396.1793. $C_{21}H_{27}NO_5Na$ requires 396.1781); ν_{max} ($CHCl_3$)/cm^{-1} 3011, 2985, 2938, 1720, 1668, 1346, 1604, 1289; δ_H (400 MHz; $CDCl_3$) 5.68 (1H, s, CH), 47 (1H, sep, *J* 6.1, OCH), 4.17-4.07 (1H, m, CH), 3.93 (3H, s, OMe), 2.85 (2H, br s, CH_2), 2.58 (2 H, br s, CH_2), 1.92 (2H, br d, *J* 13.0, CH_2), 1.70-1.35 (6H, m, 3 × CH_2), 1.42 (6H, d, *J* 6.0, Me), 1.24 (3H, t, *J* 6.1, Me); δ_C (75 MHz; $CDCl_3$) 182.3, 170.7, 165.6, 157.6, 146.0, 127.5, 126.6, 113.2, 106.7 (CH), 72.3 (CH), 58.2 (CH), 52.3 (Me), 29.2 (CH_2), 26.1 (CH_2), 24.3 (CH_2), 21.1 (Me), 19.3 (CH_2), 14.4 (Me); *m/z* (ESI) 396 ($M+Na^+$, 100 %).

2-Methoxy-7,7-dimethyl-9-(4-nitrophenyl)-7,8-dihydro-1*H*-carbazole-1,4,5(6*H*,9*H*)-trione (35)

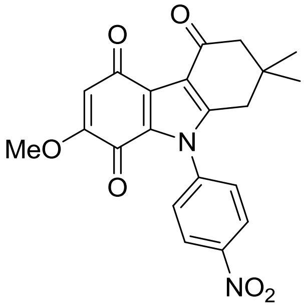

Prepared by the general procedure from quinone **6a** (0.217 g, 1.0 mmol), enamine **7r** (0.260 g, 1.0 mmol), copper(II) acetate monohydrate (0.399 g, 2.0 mmol) and potassium carbonate (0.414 g, 3.0 mmol) stirred at reflux in acetonitrile (20 mL) for 5 h. Column chromatography eluting with ethyl acetate and dichloromethane (1:4) gave the *title compound* as an orange solid (0.291 g, 74 %); mp 271-273 °C; (Found: M+Na$^+$, 417.1065. $C_{21}H_{18}N_2O_6Na$ requires 417.1057); ν_{max} ($CHCl_3$)/cm^{-1} 3011, 1646, 1311, 1499, 1351; δ_H (400 MHz; DMSO-d_6) 8.43 (2H, d, *J* 8.9, ArH), 7.83 (2H, d, *J* 8.9, ArH), 5.92 (1H, s, CH), 3.75 (3H, s, OMe), 2.50 (2H, s, CH_2), 2.41 (2H, s, CH_2), 1.00 (6H, s, Me); δ_C (75 MHz; DMSO-d_6) 190.9, 187.6, 180.5, 158.1, 150.2, 148.3, 141.3, 130.6, 128.4 (CH), 125.0 (CH), 124.5, 118.4, 108.3 (CH), 56.7 (Me), 53.2 (CH_2), 36.6 (CH_2), 34.9, 28.3 (Me); one C unobserved; *m/z* (ESI) 417 (M+Na$^+$, 100 %).

Cholesteryl 1-(2-(dimethylamino)ethyl)-6-methoxy-2-methyl-4,7-dioxo-4,7-dihydro-1*H*-indole-3-carboxylate (36)

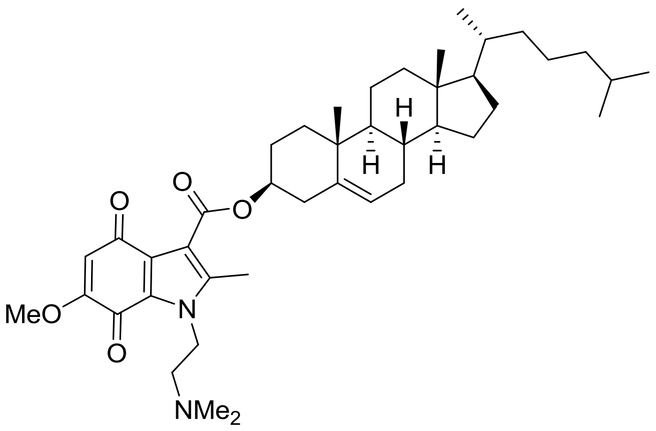

Prepared by the general procedure from quinone **6a** (0.109 g, 0.5 mmol), enamine **7v** (0.270 g, 0.5 mmol), copper(II) acetate monohydrate (0.200 g, 1.0 mmol) and potassium carbonate (0.207 g, 1.5 mmol) stirred at reflux in acetonitrile (20 mL) for 5 h. Column chromatography eluting with ethyl acetate and methanol (9:1) gave the *title compound* as an orange oil (0.190 g, 56 %); (Found: M+H$^+$, 675.4748. $C_{42}H_{63}N_2O_5$ requires 675.4731); ν_{max} ($CHCl_3$)/cm^{-1} 3011, 2952, 1669, 1352; δ_H (400 MHz; $CDCl_3$) 5.72 (1H, s, CH), 5.44 (1H, d, *J* 4.9, CH), 4.93-4.81 (1H, m, CH), 4.43 (2H, t, *J* 7.4, CH_2), 3.82 (3H, s, Me), 2.59 (2H, t, *J* 7.4, CH_2), 2.54-2.45 (5H, m), 2.35 (6H, s, NMe_2), 2.09-1.70 (7H, m), 1.62-1.48 (7H, m), 1.40-1.32 (4H, m), 1.29-0.99 (11H, m), 1.06 (3H, s, Me), 0.93 (3H, d, *J* 6.5, Me), 0.89 (3H, d, *J* 6.8, Me), 0.88 (3H, d, *J* 6.7, Me); δ_C (75 MHz; $CDCl_3$) 181.3, 171.7, 163.7, 158.7, 142.0, 139.7, 127.1, 124.8, 122.7 (CH), 114.1, 107.3 (CH), 74.9 (CH), 58.6 (CH_2), 56.7 (CH), 56.5 (Me), 56.1 (CH), 50.0 (CH), 45.7 (Me), 43.8 (CH_2), 42.3, 39.7 (CH_2), 39.5 (CH_2), 38.0 (CH_2), 37.1 (CH_2), 36.7, 36.2

(CH_2), 35.8 (CH), 31.9 (CH_2), 31.8 (CH), 28.2 (CH_2), 28.0 (CH), 27.7 (CH_2), 24.3 (CH_2), 23.8 (CH_2), 22.8 (Me), 22.6 (Me), 21.1 (CH_2), 19.4 (Me), 18.7 (Me), 11.9 (Me), 10.8 (Me); *m/z* (ESI) 675 ($M+H^+$, 100 %).

Dimethyl 1,2,6,7-tetramethyl-4,8-dioxo-1,4,7,8-tetrahydropyrrolo[3,2-*f*]indole-3,5-dicarboxylate (37)

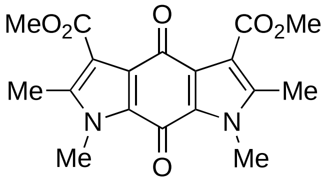

Prepared by the general procedure from quinone **6c** (0.266 g, 1.0 mmol), enamine **7a** (0.129 g, 2.0 mmol), copper(II) acetate monohydrate (0.299 g, 1.5 mmol) and potassium carbonate (0.414 g, 3.0 mmol) stirred at reflux in acetonitrile (10 mL) for 6 h. Column chromatography eluting with ethyl acetate and dichloromethane (1:10) gave the *title compound* as a red-orange solid (0.125 g, 35%); mp 250-252 °C; (Found: $M+Na^+$, 381.1058. $C_{18}H_{18}N_2O_6Na$ requires 381.1057); ν_{max} ($CHCl_3$)/cm^{-1} 3006, 2953, 1708, 1666, 1639, 1465, 1273; δ_H (400 MHz; $CDCl_3$) 3.95 (6H, s, Me), 3.93 (6H, s, Me), 2.40 (6H, s, Me); δ_C (75 MHz; $CDCl_3$) 176.0, 170.1, 165.4, 140.5, 129.3, 125.4, 113.4, 52.1 (Me), 32.3 (Me), 10.8 (Me); *m/z* (ESI) 739 ($2M+Na^+$, 100%), 381 ($M+Na^+$, 57).

Methyl 6-bromo-1-(2-(*tert*-butyldiphenylsilyloxy)ethyl)-2-methyl-4,7-dioxo-4,7-dihydro-1*H*-indole-3-carboxylate (38)

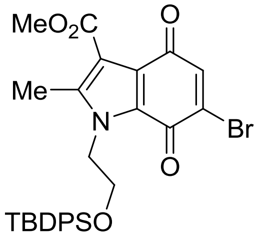

Prepared by the general procedure from quinone **6c** (0.798 g, 3.0 mmol), enamine **7w** (0.597 g, 1.5 mmol), copper(II) acetate monohydrate (0.599 g, 3.0 mmol) and potassium carbonate (0.621 g, 4.5 mmol) stirred at reflux in acetonitrile (15 mL) for 3.5 h. Column chromatography eluting with dichloromethane gave the *title compound* as an orange oil (0.394 g, 45 %); (Found: $M+H^+$, 580.1169. $C_{29}H_{31}N^{79}BrO_5Si$ requires: 580.1155); ν_{max} ($CHCl_3$)/cm^{-1} 3008, 1707, 1458, 1115; δ_H (400 MHz; $CDCl_3$) 7.45 (4H, dd, *J* 8.0, 1.5, ArH), 7.89 (2H, dt, *J* 6.4, 1.5, ArH), 7.32 (4H, dd, *J* 8.0, 6.4, ArH), 7.06 (1H, s, CH), 4.49 (2H, t, *J*

5.0, CH_2), 3.97 (2H, t, *J* 5.0, CH_2), 3.95 (3H, s, Me), 2.53 (3H, s, Me), 0.99 (9H, s, *t*Bu); δ_C (75 MHz; $CDCl_3$) 178.9, 169.8, 164.3, 143.9, 138.3 (CH), 136.7, 135.5 (CH), 132.4, 130.0 (CH), 127.8 (CH), 127.0, 124.6, 113.1, 62.4 (CH_2), 52.0 (Me), 47.6 (CH_2), 26.8 (Me), 18.9, 11.5 (Me); *m/z* (ESI) 602/604 ($M+Na^+$, 79/90 %), 580/582 ($M+H^+$, 41/49), 186 (100).

3-*tert*-Butyl 5-methyl 7-(2-(tert-butyldiphenylsilyloxy)ethyl)-1,2,6-trimethyl-4,8-dioxo-1,4,7,8-tetrahydropyrrolo[3,2-*f*]indole-3,5-dicarboxylate (39)

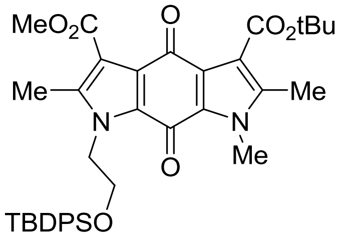

Prepared by the general procedure from quinone **38** (0.290 g, 0.5 mmol), enamine **7d** (0.171 g, 1.0 mmol), copper(II) acetate monohydrate (0.200 g, 1.0 mmol) and potassium carbonate (0.207 g, 1.5 mmol) stirred at reflux in acetonitrile (5 mL) for 14 h. Column chromatography eluting with ethyl acetate and dichloromethane (1:19) gave the *title compound* as an orange solid (0.298 g, 89 %); mp 75-77 °C; (Found: $M+Na^+$, 691.2805. $C_{38}H_{44}N_2O_7SiNa$ requires: 691.2810); ν_{max} ($CHCl_3$)/cm^{-1} 3011, 2886, 1709, 1639, 1462, 1113; δ_H (400 MHz; $CDCl_3$) 7.49 (4H, dd, *J* 7.8, 1.4, ArH), 7.35-7.27 (6H, m, ArH), 4.51 (2H, t, *J* 5.1, CH_2), 3.97 (2H, t, *J* 5.1, CH_2), 3.62 (3H, s, Me), 3.81 (3H, s, Me), 2.47 (3H, s, Me), 2.41 (3H, s, Me), 1.66 (9H, s, *t*Bu), 1.00 (9H, s, *t*Bu); δ_C (75 MHz; $CDCl_3$) 176.0, 170.2, 165.5, 164.1, 141.5, 139.8, 135.5 (CH), 132.7, 129.7 (CH), 128.8, 128.7, 127.7 (CH), 126.5, 125.9, 115.2, 113.0, 81.3, 62.9 (CH_2), 51.8 (Me), 47.2 (CH_2), 32.3 (Me), 28.1 (Me), 26.8 (Me), 18.9, 11.2 (Me), 10.6 (Me); *m/z* (ESI) 691 ($M+Na^+$, 100 %).

Dimethyl 1,2,5,6-tetramethyl-4,8-dioxo-1,4,5,8-tetrahydropyrrolo[2,3-*f*]indole-3,7-dicarboxylate (40)

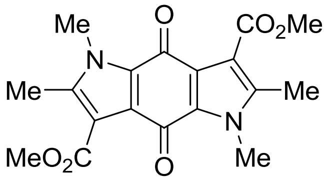

Prepared by the general procedure from quinone **6e** (0.133 g, 0.5 mmol), enamine **7a** (0.194 g, 1.0 mmol), copper(II) acetate monohydrate (0.399 g, 2.0 mmol) and potassium carbonate (0.414 g, 3.0 mmol) stirred at reflux in acetonitrile (10 mL) for 15 h. Column chromatography

eluting with ethyl acetate and dichloromethane (1:4) gave the *title compound* as a yellow-orange solid (0.035 g, 20 %); mp 290-293 °C; (Found: M+H$^+$, 359.1225. $C_{18}H_{18}N_2O_6$ requires 359.1240); ν_{max} ($CHCl_3$)/cm^{-1} 3011, 1718, 1651, 1602, 1458, 1249; δ_H (400 MHz; $CDCl_3$) 3.96 (6H, s, Me), 3.95 (6H, s, Me), 2.41 (6H, s, Me); δ_C (75 MHz; $CDCl_3$) 173.8, 165.2, 140.2, 130.5, 123.7, 113.8, 52.0 (Me), 32.7 (Me), 10.9 (Me); *m/z* (ESI) 381 (M+Na$^+$, 100 %), 359 (M+H$^+$, 56).

***tert*-Butyl 5-bromo-1,2-dimethyl-4,7-dioxo-4,7-dihydro-1*H*-indole-3-carboxylate (41)**

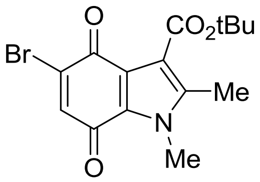

Prepared by the general procedure from quinone **6e** (0.266 g, 1.0 mmol), enamine **7d** (0.171 g, 1.0 mmol), copper(II) acetate monohydrate (0.399 g, 2.0 mmol) and potassium carbonate (0.414 g, 3.0 mmol) stirred at reflux in acetonitrile (10 mL) for 3.5 h. Column chromatography eluting with light petroleum and dichloromethane (1:1 then 0:1) gave the *title compound* as an orange solid (0.110 g, 31 %); mp 146-148 °C; (Found: M+Na$^+$, 376.0151. $C_{15}H_{16}NO_4{}^{79}BrNa$ requires 376.0155); ν_{max} ($CHCl_3$)/cm^{-1} 1676, 1650, 1309, 1152; δ_H (400 MHz; $CDCl_3$) 7.07 (1H, s, CH), 3.92 (3H, s, Me), 2.44 (3H, s, Me), 1.63 (9H, s, *t*Bu); δ_C (75 MHz; $CDCl_3$) 175.6, 173.3, 163.3, 141.1, 138.8, 137.2 (CH), 128.5, 122.1, 116.5, 82.1, 32.5 (Me), 28.1 (Me), 13.5 (Me); *m/z* (ESI) 378/376 (M+Na$^+$, 100/99 %).

Methyl 6-methoxy-2-methyl-1-(4-methylpiperazin-1-yl)-4,7-dioxo-4,7-dihydro-1*H*-indole-3-carboxylate (44a)

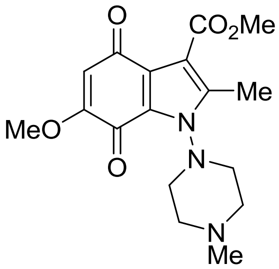

(a) 1-Amino-4-methylpiperazine (2.30 g, 20 mmol) was added to a stirred solution of methyl acetoacetate (2.32 g, 20 mmol) in dichloromethane (20 mL), and the resulting mixture was stirred at room temperature for 15 h and concentrated to give a crude mixture of the enamine and hydrazone tautomers of **43a** as a pale yellow oil (4.18 g, 98 %).

(b) The *title* compound was prepared by the general procedure from quinone **6a** (0.434 g, 2.0 mmol), the above hydrazone mixture (0.426 g, 2.0 mmol), copper(II) acetate monohydrate (0.798 g, 4.0 mmol) and potassium carbonate (0.828 g, 6.0 mmol) stirred at reflux in acetonitrile (20 mL) for 6 h. Column chromatography eluting with methanol, ethyl acetate and triethylamine (1:9:0.1) gave the *title compound* as an orange-yellow solid (0.407 g, 59 %); mp 197-199 °C; (Found: M+H$^+$, 348.1554. $C_{17}H_{22}N_3O_5$ requires 348.1554); ν_{max} ($CHCl_3$)/cm^{-1} 3006, 2951, 1722, 1668, 1652; δ_H (400 MHz; $CDCl_3$) 5.74 (1H, s, CH), 3.94 (3H, s, OMe), 3.92-3.87 (2H, m, 2CH), 3.84 (3H, s, OMe), 2.87 (2H, d, *J* 11.8, 2CH), 2.73 (2H, d, *J* 10.1, 2CH), 2.46 (3H, s, Me), 2.43-2.38 (2H, m, 2CH), 2.39 (3H, s, NMe); δ_C (75 MHz; $CDCl_3$) 181.6, 170.0, 164.8, 159.2, 143.6, 127.5, 124.9, 110.7, 106.6 (CH), 56.7 (Me), 55.0 (CH_2), 52.2 (Me), 50.5 (CH_2), 45.6 (Me), 11.1 (Me); *m/z* (ESI) 348 (M+H$^+$, 100 %).

Methyl 6-methoxy-2-methyl-1-morpholino-4,7-dioxo-4,7-dihydro-1H-indole-3-carboxylate (44b)

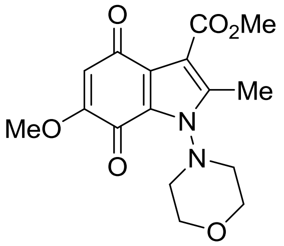

(a) 1-Aminomorpholine (1.02 g, 10 mmol) was added to a stirred solution of methyl acetoacetate (1.16 g, 10 mmol) in ethanol (10 mL), and the mixture was stirred at room temperature for 15 h, filtered through a silica pad and concentrated to give a crude mixture of enamine and hydrazone tautomers of **43b** as a pale yellow oil (1.550 g, 78 %).

(b) The *title* compound was prepared by the general procedure from quinone **6a** (0.109 g, 0.5 mmol), the above hydrazone mixture (0.100 g, 0.5 mmol), copper(II) acetate monohydrate (0.200 g, 1.0 mmol) and potassium carbonate (0.207 g, 1.5 mmol) stirred at reflux in acetonitrile (5 mL) for 7 h. Column chromatography eluting with ethyl acetate and dichloromethane (1:4) gave the *title compound* as an orange solid (0.098 g, 59 %); mp 207-209 °C; (Found: M+Na$^+$, 357.1056. $C_{16}H_{18}N_2O_6Na$ requires: 357.1057); ν_{max} ($CHCl_3$)/cm^{-1} 3011, 2862, 1723, 1668, 1653, 1612; δ_H (400 MHz; $CDCl_3$) 5.77 (1H, s, CH), 4.02-3.92 (4H, m, CH_2), 3.95 (3H, s, Me), 3.86 (3H, s, Me), 3.81-3.76 (m, 2H, CH_2), 2.72 (2H, s, *J* 9.3, CH_2), 2.49 (3H, s, Me); δ_C (75 MHz; $CDCl_3$) 181.4, 170.1, 164.7, 159.1, 143.5, 127.3, 125.0, 110.8,

106.7 (CH), 67.1 (CH_2), 56.7 (Me), 52.2 (Me), 51.6 (CH_2), 11.1 (Me); *m/z* (ESI) 357 ($M+Na^+$, 100 %).

Methyl 1,6-dimethoxy-2-methyl-4,7-dioxo-4,7-dihydro-1H-indole-3-carboxylate (44c)

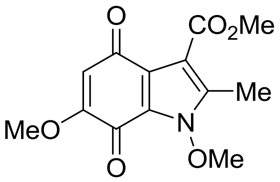

(a) Methoxylamine hydrochloride (0.913 g, 11 mmol) and triethylamine (1.111 g, 11 mmol) were added sequentially as single portions to a stirred solution of methyl acetoacetate (1.16 g, 10 mmol) in ethanol (10 mL), and the resulting mixture was stirred at room temperature for 14 h. The mixture was concentrated, diluted with water (15 mL) and extracted with dichloromethane (3 × 15 mL). The combined organic phases were dried ($MgSO_4$), filtered through a silica pad and concentrated to give the crude oxime ether **43c** as a colorless oil (0.895 g, 62 %).

(b) The *title compound* was prepared by the general procedure from quinone **6a** (0.109 g, 0.5 mmol), the above oxime mixture (0.073 g, 0.5 mmol), copper(II) acetate monohydrate (0.200 g, 1.0 mmol) and sodium *t*-butoxide (0.144 g, 1.5 mmol) stirred at reflux in acetonitrile (5 mL) for 6.5 h. Column chromatography eluting with ethyl acetate and dichloromethane (1:6) gave the *title compound* as an orange solid (0.022 g, 16 %), mp 184-186 °C; (Found: 302.0634. $C_{13}H_{13}NO_6Na$ requires: 3002.0635); ν_{max} ($CHCl_3$)/cm^{-1} 3008, 2942, 1724, 1674, 1653, 1608, 1491, 1107; δ_H (400 MHz; $CDCl_3$) 5.78 (1H, s, CH), 4.14 (3H, s, Me), 3.94 (3H, s, Me), 3.85 (3H, s, Me), 2.52 (3H, s, Me); δ_C (75 MHz; $CDCl_3$) 180.6, 169.8, 163.8, 158.4, 138.8, 123.7, 120.6, 108.8, 107.9 (CH), 66.7 (Me), 56.6 (Me), 52.1 (Me), 9.5 (Me); *m/z* (ESI) 302 ($M+Na^+$, 100 %), 581 ($2M+Na^+$, 51).

3-Hydroxymethyl-6-methoxy-1,2-dimethyl-1*H*-indole-4,7-dione (9)

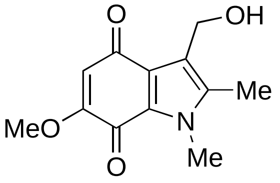

(a) A solution of sodium dithionite (6.615 g, 38.0 mmol) in water (70 mL) was added as a single portion to a solution of indolequinone **8** (2.00 g, 7.6 mmol) in chloroform (70 mL), and the resulting mixture was stirred vigorously at room temperature under argon for 2 h, and

extracted with dichloromethane (3 × 50 mL). The combined organic phases were dried ($MgSO_4$), filtered and concentrated to give the crude hydroquinone as a white solid.

(b) To a solution of this solid in THF (80 mL) was added portionwise over 5 min lithium aluminium hydride (1.452 g, 38.0 mmol). The resulting mixture was stirred at room temperature for 2 h and quenched by sequential slow addition of ethyl acetate (10 mL), water (5 mL), aqueous NaOH (1 M; 5mL) and silica gel (approx. 3 g). The mixture was filtered and concentrated *in vacuo*, and the residue was dissolved in methanol (70 mL). A solution of iron(III) chloride hexahydrate (10.265 g, 38.0 mmol) in water (70 mL) was added as a single portion, and the mixture was stirred at room temperature for 1 h, concentrated to half its volume *in vacuo* and extracted with dichloromethane (4 × 40 mL). The combined organic phases were dried ($MgSO_4$), filtered and concentrated. Chromatography of the residue eluting with dichloromethane and ethyl acetate (5:1) gave the product as a red-orange solid (0.875 g, 49%); mp 196-198 °C (lit.,[17] 197-199 °C); (Found: $M+Na^+$, 258.0749. $C_{12}H_{13}NO_4Na$ requires 258.0737); δ_H (400 MHz; $CDCl_3$) 5.70 (1H, s, 5-H), 4.61 (2H, s, CH_2), 4.50 (1H, br s, OH), 3.91 (3H, s, Me), 3.85 (3H, s, Me), 2.26 (3H, s, Me); δ_C (75 MHz; $CDCl_3$) 185.6, 170.1, 160.5, 136.8, 127.5, 124.8, 123.3, 106.1 (CH), 56.7 (Me), 56.0 (CH_2), 32.6 (Me), 9.8 (Me); *m/z* (ESI) 258 ($M+Na^+$, 100%).

References

(1) Morita, I.; Haruta, Y.; Tomita, T.; Tsuda, M.; Kandori, K.; Kise, M.; Kimura, K. *Chem. Pharm. Bull,.* **1987**, *35*, 4819-4828.

(2) Antonov, V. K.; Berlin, A. Y. *Zh. Obshch. Khim.*, **1959**, *29*, 4003-4010.

(3) Langer, P.; Eckhardt, T.; *Synlett*, **2000**, 844-846.

(4) Thansandote, P.; Gouliaras, C.; Turcotte-Savard, M. O.; Lautens, M. *J. Org. Chem.* **2009**, *74*, 1791-1793.

(5) Chhabra, S. R.; Mahajan, A.; Chan, W. C. *J. Org. Chem.*, **2002**, *67*, 4017-4029.

(6) Chen, Y.L.; Mariano, P.S.; Little, G.M.; O'Brien, D; Huesmann, P.L.; *J. Org. Chem.*, **1981,** *46*, 4643-4654.

(7) Sridharan, V.; Ruiz, M.; Menendez, J.C.; *Synthesis*, **2010,** 1053-1057.

(8) Edmondson, S.D.; Mastracchio, A.; Parmee, E.R.; *Org. Lett.*, **2000,** *2*, 1109-1112.

(9) Huang, J; Liang, Y.; Pan, W.; Yang, Y.; Dong, D.; *Org. Lett.*, **2009**, *9*, 5345-5348.

(10) Shinozuka, T.; Yamamoto, Y.; Hasegawa, T.; Sato, K.; Naito, S.; *Tetrahedron Lett.*, **2008**, *49*, 1619-1622.

(11) Lang, M.; Muhlbauer, A.; Graf, C.; Beyer, J.; Lang-Fugmann, S.; Polborn, K.; Steglich, W. *Eur. J. Org. Chem.*, **2008**, 816-825.

(12) Saa, J. M.; Morey, J.; Costa, A. *Tetrahedron Lett.*, **1986**, *27*, 5125-5128.

(13) Sutherland, H. S.; Higgs, K. C.; Taylor, N. J.; Rodrigo, R. *Tetrahedron,* **2001**, *57*, 309-317.

(14) Perumal, P. T.; Bhatt, M. V. *Synthesis,* **1979**, 205-206.

(15) Esser, T.; Farkas, F.; Mangholz, S.; Séquin, U.; *Tetrahedron*, **1994,** *50*, 3709-3720.

(16) Janusz, J. M.; Young, P. A.; Enzweiler, K.; Wu, L. I.; Gan, L.; Pikul, S.; McDow-Dunham, K. L.; Johnson, C. R.; Senanayake, C. B.; Kellstein, D. E.; Green, S. A.; Tulich, J. L.; Rosario-Jansen, T.; Magrisso, I. J.; Wehmeyer, K. R.; Kuhlenbeck, D. L.; Eichold, T. H.; Dobson, R. L. M.; *J. Med. Chem.*, **1998**, *41*, 1124-1137.

(17) Colucci, M. A.; Reigan, P.; Siegel, D.; Chilloux, A.; Ross, D.; Moody, C. J. *J. Med. Chem.*, **2007**, *50*, 5780-5789.

(E/Z)-Ethyl 4-allyloxy-3-(methylamino)but-2-enoate (7c)

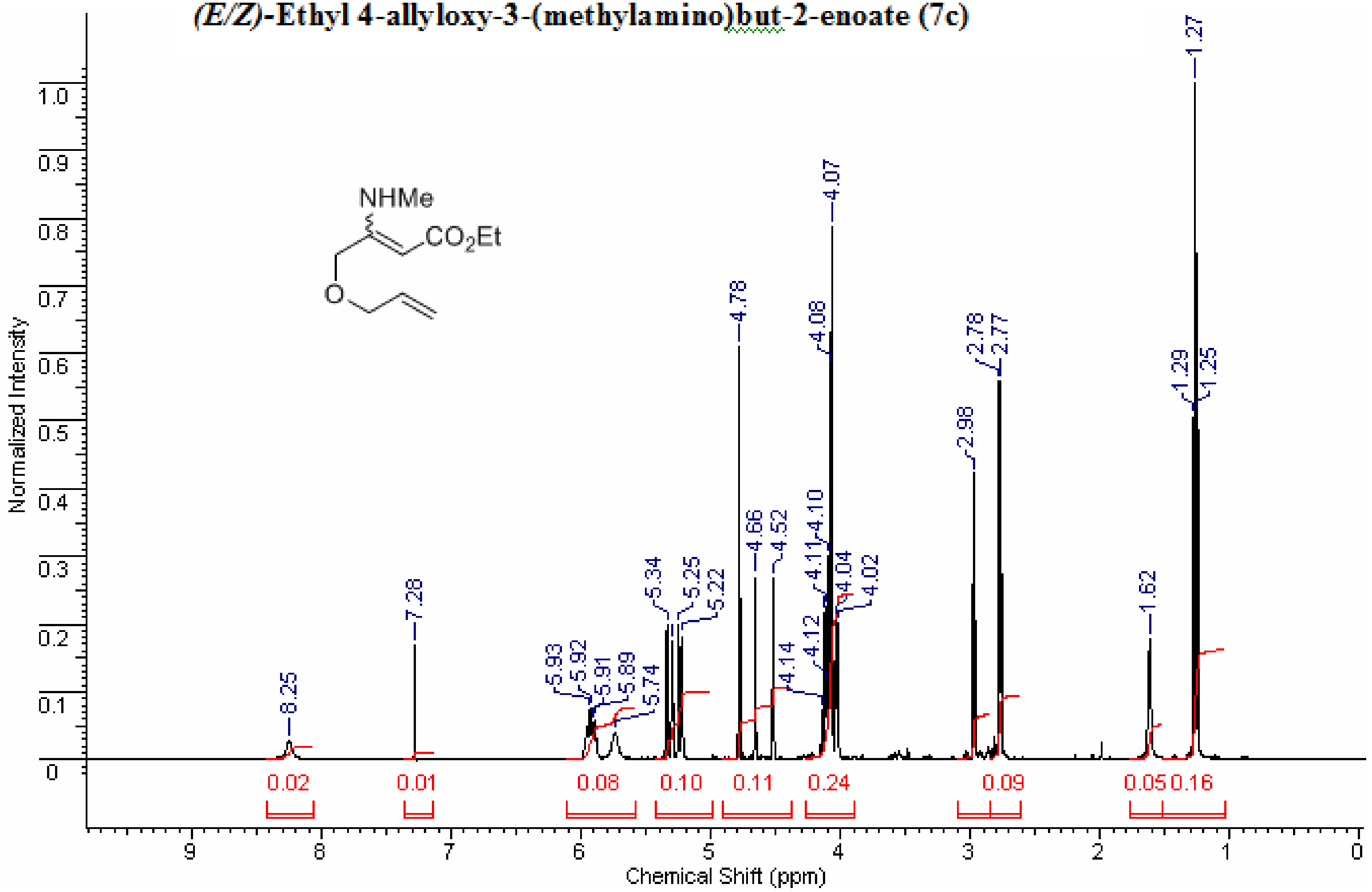

***(E/Z)*-Ethyl 4-allyloxy-3-(methylamino)but-2-enoate (7c)**

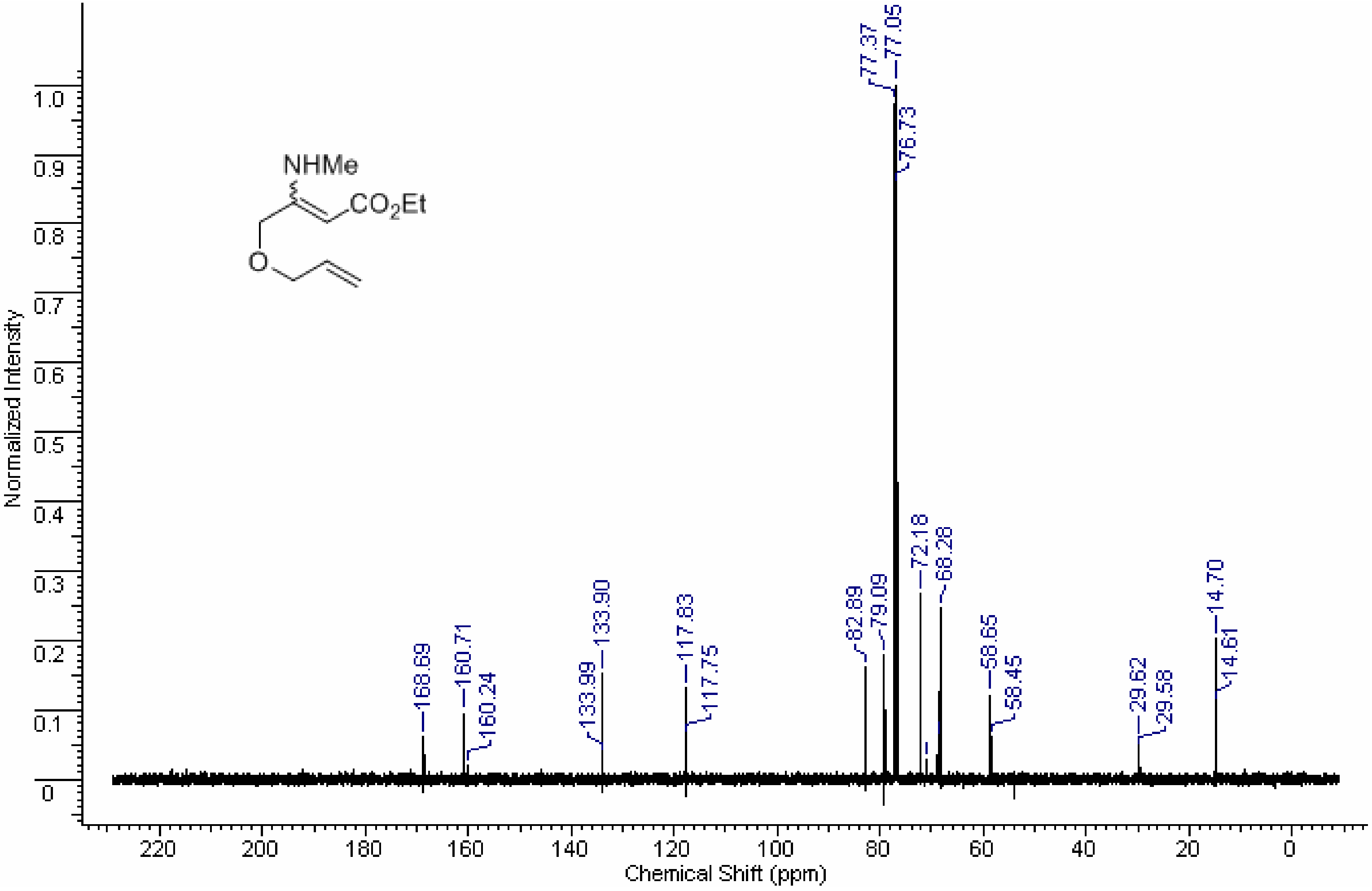

(Z)-*tert*-Butyl 3-(methylamino)but-2-enoate (7d)

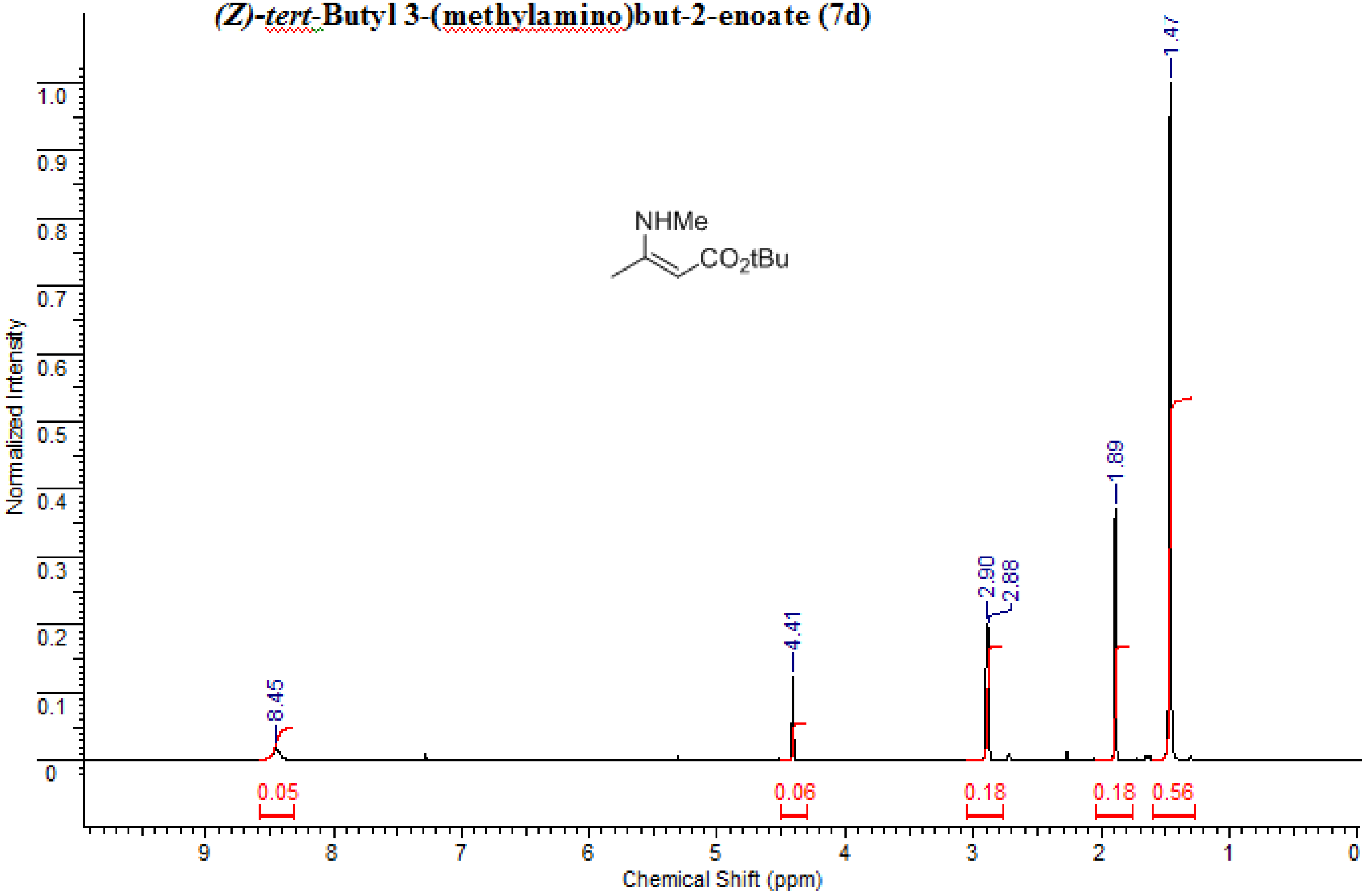

***(Z)-tert*-Butyl 3-(methylamino)but-2-enoate (7d)**

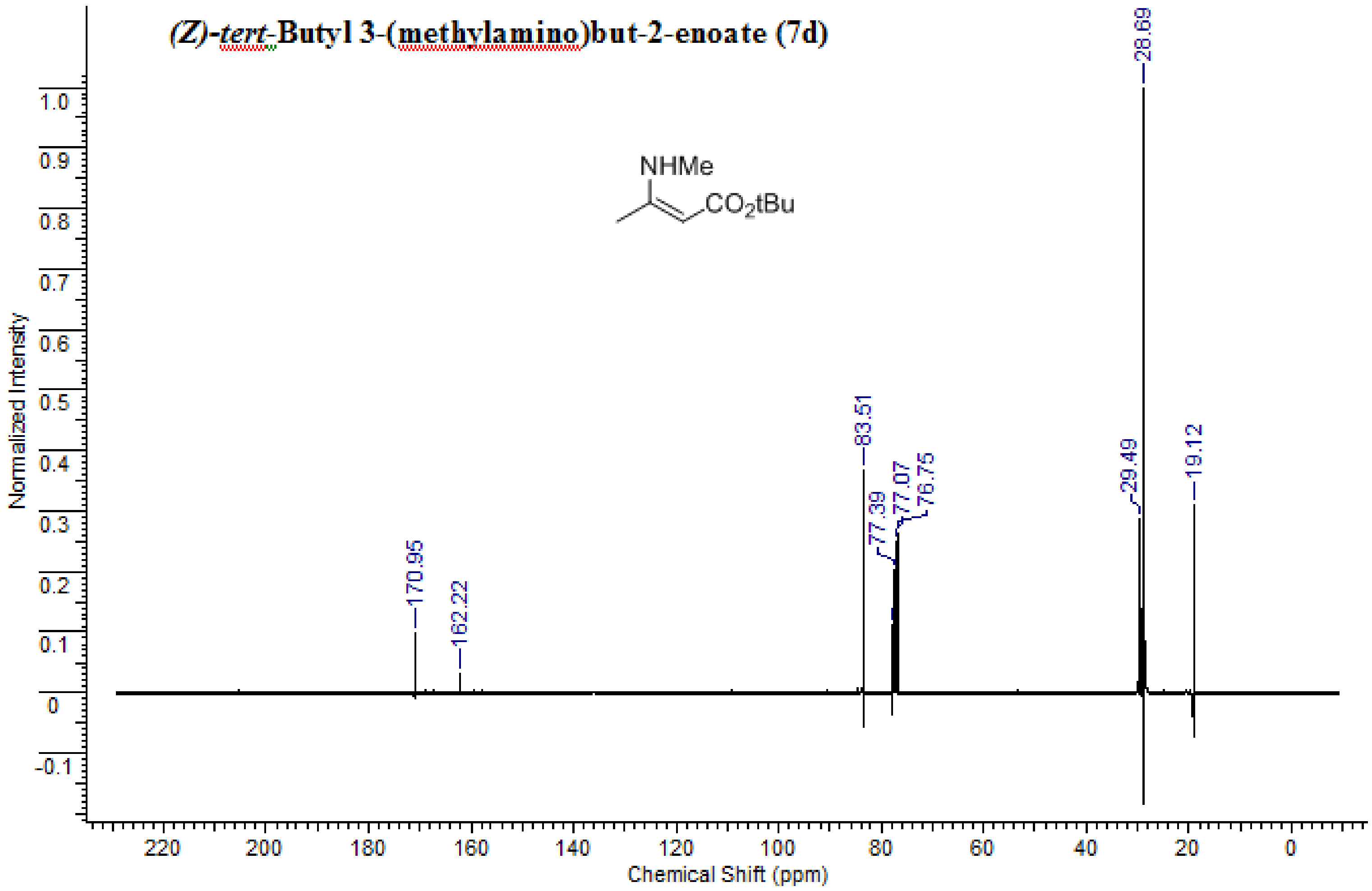

(Z)-Methyl 3-(4-methoxybenzylamino)but-2-enoate (7e)

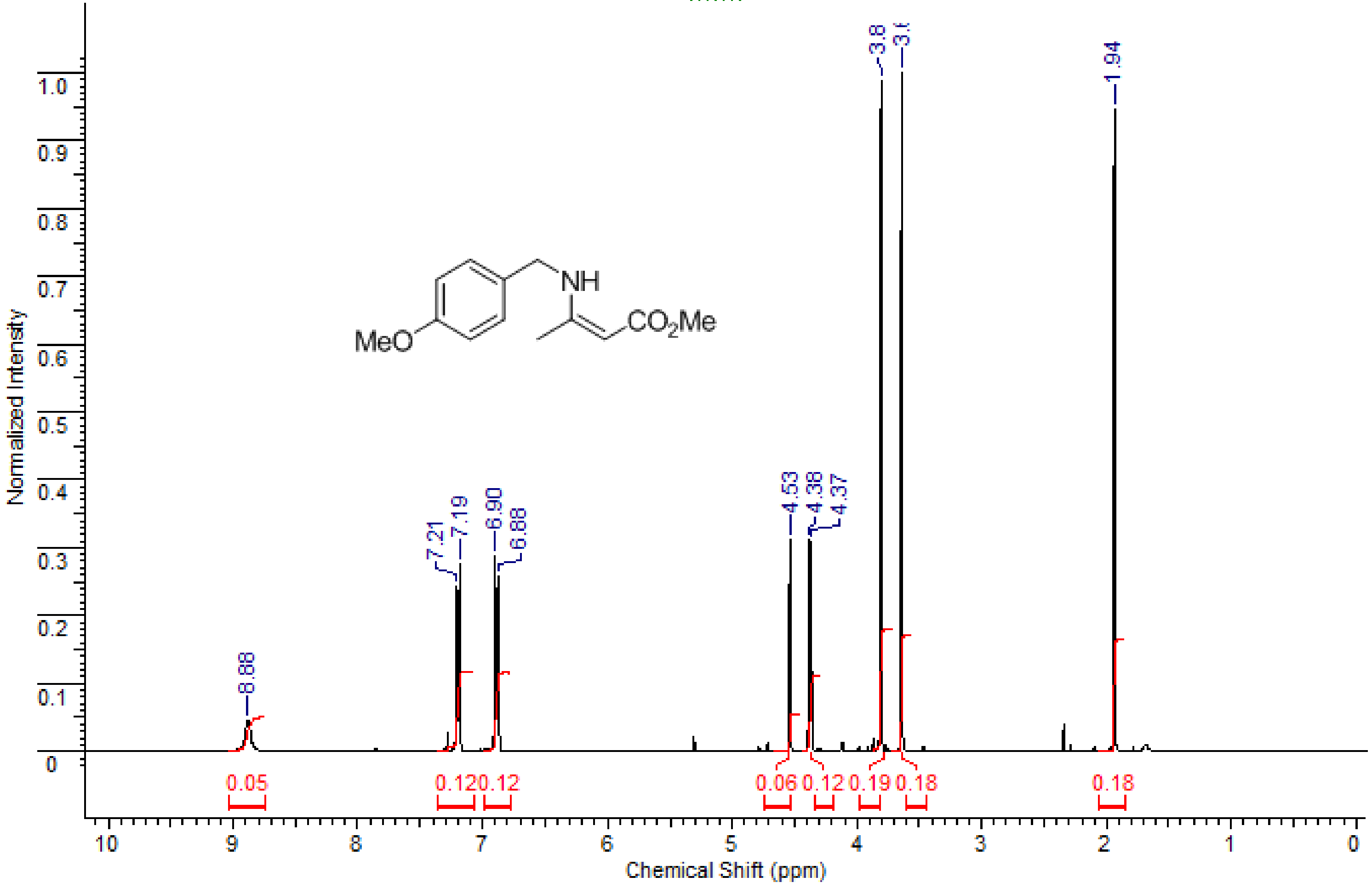

(*Z*)-Methyl 3-(4-methoxybenzylamino)but-2-enoate (7e)

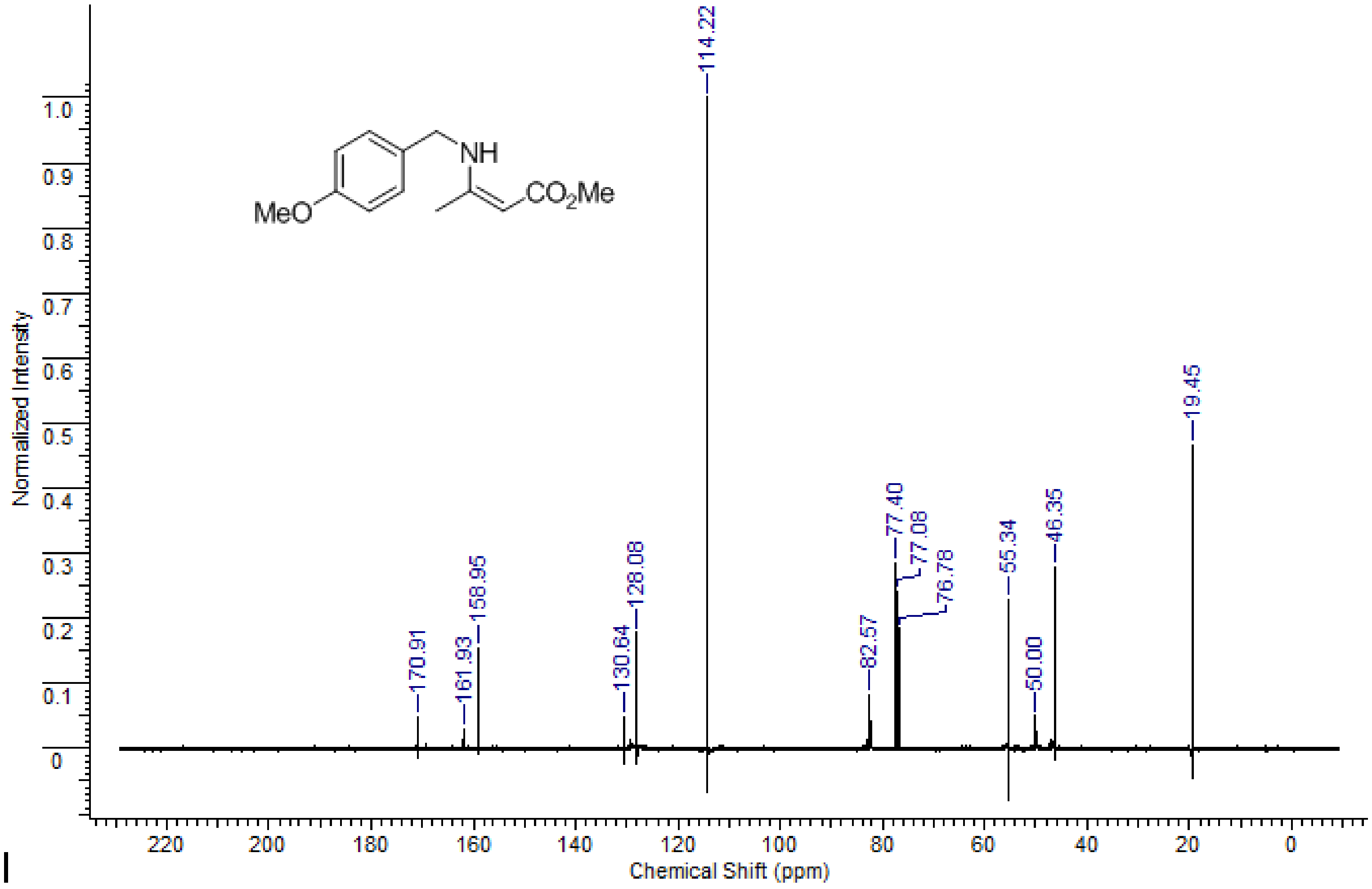

(Z)-*tert*-Butyl 3-(3-methoxyphenylamino)but-2-enoate (7f)

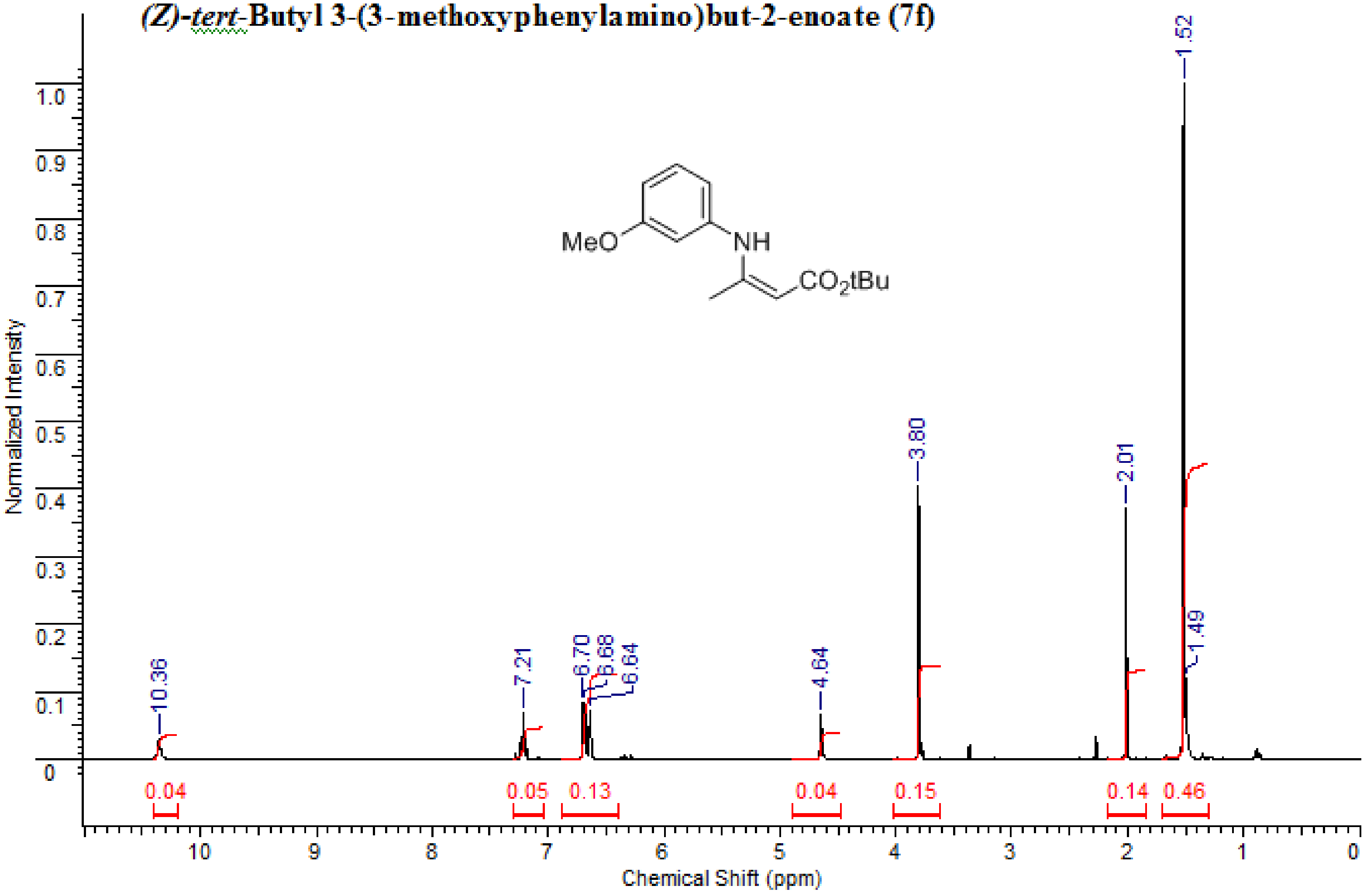

(Z)-*tert*-Butyl 3-(3-methoxyphenylamino)but-2-enoate (7f)

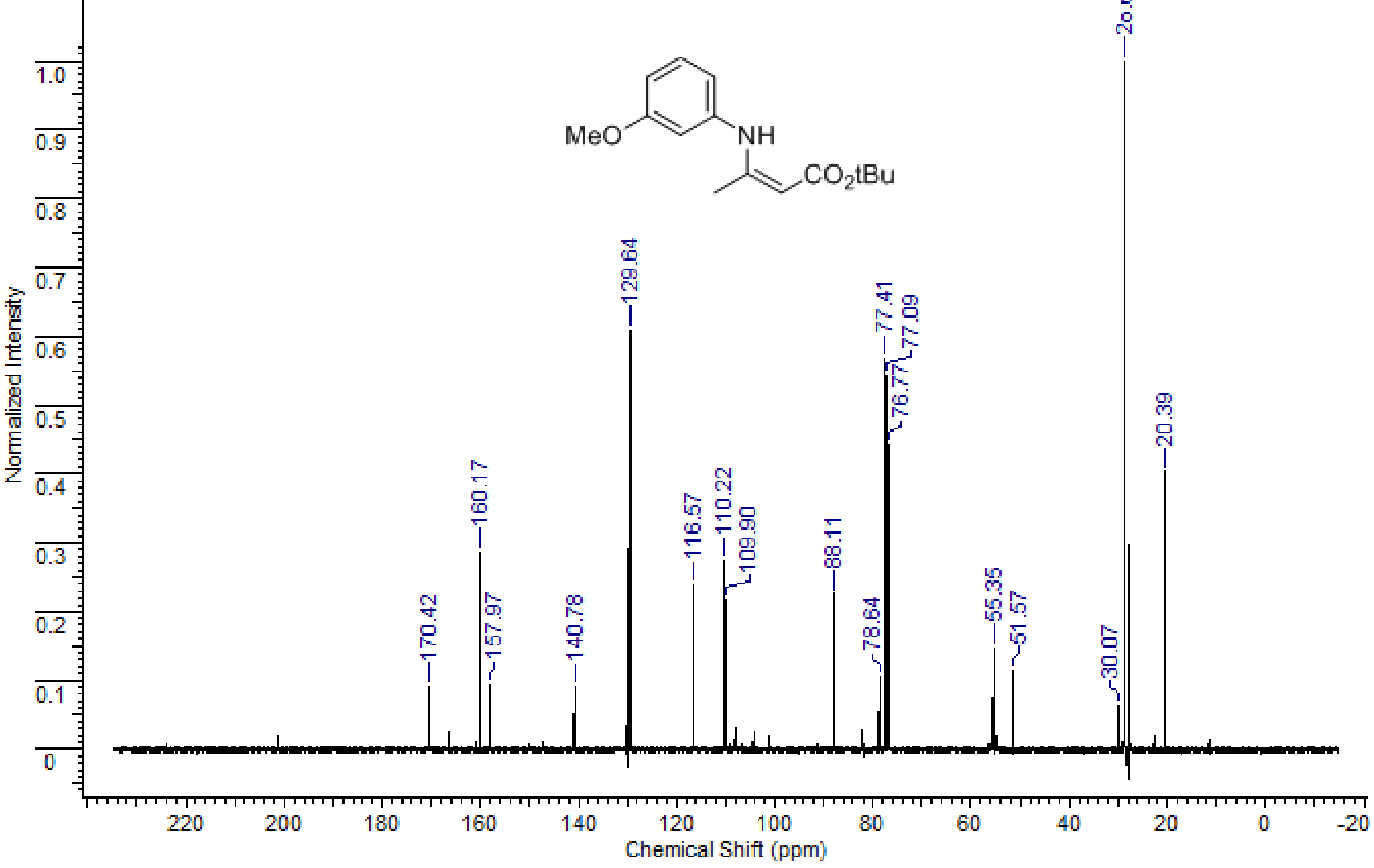

(Z)-*tert*-Butyl 3-(2-morpholinoethylamino)but-2-enoate (7g)

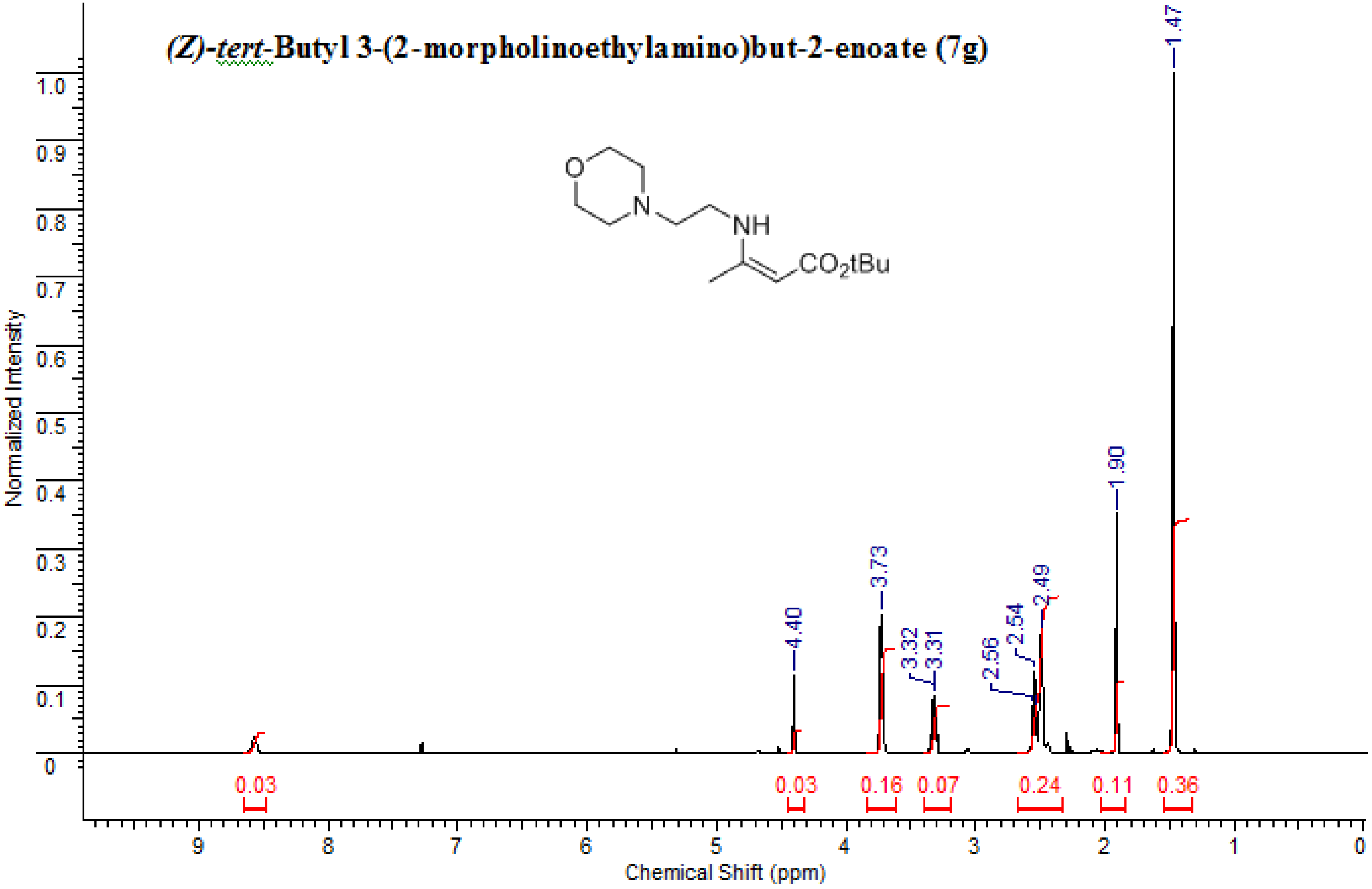

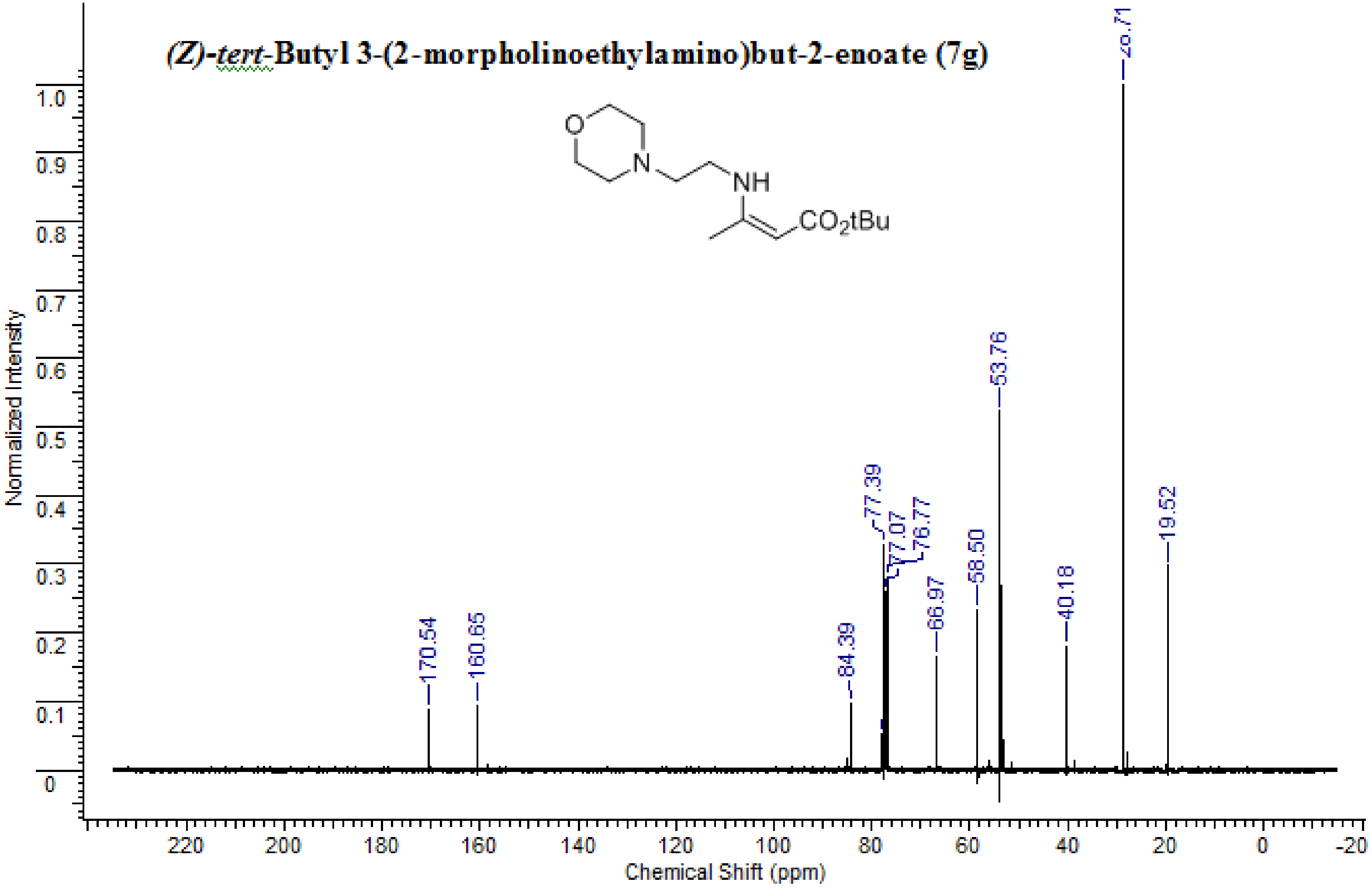
(Z)-tert-Butyl 3-(2-morpholinoethylamino)but-2-enoate (7g)
O
N
NH
CO2tBu
170.54
160.65
84.39
77.39
77.07
76.77
66.97
58.50
53.76
40.18
19.52
Normalized Intensity
Chemical Shift (ppm)

(Z)-Dimethyl 2-(4-methoxybenzylamino)fumarate (7h)

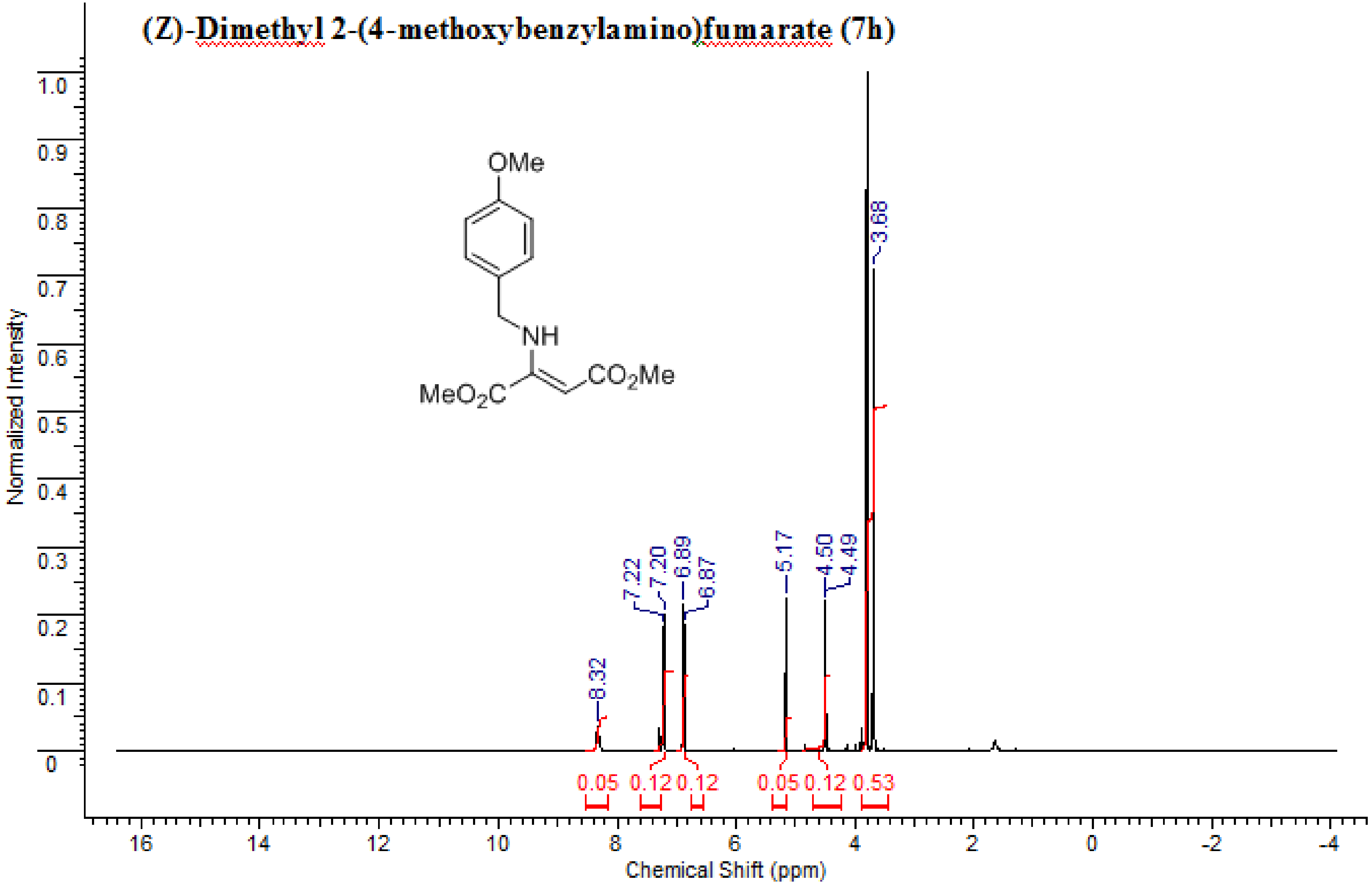

(Z)-Dimethyl 2-(4-methoxybenzylamino)fumarate (7h)

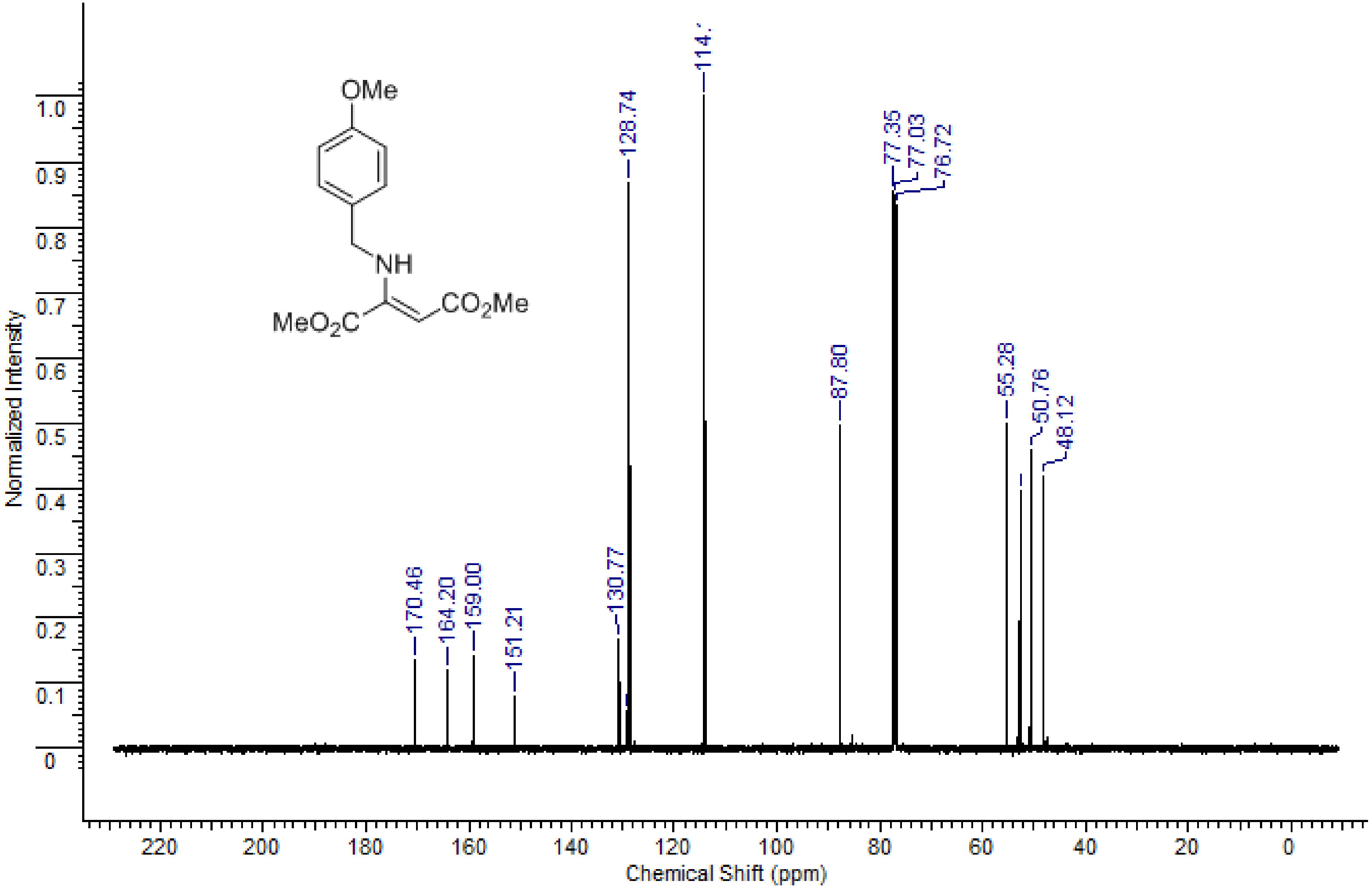

(*Z*)-Methyl 3-(2-(*tert*-butyldimethylsiloxy)ethylamino)but-2-enoate (7i)

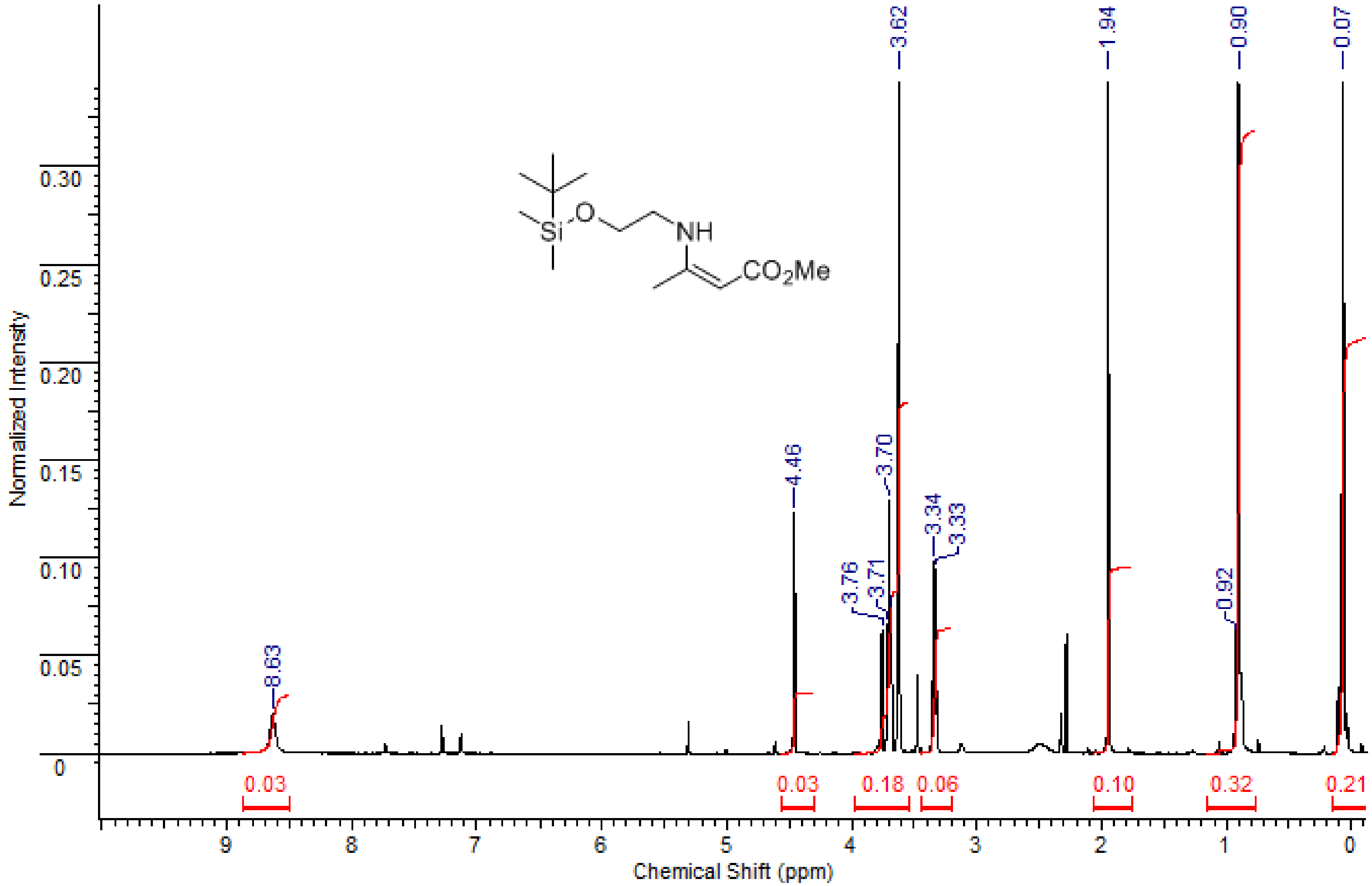

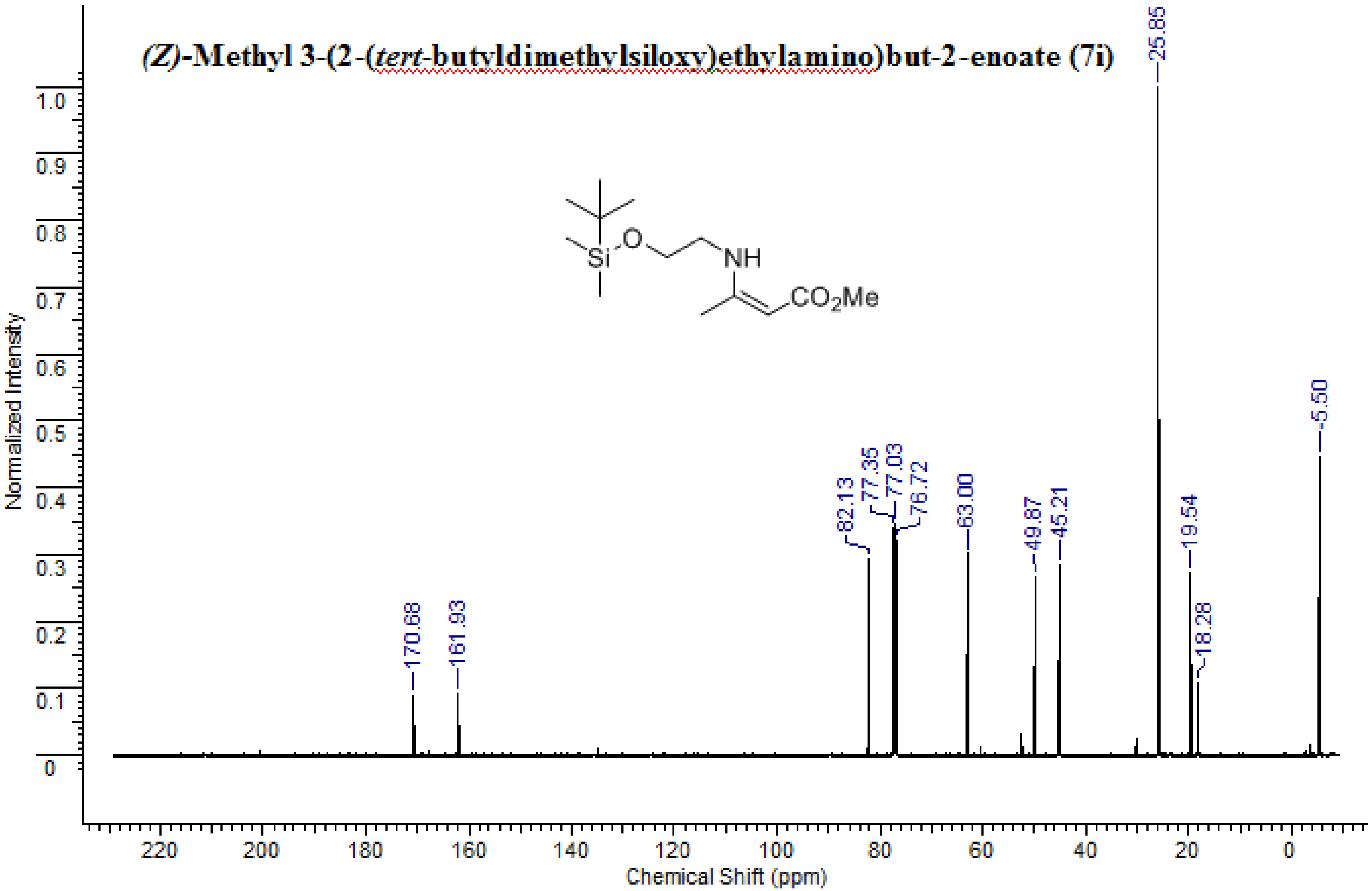

(Z)-Methyl 3-(2-(tert-butyldimethylsiloxy)ethylamino)but-2-enoate (7i)
Si
O
NH
CO2Me
170.68
161.93
82.13
77.35
77.03
76.72
63.00
49.87
45.21
25.85
19.54
18.28
-5.50
Normalized Intensity
1.0
0.9
0.8
0.7
0.6
0.5
0.4
0.3
0.2
0.1
0
220
200
180
160
140
120
100
80
60
40
20
0
Chemical Shift (ppm)

(*Z*)-Methyl 3-(2-(*tert*-butoxycarbonylamino)ethylamino)but-2-enoate (7j)

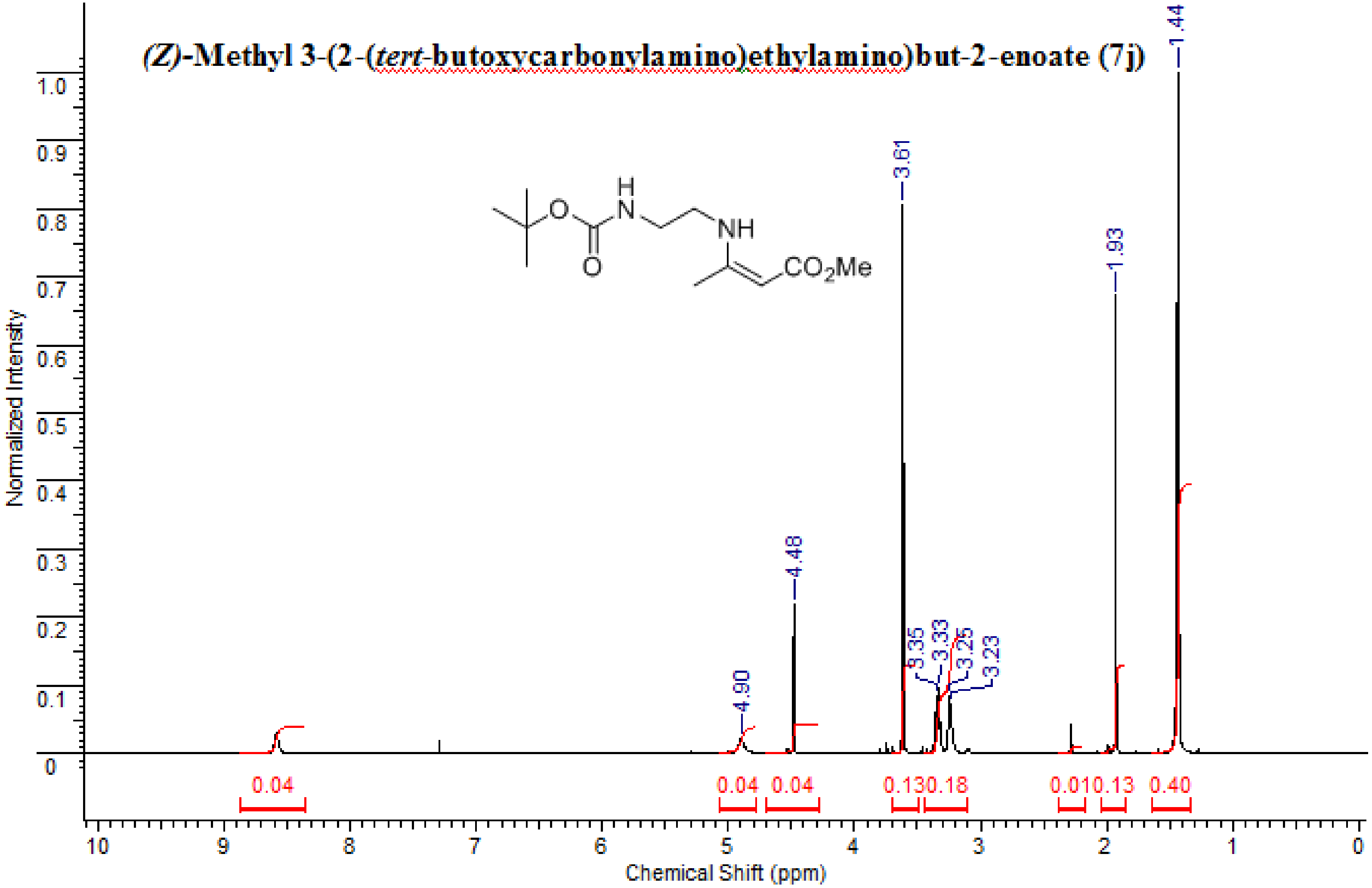

(*Z*)-Methyl 3-(2-(*tert*-butoxycarbonylamino)ethylamino)but-2-enoate (7j)

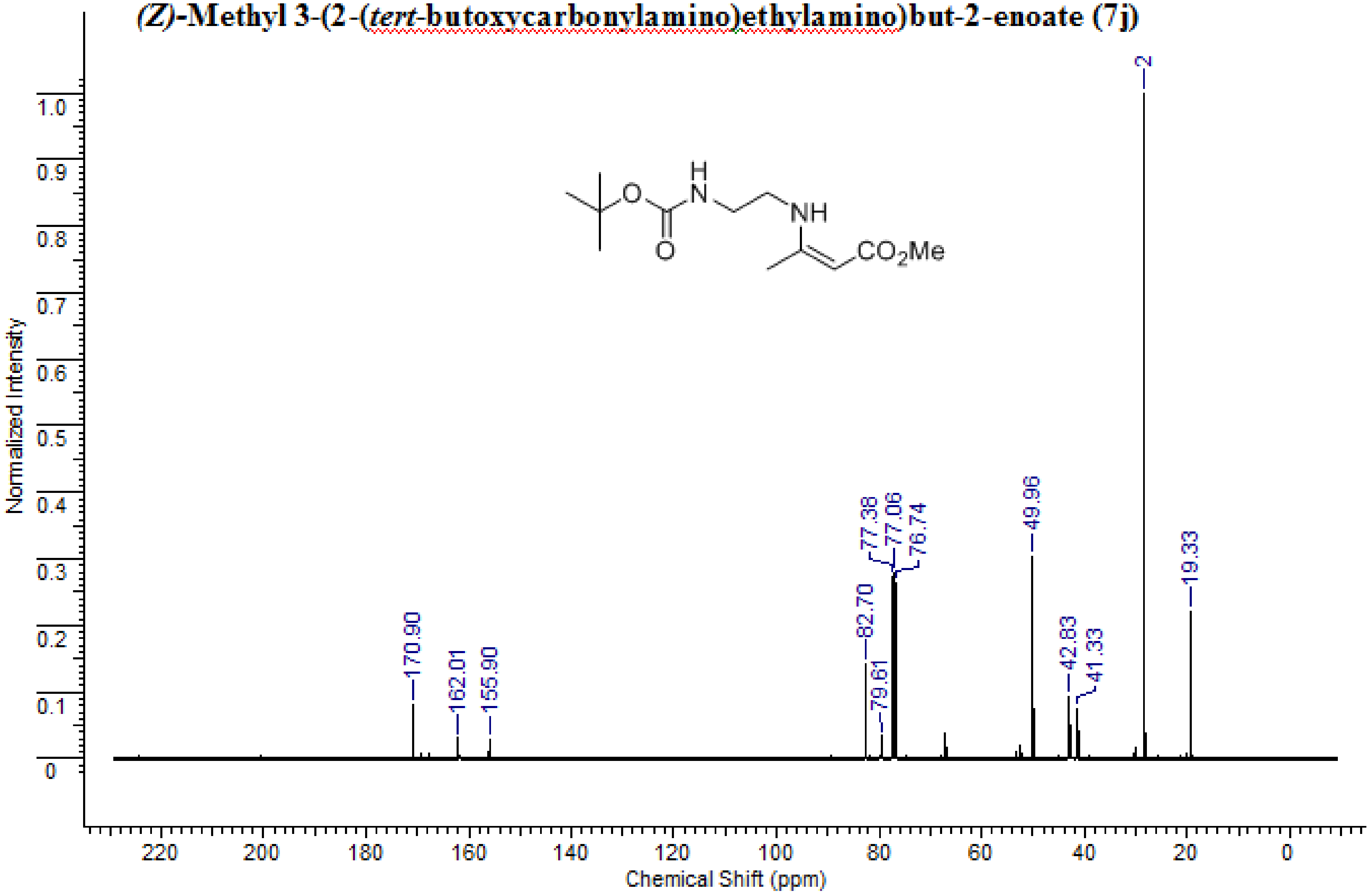

Ethyl 4-(4-methoxybenzyloxy)-3-oxobutyrate

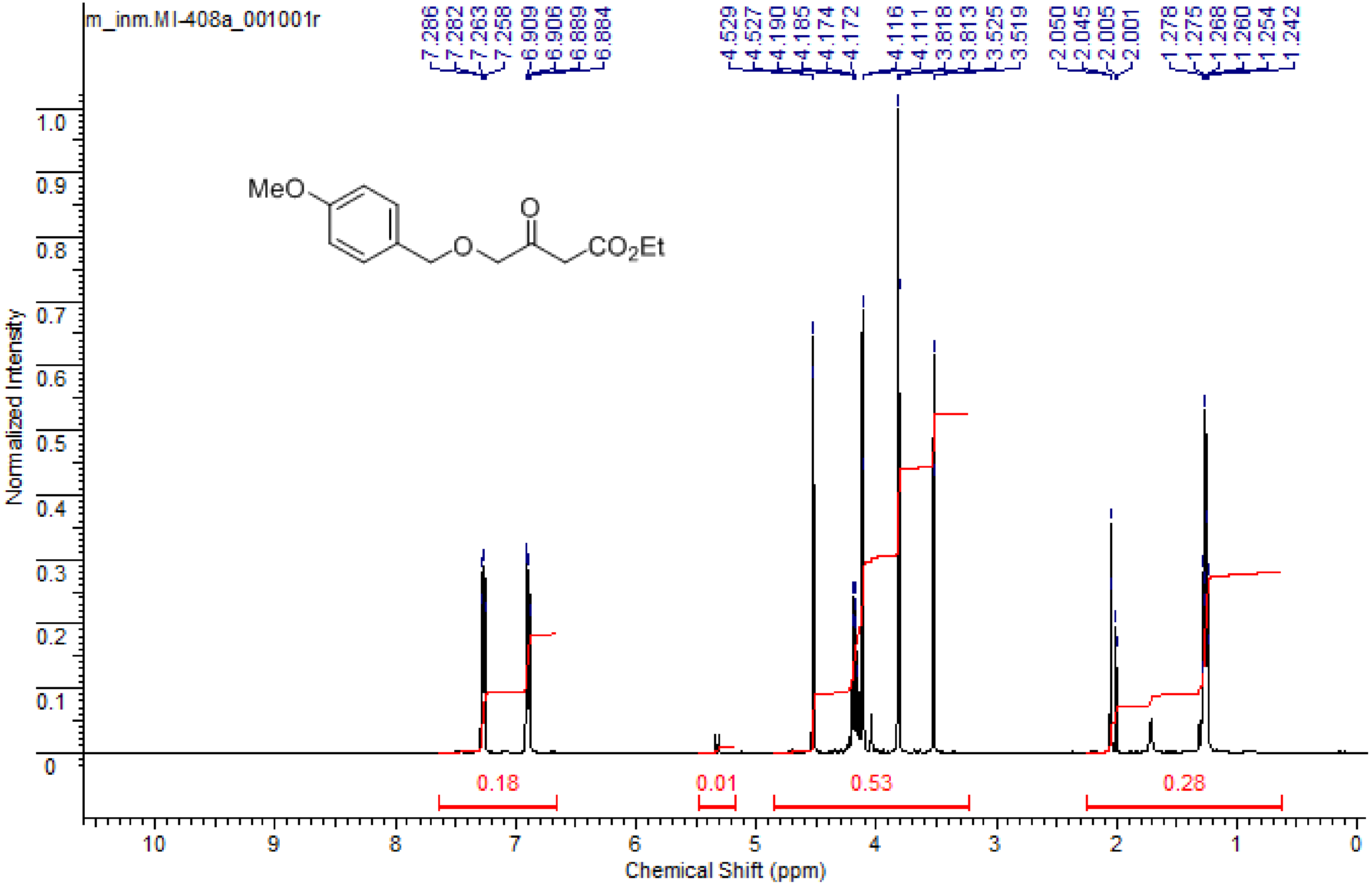

Ethyl 4-(4-methoxybenzyloxy)-3-oxobutyrate

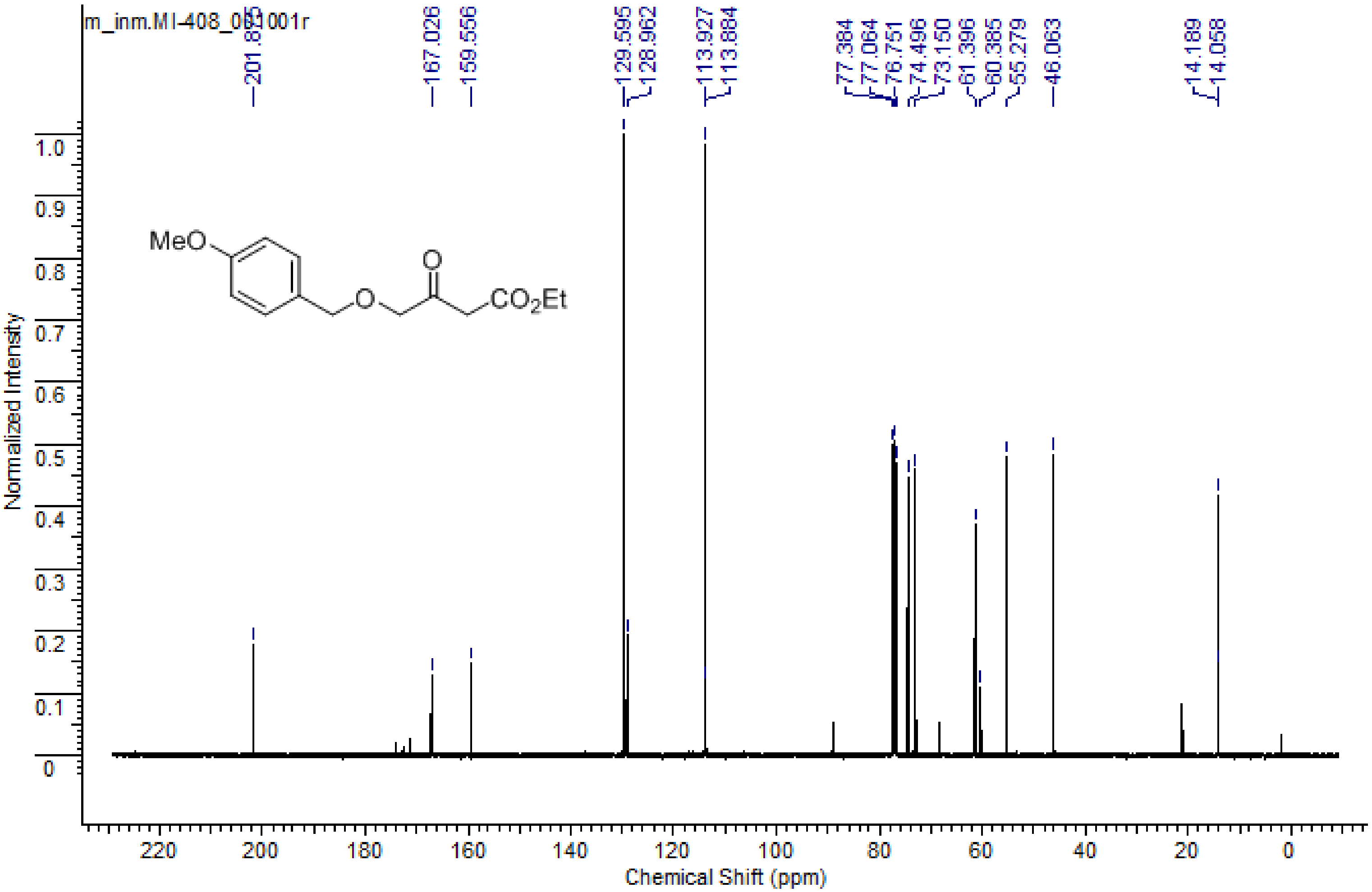

(E/Z)-**Ethyl 4-(4-methoxybenzyloxy)-3-(methylamino)but-2-enoate (7k)**

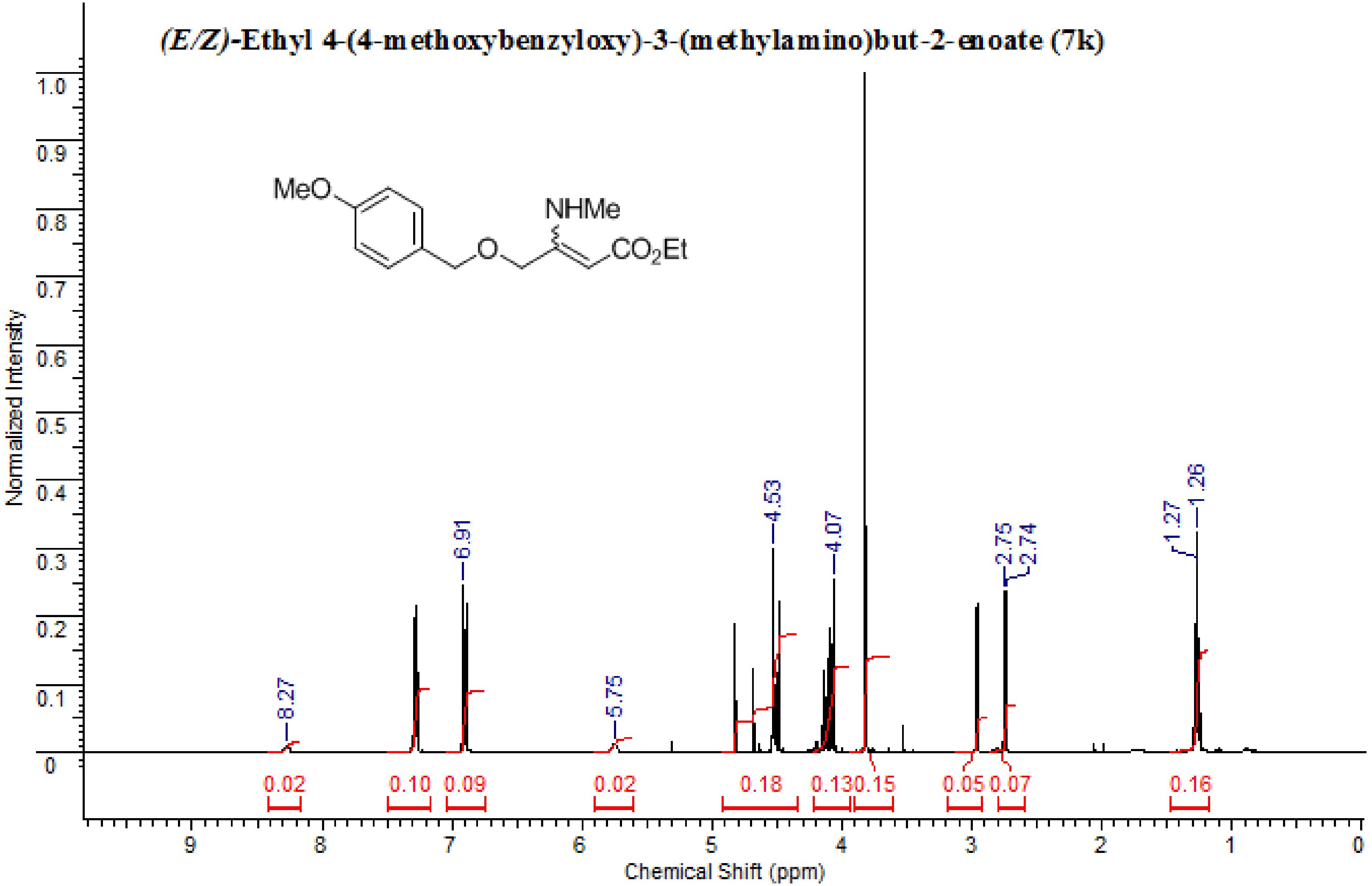

(E/Z)-Ethyl 4-(4-methoxybenzyloxy)-3-(methylamino)but-2-enoate (7k)

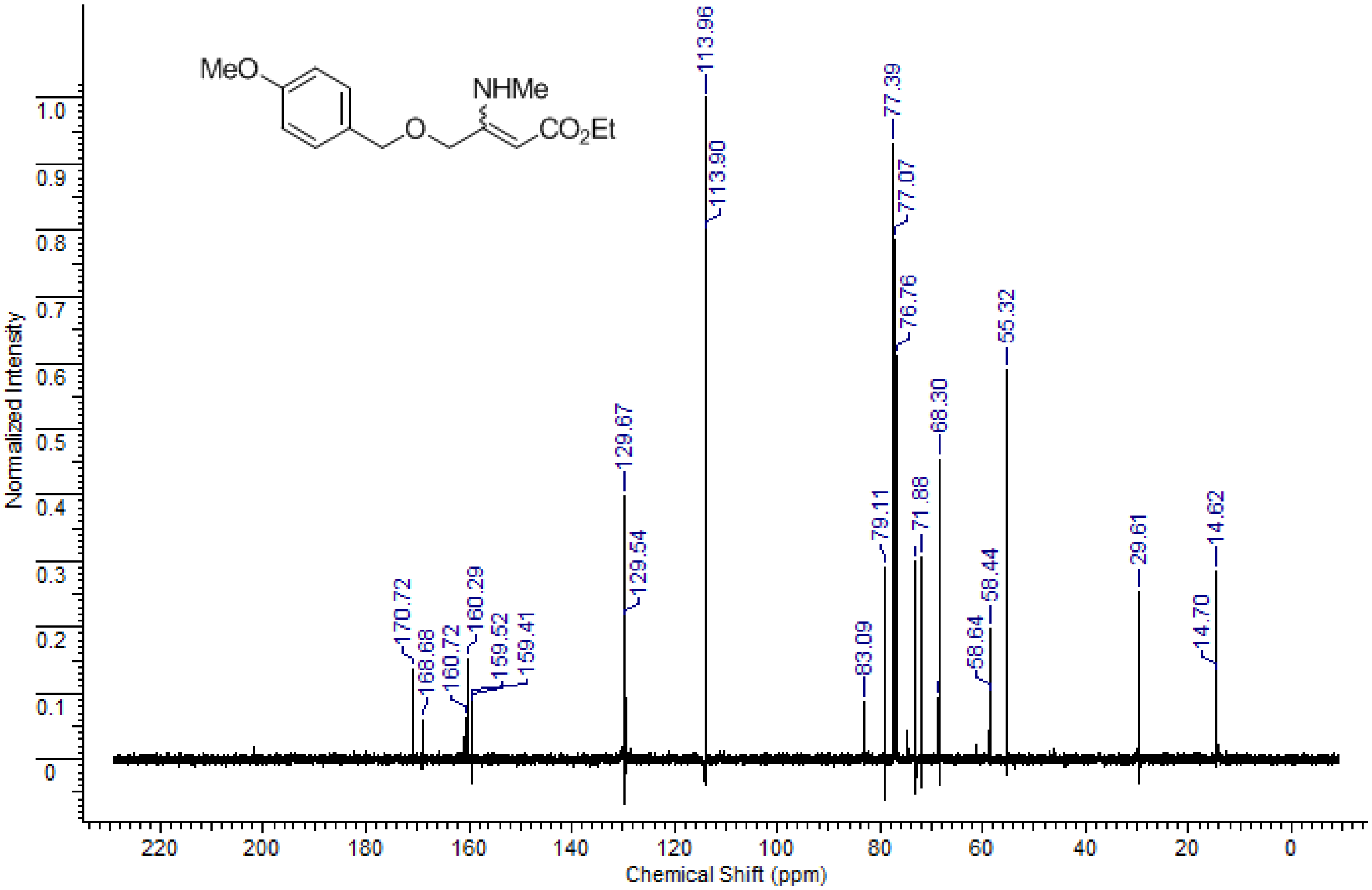

(Z)-*tert*-Butyl 3-(2-(1H-indol-3-yl)ethylamino)but-2-enoate (7m)

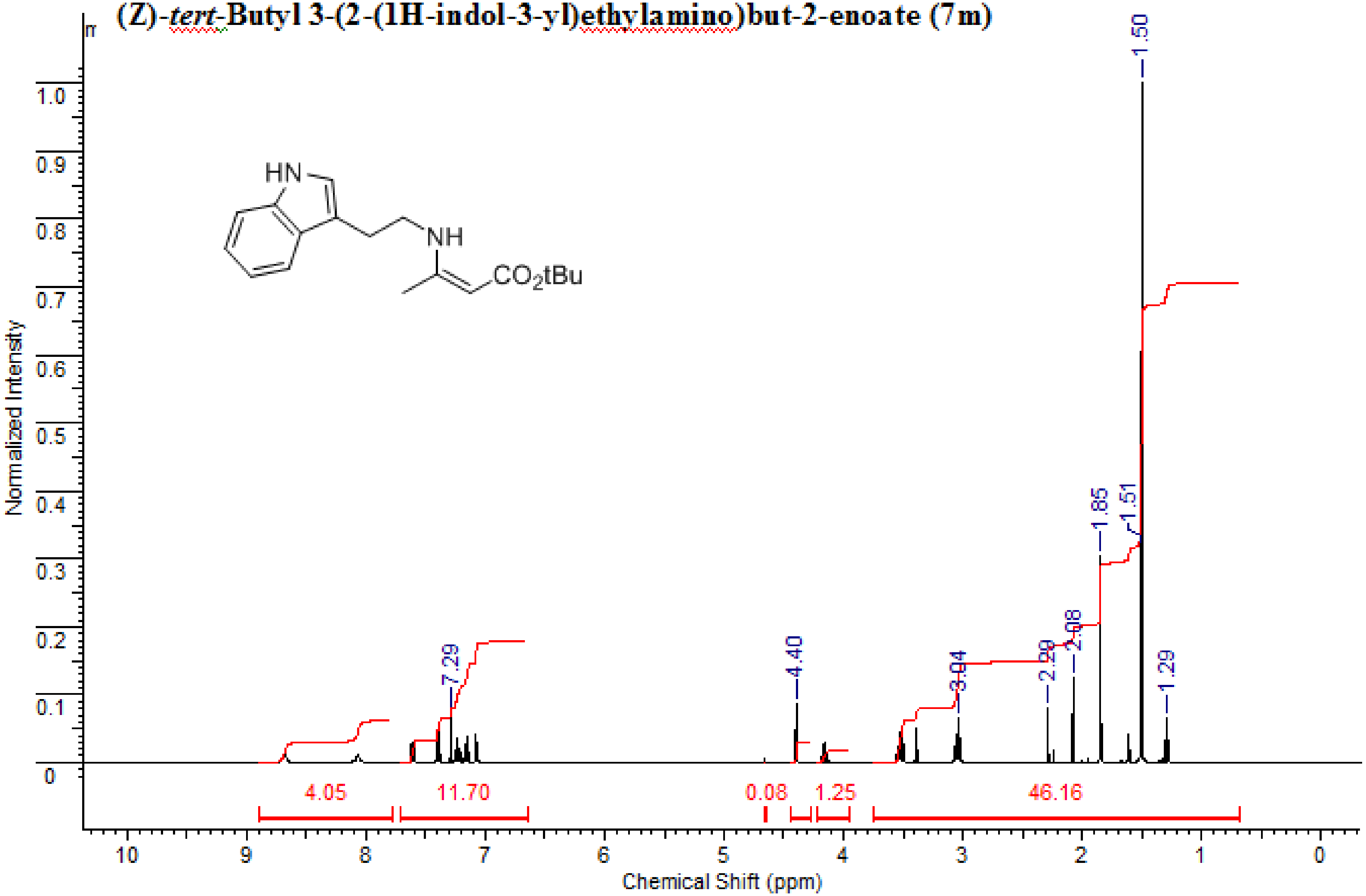

(Z)-*tert*-Butyl 3-(2-(1H-indol-3-yl)ethylamino)but-2-enoate (7m)

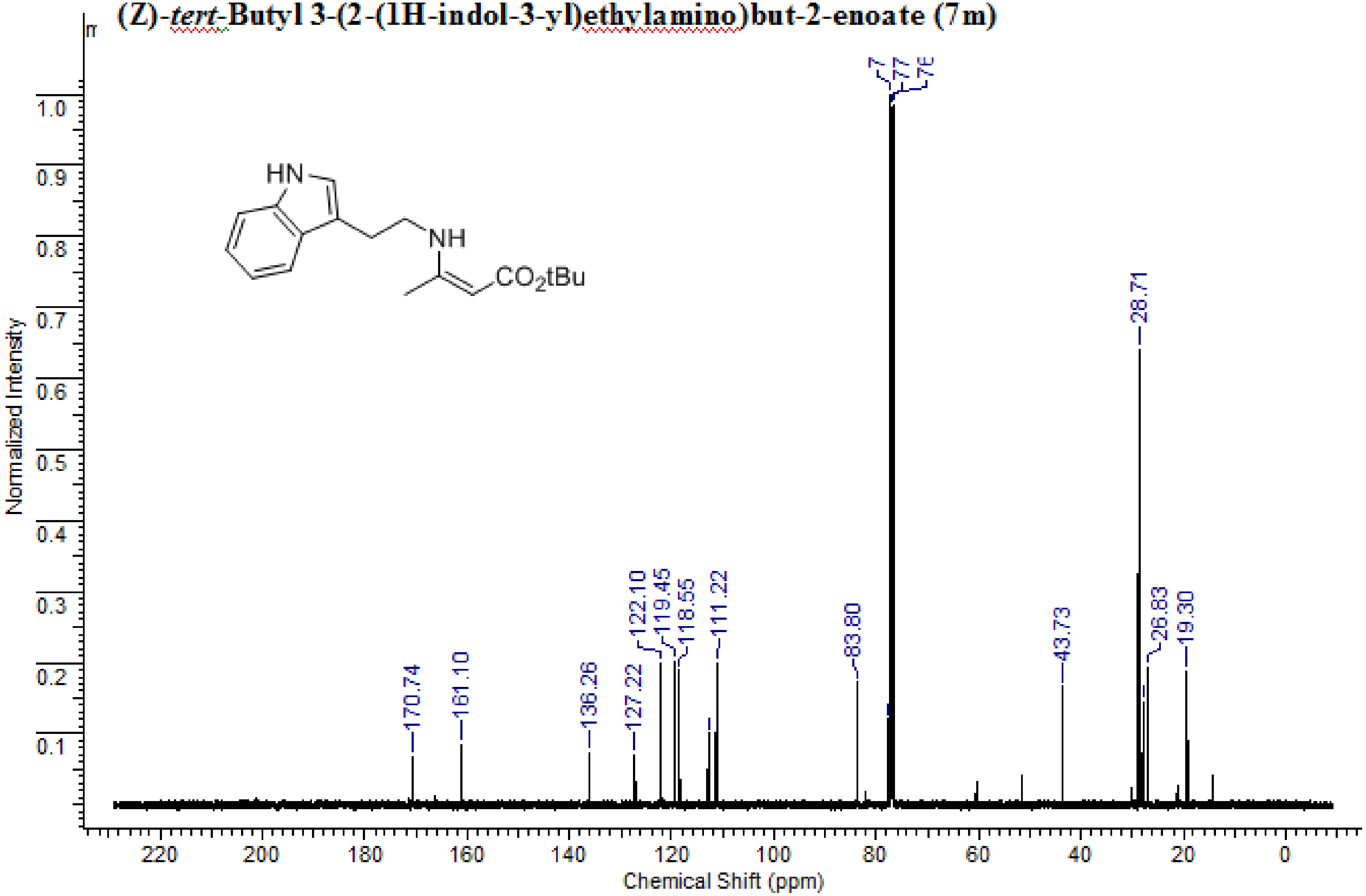

(S)-(E,Z)-Methyl 2-(4-*tert*-butoxy-4-oxobut-2-en-2-ylamino)-4-methylpentanoate (7n)

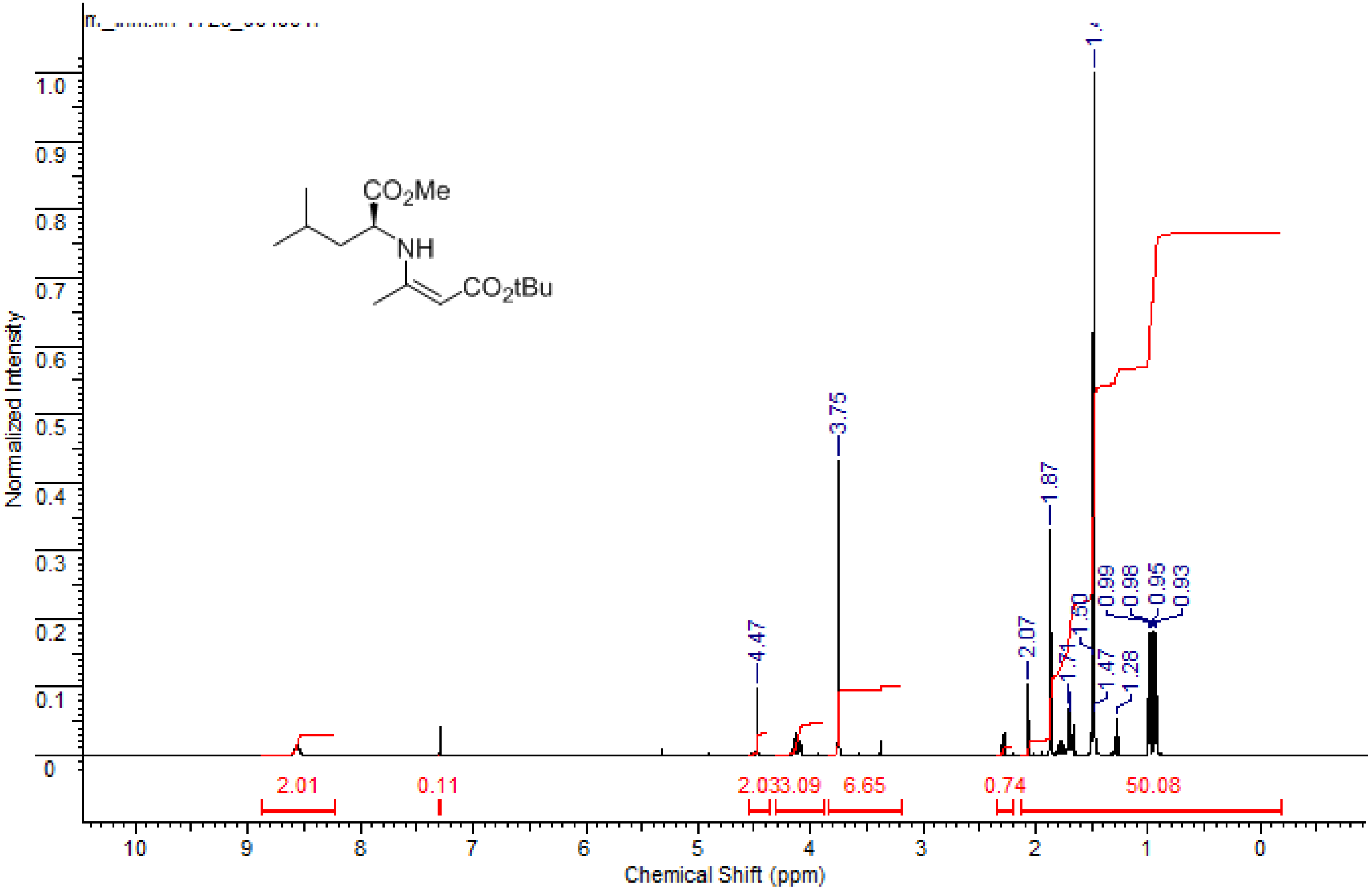

(S)-(E,Z)-Methyl 2-(4-*tert*-butoxy-4-oxobut-2-en-2-ylamino)-4-methylpentanoate (7n)

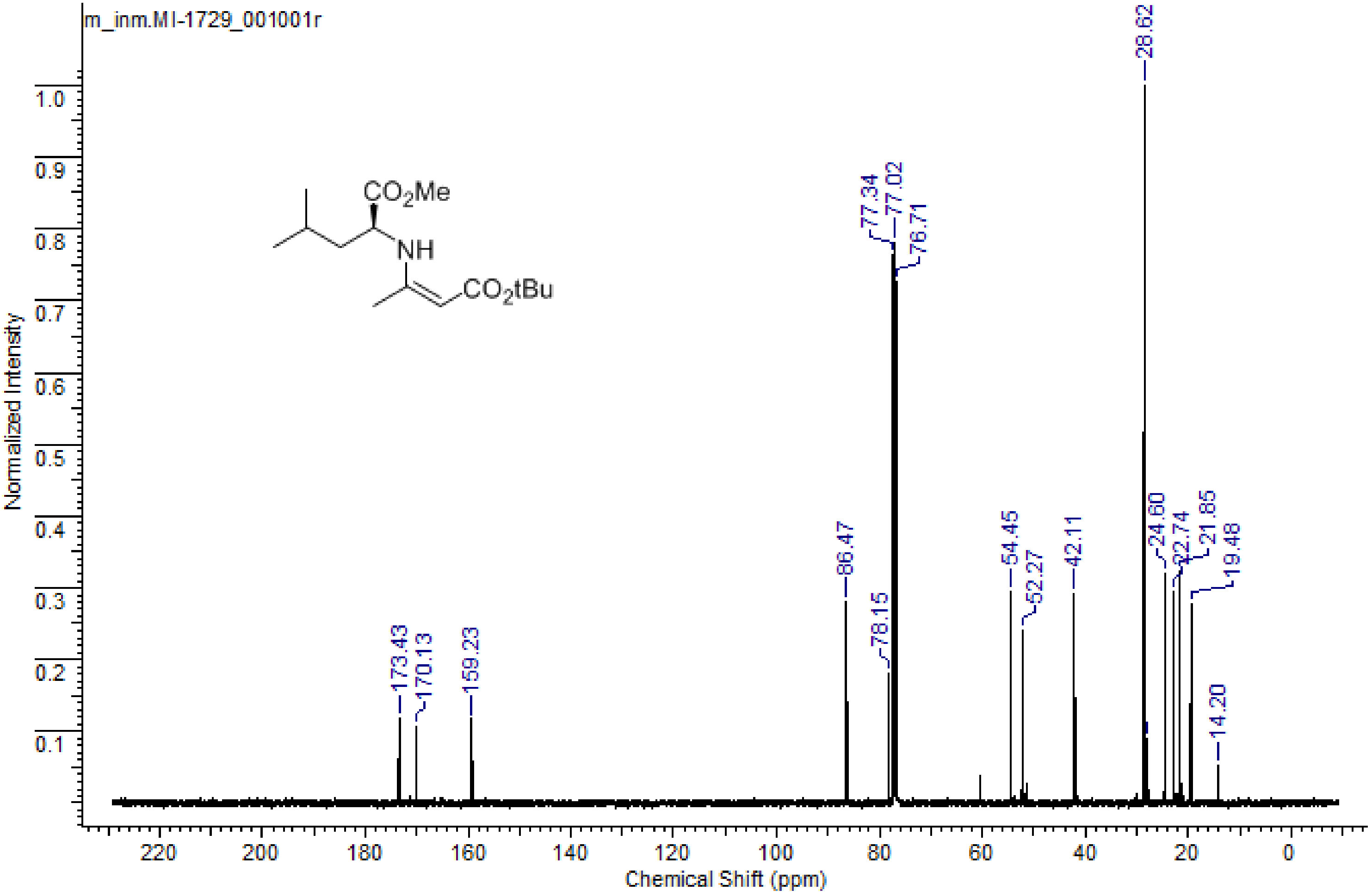

(±)-Menthyl 3-(2-(1H-indol-3-yl)ethylamino)but-2-enoate (7o)

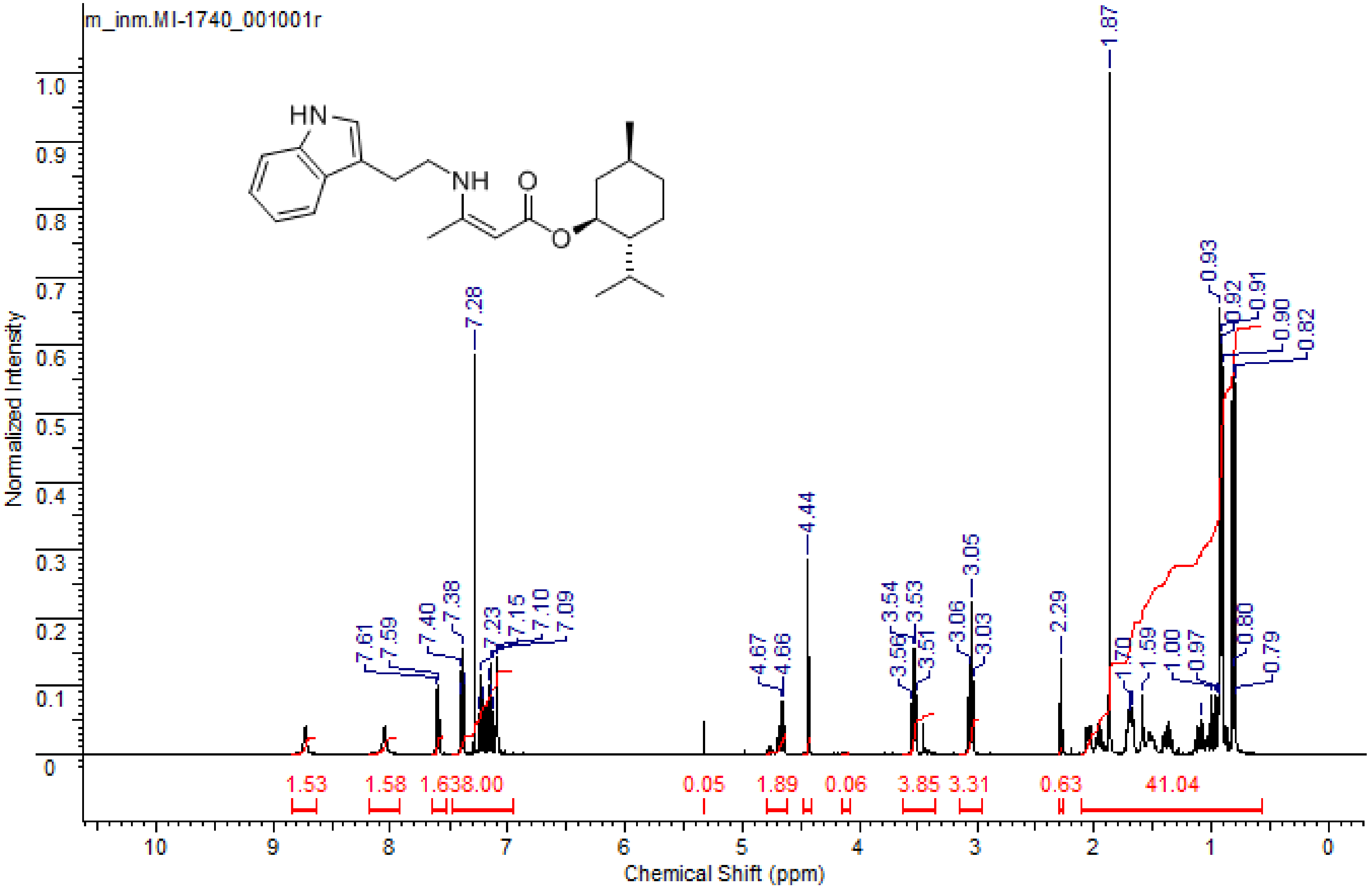

(±)-Menthyl 3-(2-(1H-indol-3-yl)ethylamino)but-2-enoate (7o)

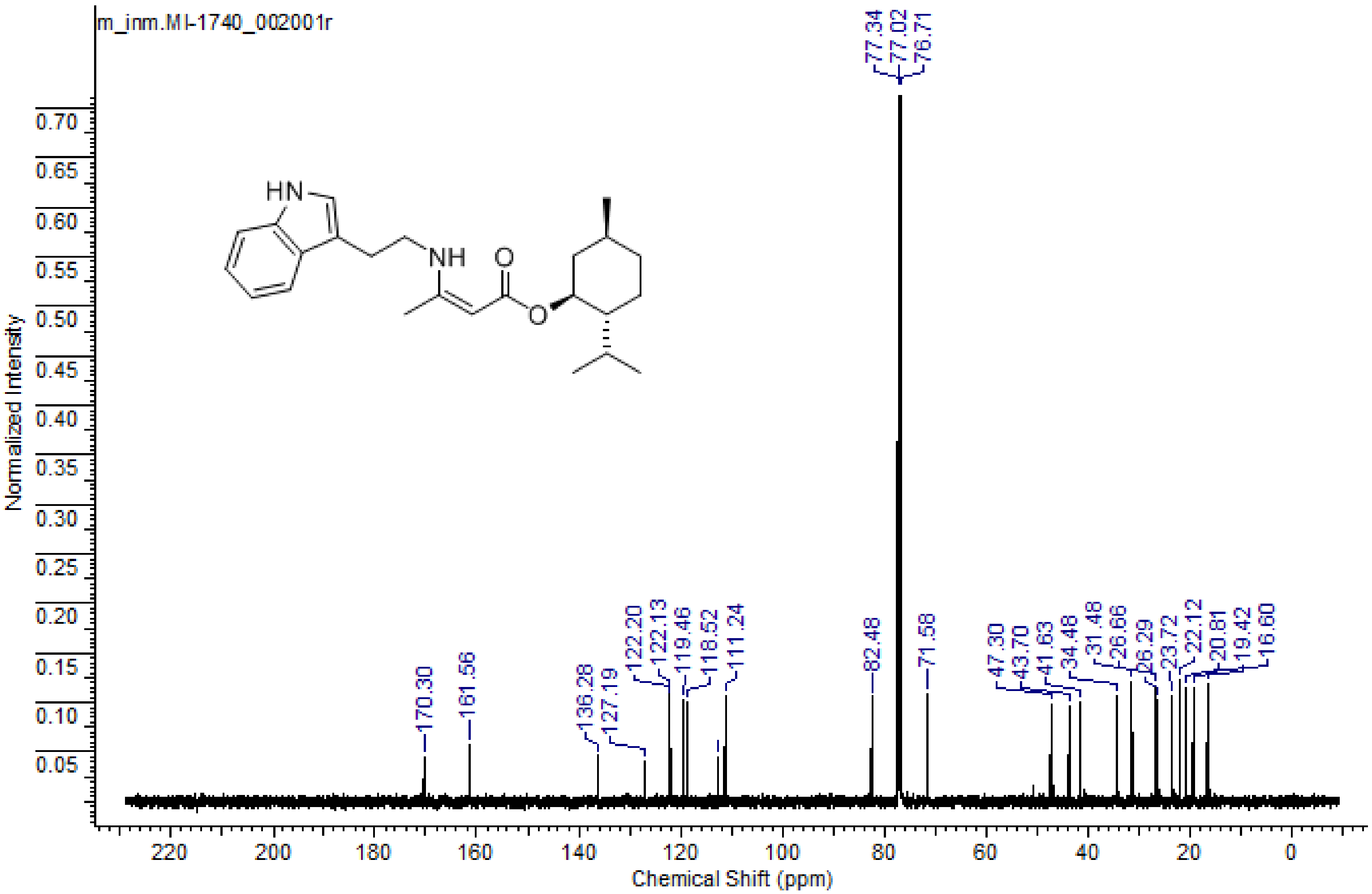

(Z)-3-(4-Methoxybenzylamino)-1-morpholinobut-2-en-1-one (7p)

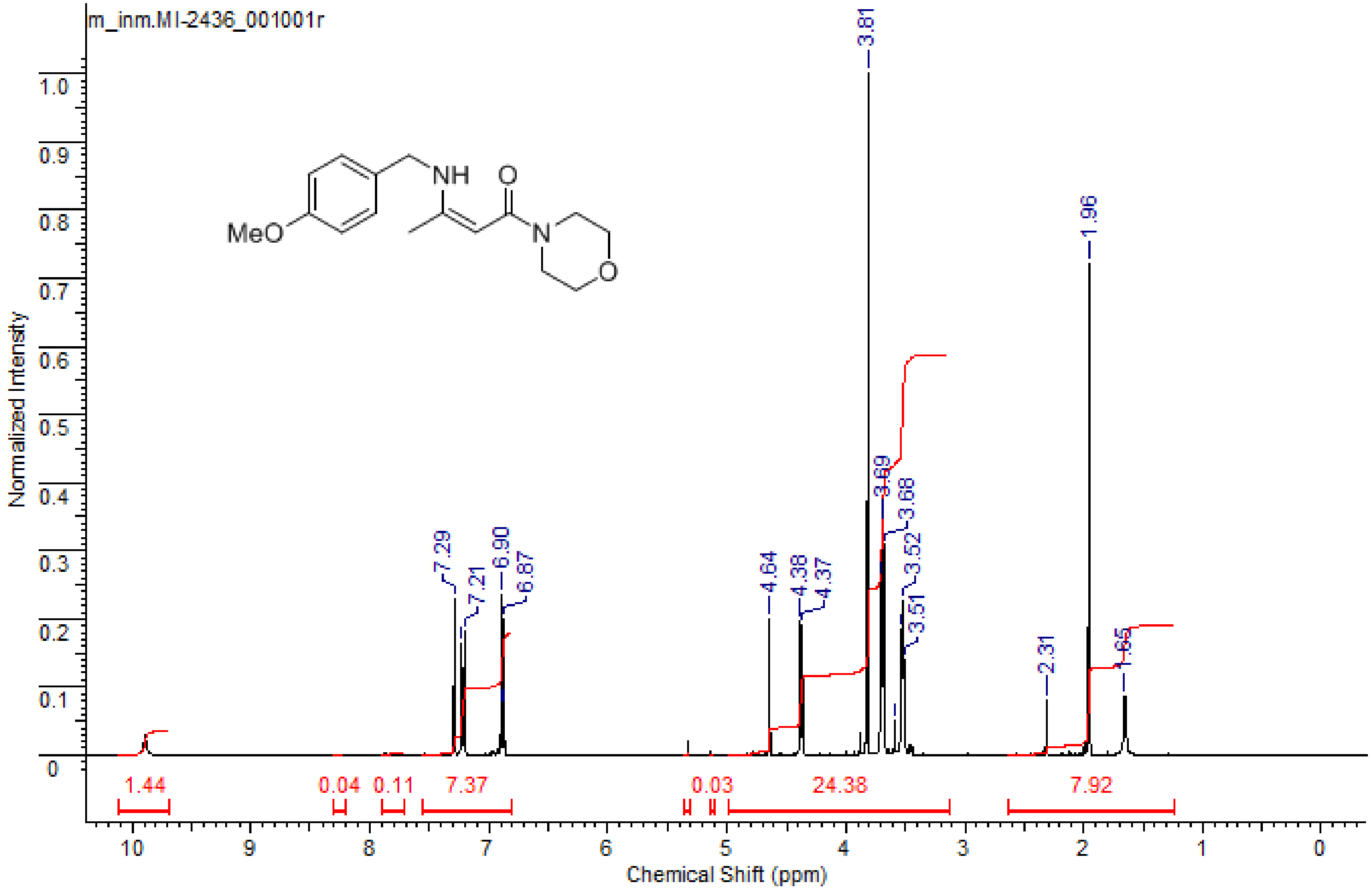

(Z)-3-(4-Methoxybenzylamino)-1-morpholinobut-2-en-1-one (7p)

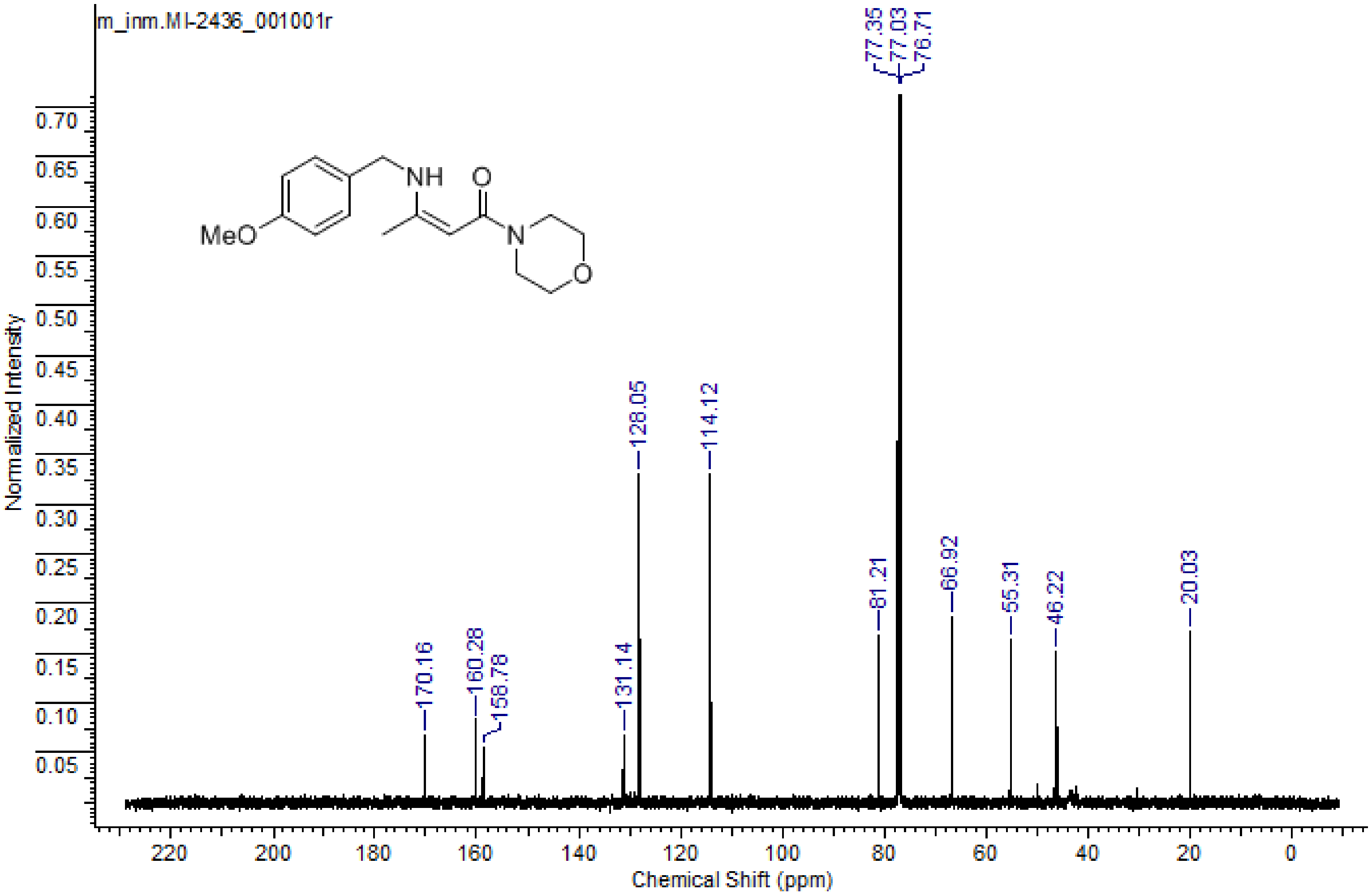

(Z)-Methyl 3-(2,2-diethoxyethylamino)-3-phenylpropenoate (7q)

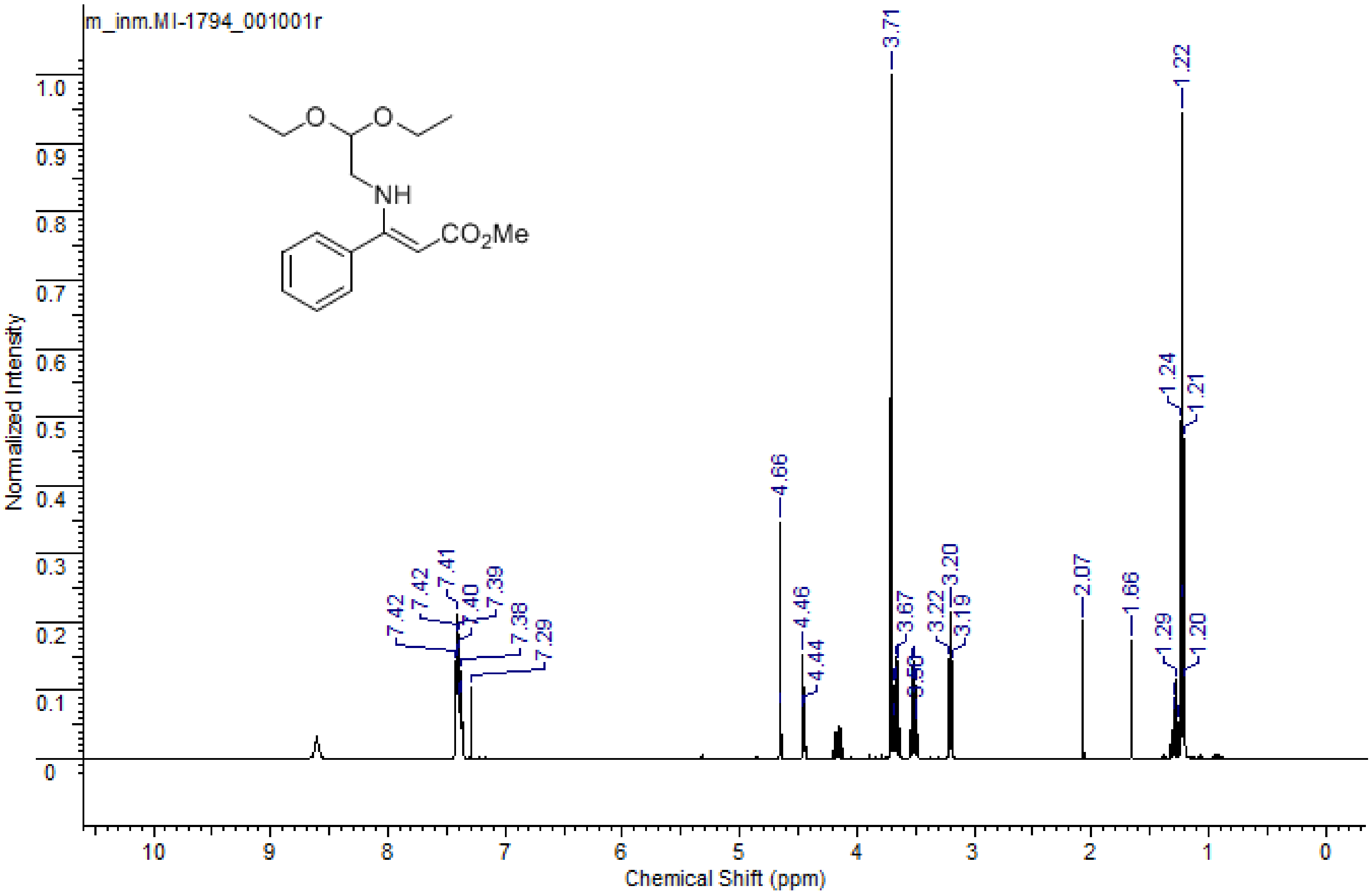

(Z)-Methyl 3-(2,2-diethoxyethylamino)-3-phenylpropenoate (7q)

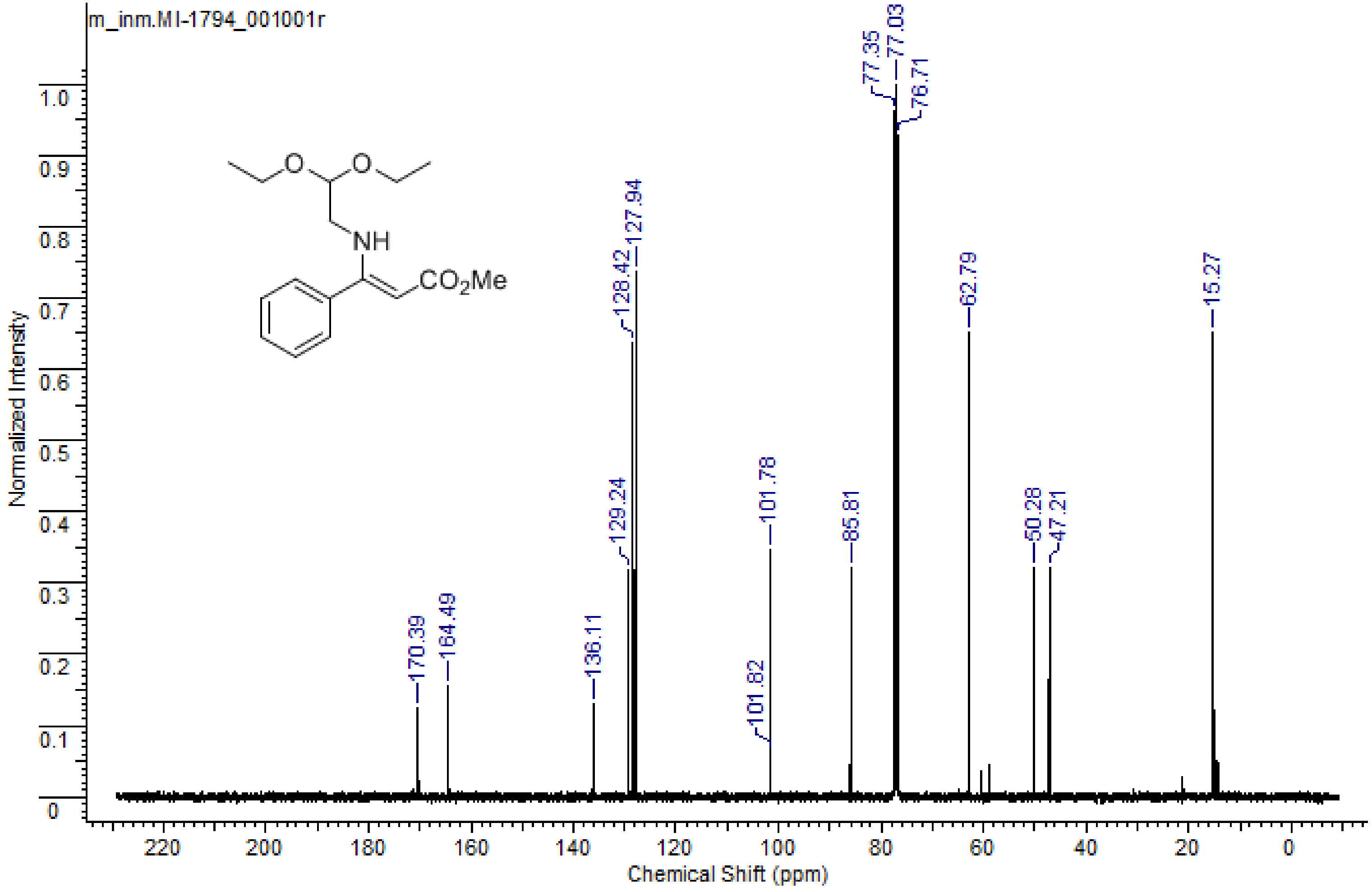

(Z)-Methyl 3-(cyclohexylamino)pent-2-enoate (7s)

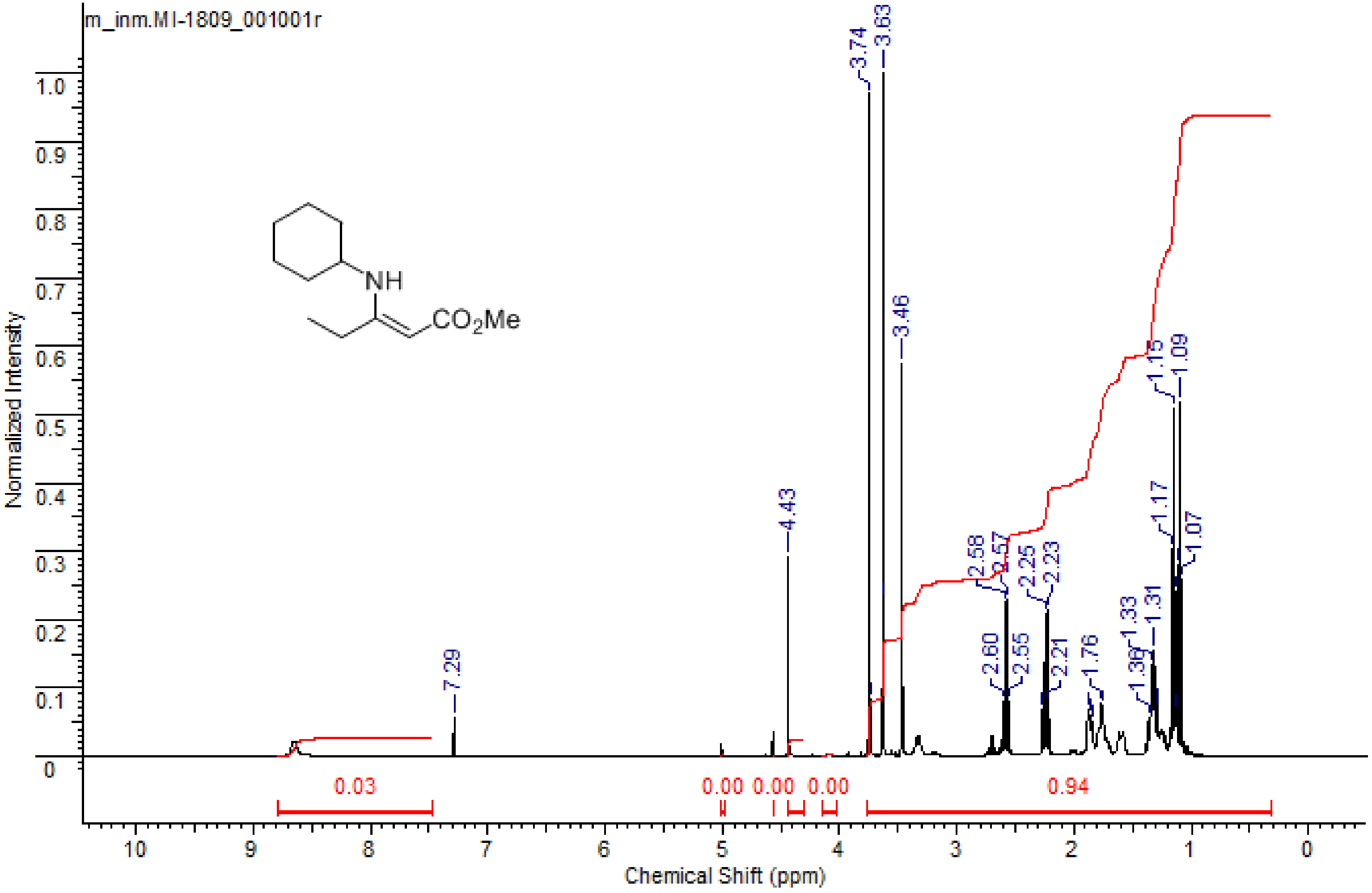

(Z)-Methyl 3-(cyclohexylamino)pent-2-enoate (7s)

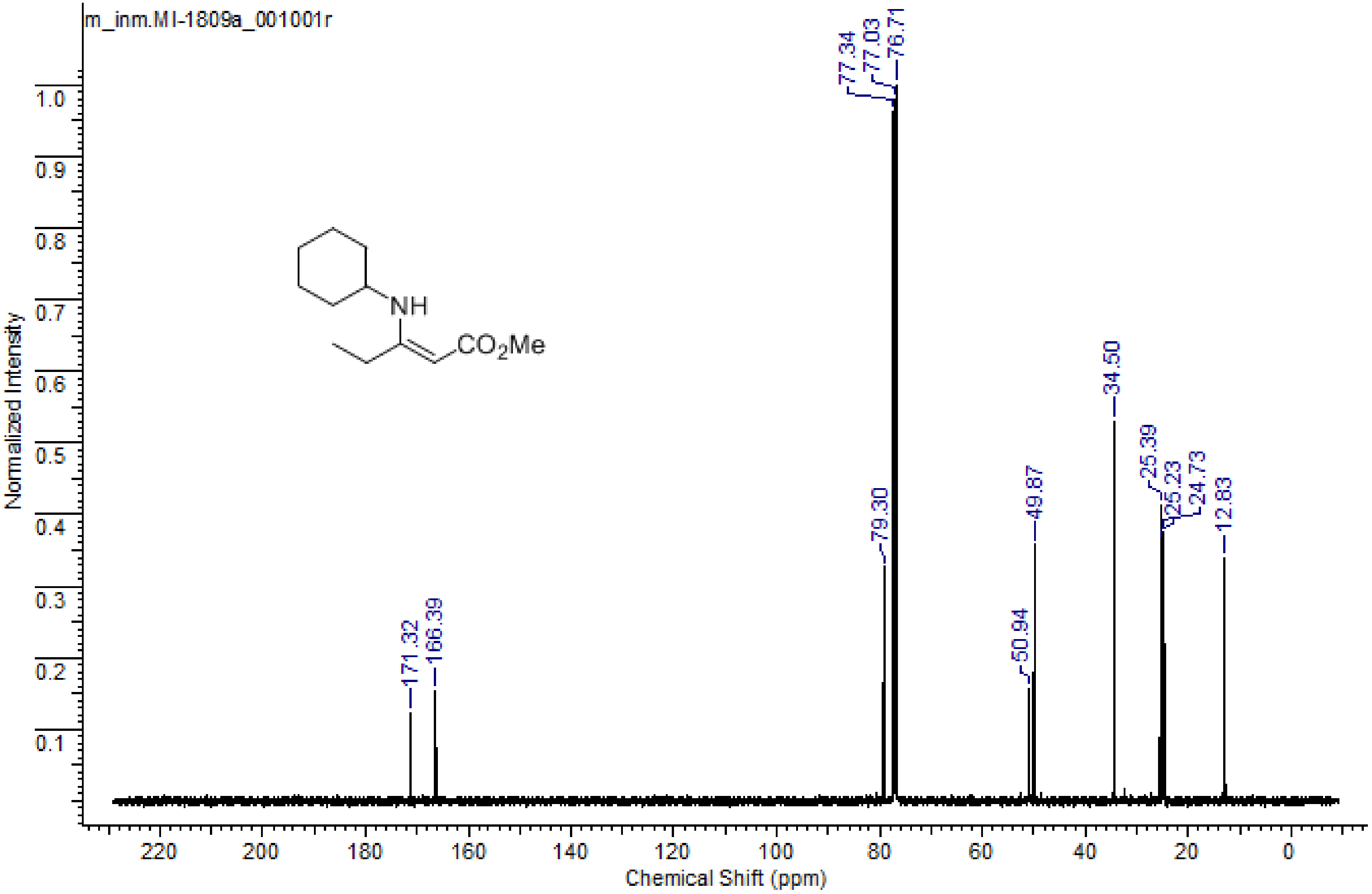

(Z)-Methyl 3-(2-*tert*-butoxy-2-oxoethylamino)but-2-enoate (7t)

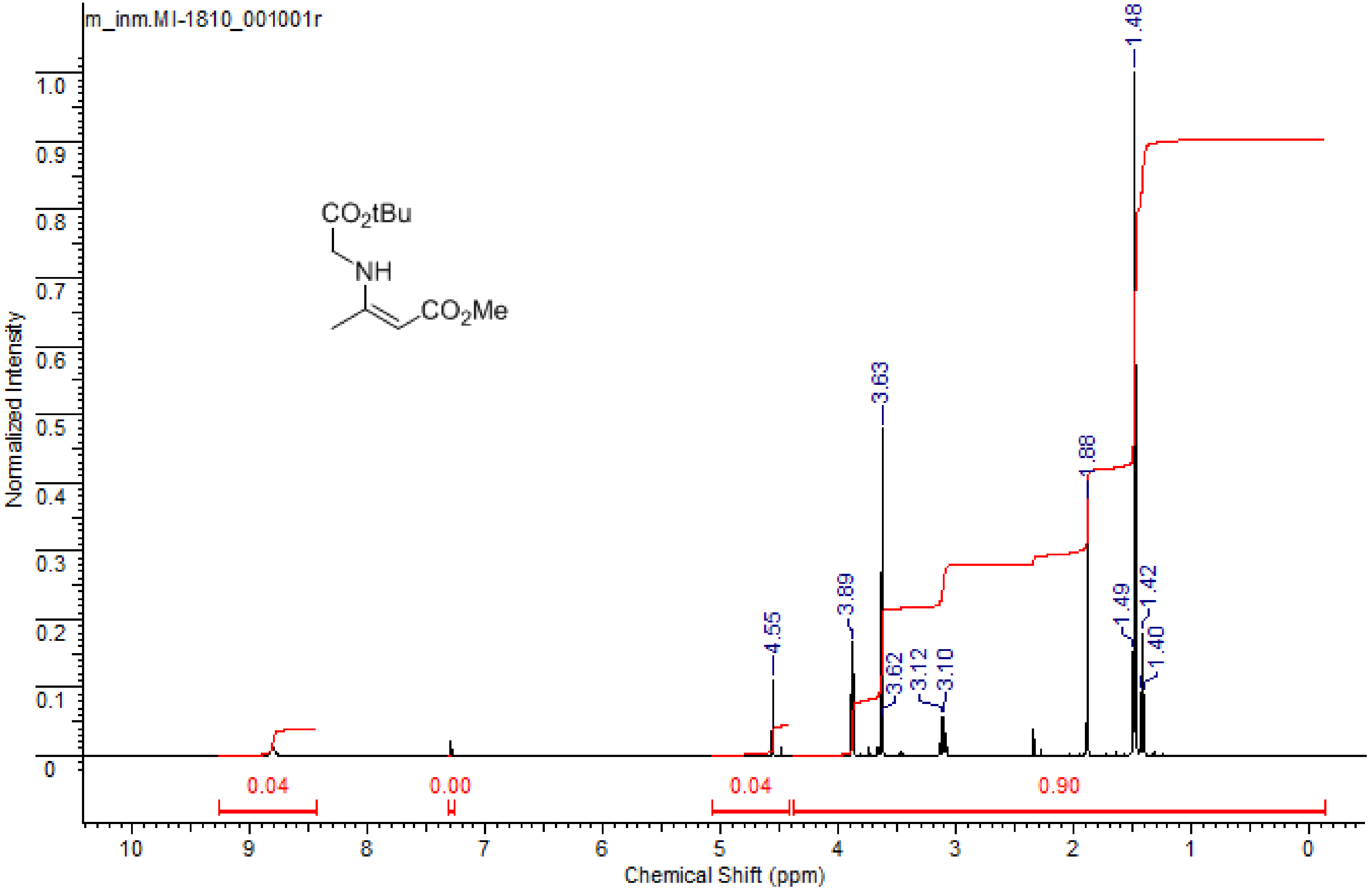

(Z)-Methyl 3-(2-*tert*-butoxy-2-oxoethylamino)but-2-enoate (7t)

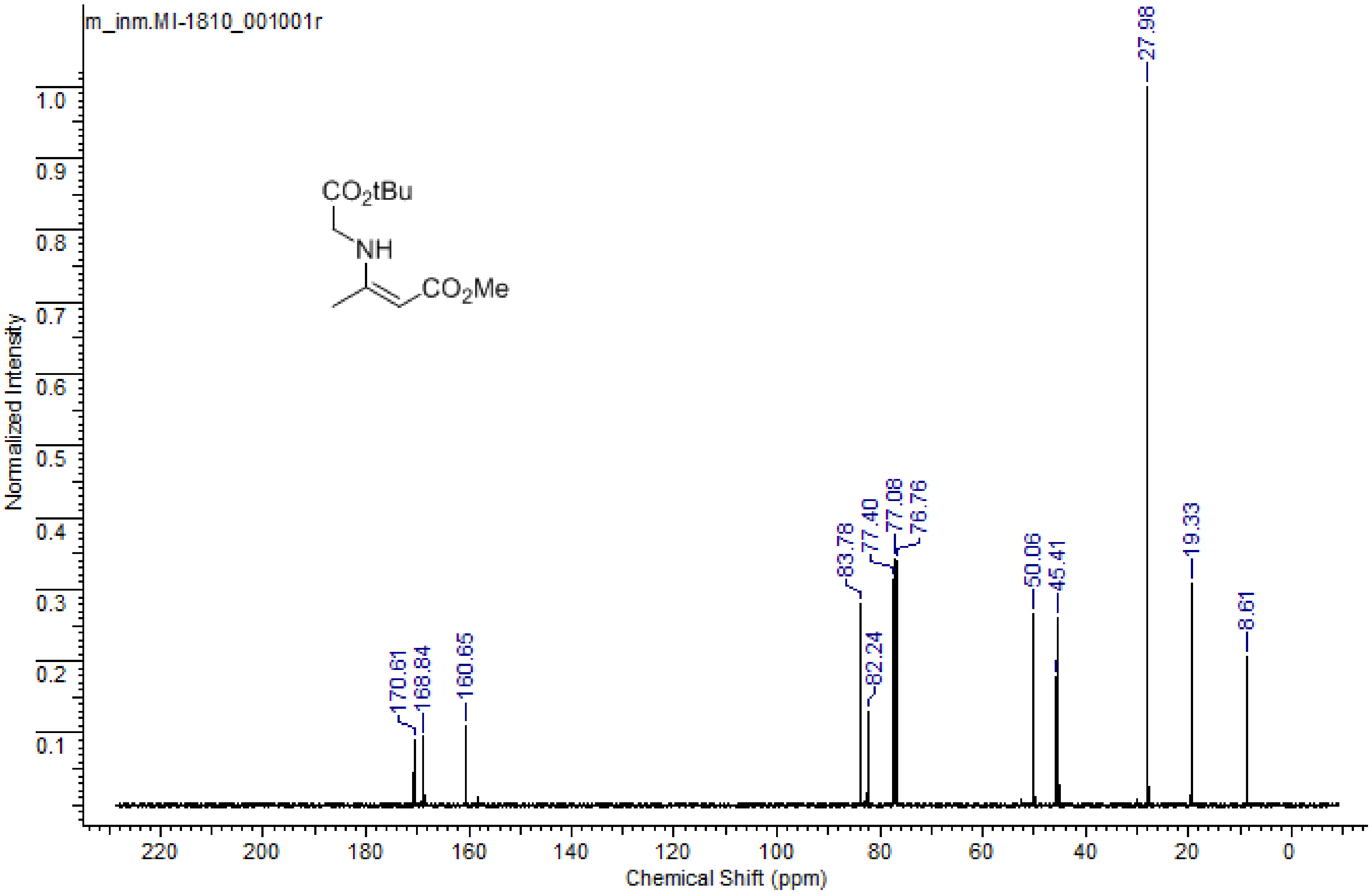

Cholesteryl 3-(2-(dimethylamino)ethylamino)but-2-enoate (7v)

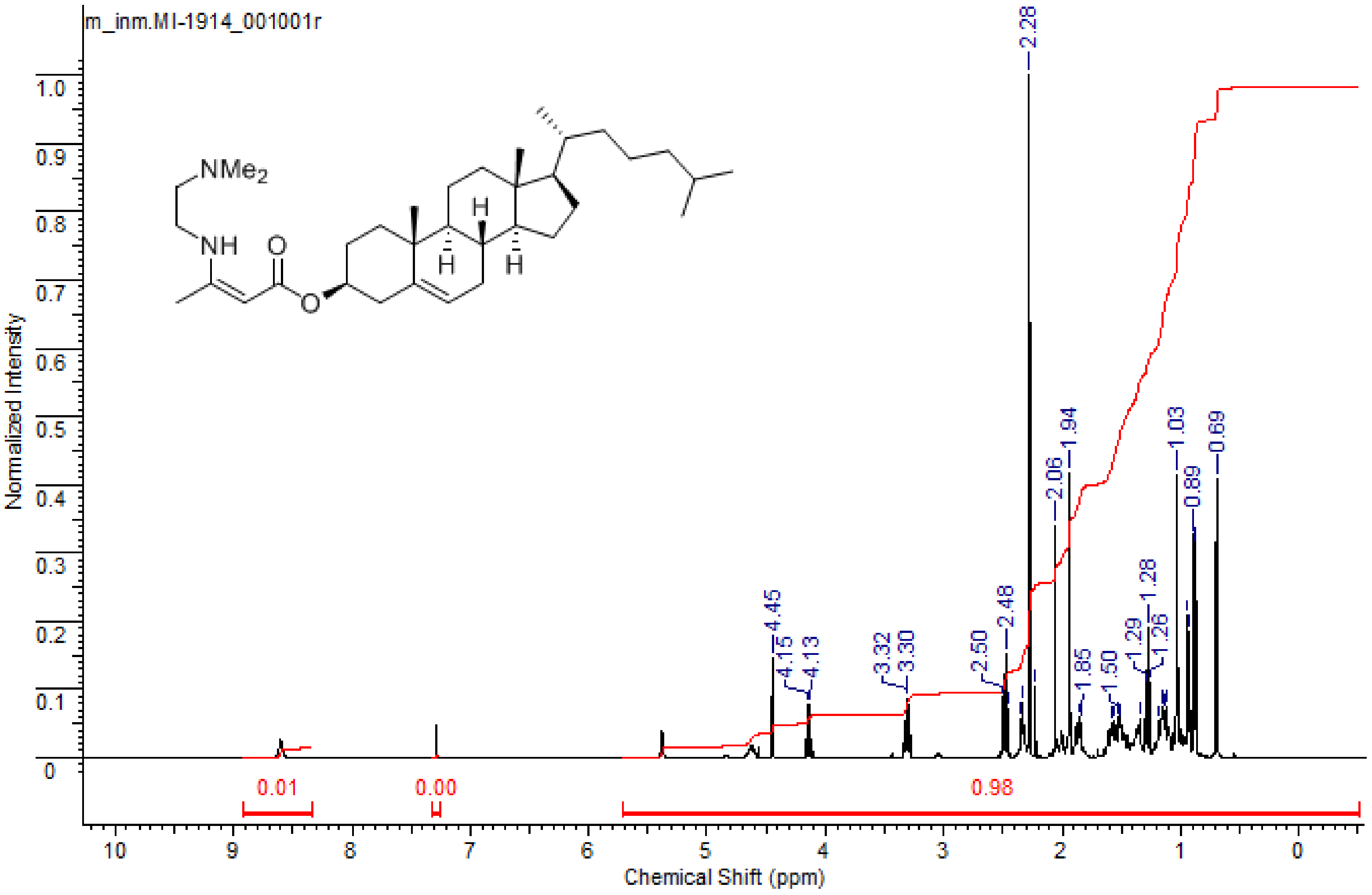

Cholesteryl 3-(2-(dimethylamino)ethylamino)but-2-enoate (7v)

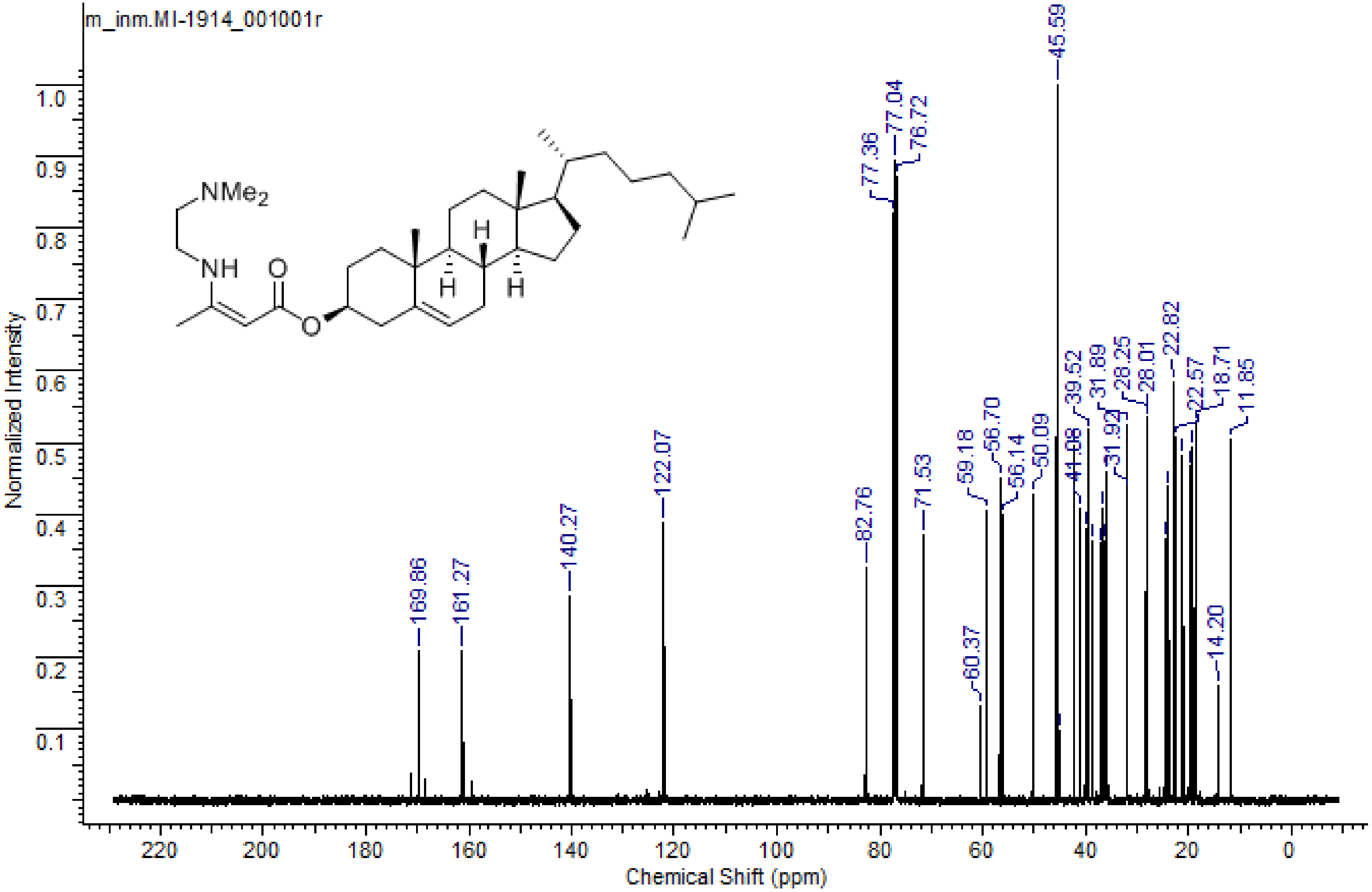

(Z)-Methyl 3-(2-(*tert*-butyldiphenylsilyloxy)ethylamino)but-2-enoate (7w)

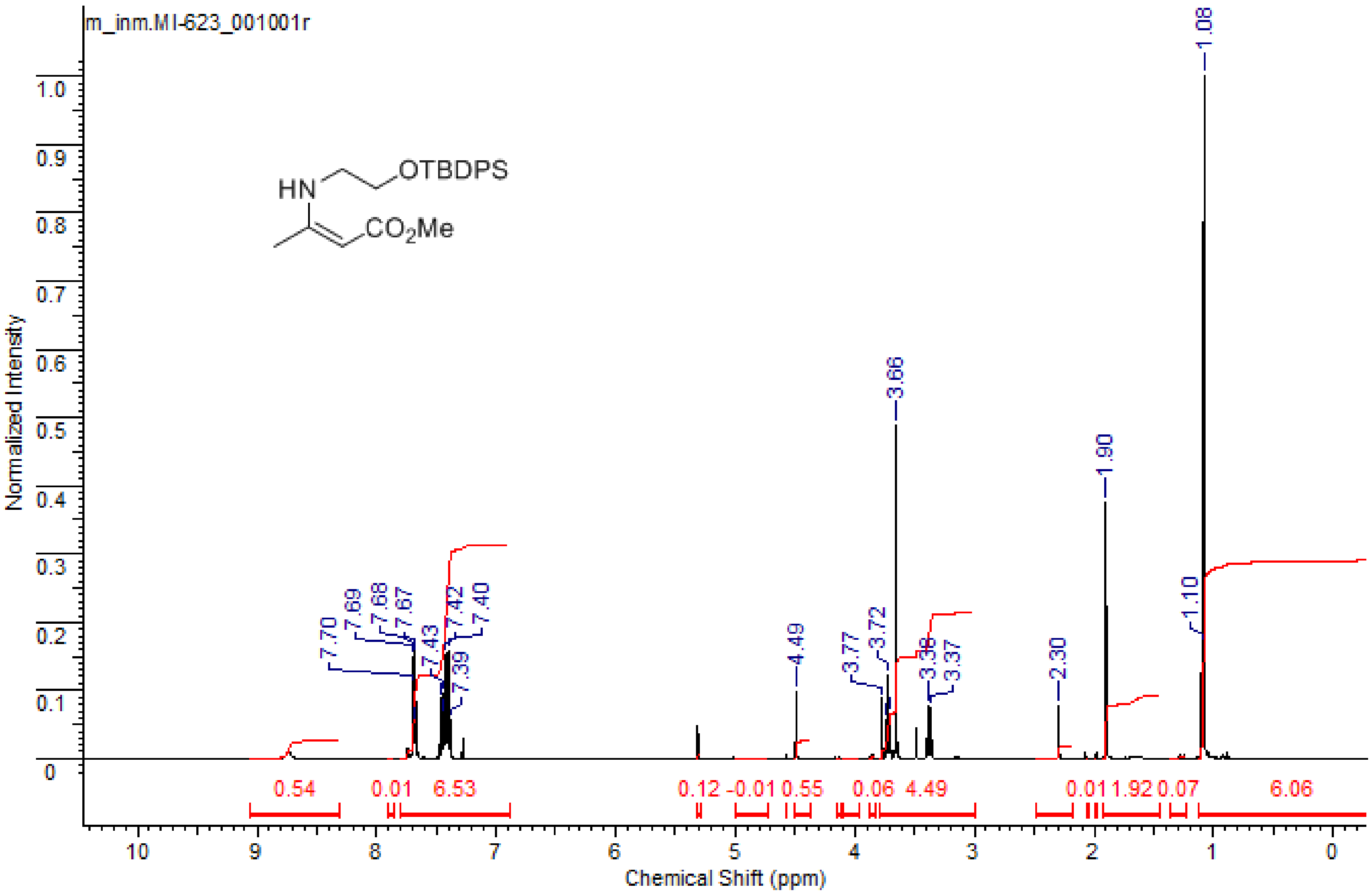

(Z)-Methyl 3-(2-(*tert*-butyldiphenylsilyloxy)ethylamino)but-2-enoate (7w)

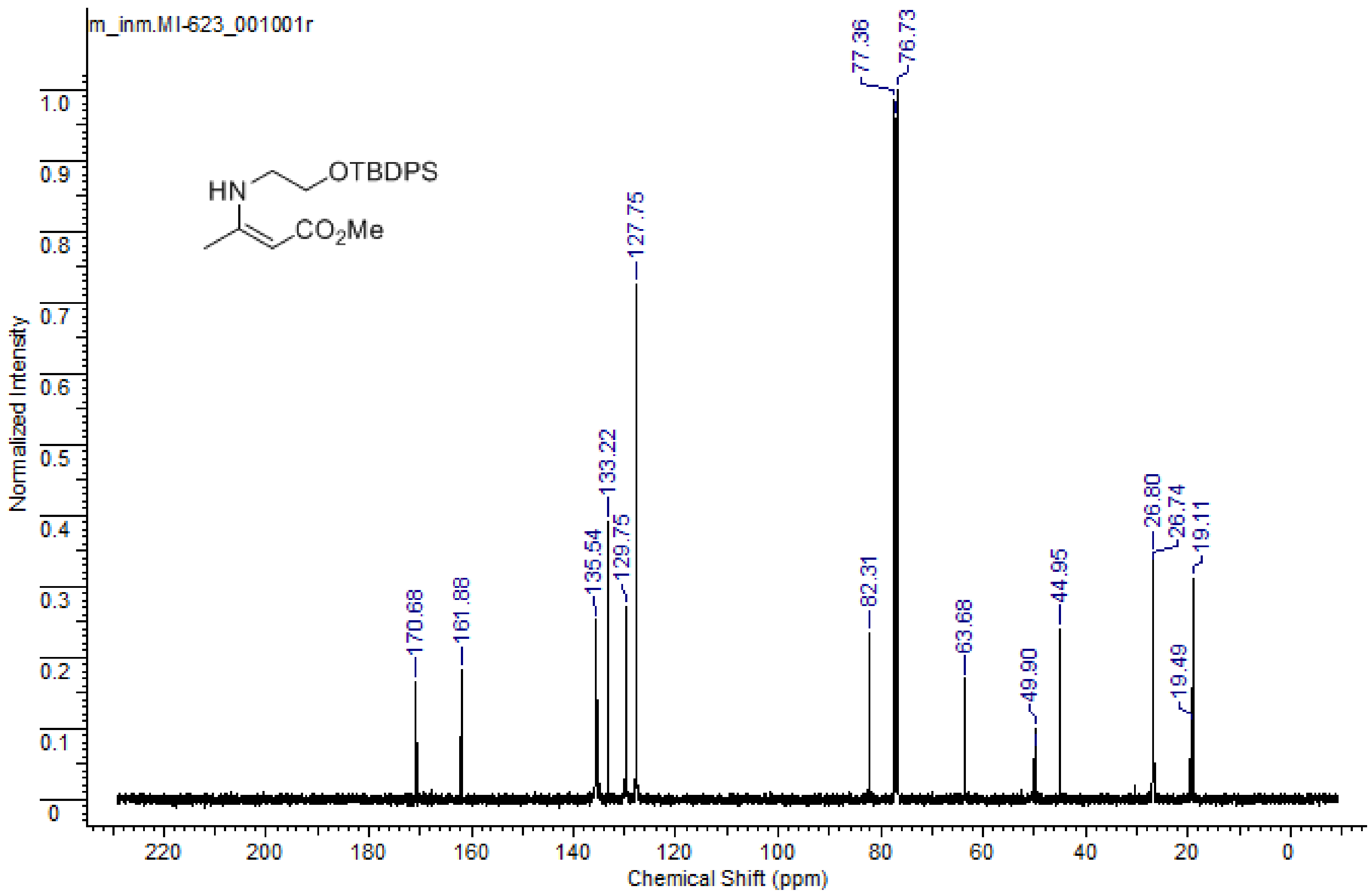

***tert*-Butyl 3-bromo-2-hydroxy-5-methoxyphenyl carbamate**

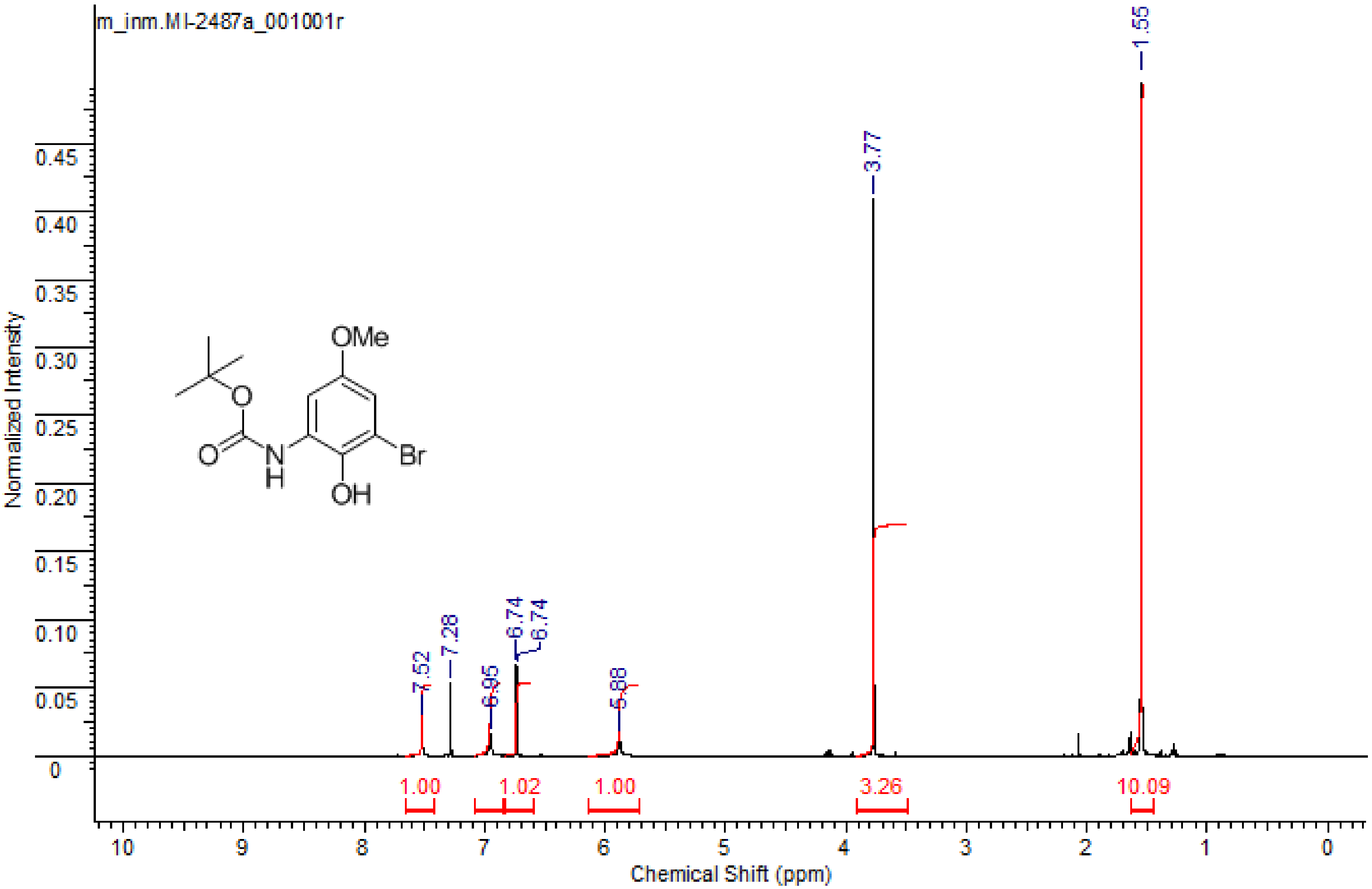

tert-Butyl 3-bromo-2-hydroxy-5-methoxyphenyl carbamate

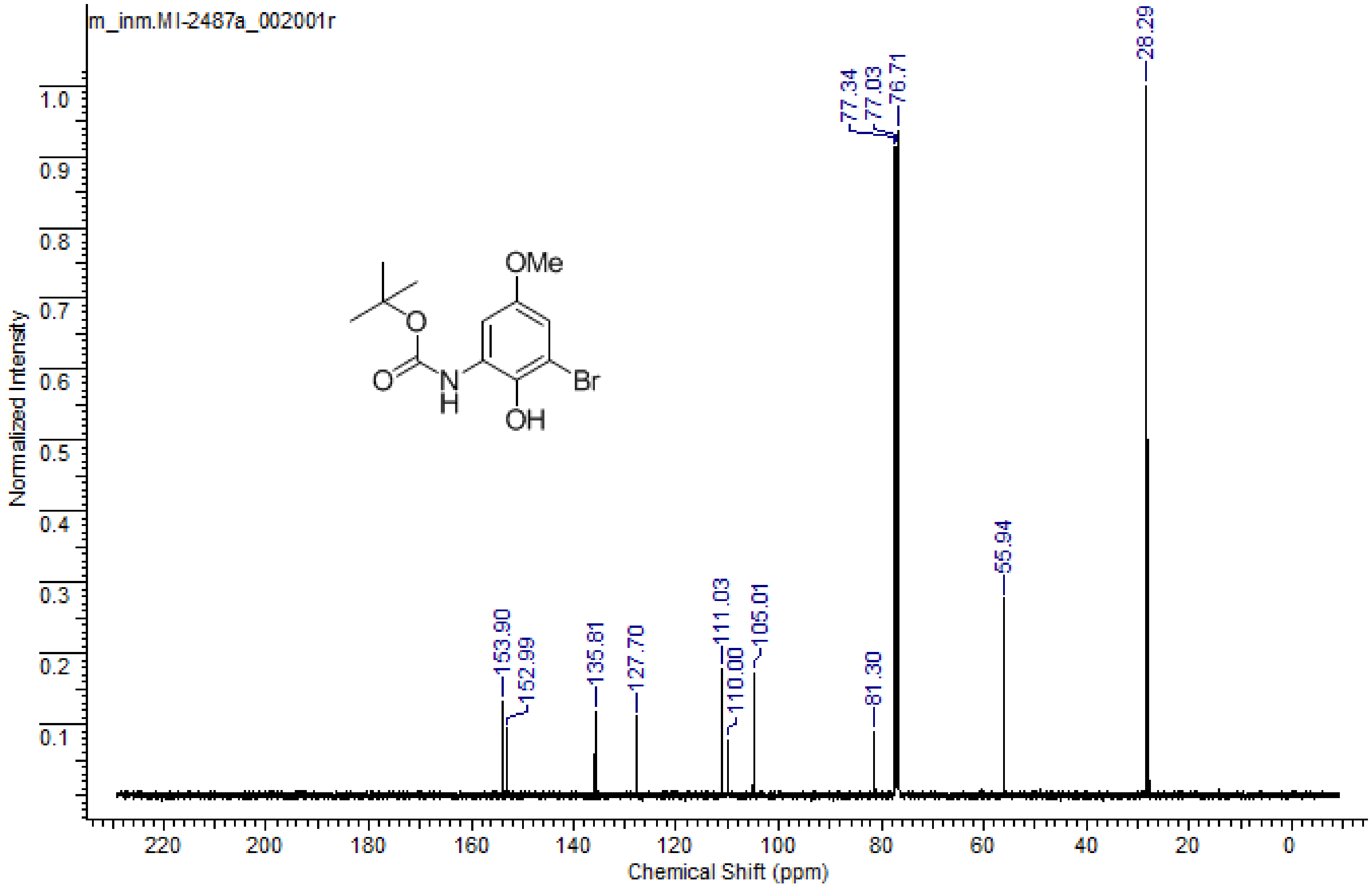

2-Bromo-6-(*tert*-butoxycarbonylamino)-1,4-benzoquinone (6d)

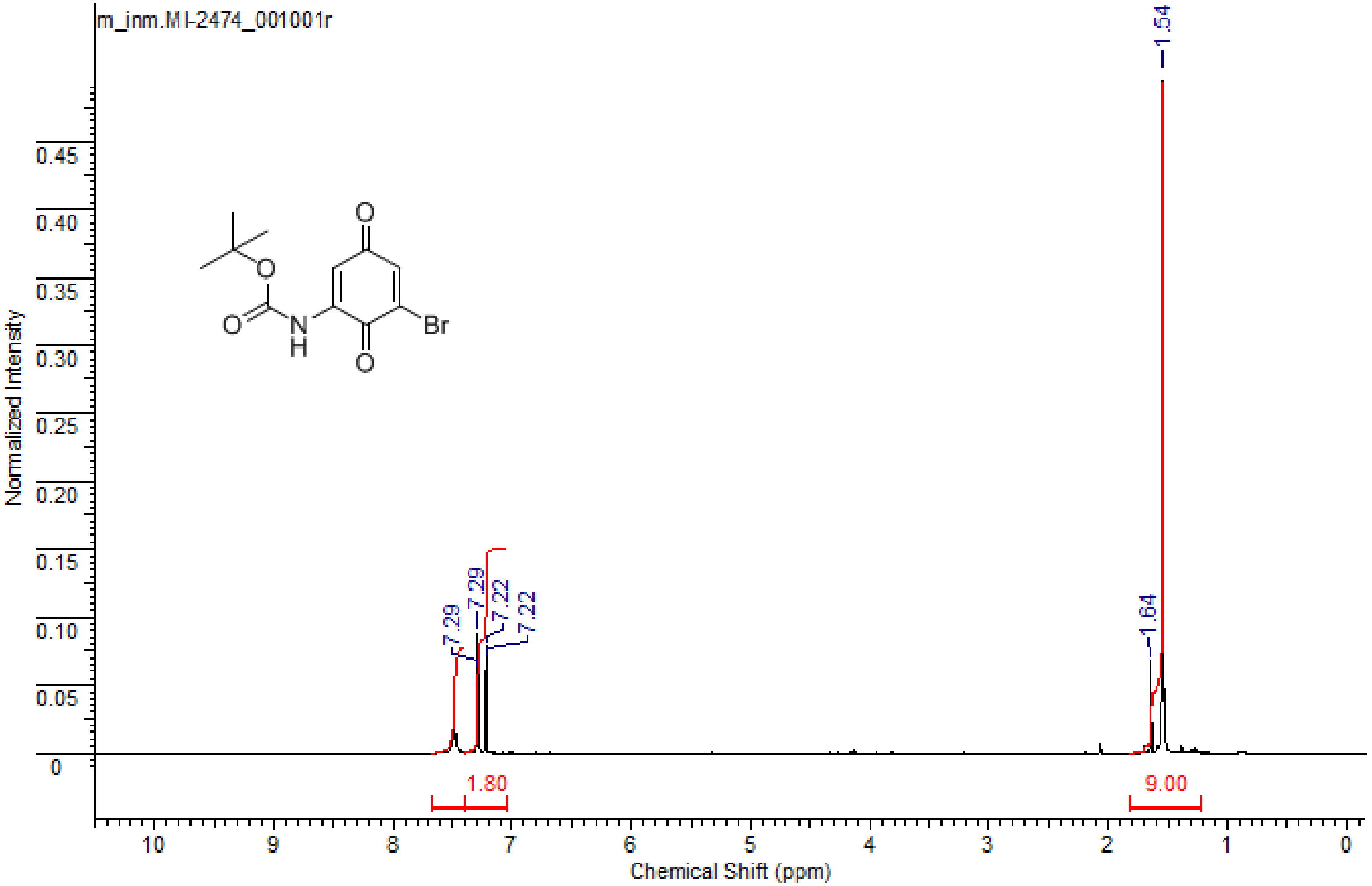

2-Bromo-6-(*tert*-butoxycarbonylamino)-1,4-benzoquinone (6d)

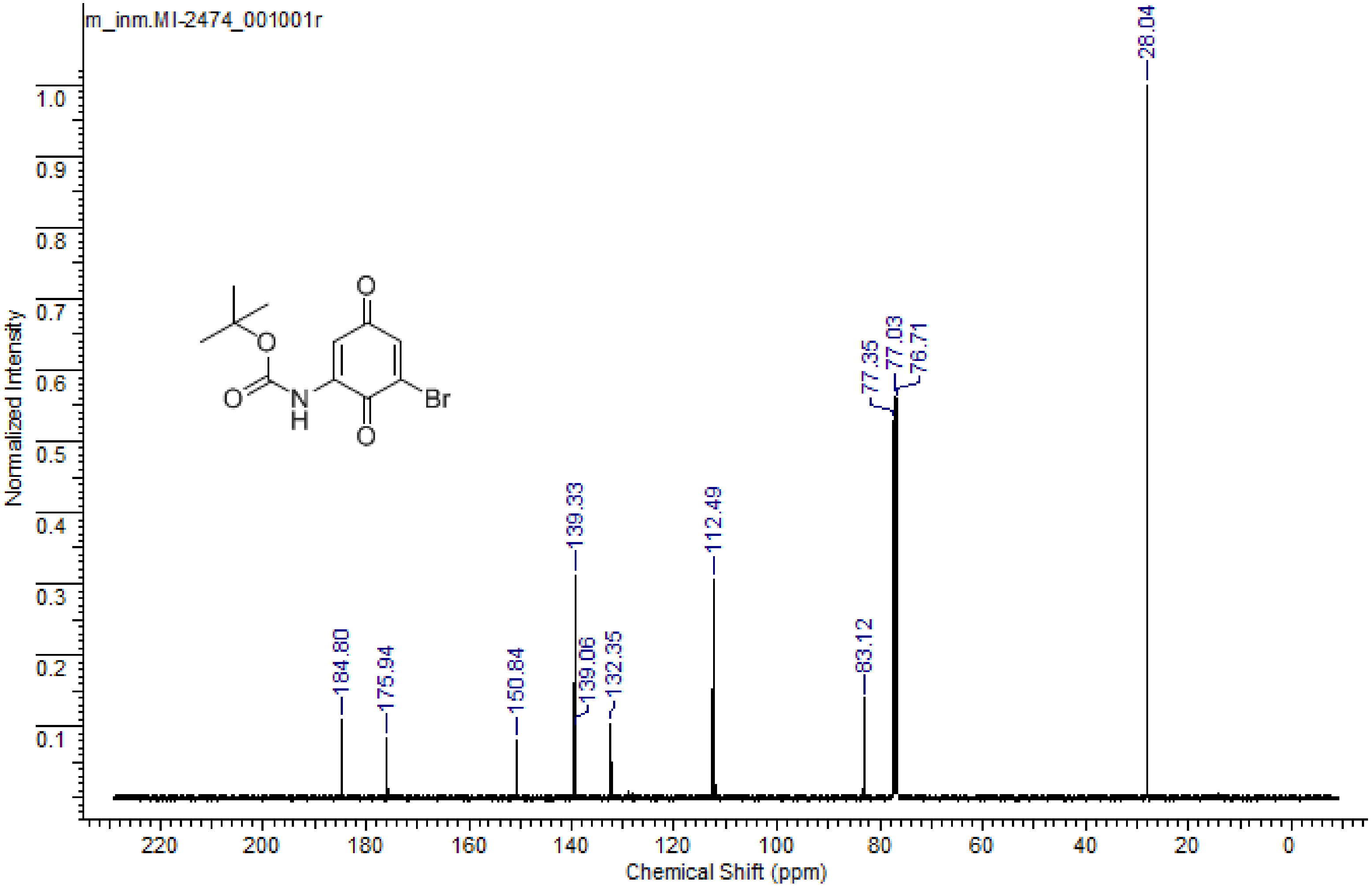

2-Bromo-6-ethyl-1,4-benzoquinone (6f)

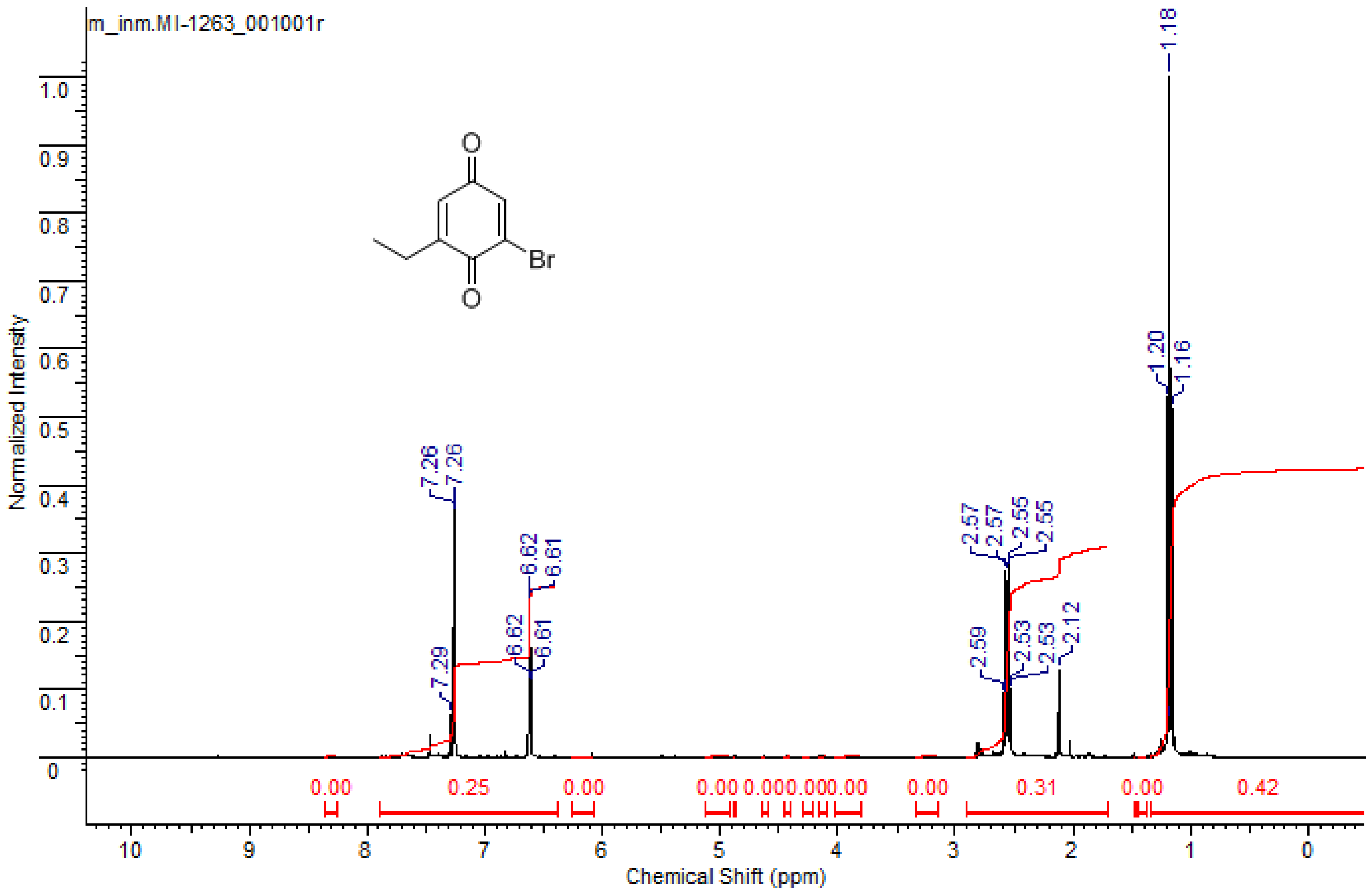

2-Bromo-6-ethyl-1,4-benzoquinone (6f)

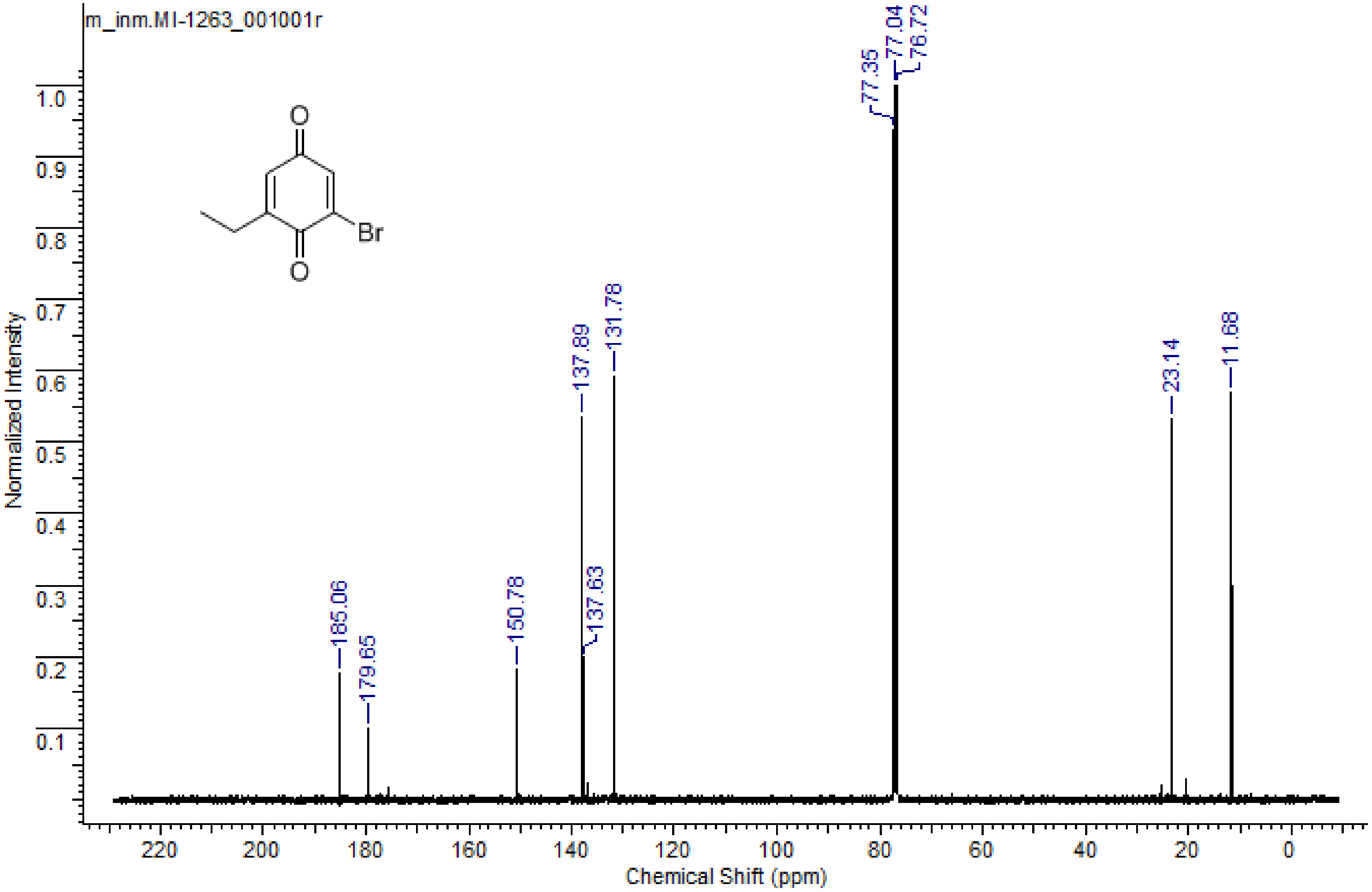

2-Bromo-6-isopropoxy-1,4-benzoquinone (6g)

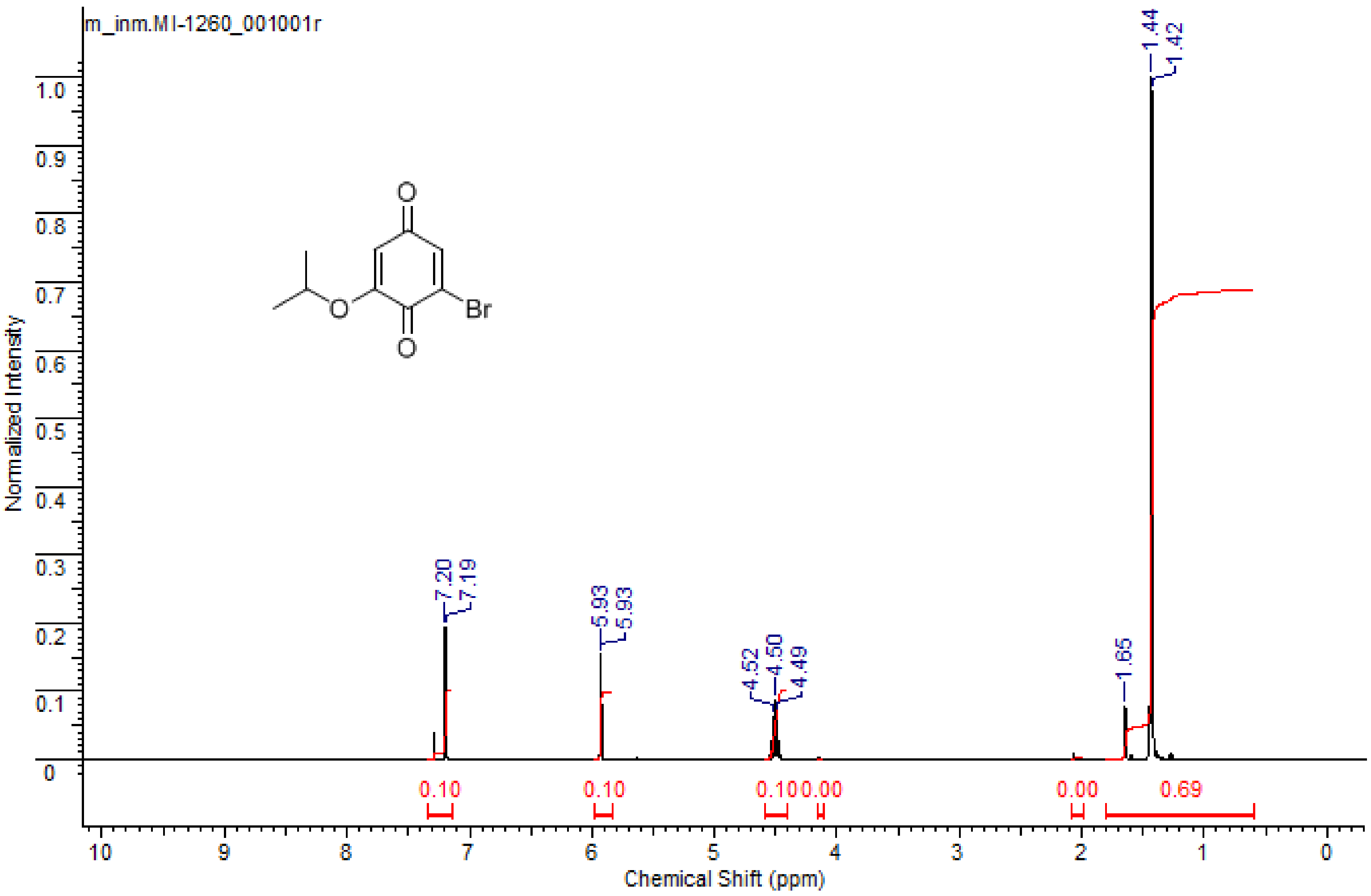

2-Bromo-6-isopropoxy-1,4-benzoquinone (6g)

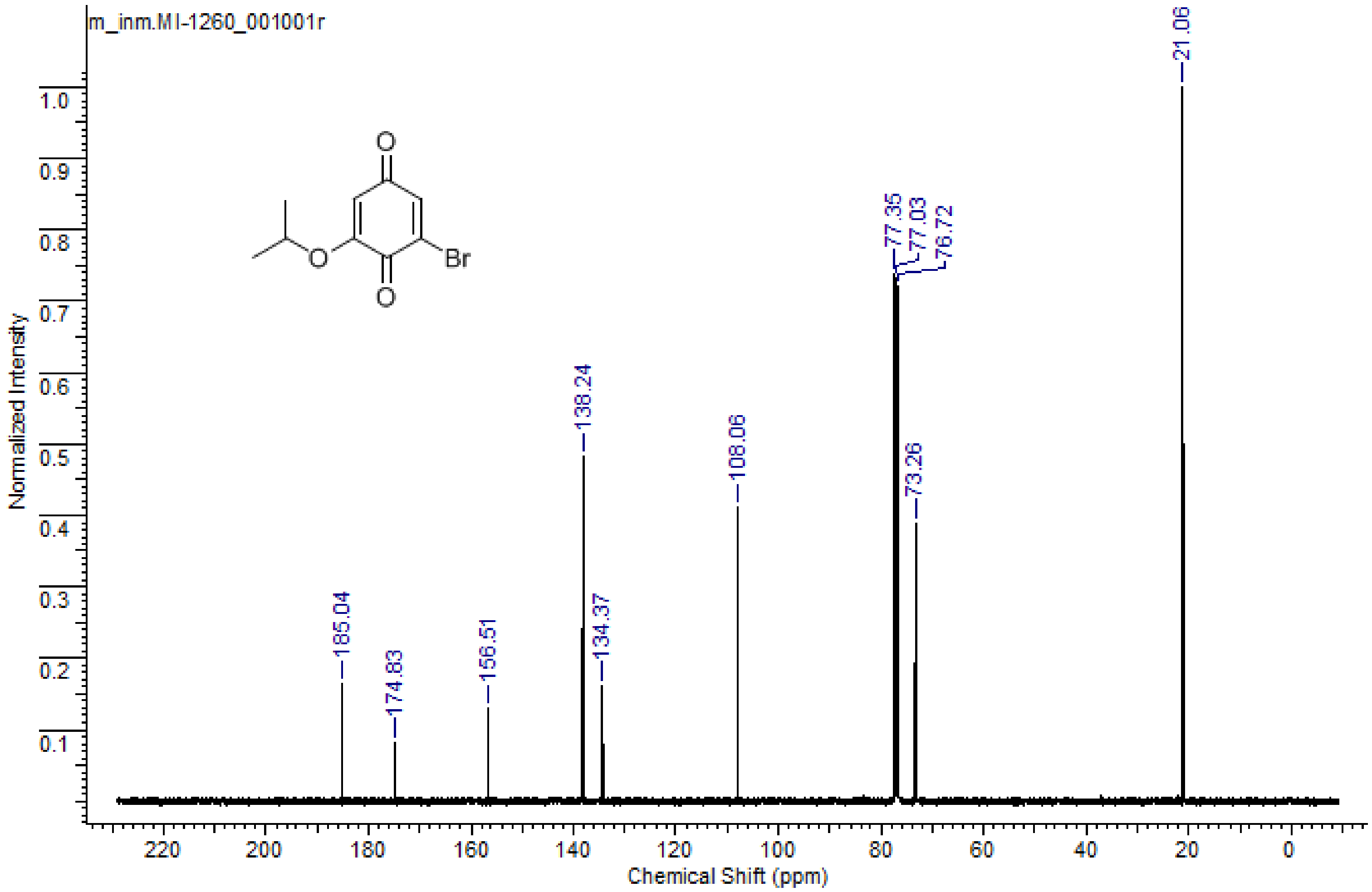

2,3-dihydrobenzo[*b*][1,4]dioxine-5-carbaldehyde

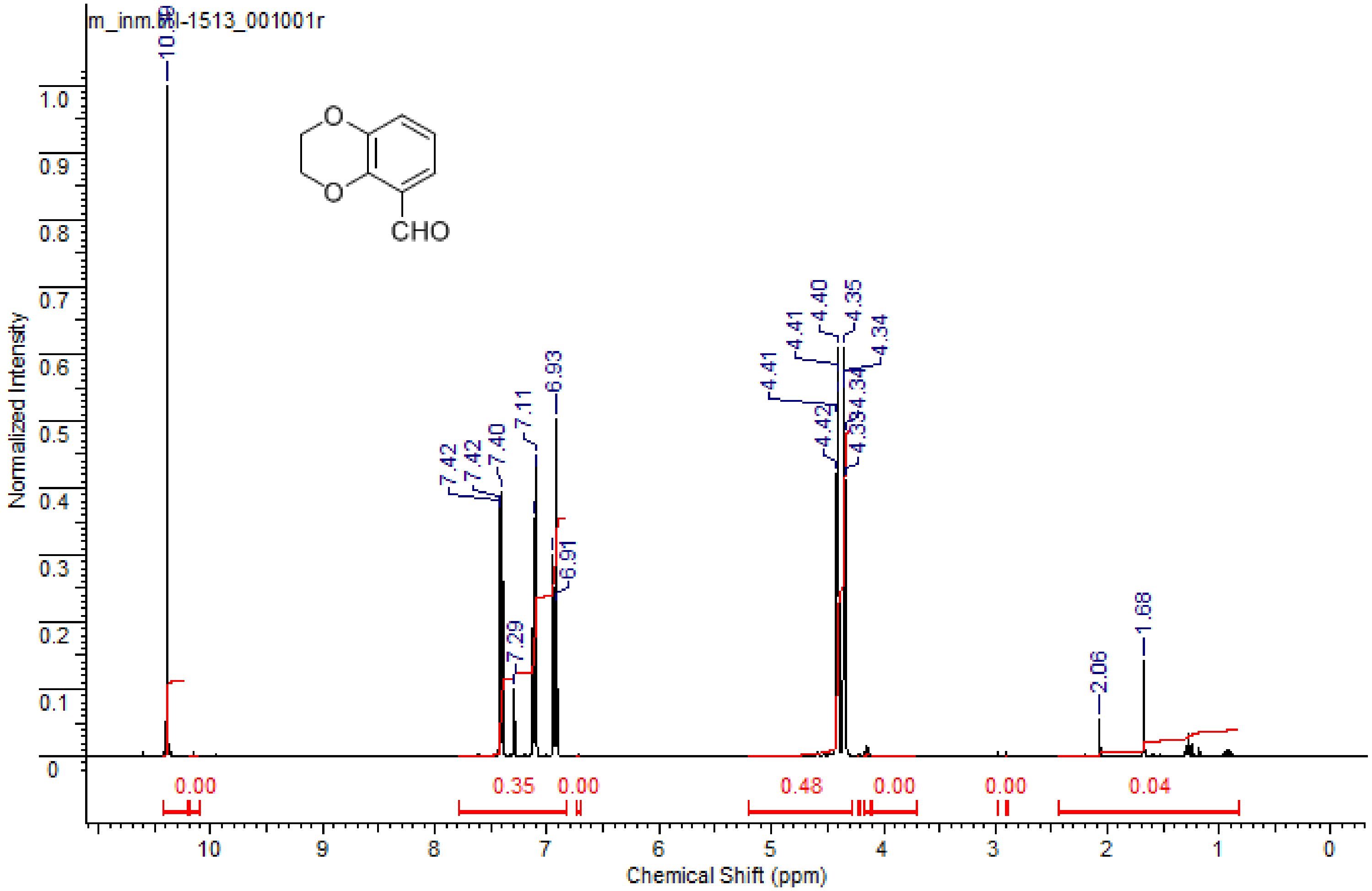

2,3-dihydrobenzo[*b*][1,4]dioxine-5-carbaldehyde

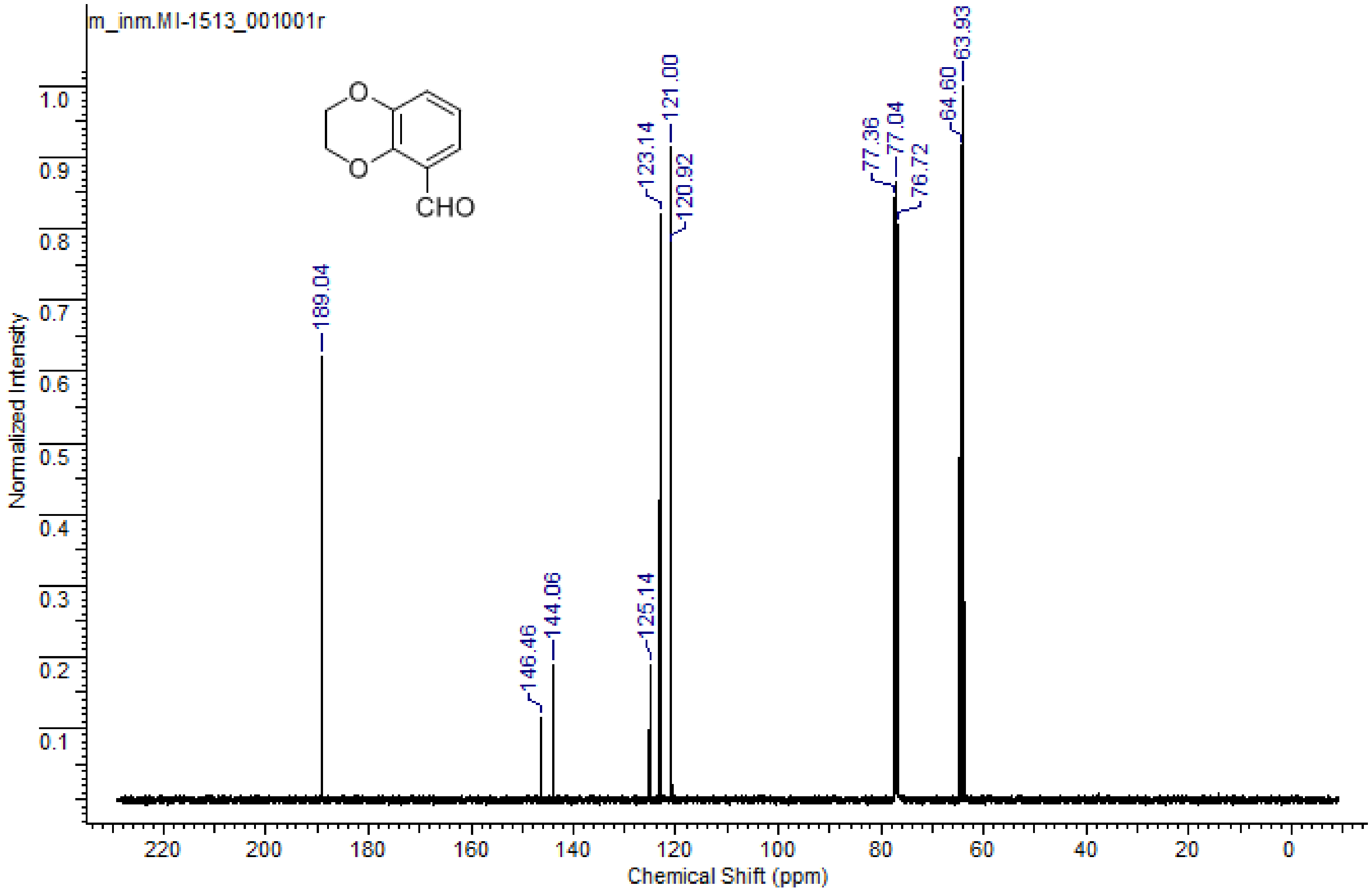

2,3-dihydrobenzo[*b*][1,4]dioxin-5-ol

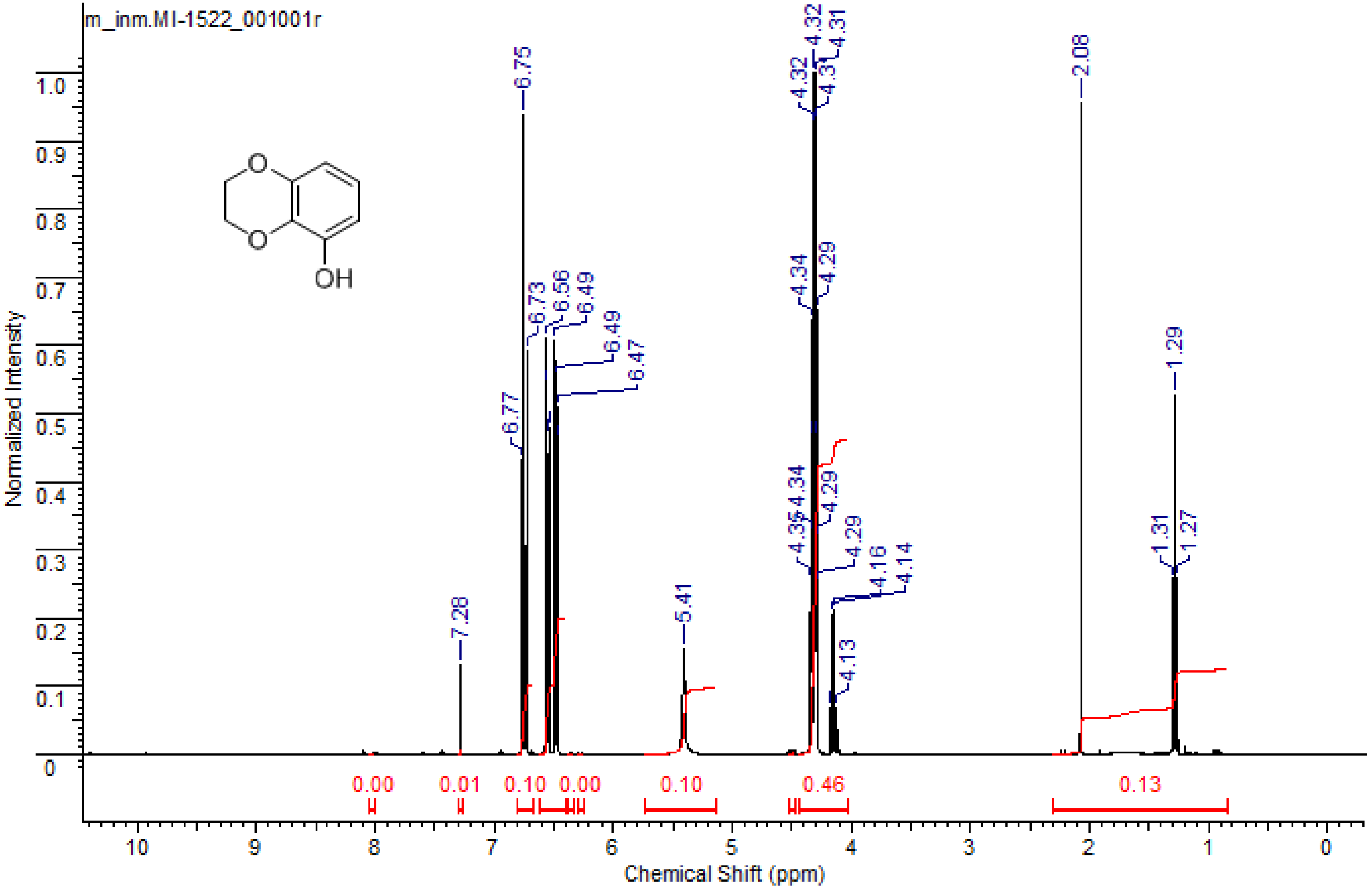

2,3-dihydrobenzo[*b*][1,4]dioxin-5-ol

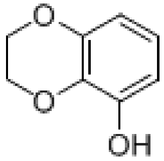

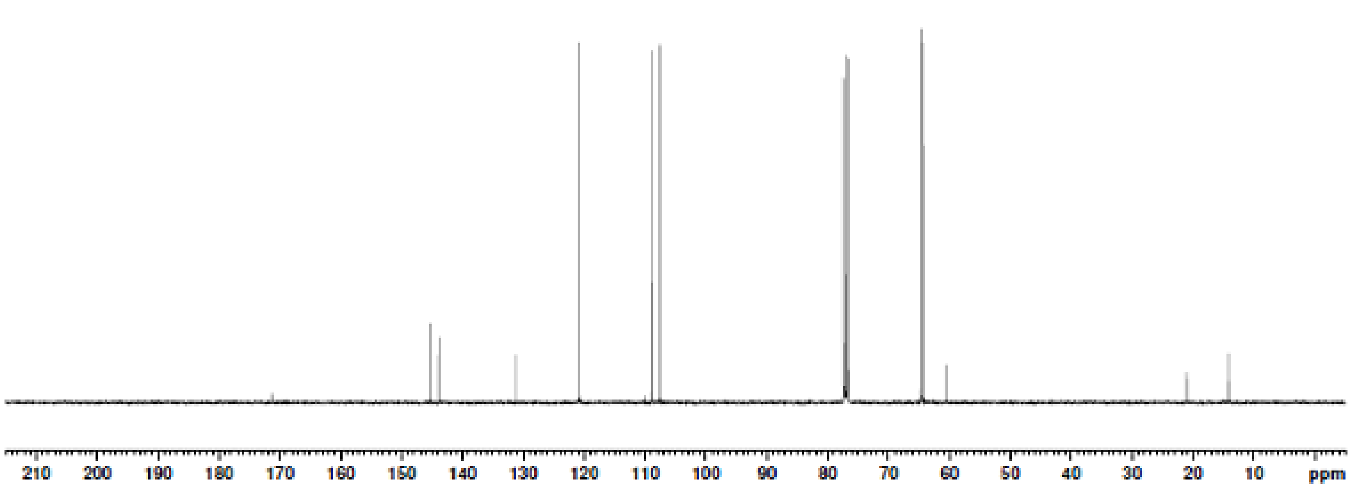

6,8-dibromo-2,3-dihydrobenzo[*b*][1,4]dioxin-5-ol

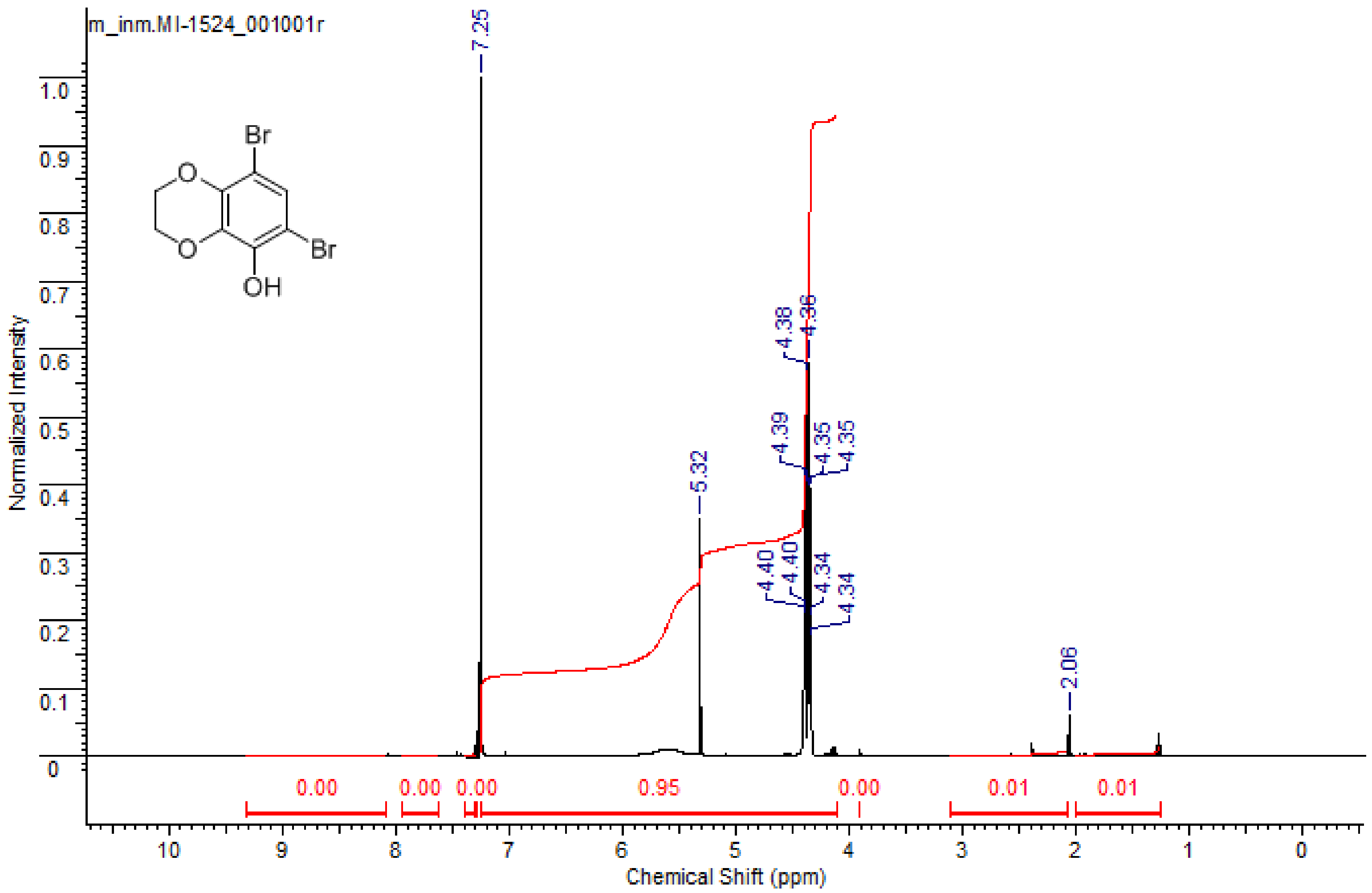

6,8-dibromo-2,3-dihydrobenzo[*b*][1,4]dioxin-5-ol

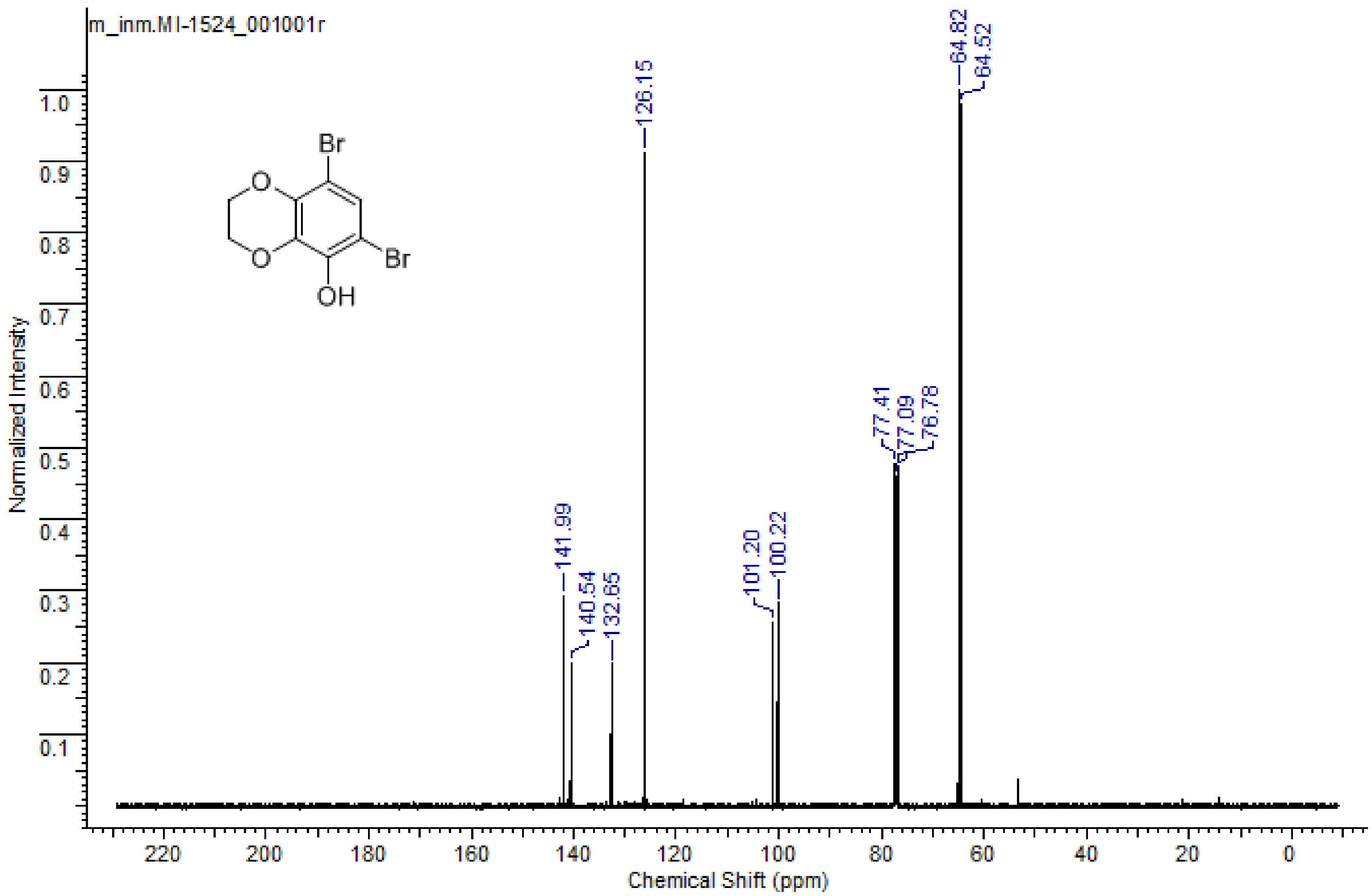

6-bromo-2,3-dihydrobenzo[*b*][1,4]dioxine-5,8-dione (6h)

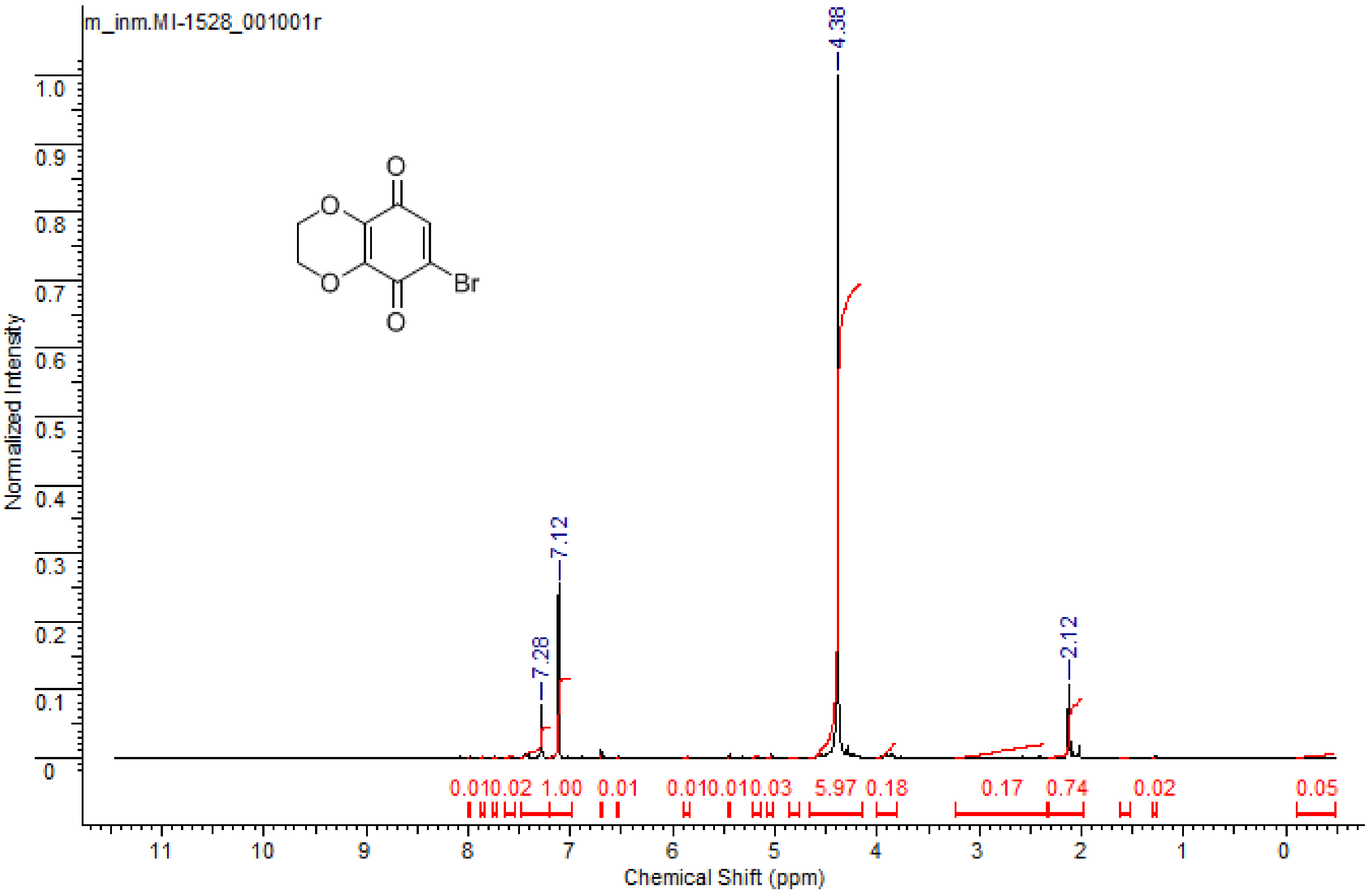

6-bromo-2,3-dihydrobenzo[*b*][1,4]dioxine-5,8-dione (6h)

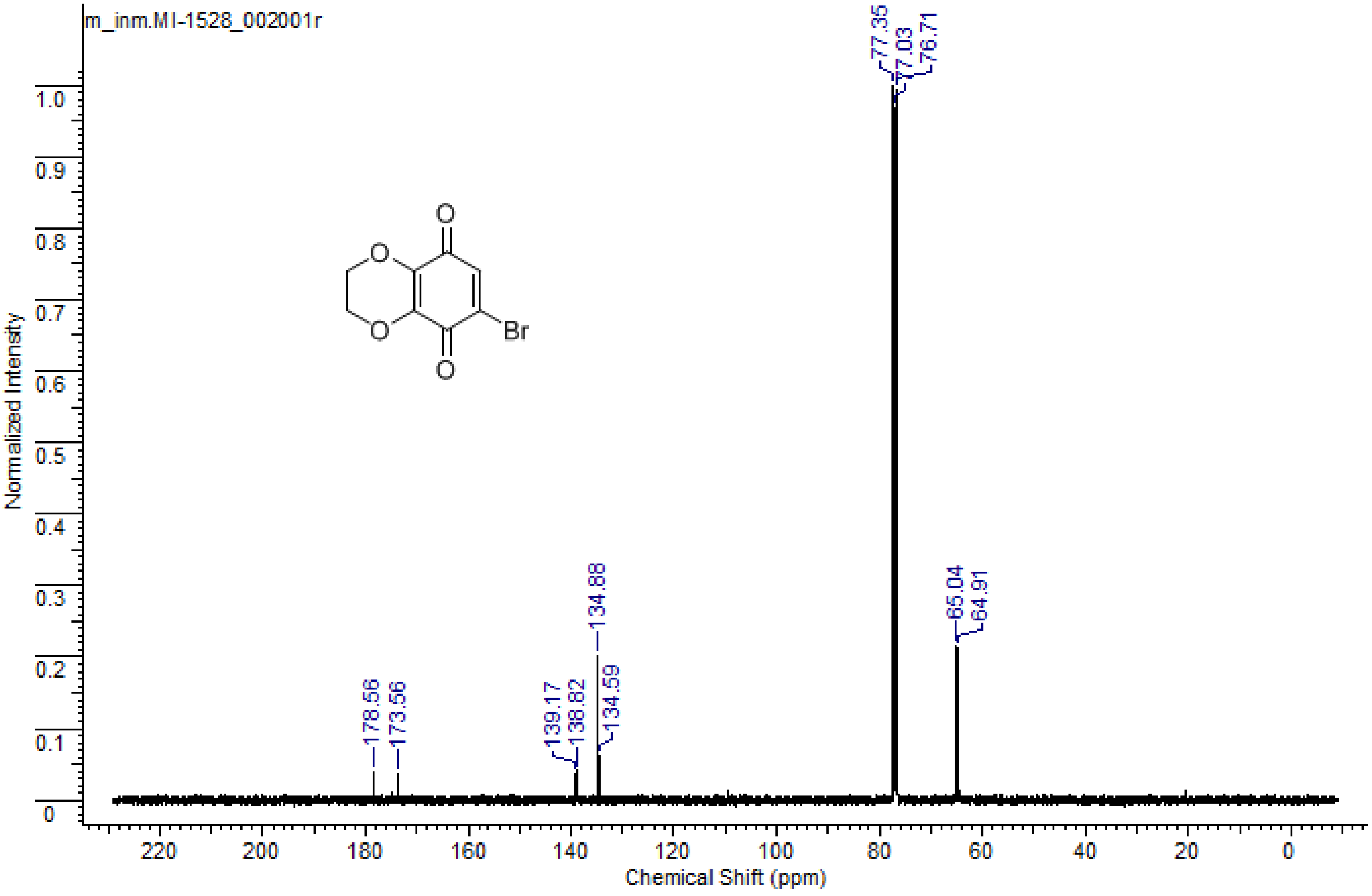

Methyl 6-methoxy-1,2-dimethyl-4,7-dioxo-4,7-dihydro-1*H*-indole-3-carboxylate (8)

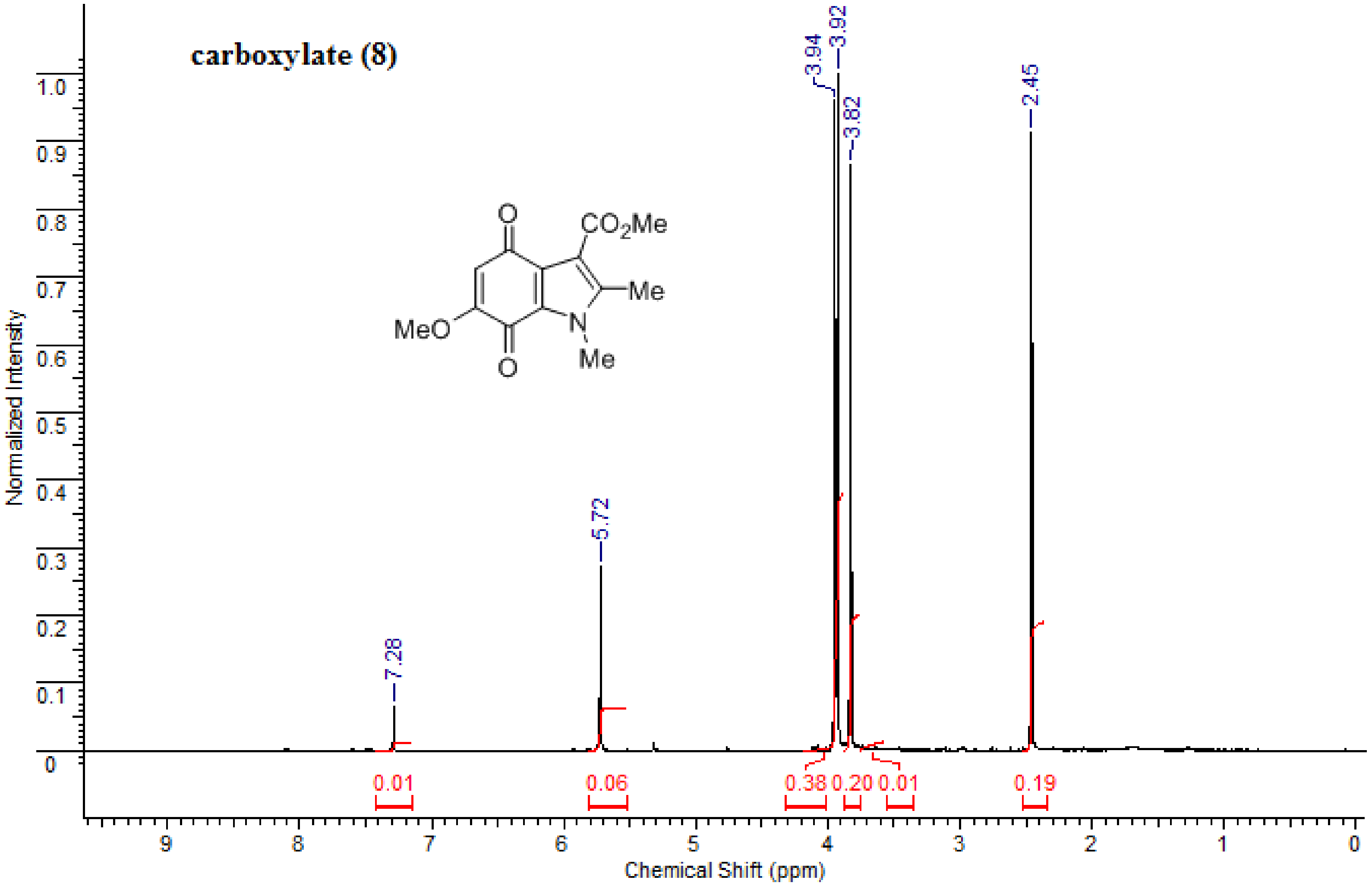

Methyl 6-methoxy-1,2-dimethyl-4,7-dioxo-4,7-dihydro-1*H*-indole-3-carboxylate (8)

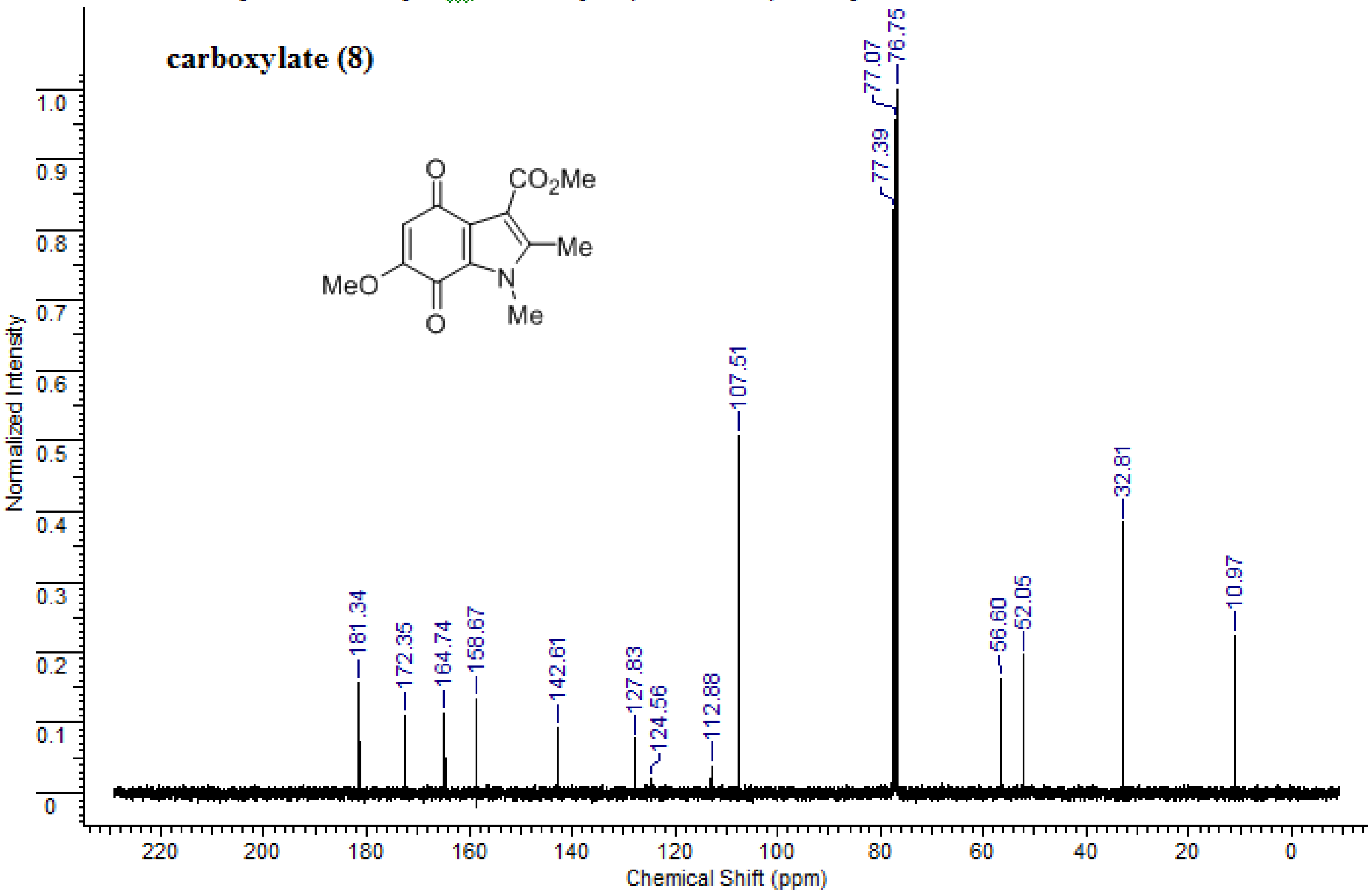

Methyl 5-methoxy-1,2-dimethyl-4,7-dioxo-4,7-dihydro-1*H*-indole-3-carboxylate (10)

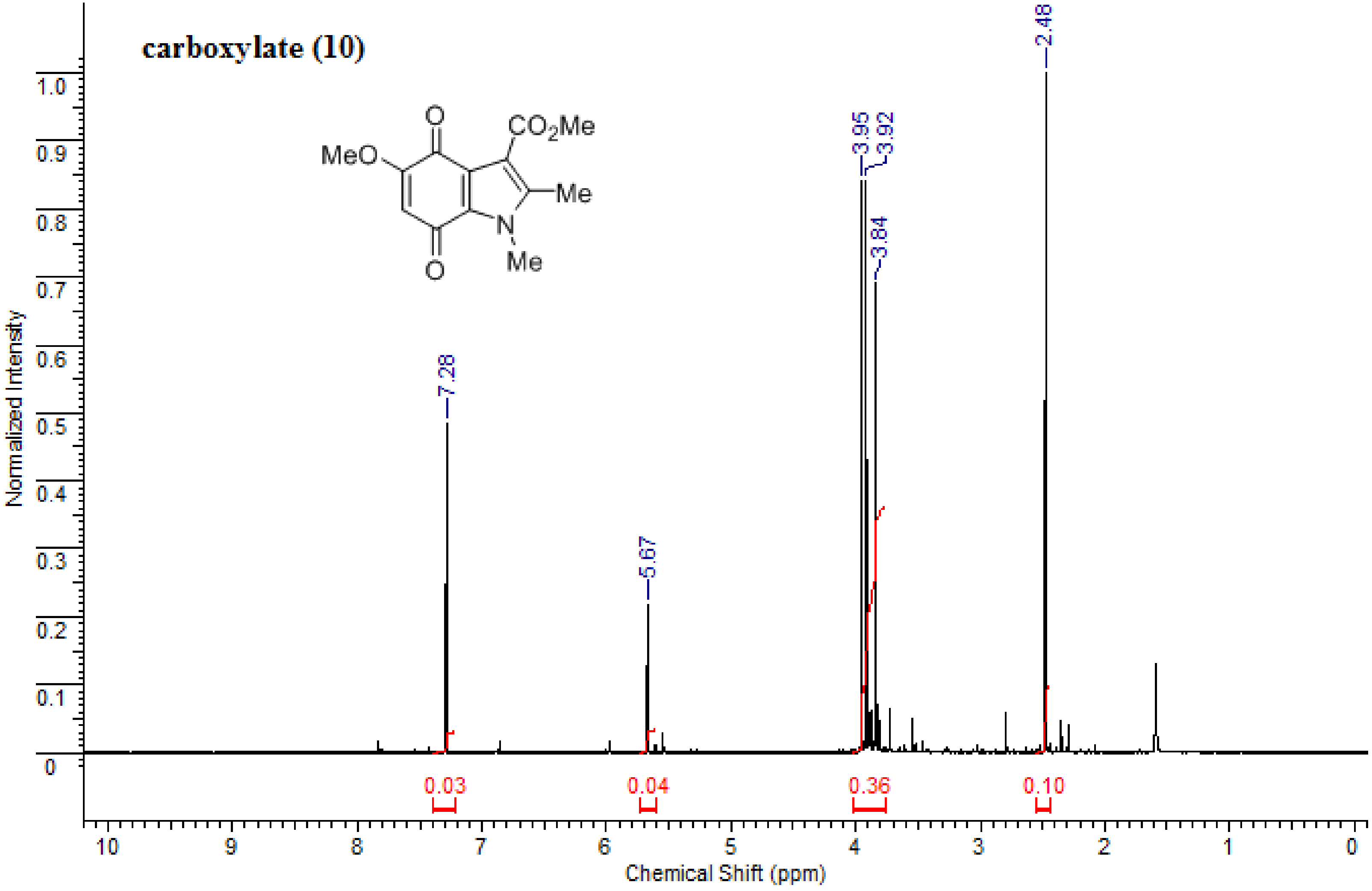

Methyl 5-methoxy-1,2-dimethyl-4,7-dioxo-4,7-dihydro-1*H*-indole-3-carboxylate (10)

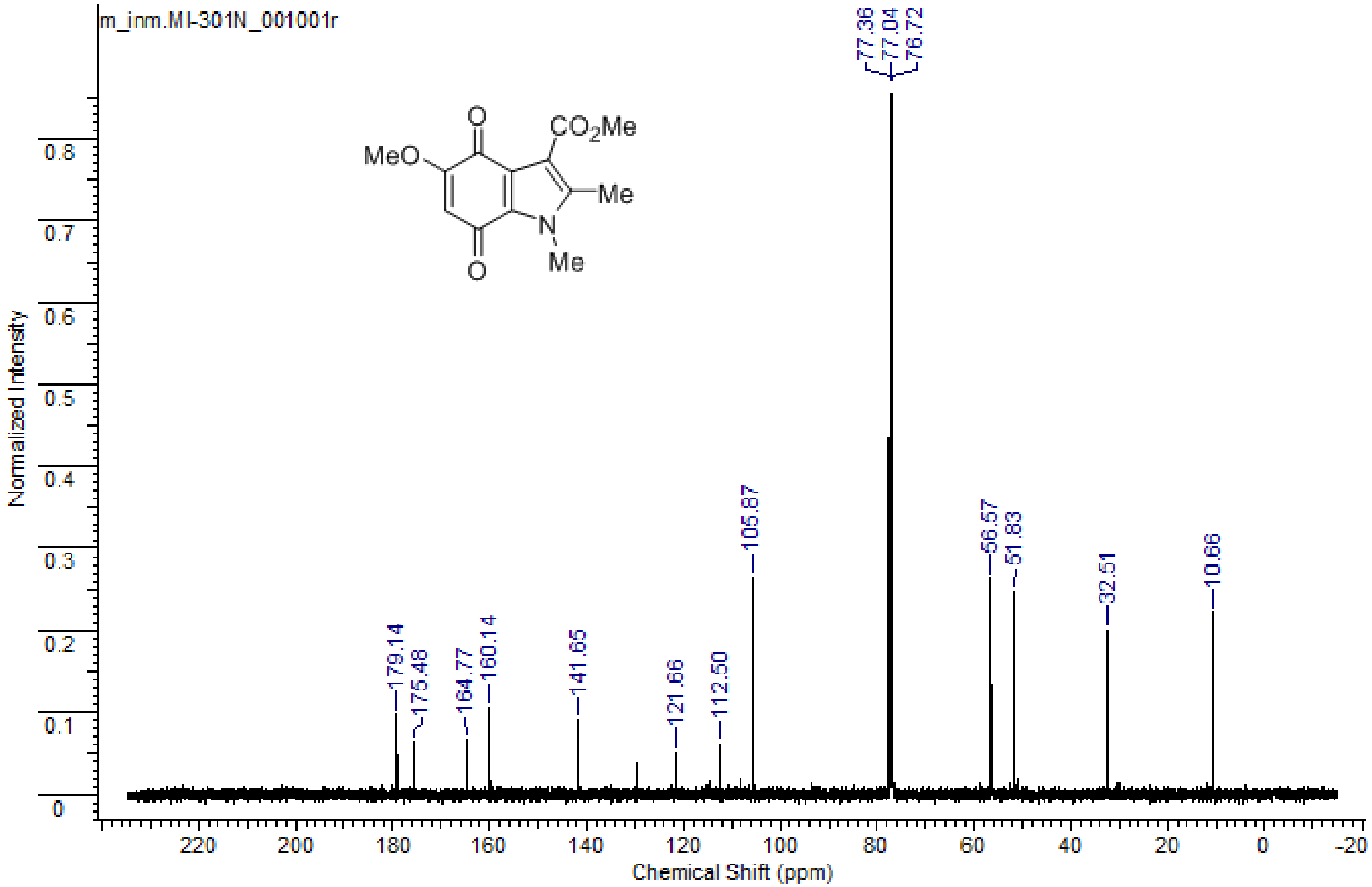

Methyl 6-methoxy-1-methyl-4,7-dioxo-4,7-dihydro-1*H*-indole-3-carboxylate (11)

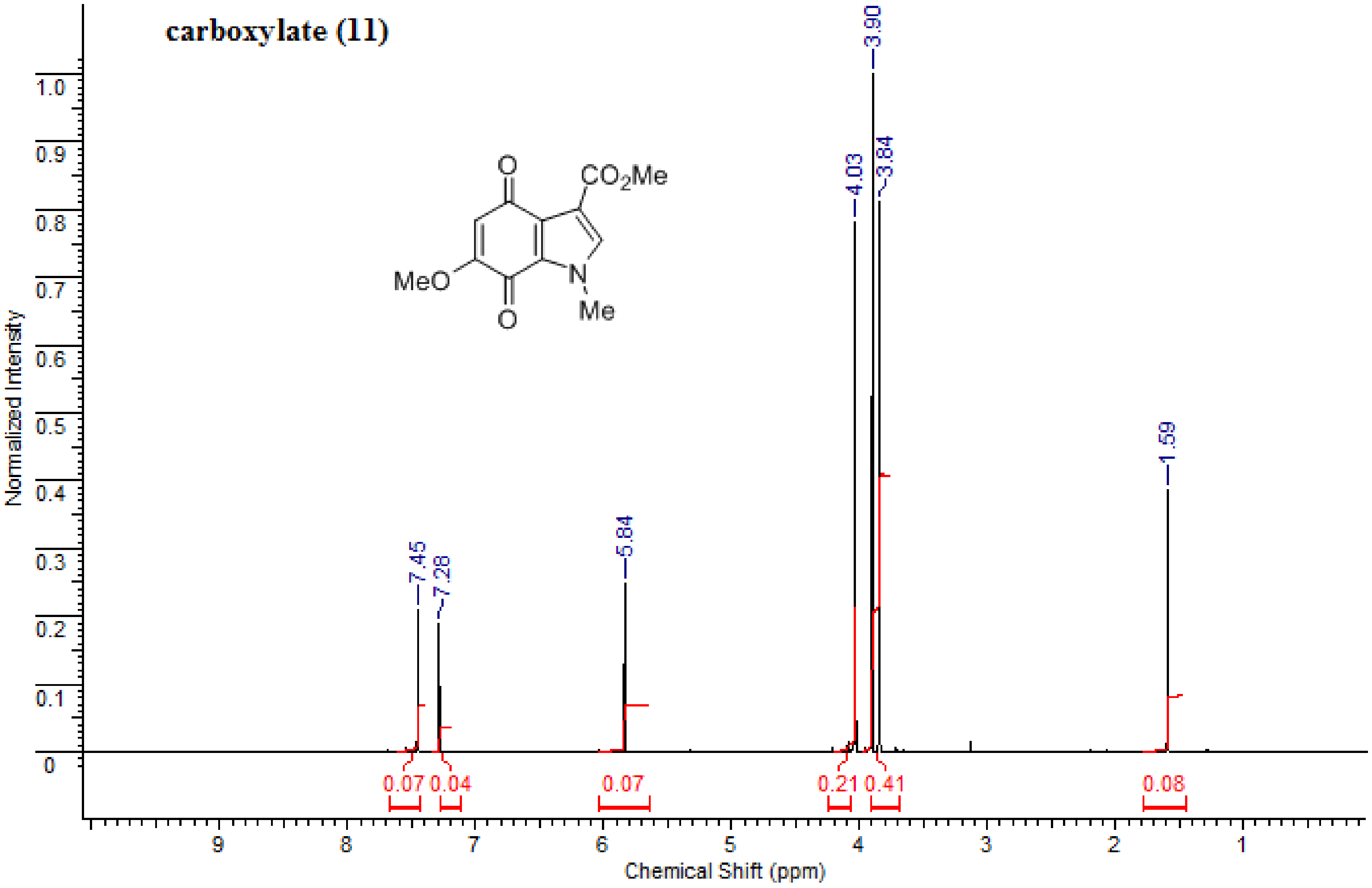

Methyl 6-methoxy-1-methyl-4,7-dioxo-4,7-dihydro-1*H*-indole-3-carboxylate (11)

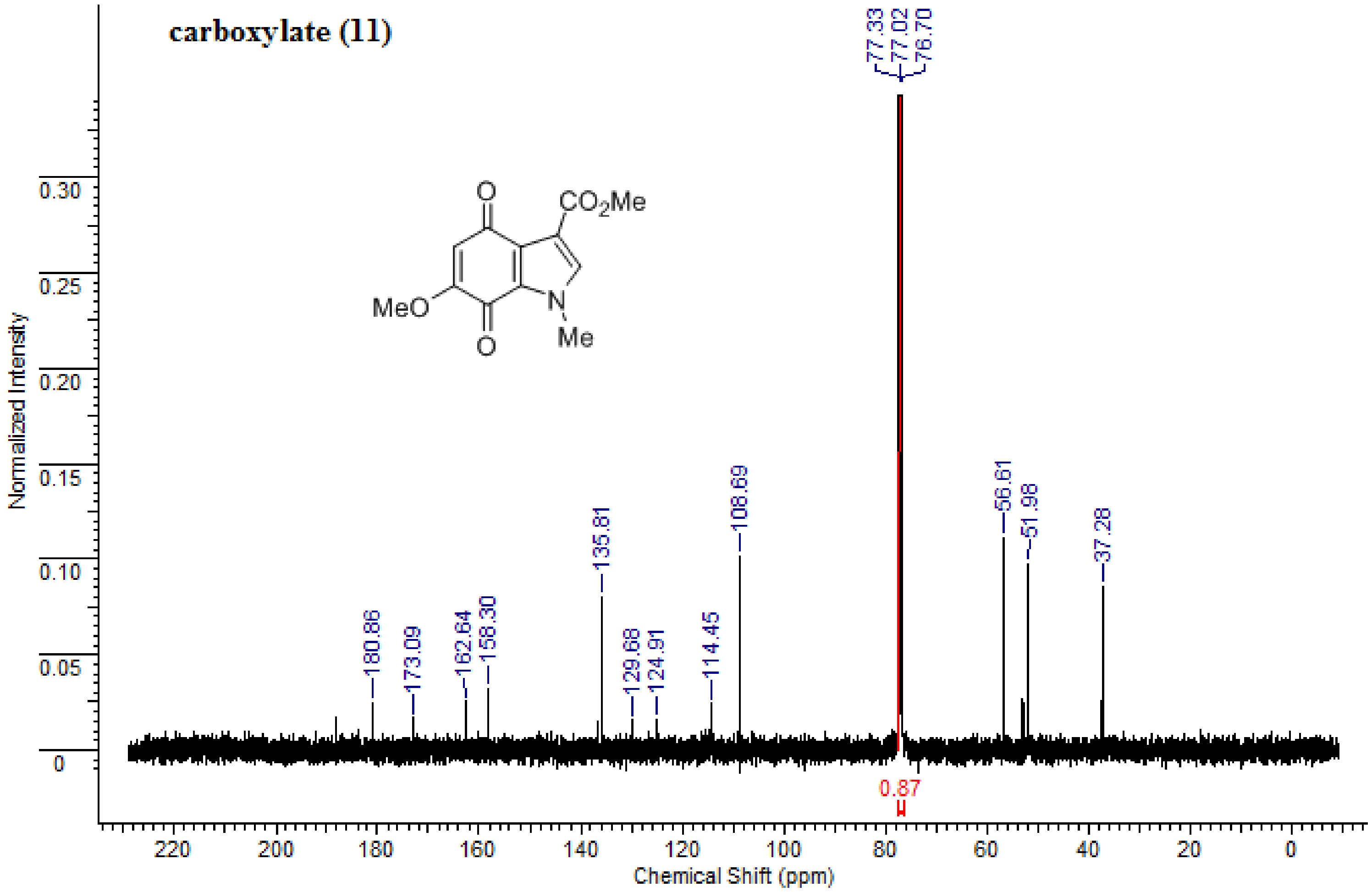

Ethyl 2-(allyloxymethyl)-6-methoxy-1-methyl-4,7-dioxo-4,7-dihydro-1*H*-indole-3-carboxylate (12)

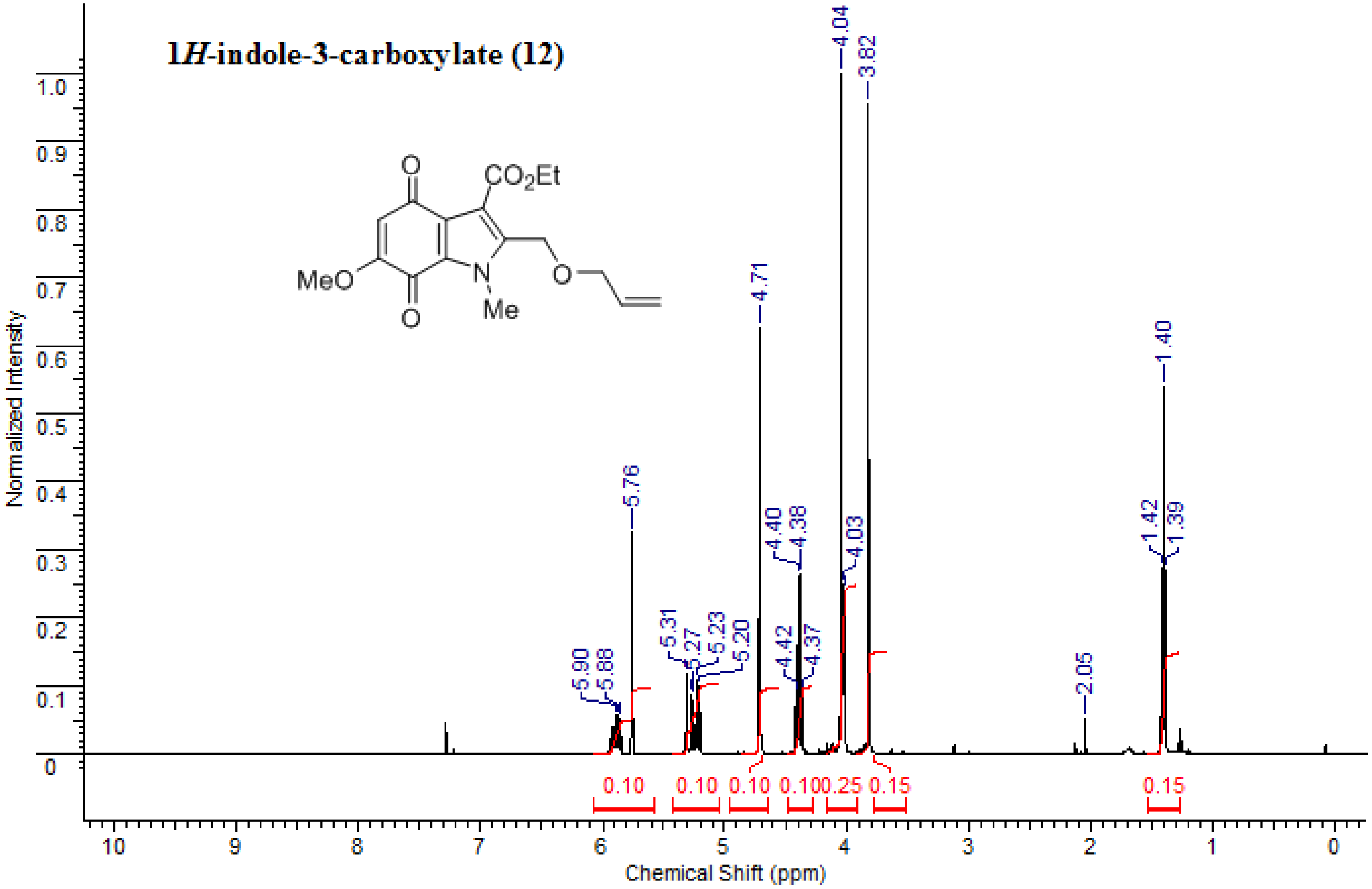

Ethyl 2-(allyloxymethyl)-6-methoxy-1-methyl-4,7-dioxo-4,7-dihydro-1*H*-indole-3-carboxylate (12)

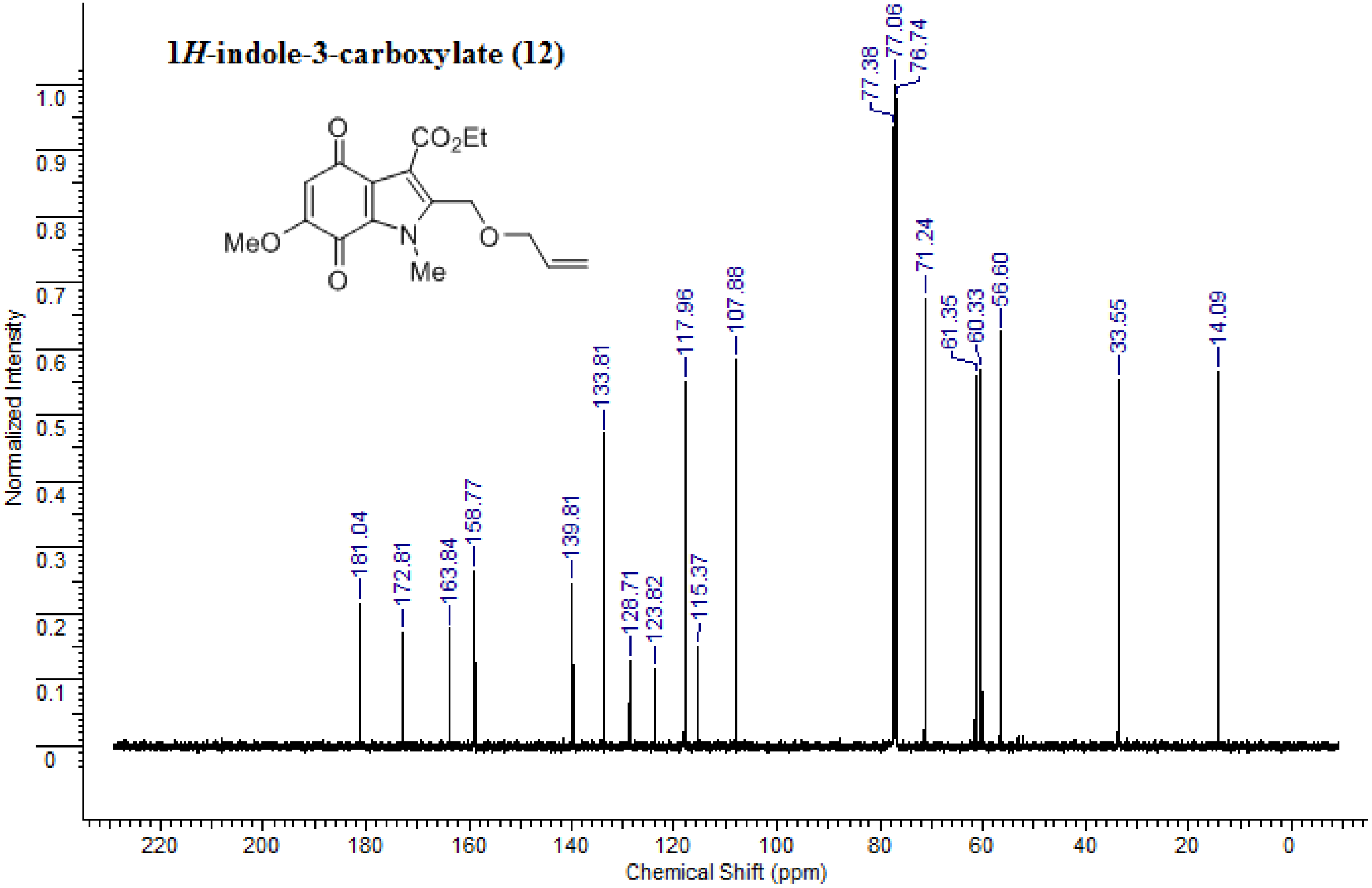

***tert*-Butyl 6-methoxy-1,2-dimethyl-4,7-dioxo-4,7-dihydro-1*H*-indole-3-carboxylate (13)**

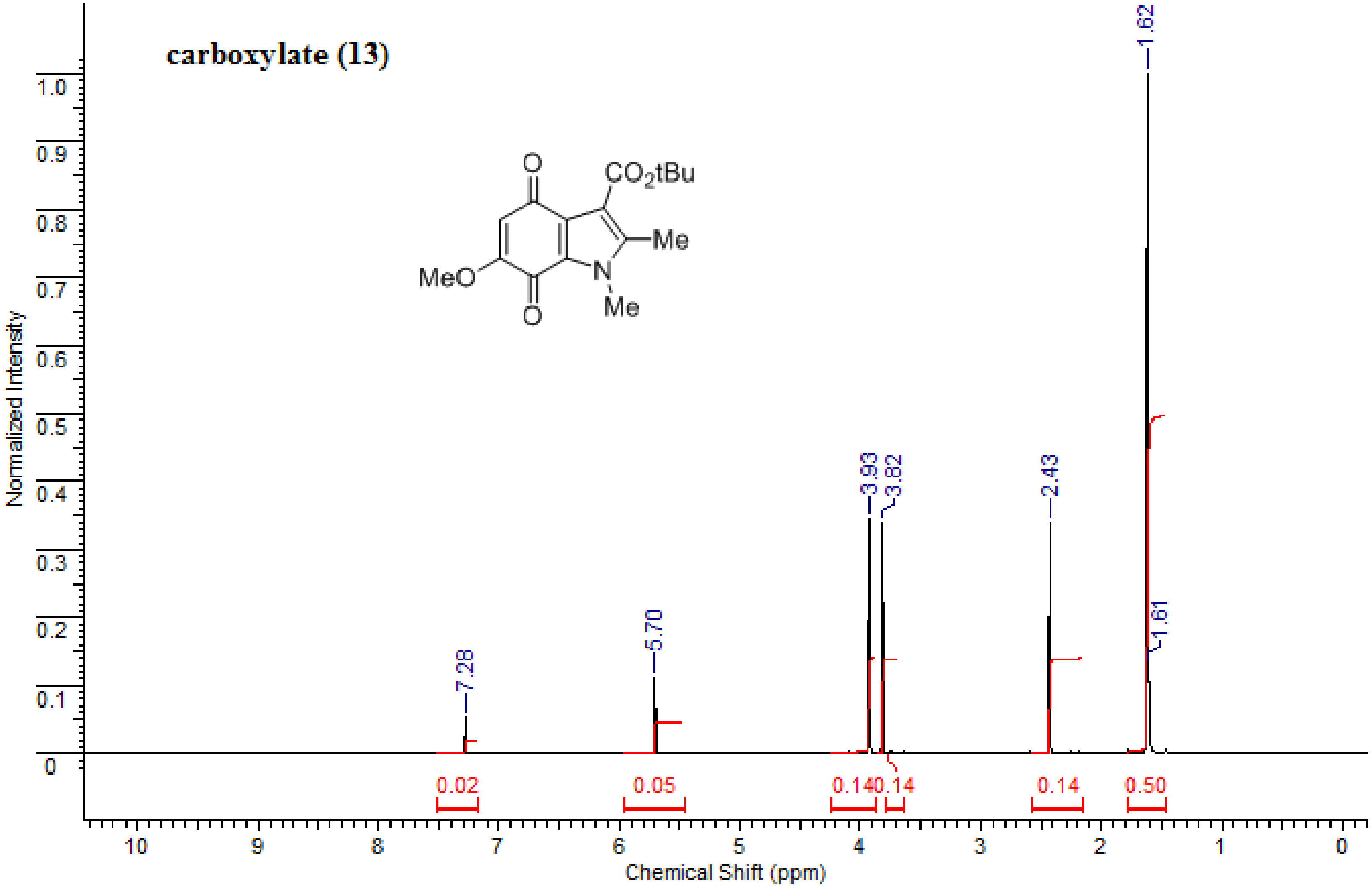

***tert*-Butyl 6-methoxy-1,2-dimethyl-4,7-dioxo-4,7-dihydro-1*H*-indole-3-carboxylate (13)**

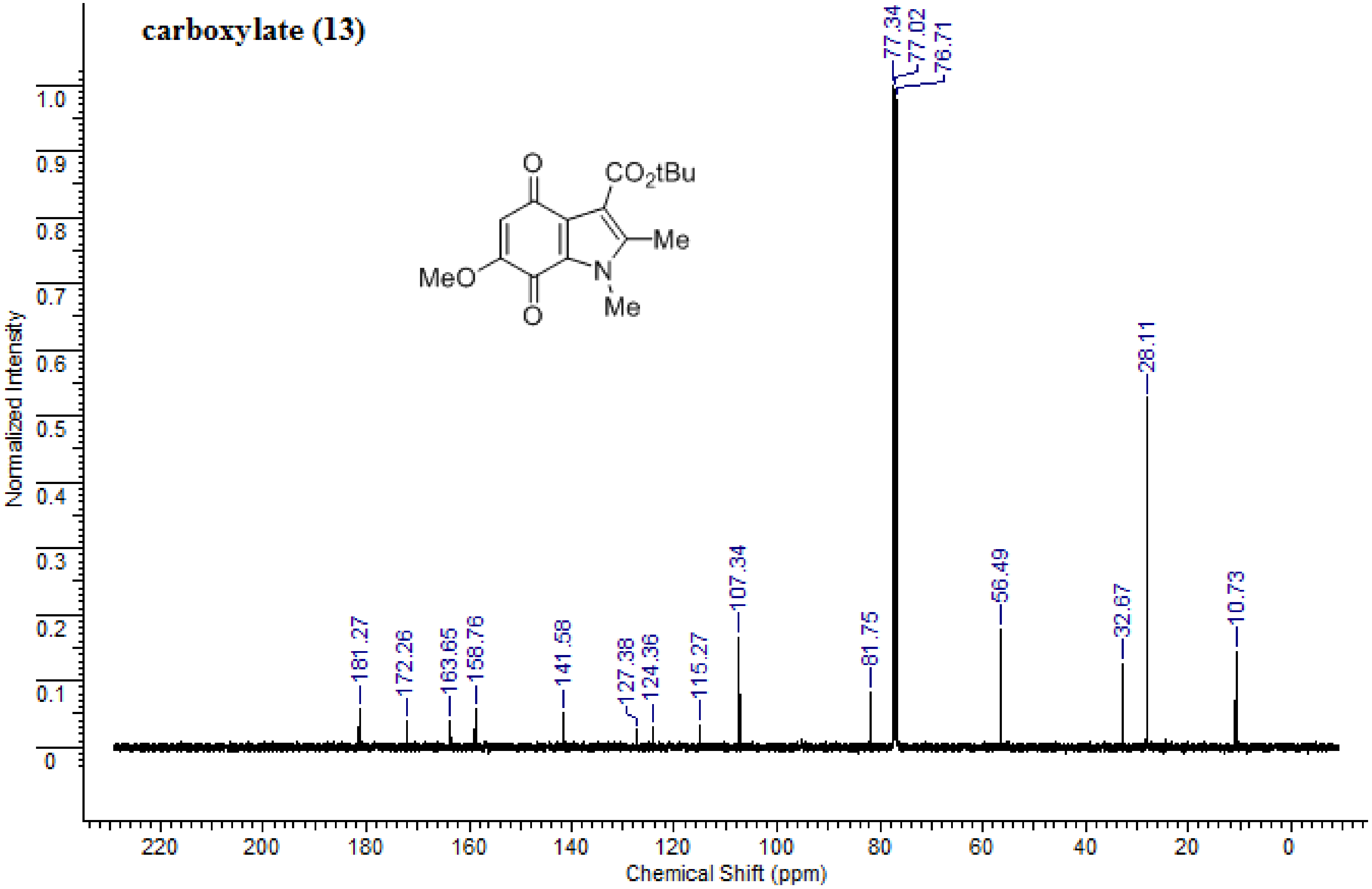

Methyl 1,2-dimethyl-4,9-dioxo-4,9-dihydro-1*H*-benzo[*f*]indole-3-carboxylate (14)

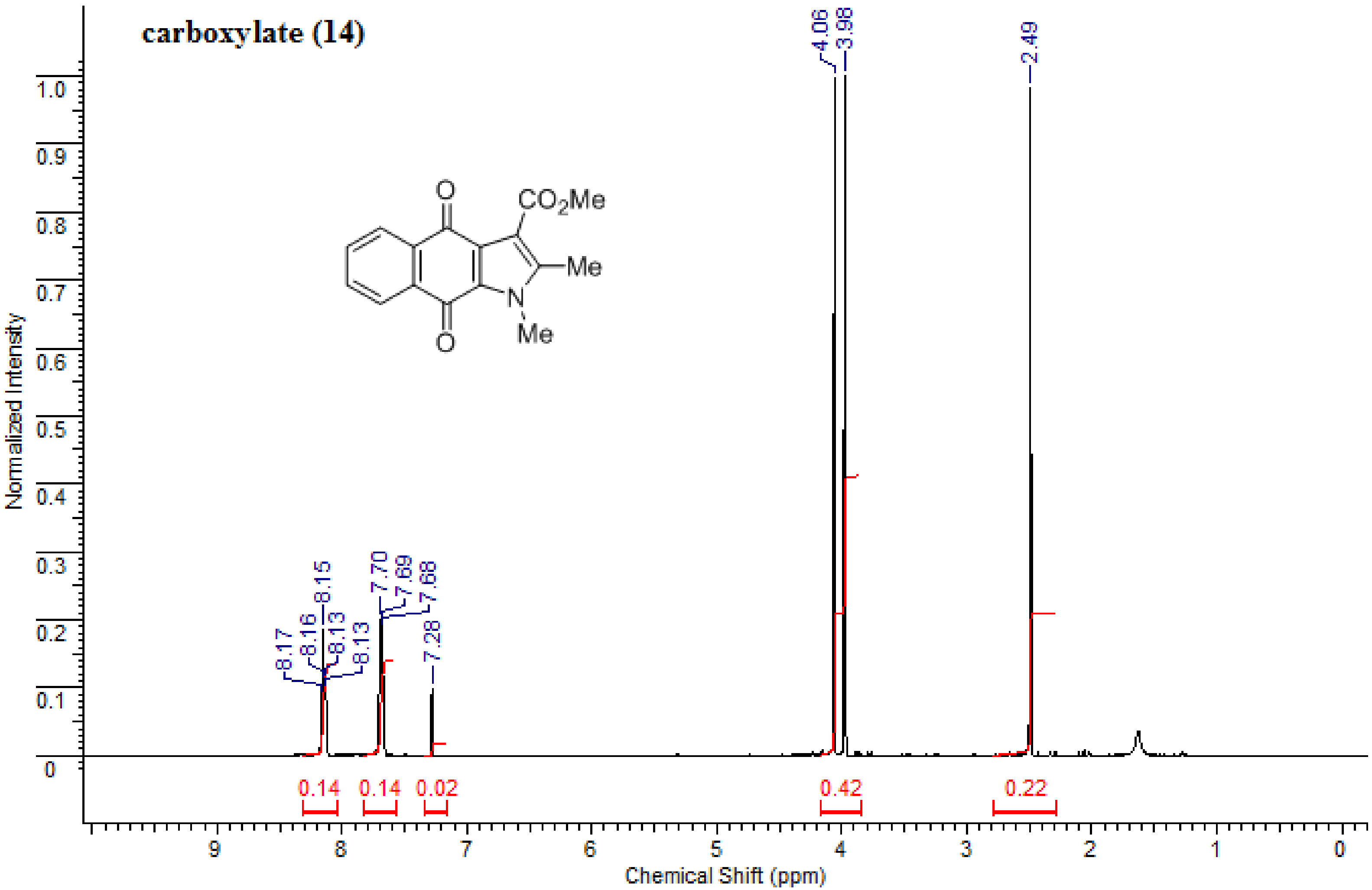

Methyl 1,2-dimethyl-4,9-dioxo-4,9-dihydro-1*H*-benzo[*f*]indole-3-carboxylate (14)

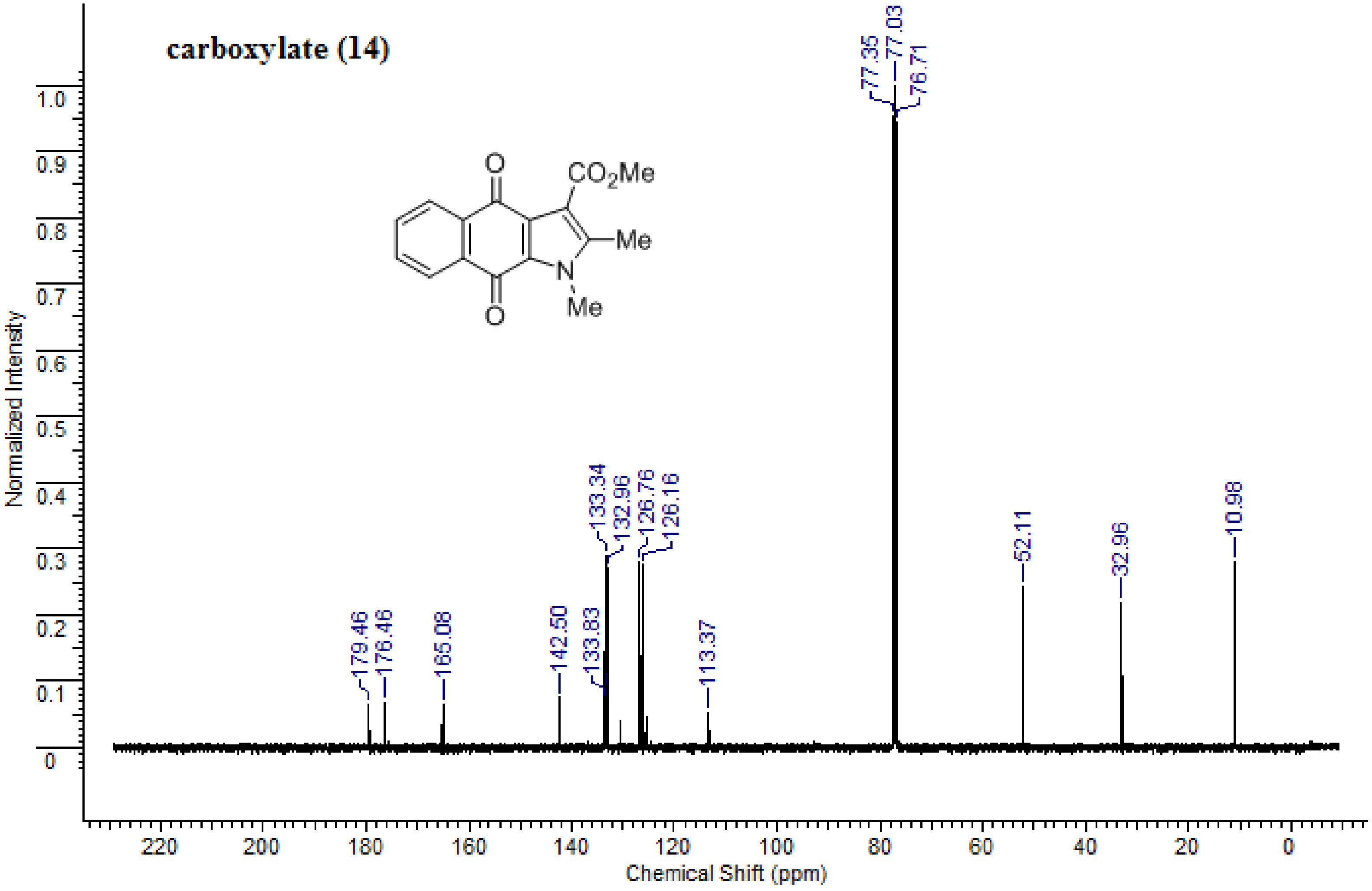

3-Acetyl-6-methoxy-1,2-dimethyl-1*H*-indole-4,7-dione (15)

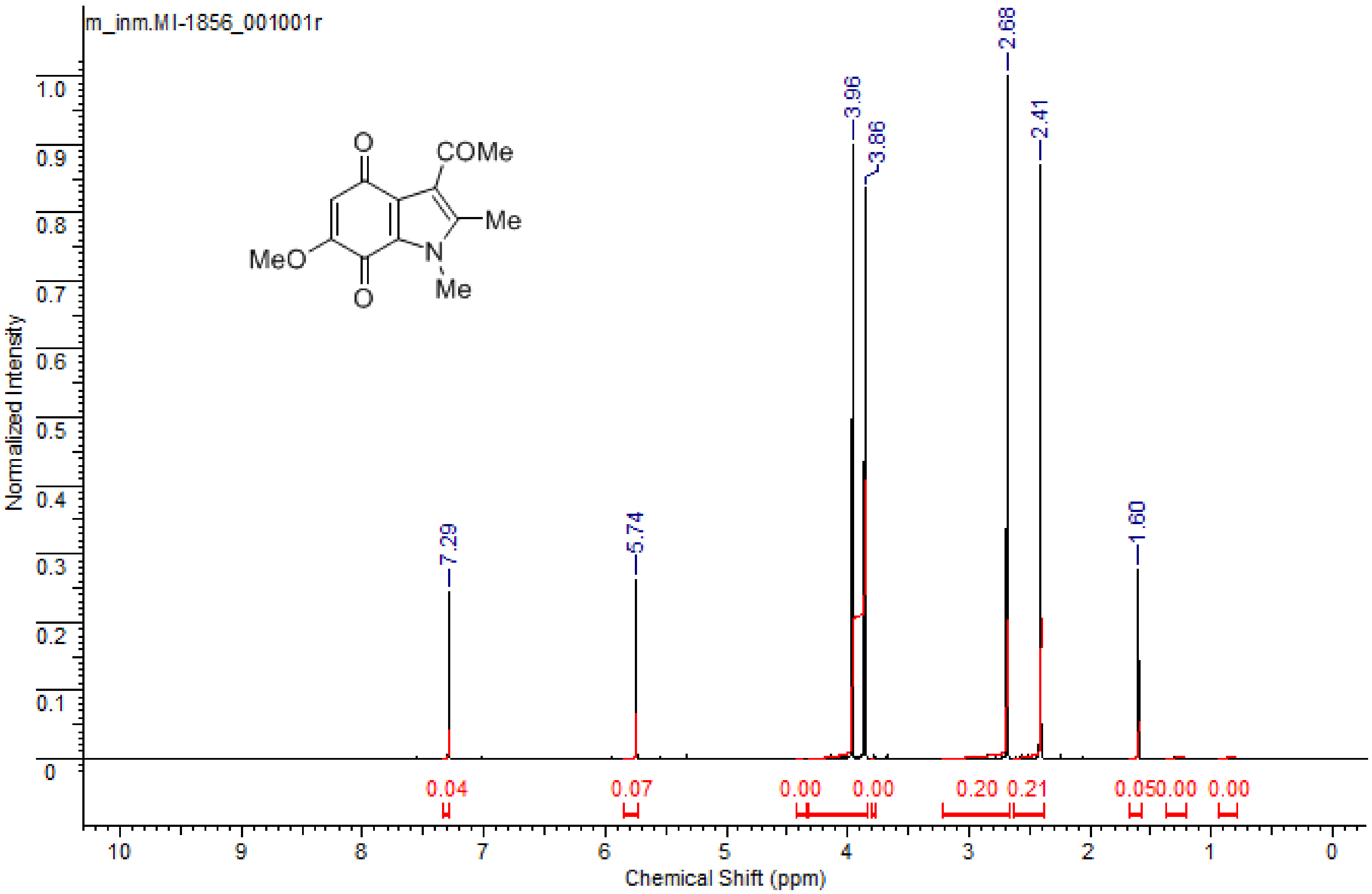

3-Acetyl-6-methoxy-1,2-dimethyl-1*H*-indole-4,7-dione (15)

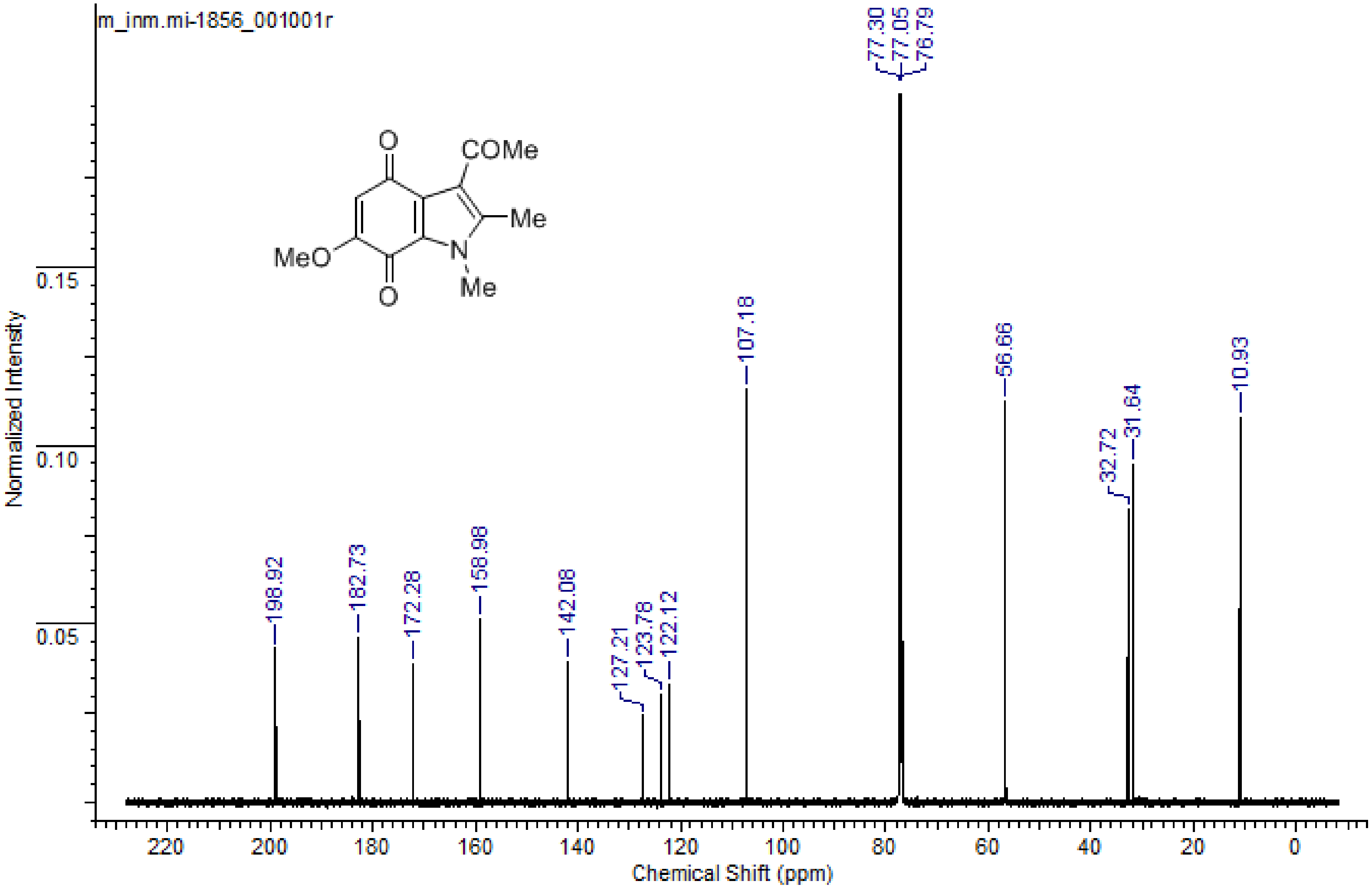

Methyl 6-methoxy-1-(4-methoxybenzyl)-2-methyl-4,7-dioxo-4,7-dihydro-1*H*-indole-3-carboxylate (16)

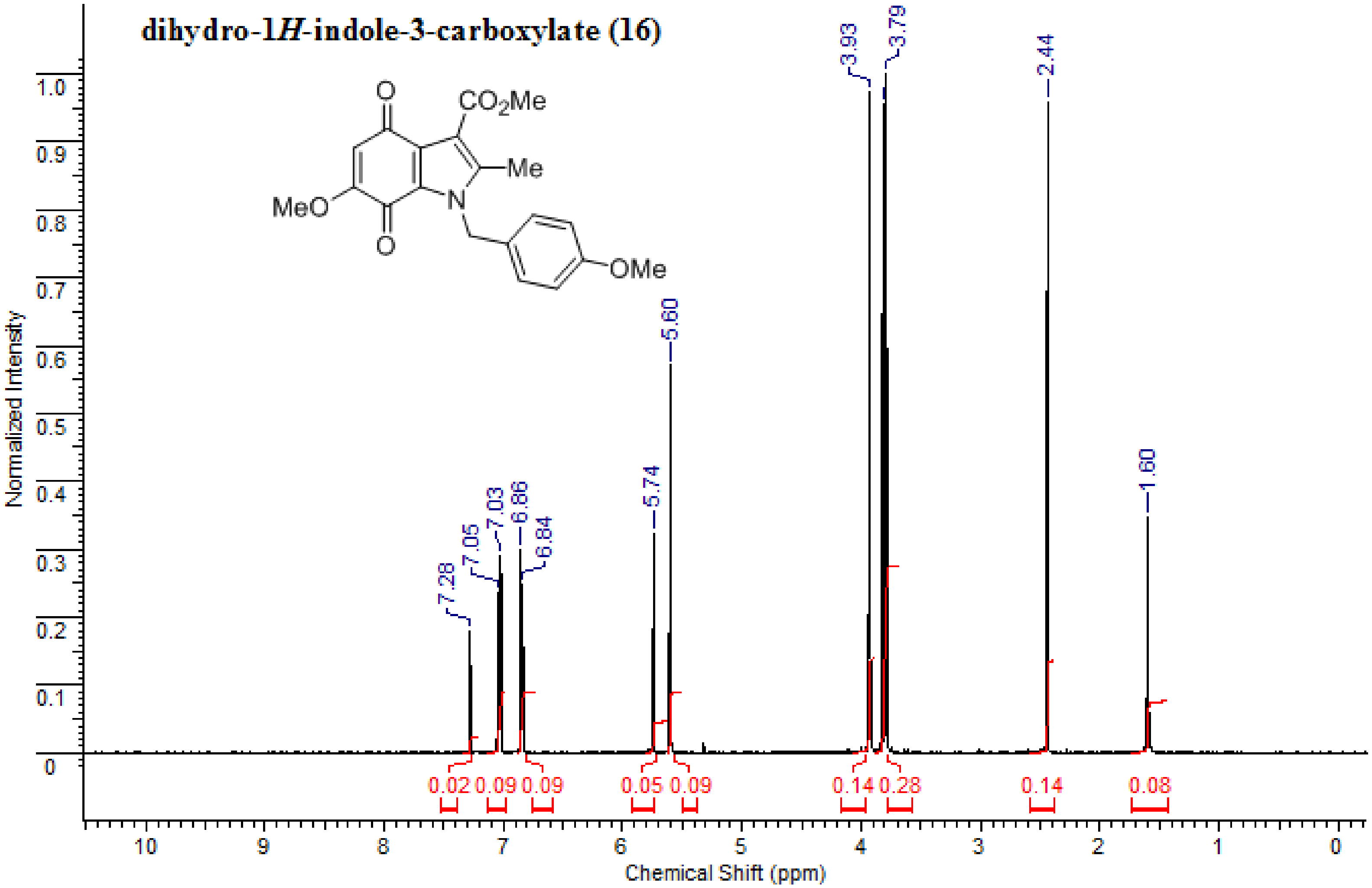

Methyl 6-methoxy-1-(4-methoxybenzyl)-2-methyl-4,7-dioxo-4,7-dihydro-1*H*-indole-3-carboxylate (16)

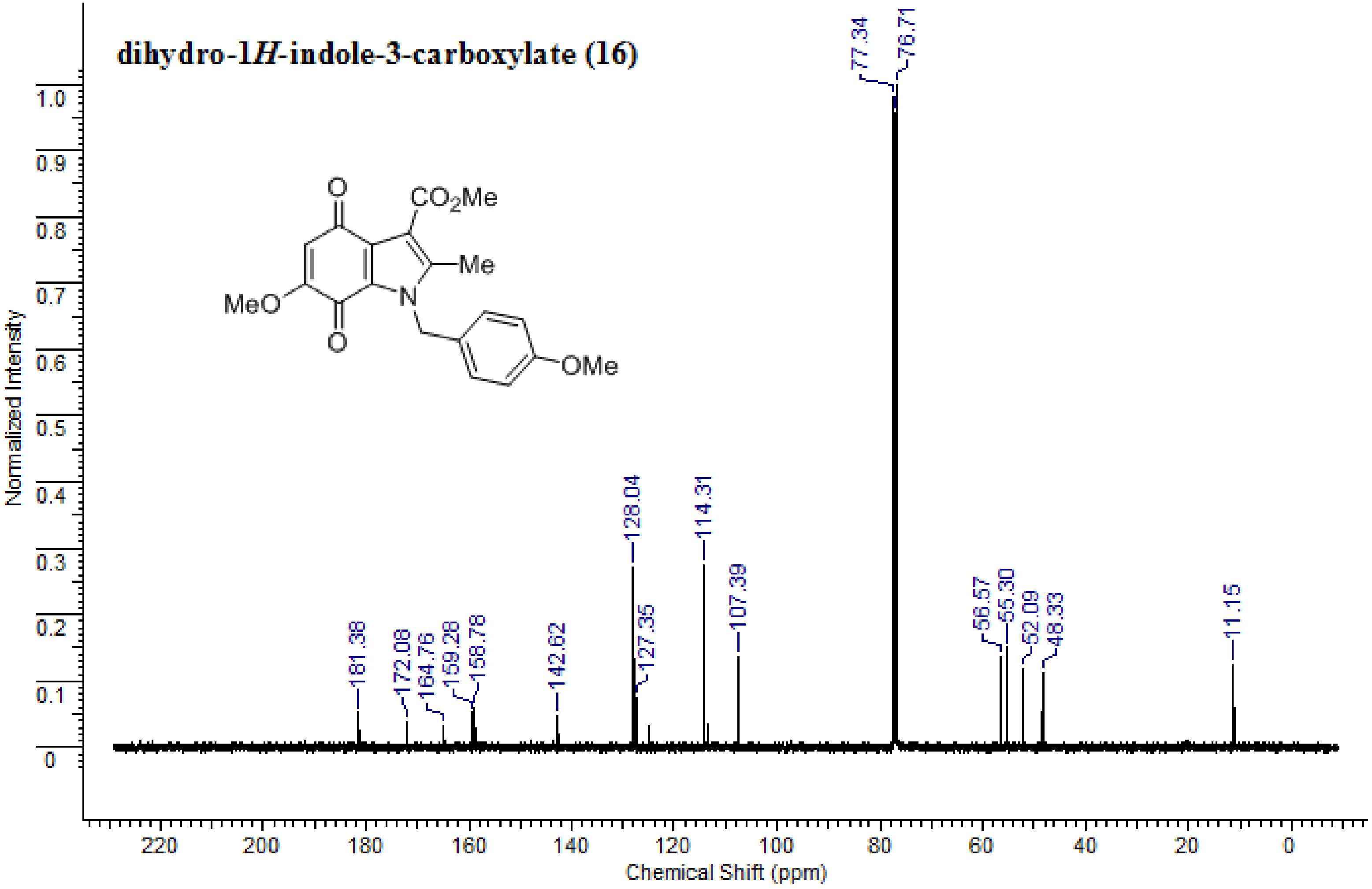

***tert*-Butyl 6-methoxy-1-(3-methoxyphenyl)-2-methyl-4,7-dioxo-4,7-dihydro-1*H*-indole-3-carboxylate (17)**

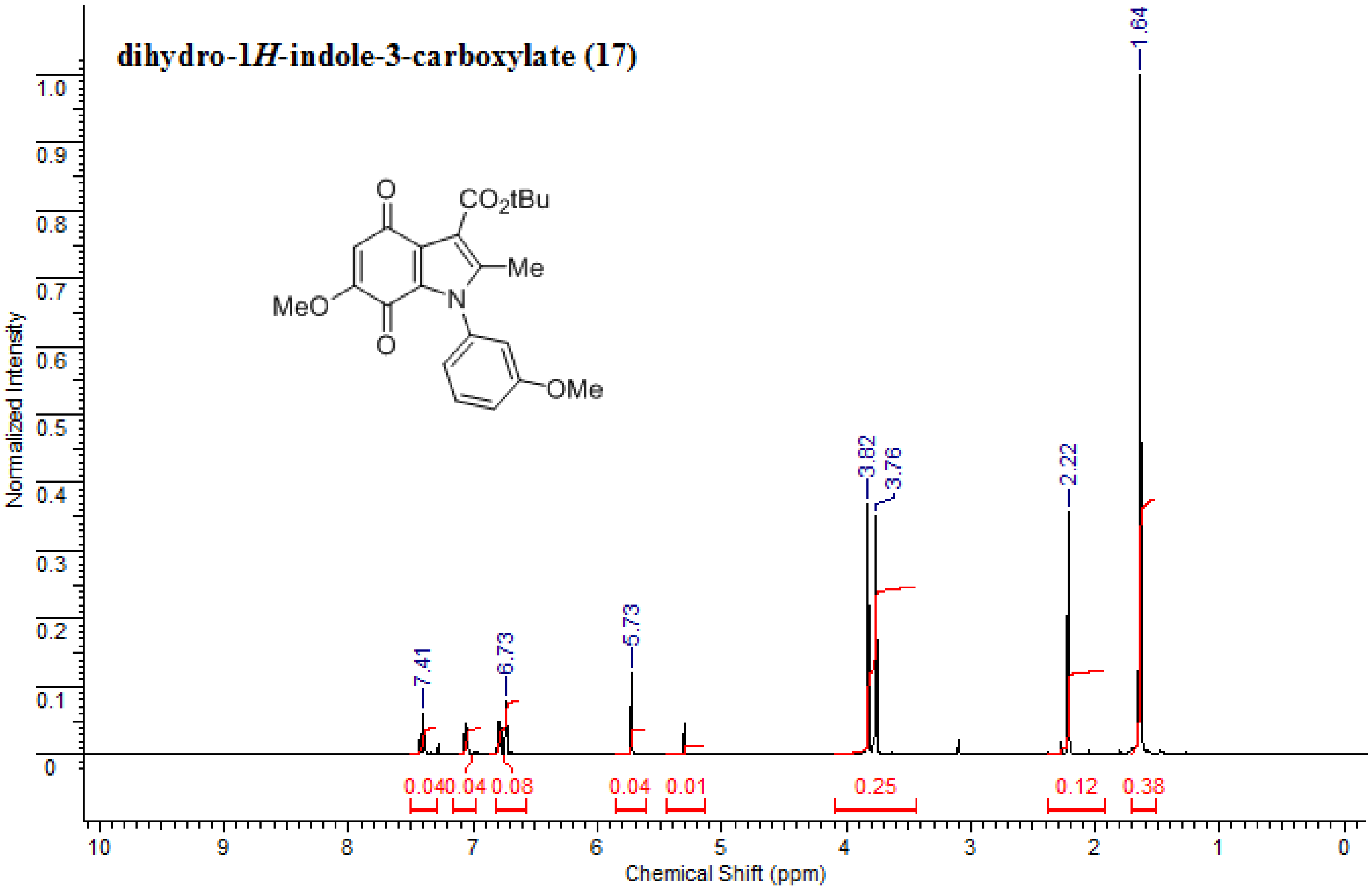

tert-Butyl 6-methoxy-1-(3-methoxyphenyl)-2-methyl-4,7-dioxo-4,7-dihydro-1_H_-indole-3-carboxylate (17)

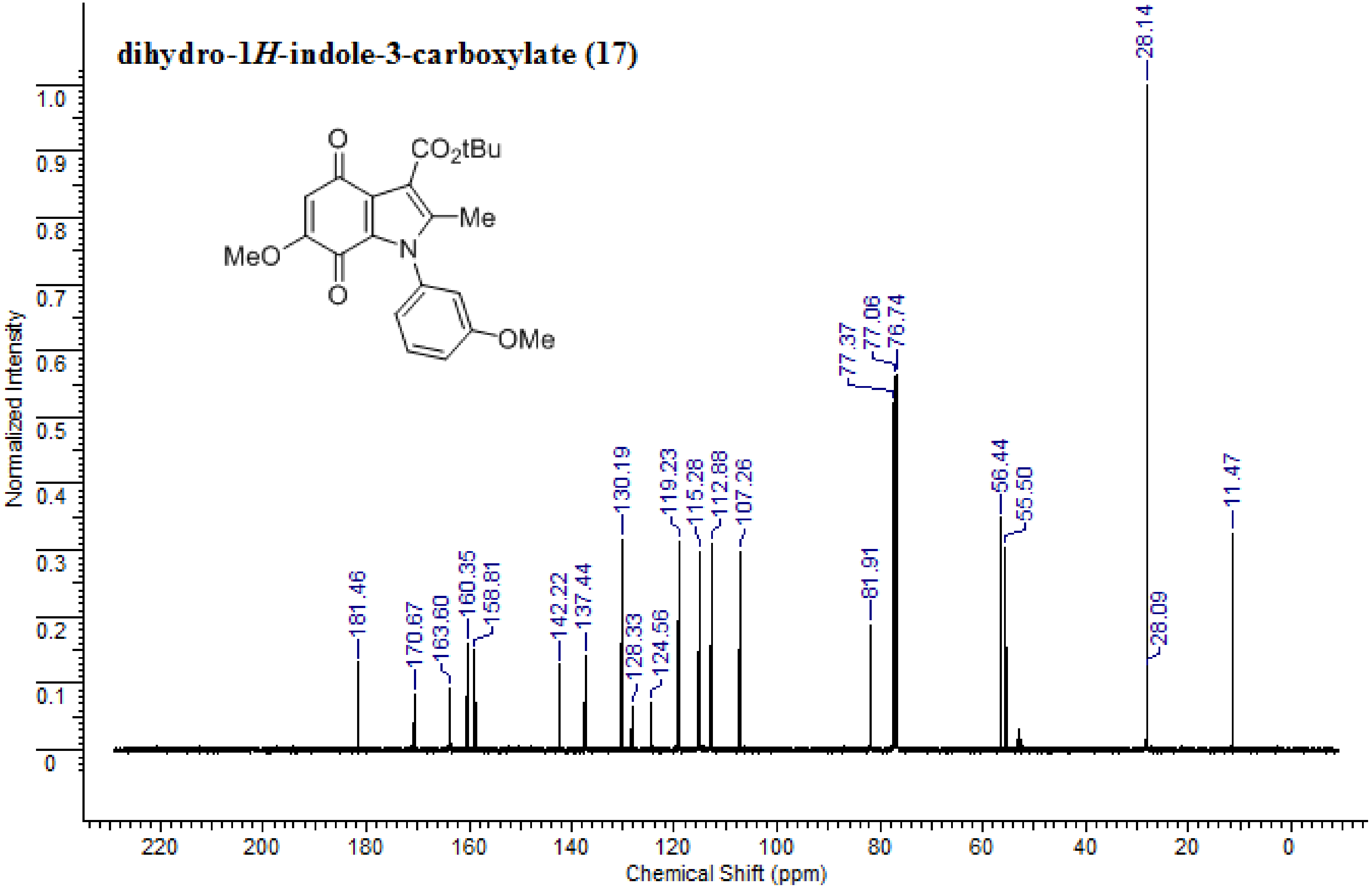

***tert*-Butyl 2-methyl-1-(2-morpholinoethyl)-4,9-dioxo-4,9-dihydro-1*H*-benzo[*f*]indole-3-carboxylate (18)**

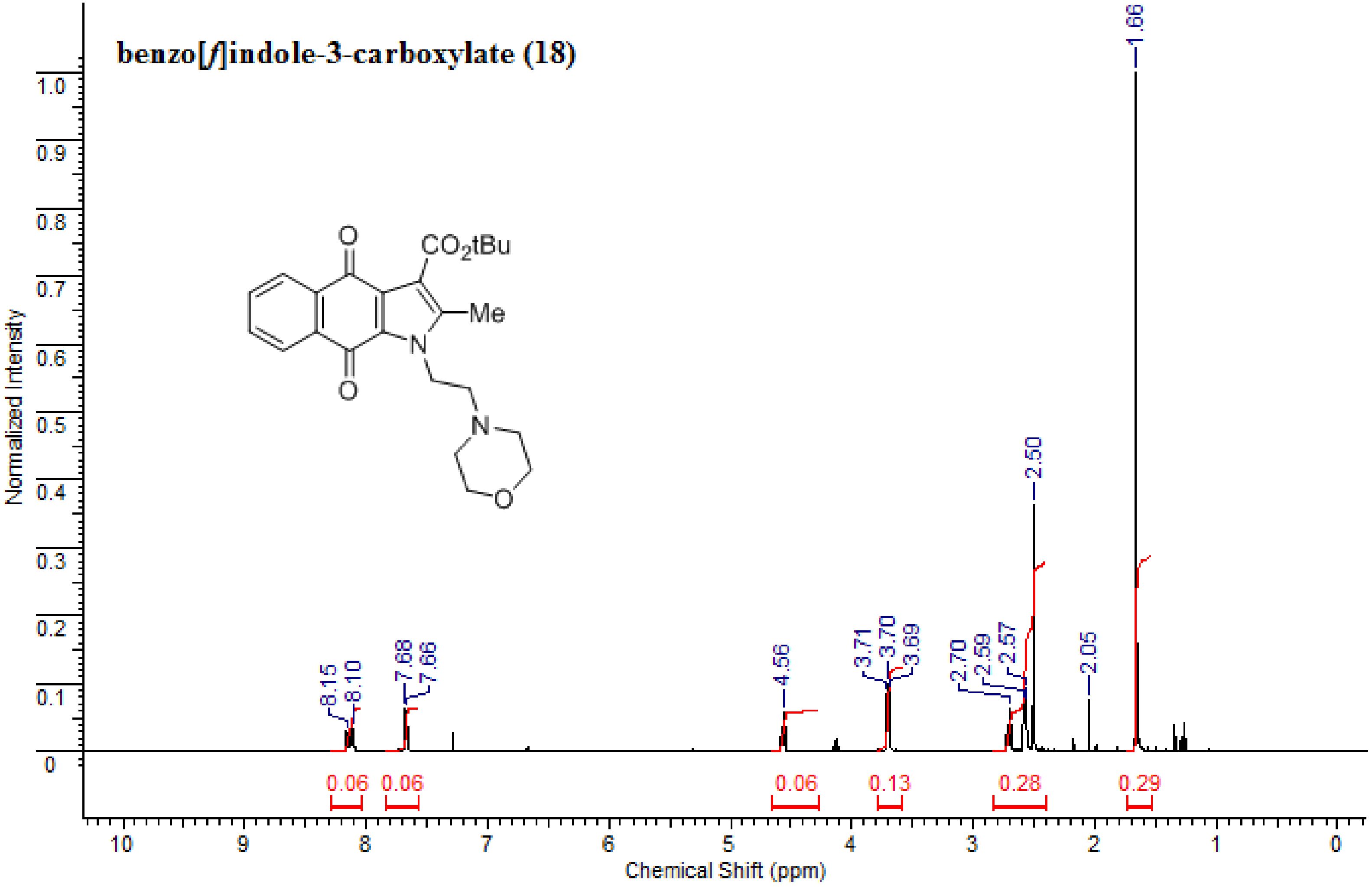

tert-Butyl 2-methyl-1-(2-morpholinoethyl)-4,9-dioxo-4,9-dihydro-1_H_-benzo[_f_]indole-3-carboxylate (18)

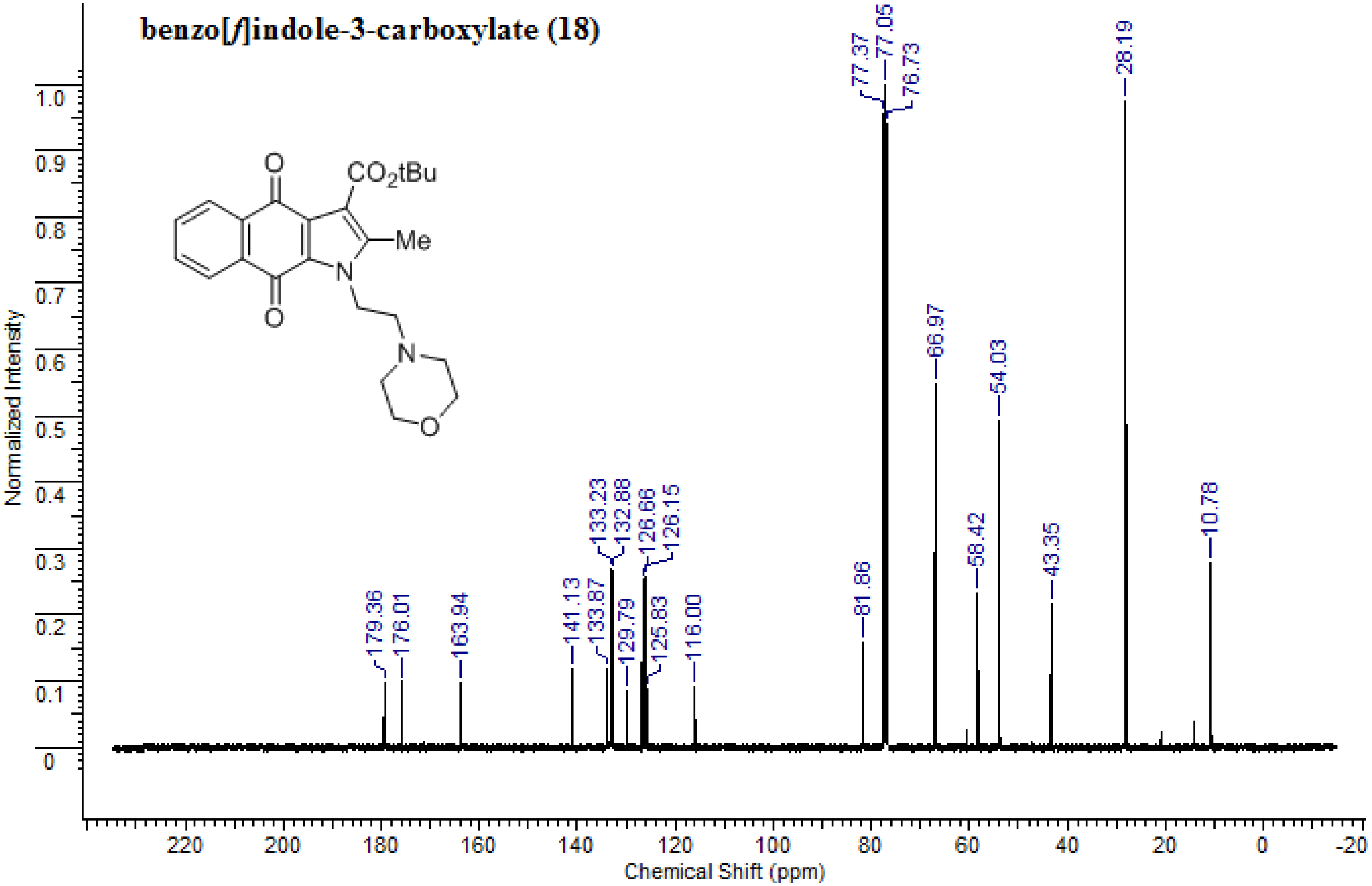

Dimethyl 6-methoxy-1-(4-methoxybenzyl)-4,7-dioxo-4,7-dihydro-1*H*-indole-2,3-dicarboxylate (19)

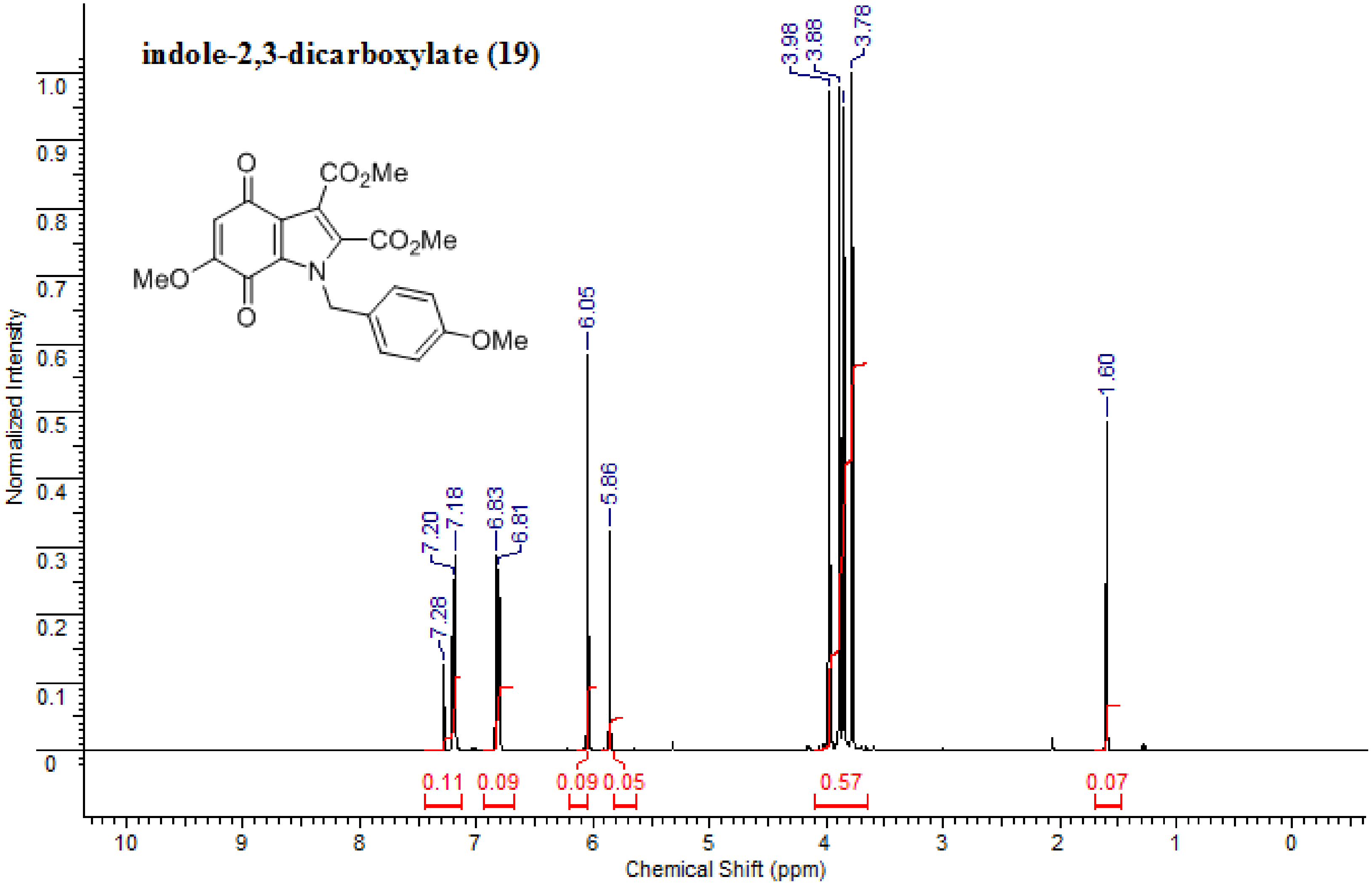

Dimethyl 6-methoxy-1-(4-methoxybenzyl)-4,7-dioxo-4,7-dihydro-1*H*-indole-2,3-dicarboxylate (19)

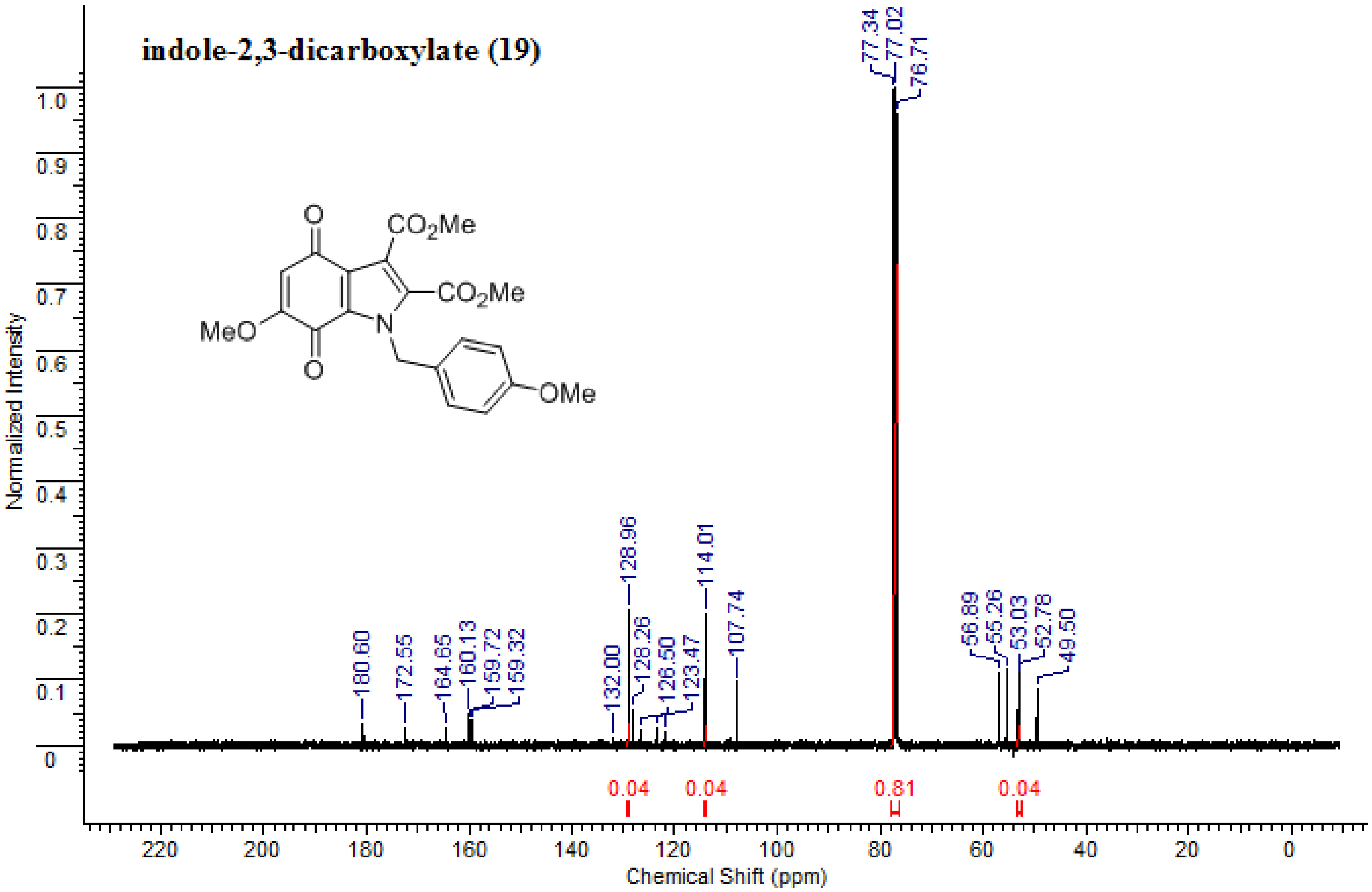

Methyl 1-(2-(*tert*-butyldimethylsiloxy)ethyl)-6-methoxy-2-methyl-4,7-dioxo-4,7-dihydro-1*H*-indole-3-carboxylate (20)

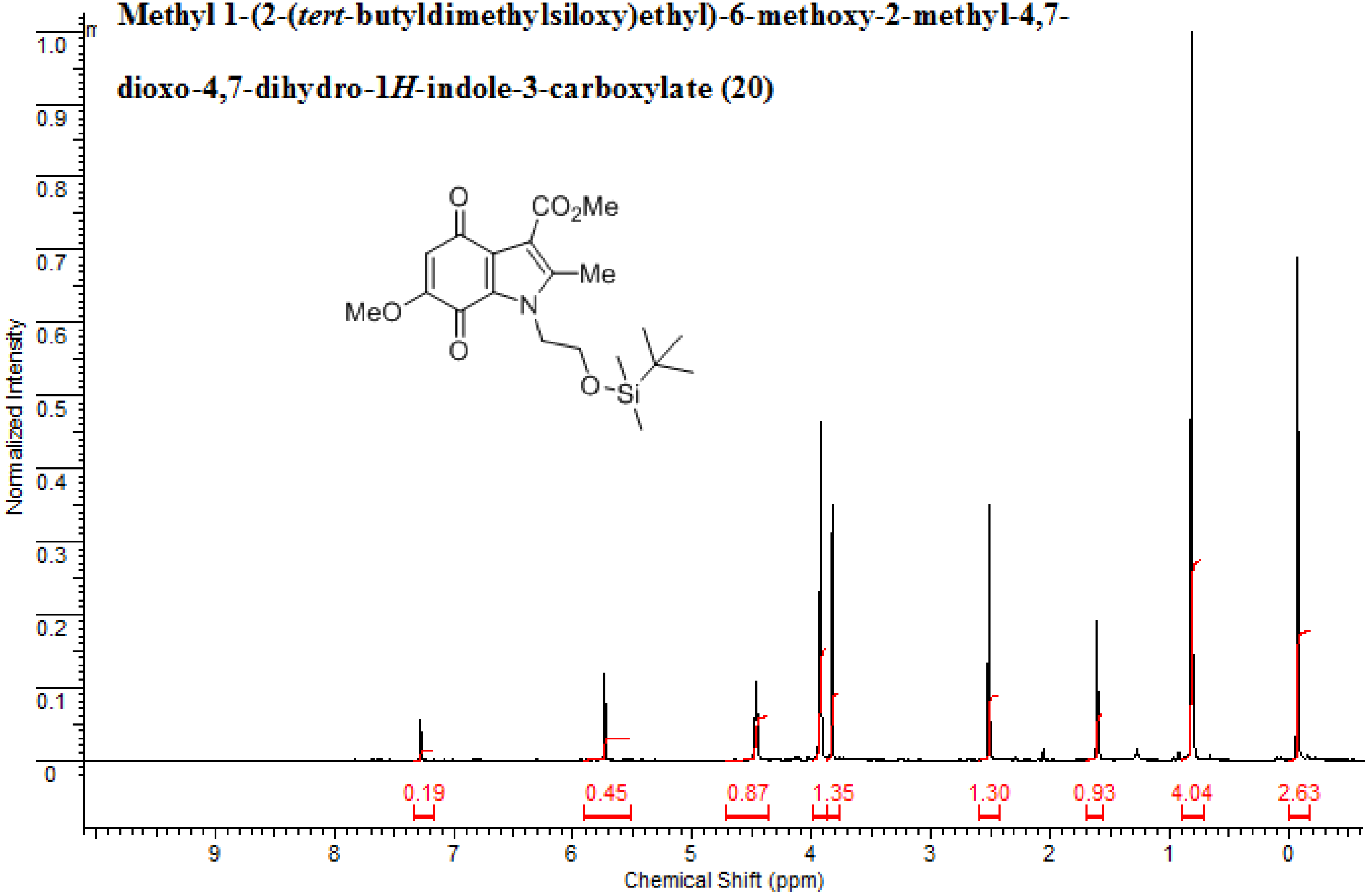

Methyl 1-(2-(*tert*-butyldimethylsiloxy)ethyl)-6-methoxy-2-methyl-4,7-dioxo-4,7-dihydro-1*H*-indole-3-carboxylate (20)

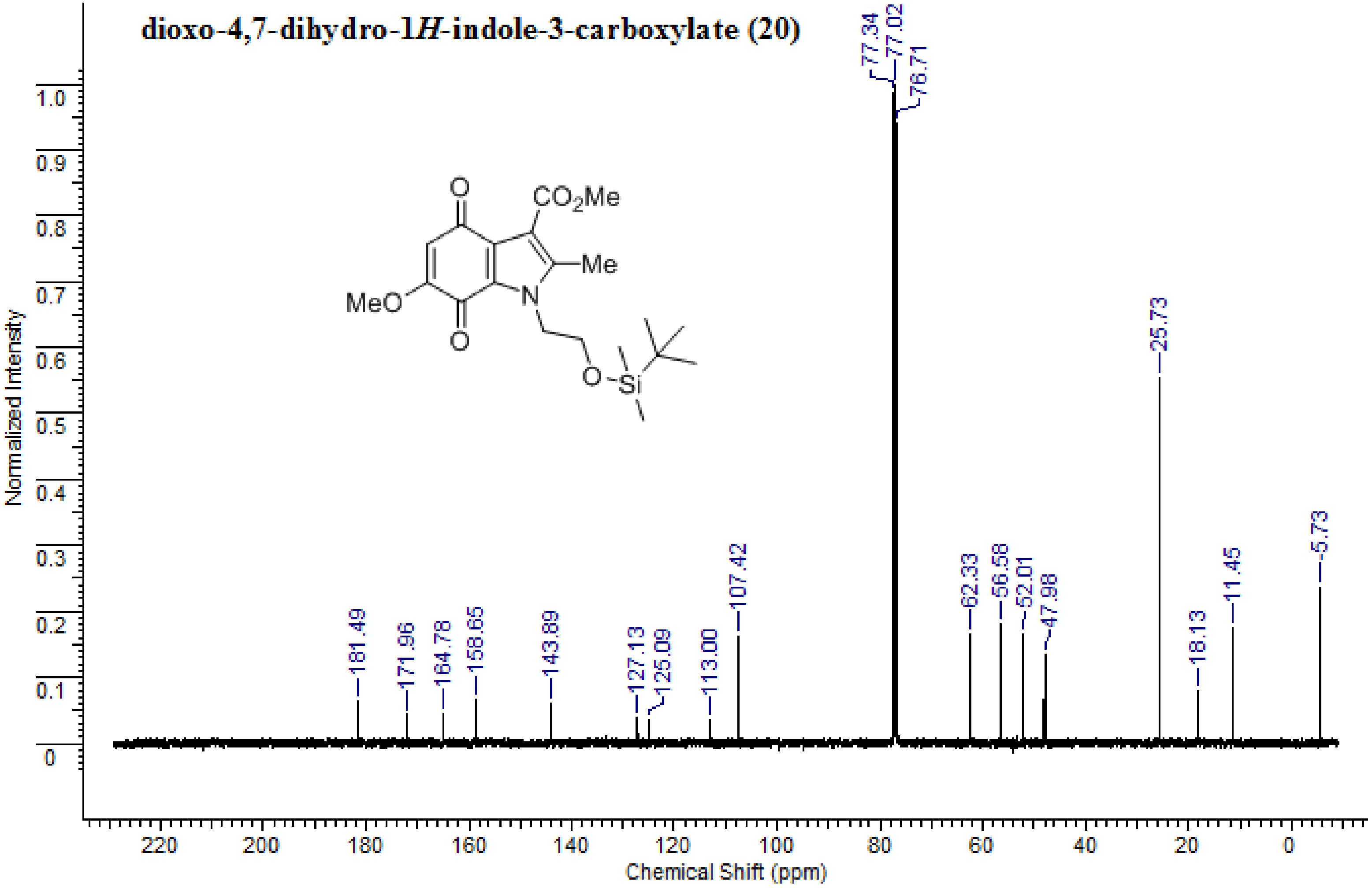

Methyl 1-(2-(*tert*-butoxycarbonylamino)ethyl)-6-methoxy-2-methyl-4,7-dioxo-4,7-dihydro-1*H*-indole-3-carboxylate (21)

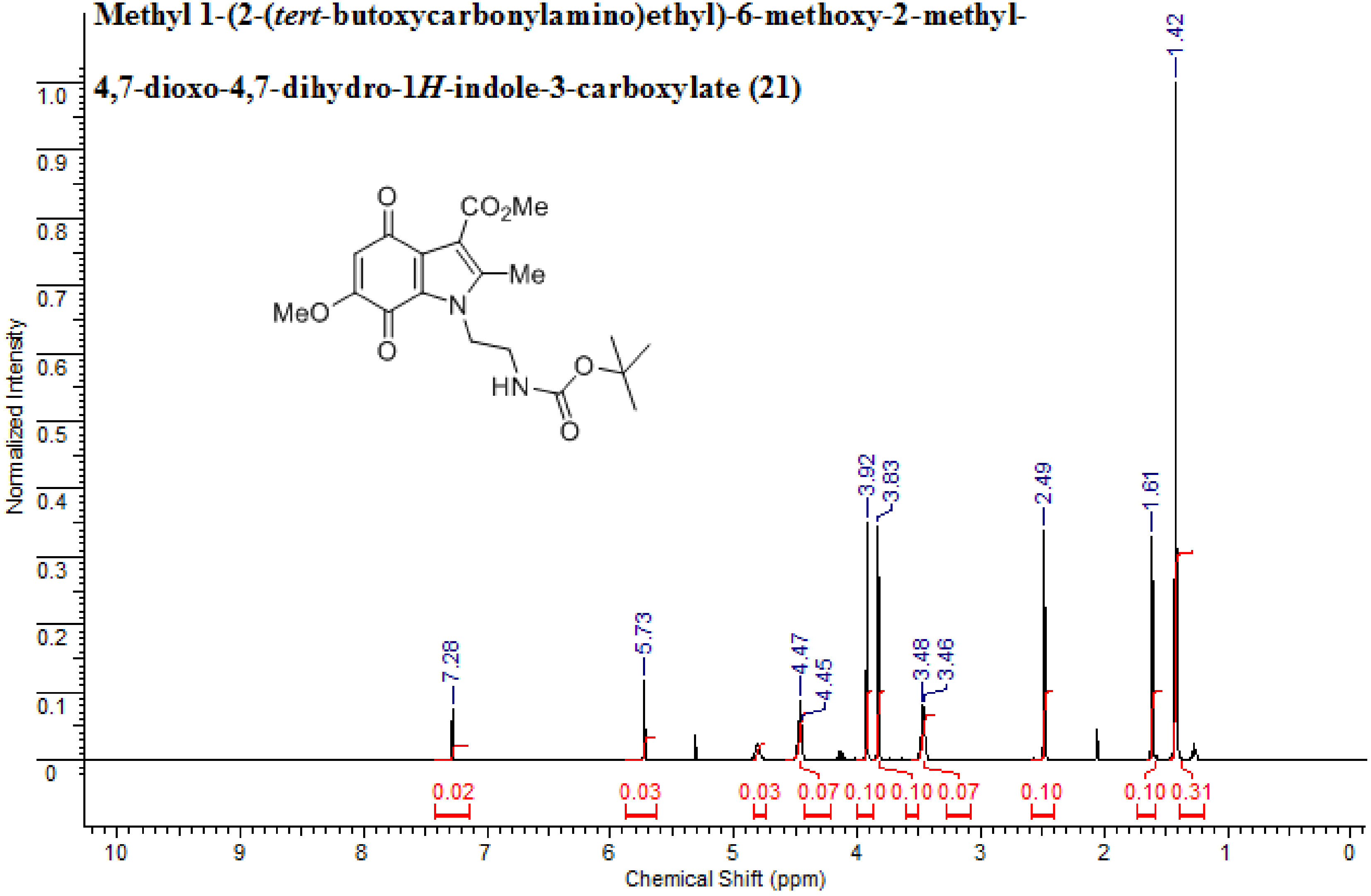

Methyl 1-(2-(*tert*-butoxycarbonylamino)ethyl)-6-methoxy-2-methyl-4,7-dioxo-4,7-dihydro-1*H*-indole-3-carboxylate (21)

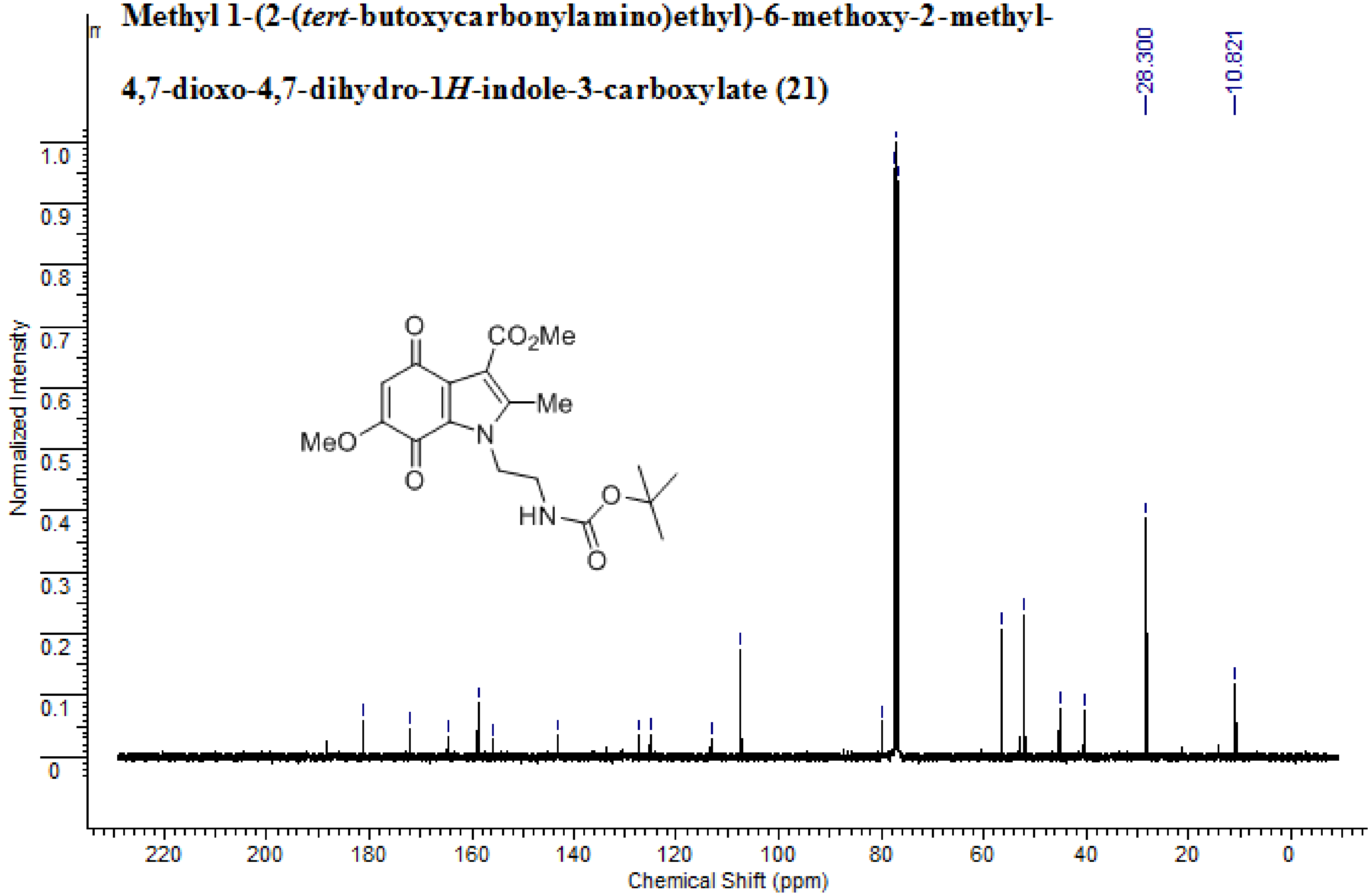

Ethyl 6-methoxy-2-(4-methoxybenzyloxy)methyl-1-methyl-4,7-dioxo-4,7-dihydro-1*H*-indole-3-carboxylate (22)

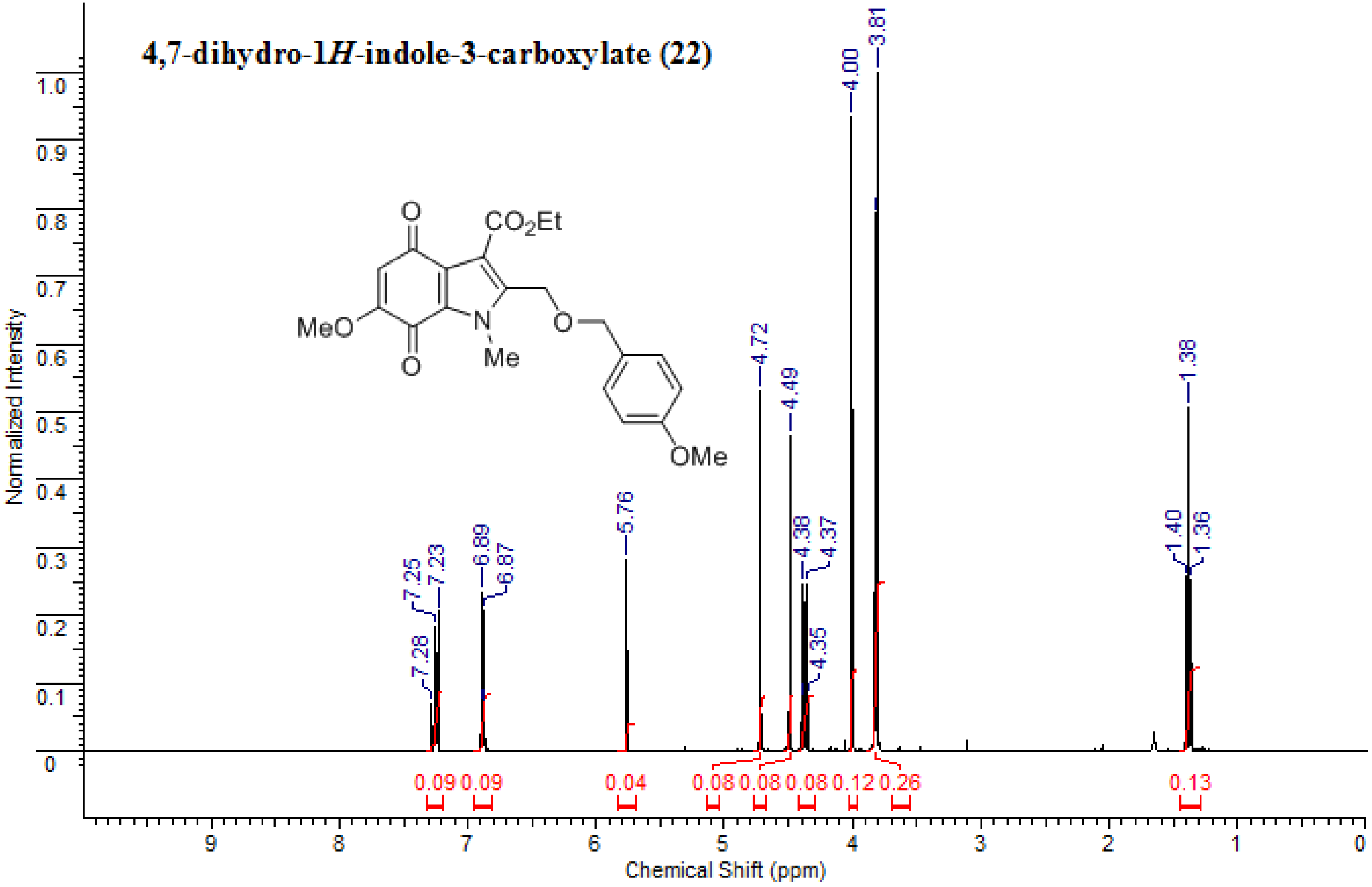

Ethyl 6-methoxy-2-(4-methoxybenzyloxy)methyl-1-methyl-4,7-dioxo-4,7-dihydro-1*H*-indole-3-carboxylate (22)

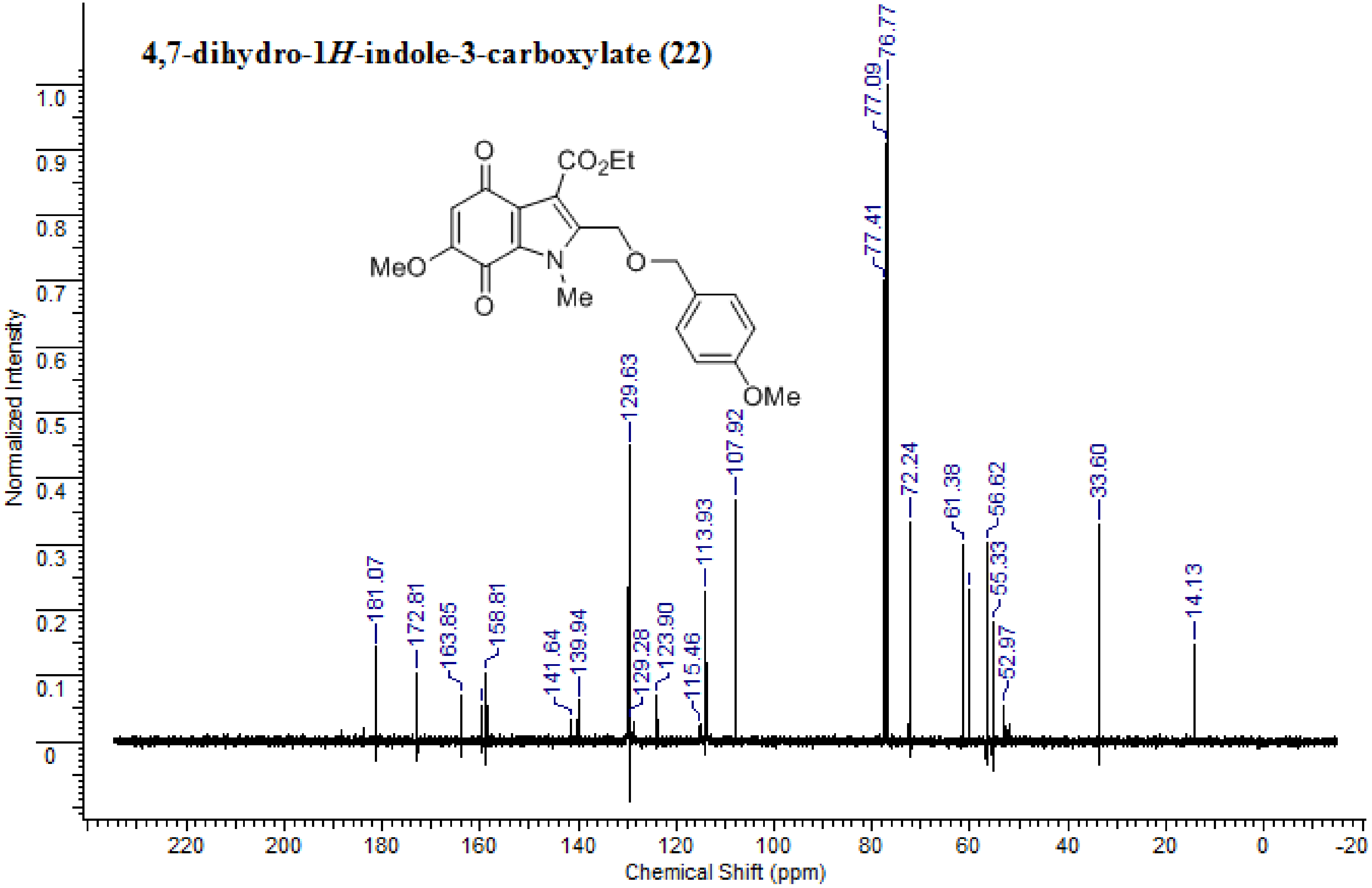

Methyl 6-ethyl-1,2-dimethyl-4,7-dioxo-4,7-dihydro-1*H*-indole-3-carboxylate (23)

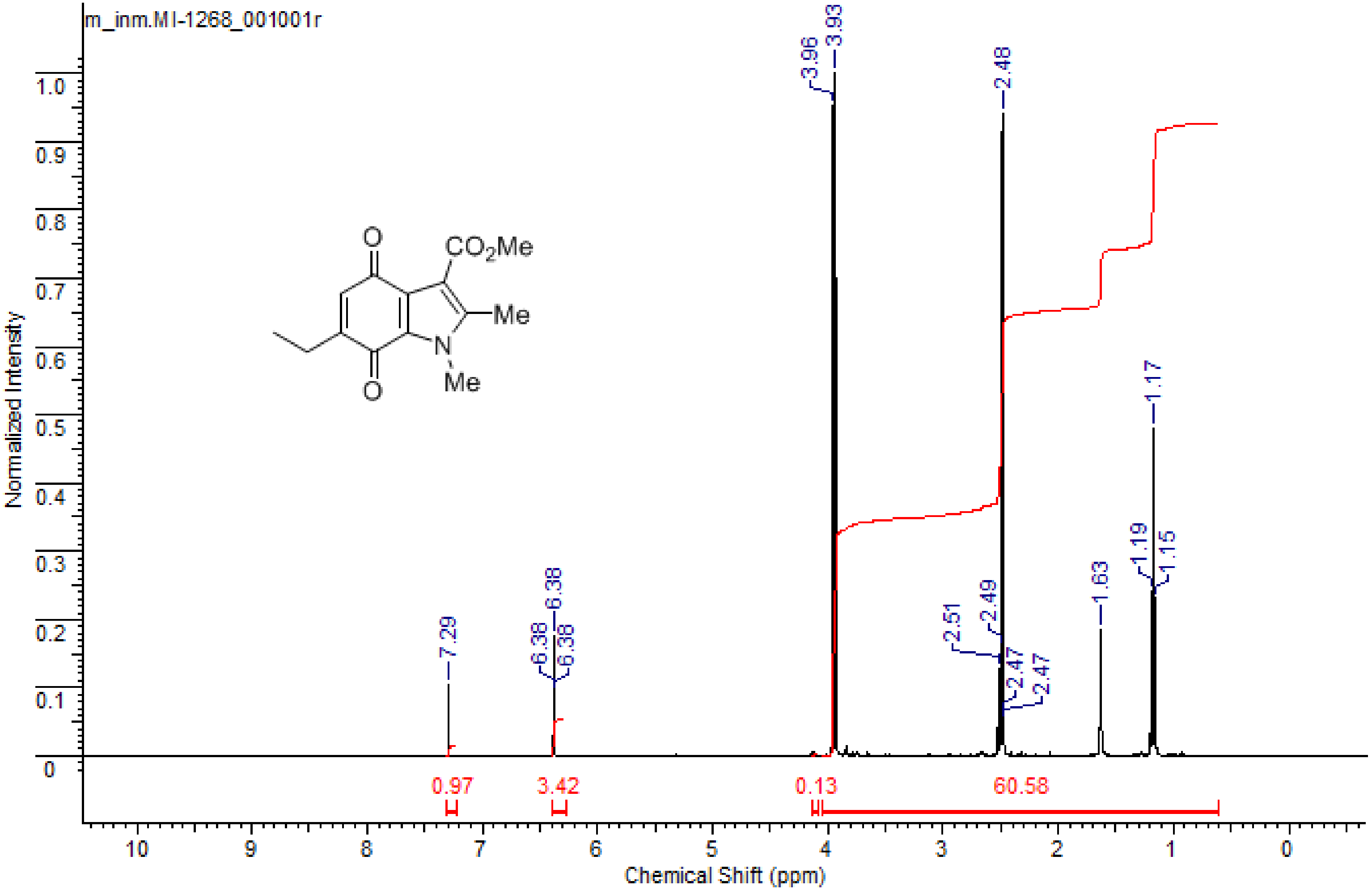

Methyl 6-ethyl-1,2-dimethyl-4,7-dioxo-4,7-dihydro-1*H*-indole-3-carboxylate (23)

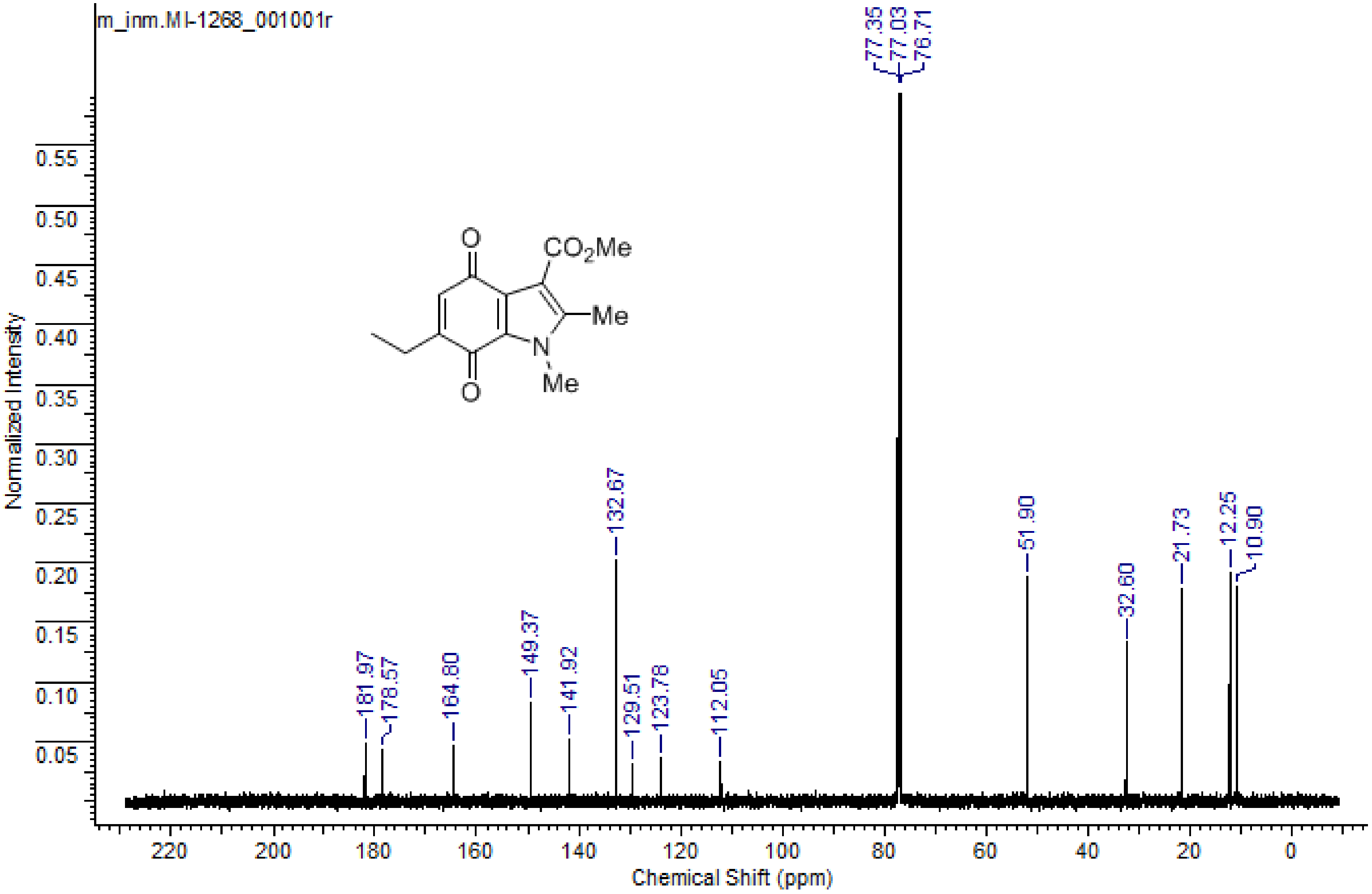

Methyl 6-isopropoxy-1,2-dimethyl-4,7-dioxo-4,7-dihydro-1*H*-indole-3-carboxylate (24)

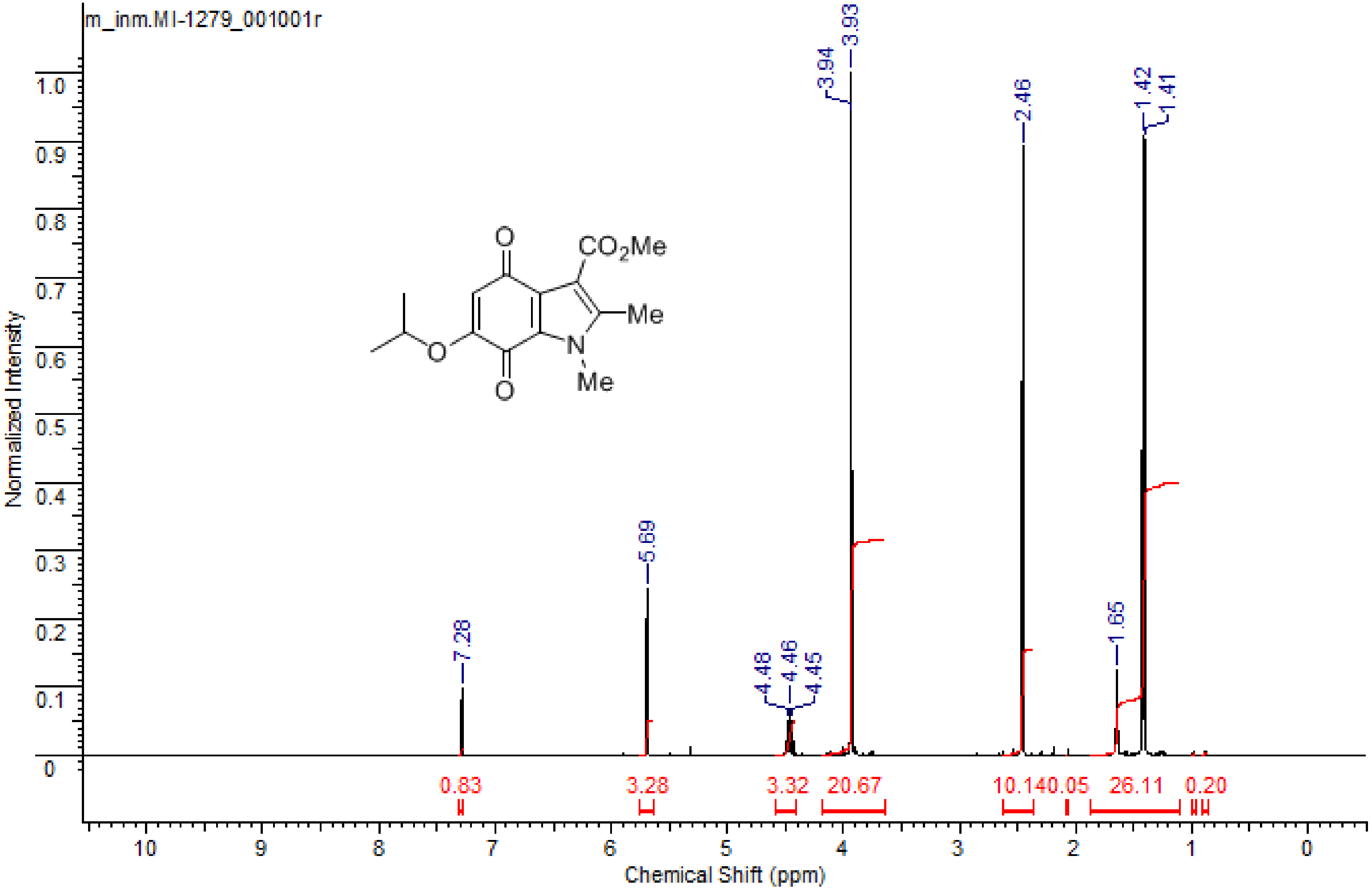

Methyl 6-isopropoxy-1,2-dimethyl-4,7-dioxo-4,7-dihydro-1*H*-indole-3-carboxylate (24)

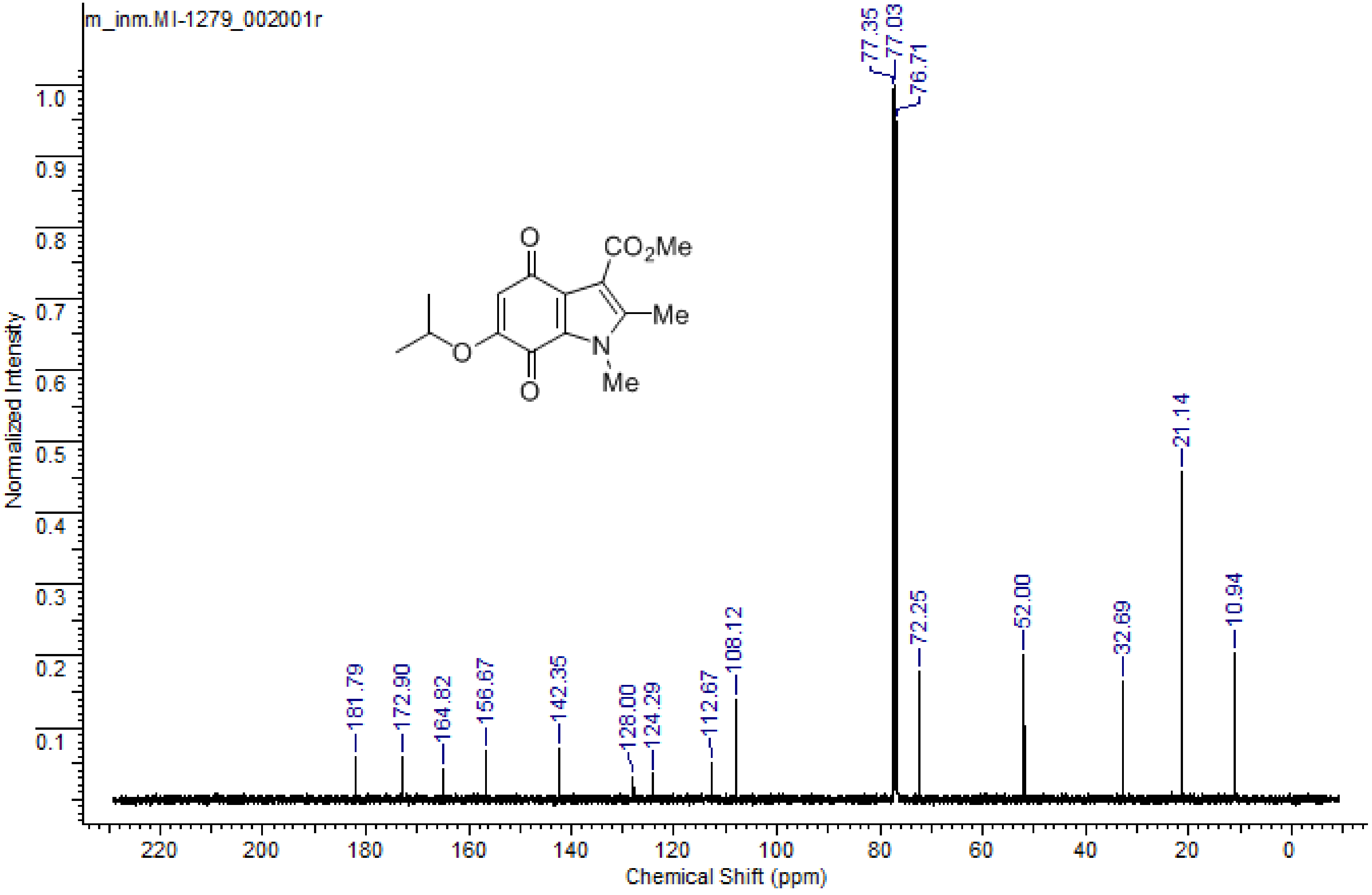

Methyl 6,7-dimethyl-5,9-dioxo-3,5,6,9-tetrahydro-2*H*-[1,4]dioxino[2,3-f]indole-8-carboxylate (25)

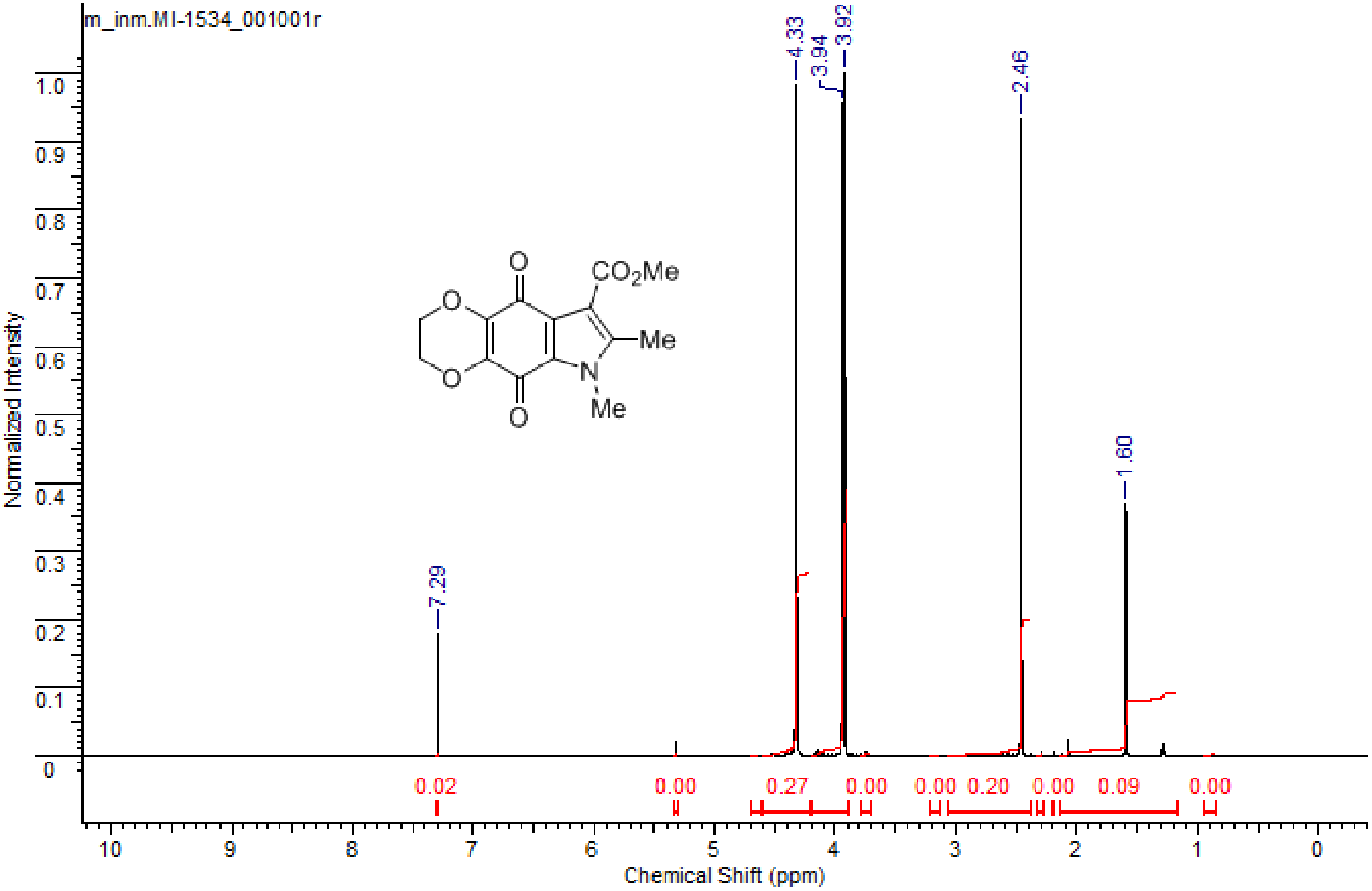

Methyl 6,7-dimethyl-5,9-dioxo-3,5,6,9-tetrahydro-2*H*-[1,4]dioxino[2,3-f]indole-8-carboxylate (25)

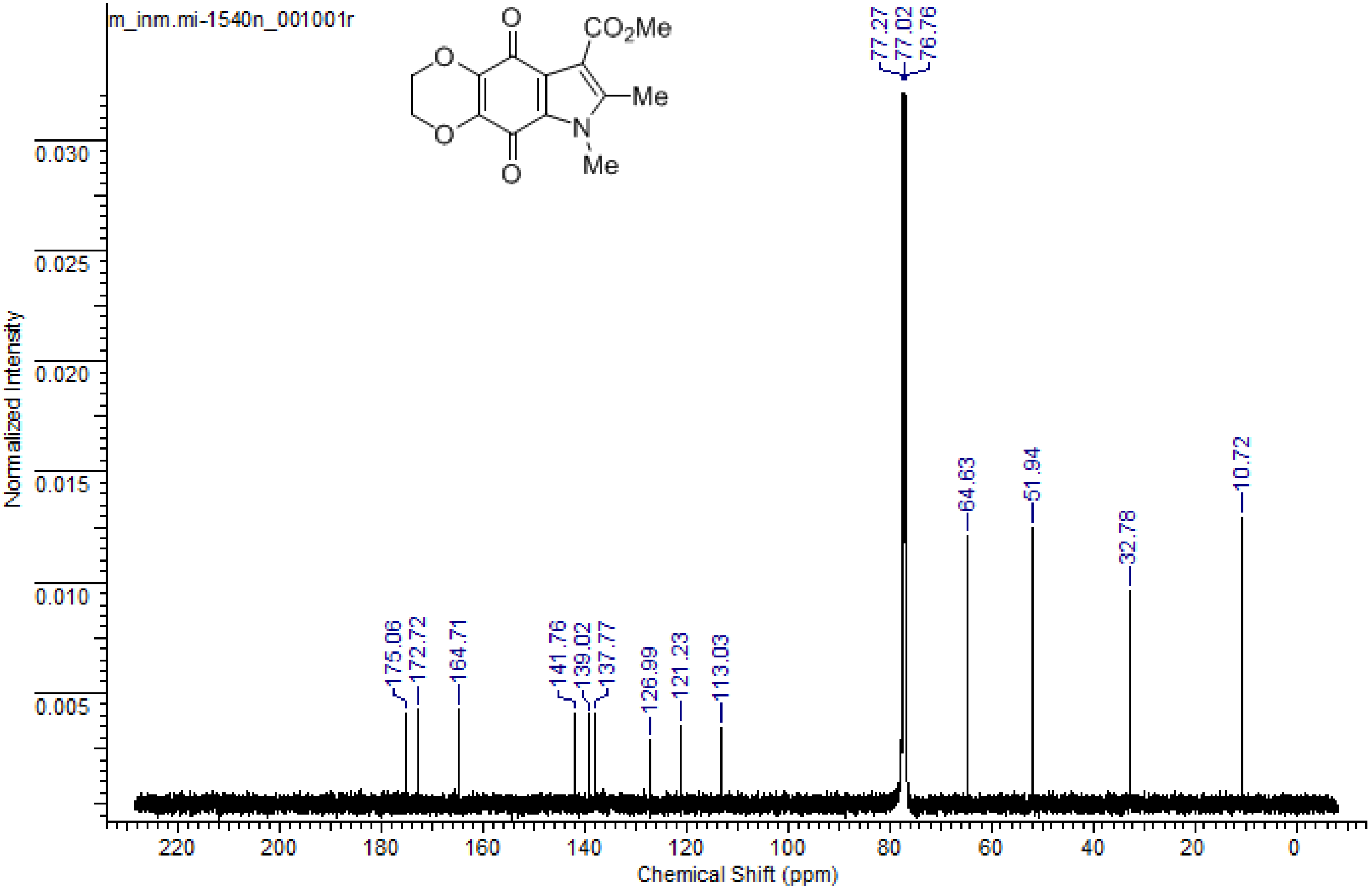

2-Methoxy-9-methyl-7,8-dihydro-1*H*-carbazole-1,4,5(6*H*,9*H*)-trione (26)

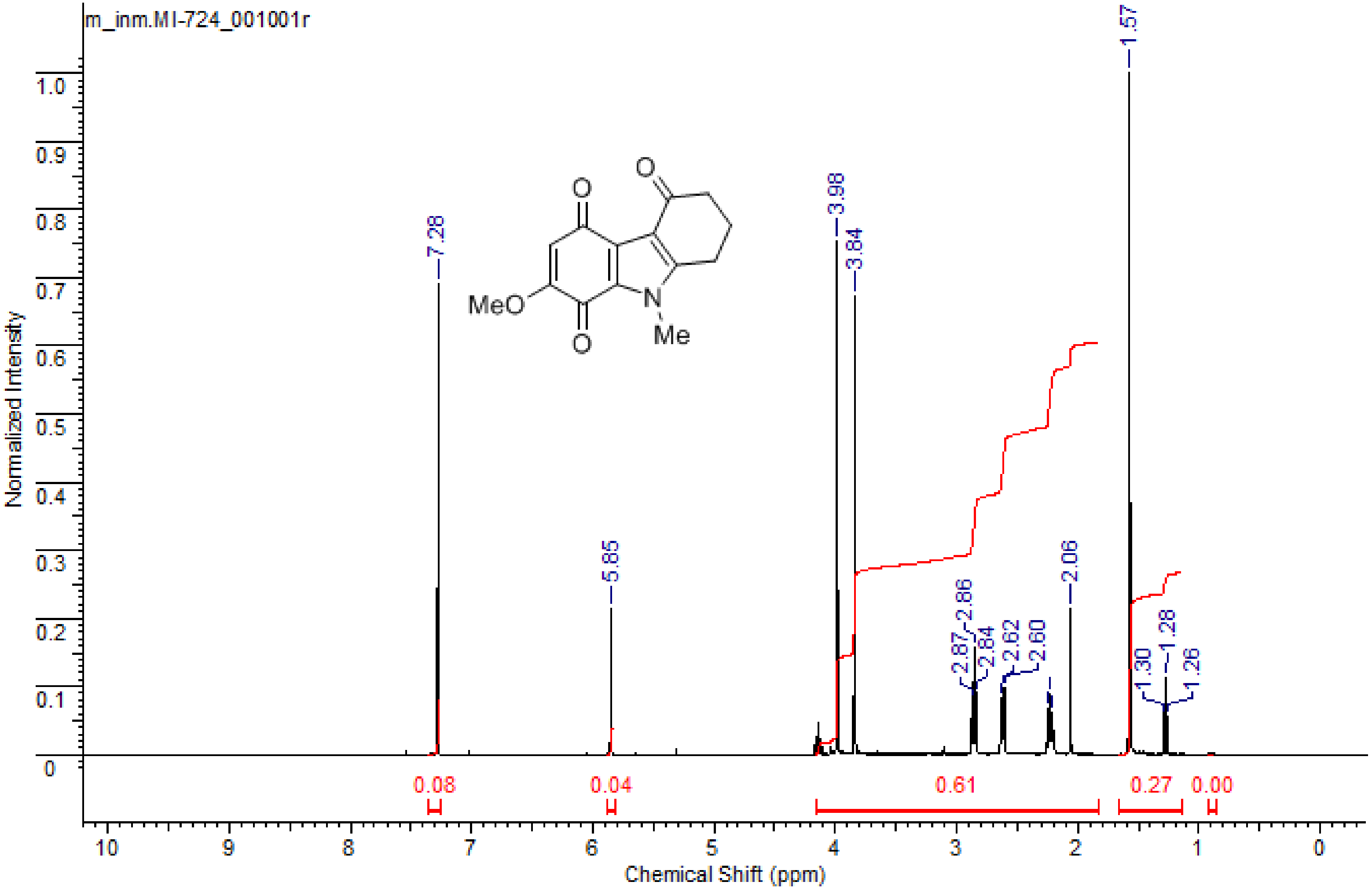

2-Methoxy-9-methyl-7,8-dihydro-1*H*-carbazole-1,4,5(6*H*,9*H*)-trione (26)

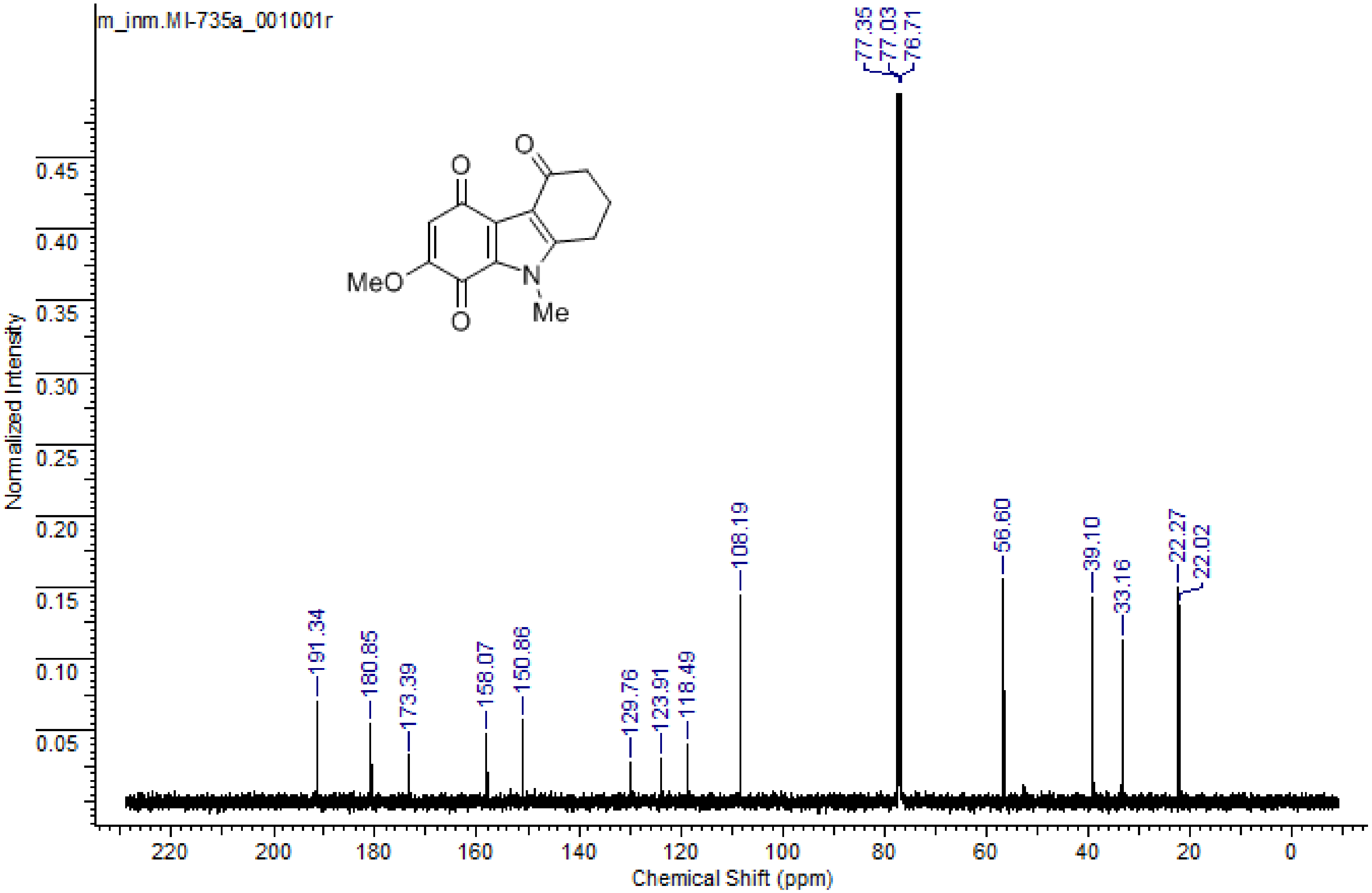

(S)-tert-Butyl 6-methoxy-1-(1-methoxy-4-methyl-1-oxopentan-2-yl)-2-methyl-4,7-dioxo-4,7-dihydro-1*H*-indole-3-carboxylate (27)

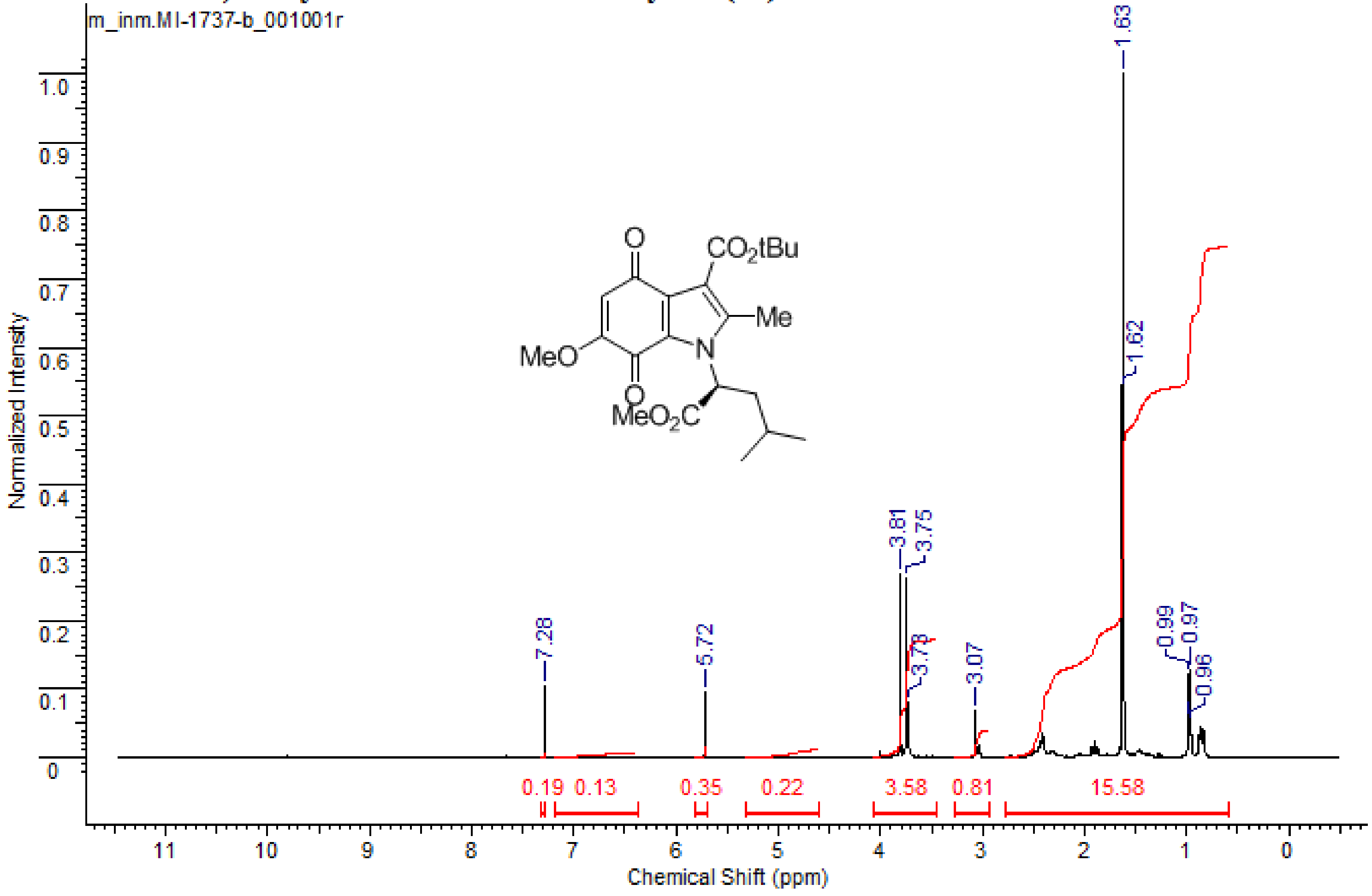

(S)-tert-Butyl 6-methoxy-1-(1-methoxy-4-methyl-1-oxopentan-2-yl)-2-methyl-4,7-dioxo-4,7-dihydro-1*H*-indole-3-carboxylate (27)

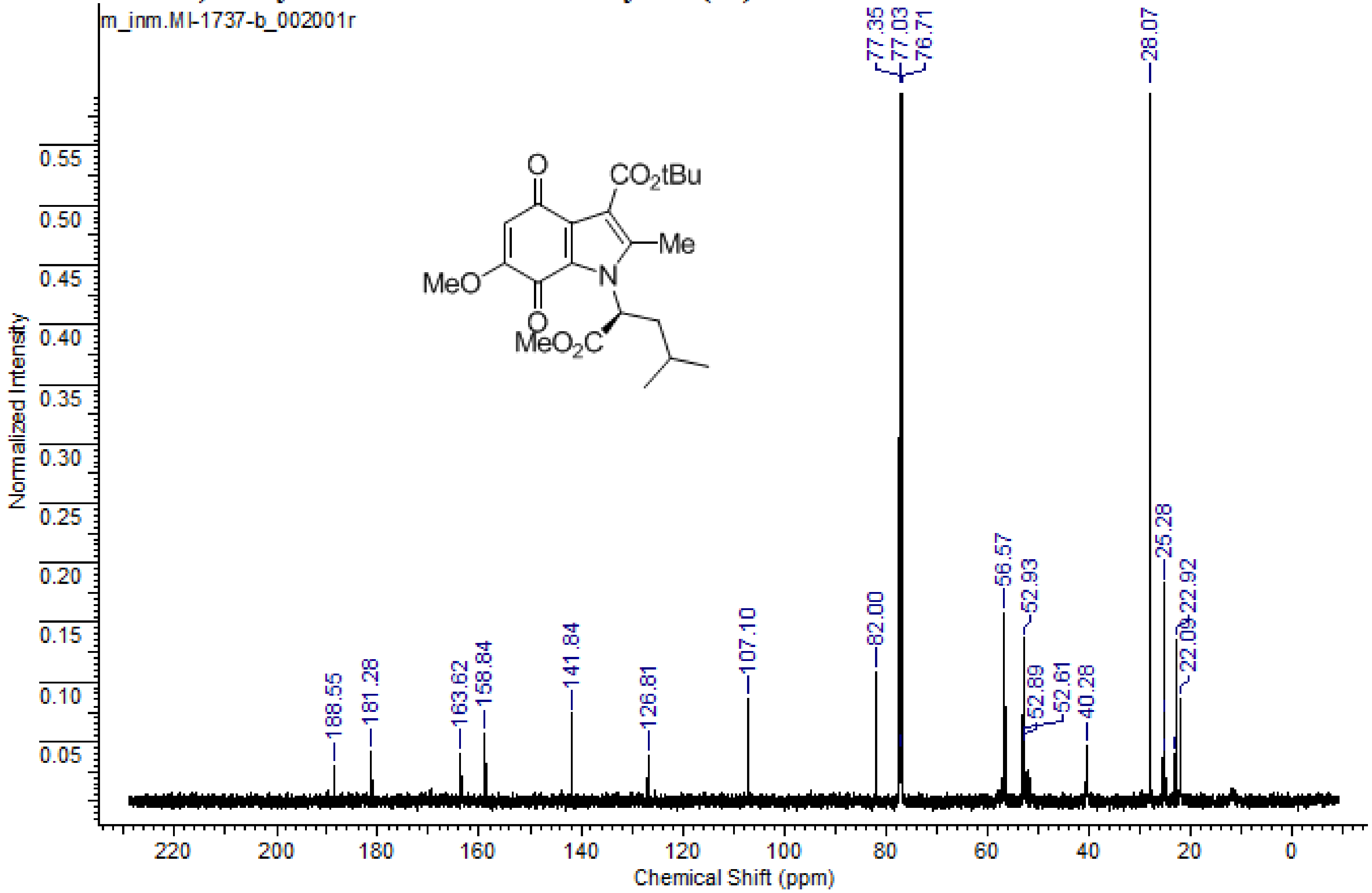

(±)-Menthyl 1-(2-(1*H*-indol-3-yl)ethyl)-6-methoxy-2-methyl-4,7-dioxo-4,7-dihydro-1*H*-indole-3-carboxylate (28)

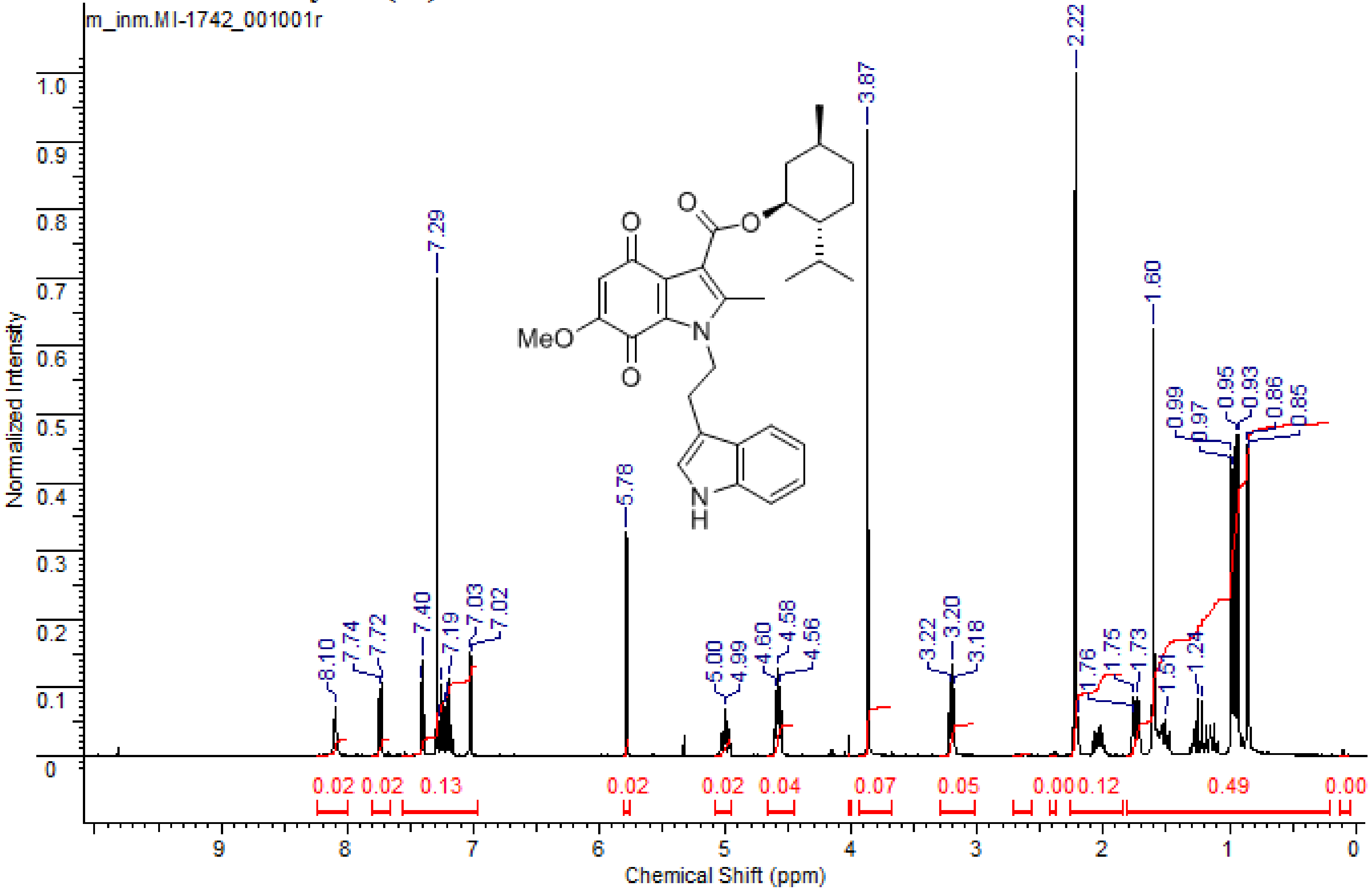

(±)-Menthyl 1-(2-(1*H*-indol-3-yl)ethyl)-6-methoxy-2-methyl-4,7-dioxo-4,7-dihydro-1*H*-indole-3-carboxylate (28)

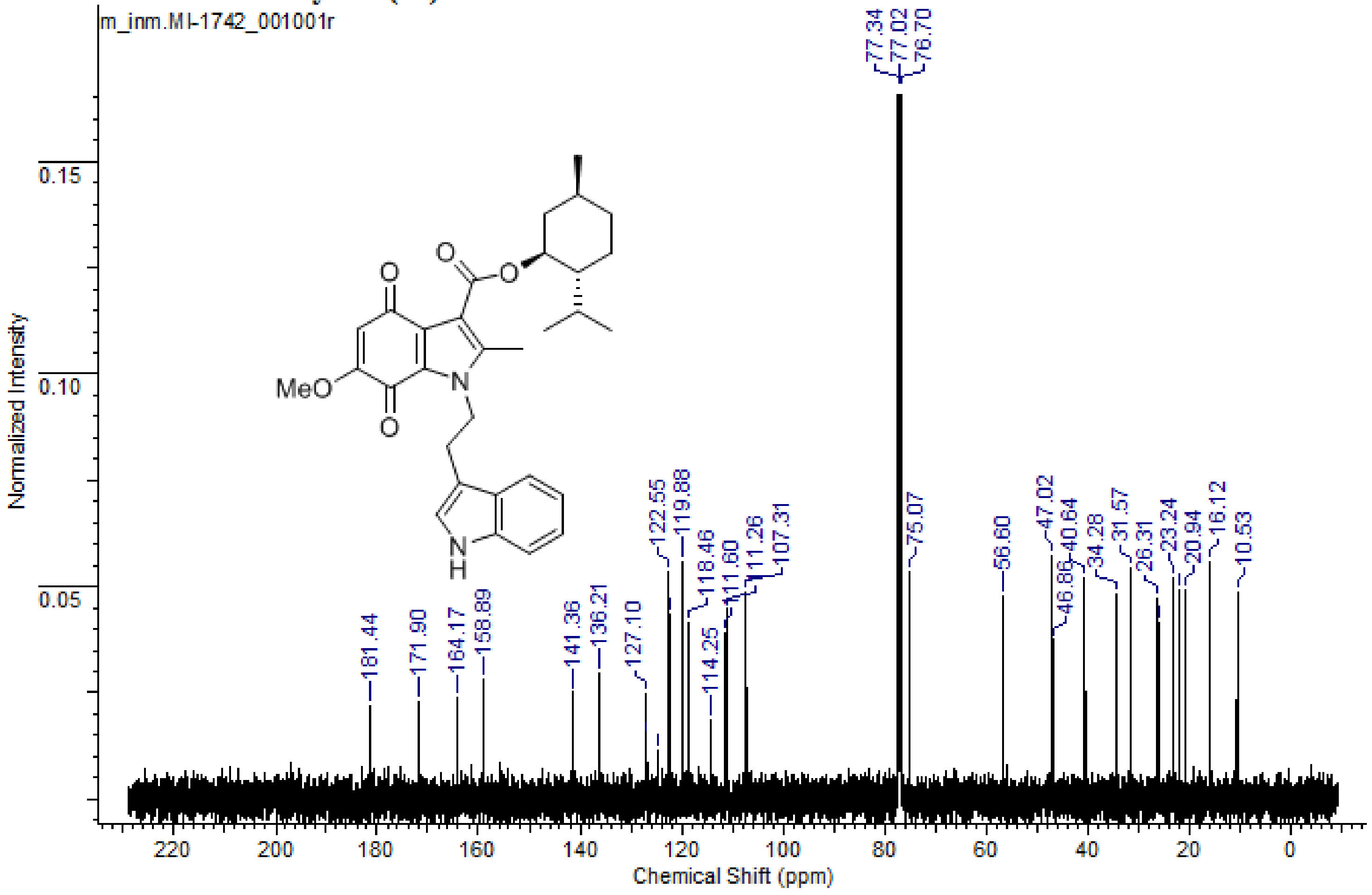

6-Methoxy-1-(4-methoxybenzyl)-2-methyl-3-(morpholine-4-carbonyl)-1*H*-indole-4,7-dione (29)

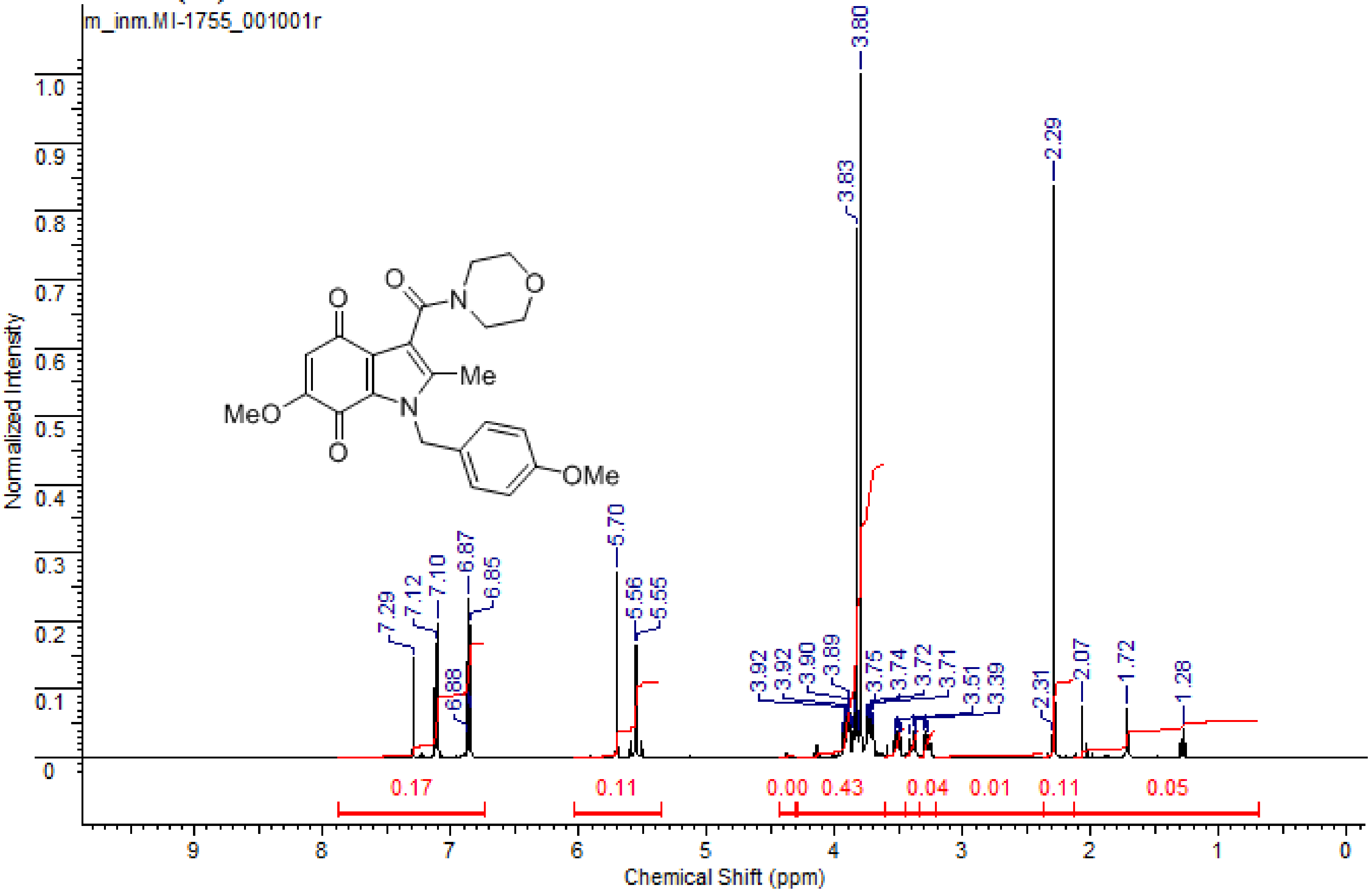

6-Methoxy-1-(4-methoxybenzyl)-2-methyl-3-(morpholine-4-carbonyl)-1*H*-indole-4,7-dione (29)

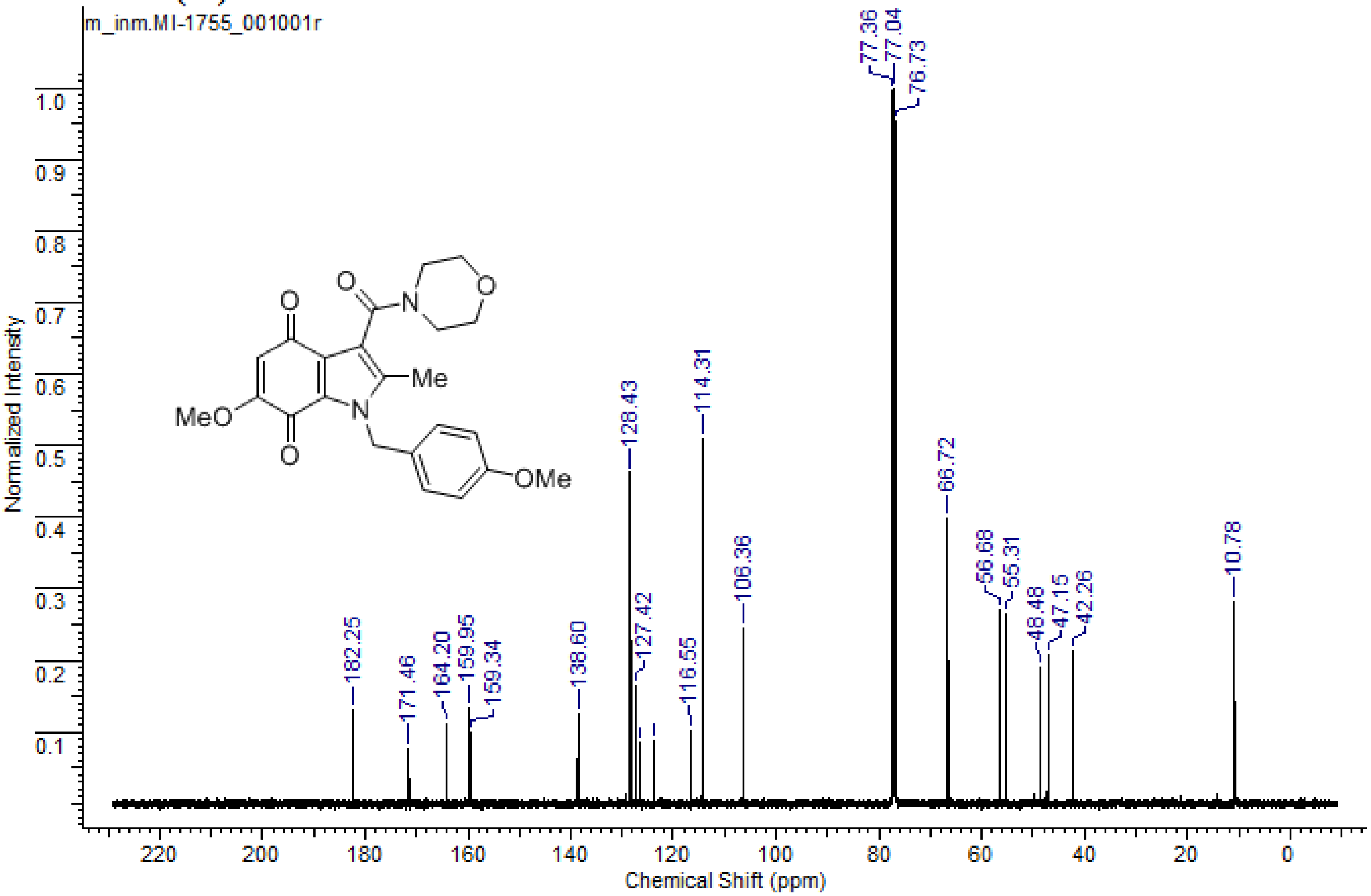

***tert*-Butyl 1-(4-methoxybenzyl)-2-methyl-3-(morpholine-4-carbonyl)-4,7-dioxo-4,7-dihydro-1*H*-indol-6-ylcarbamate (30)**

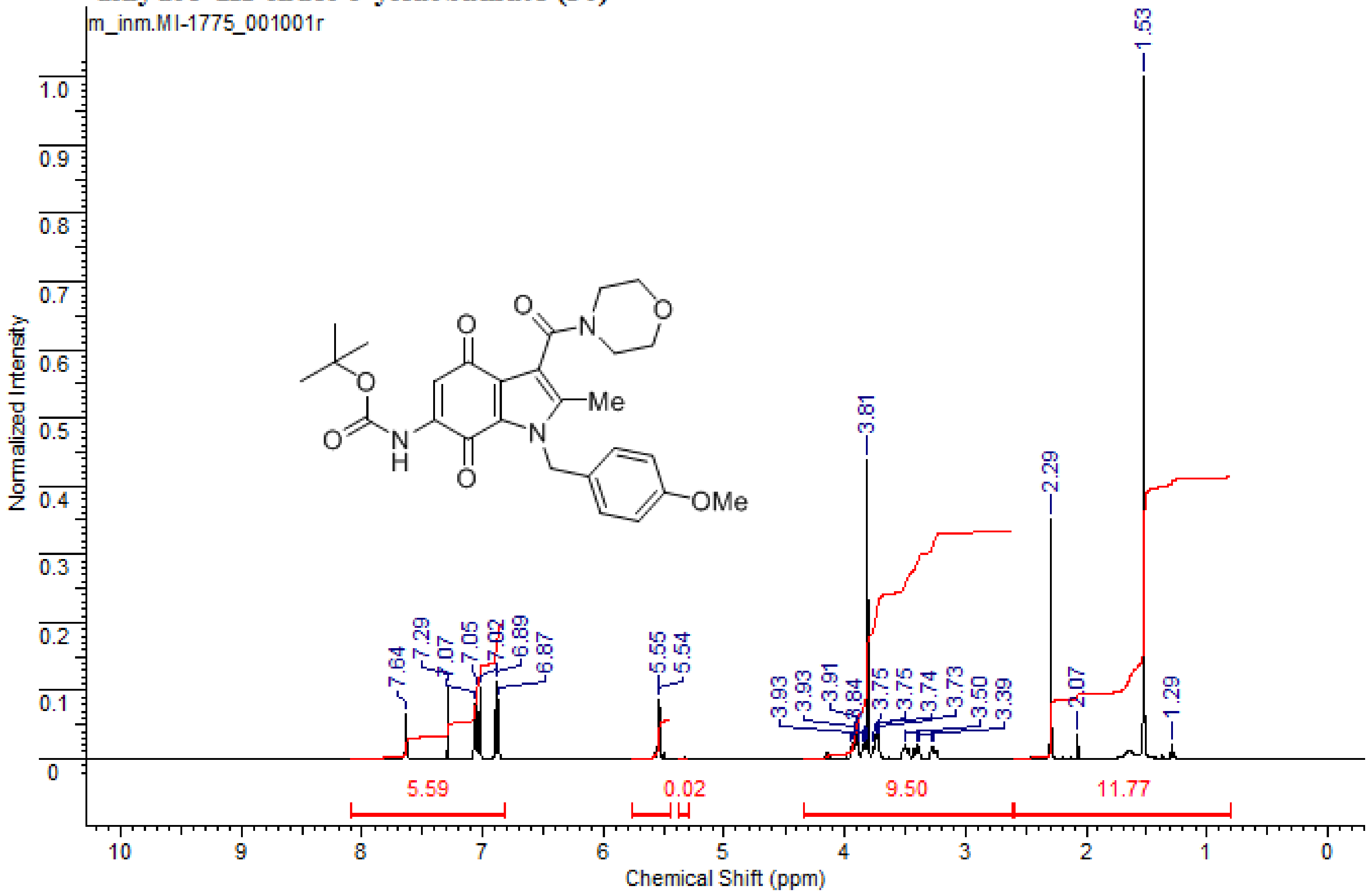

tert-Butyl 1-(4-methoxybenzyl)-2-methyl-3-(morpholine-4-carbonyl)-4,7-dioxo-4,7-dihydro-1_H_-indol-6-ylcarbamate (30)

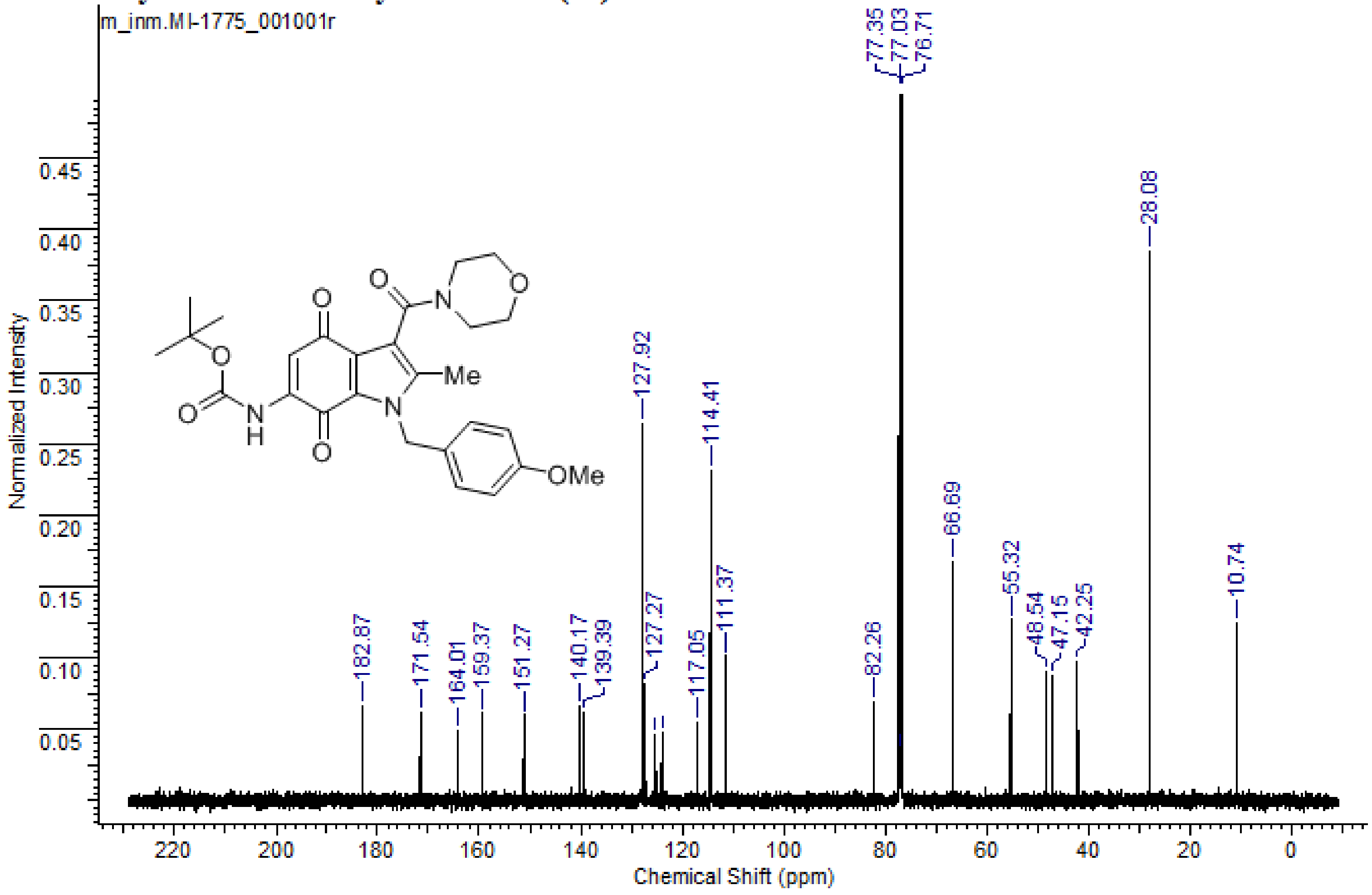

Methyl 1-(2,2-diethoxyethyl)-6-methoxy-4,7-dioxo-2-phenyl-4,7-dihydro-1*H*-indole-3-carboxylate (31)

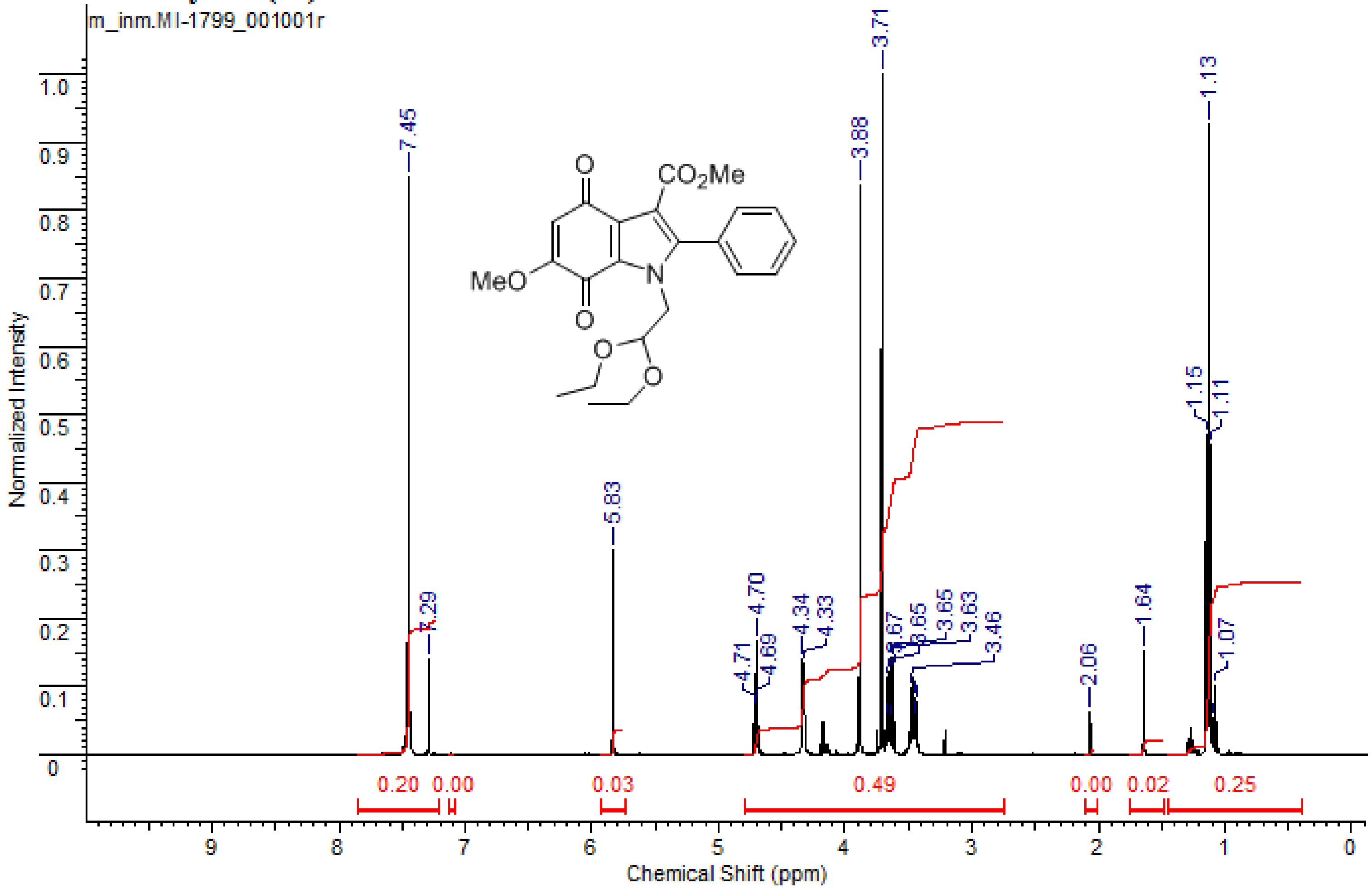

Methyl 1-(2,2-diethoxyethyl)-6-methoxy-4,7-dioxo-2-phenyl-4,7-dihydro-1*H*-indole-3-carboxylate (31)

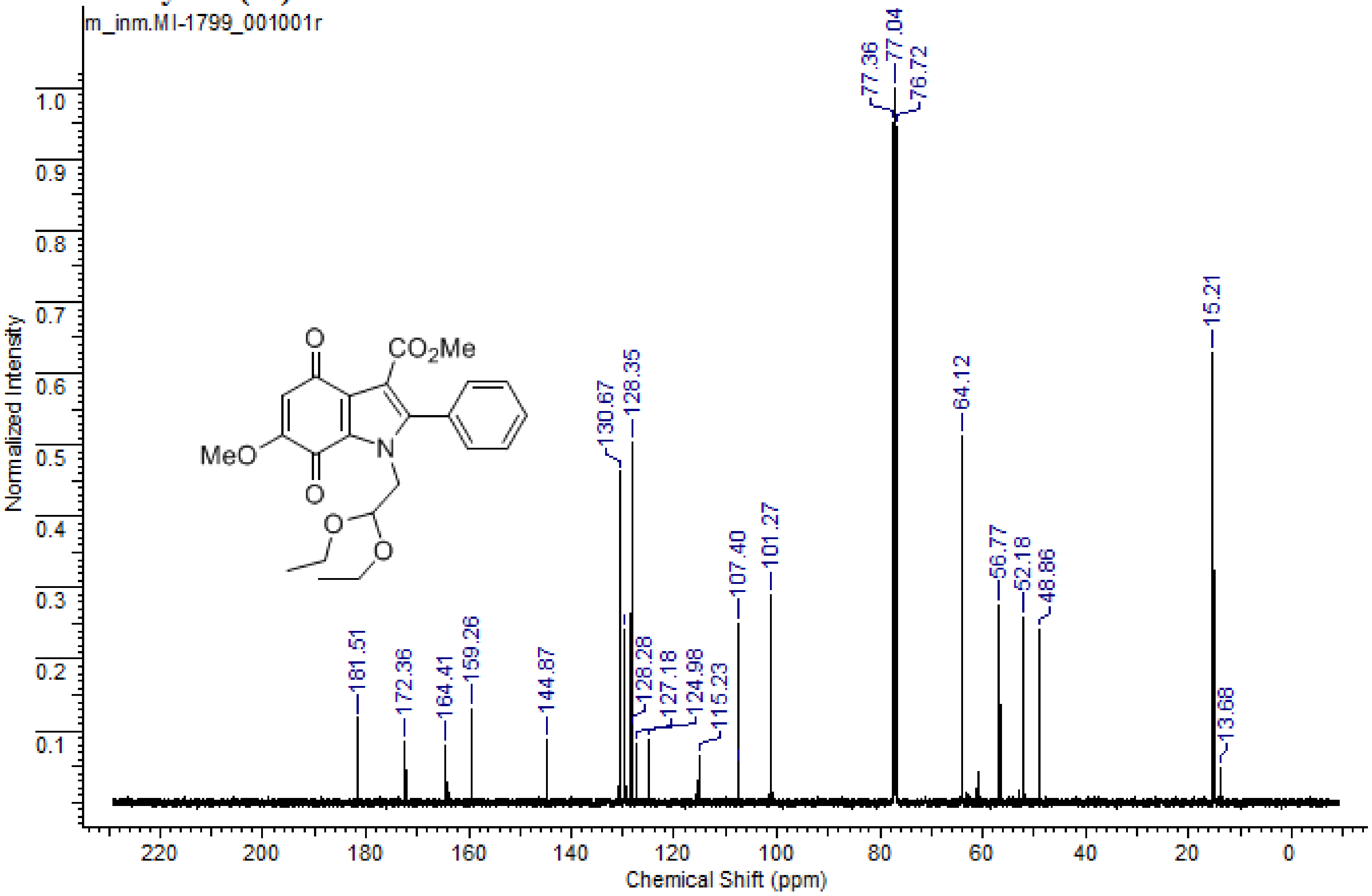

tert-Butyl 1-(2-(1*H*-indol-3-yl)ethyl)-6-isopropoxy-2-methyl-4,7-dioxo-4,7-dihydro-1*H*-indole-3-carboxylate (32)

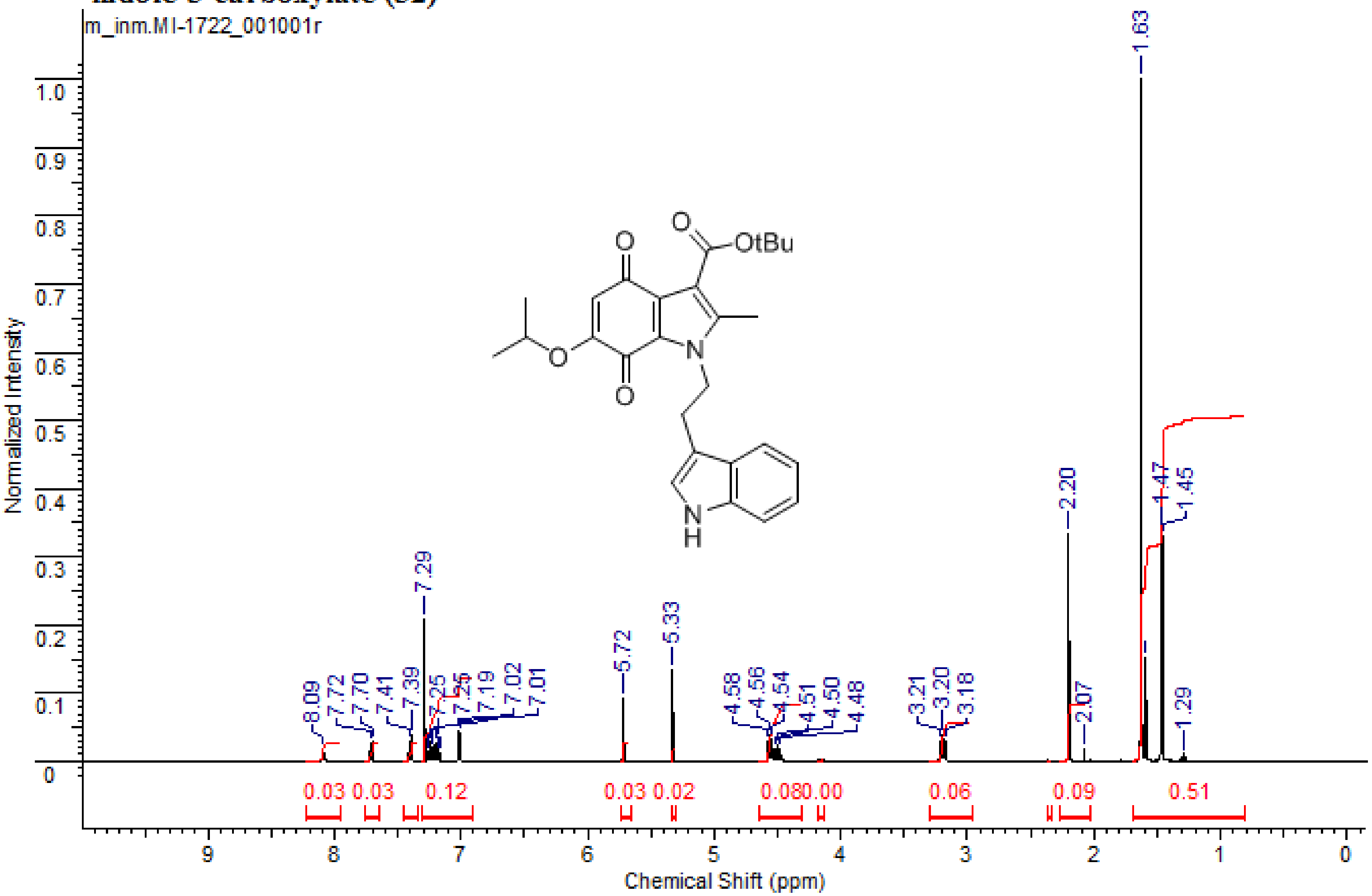

tert-Butyl 1-(2-(1*H*-indol-3-yl)ethyl)-6-isopropoxy-2-methyl-4,7-dioxo-4,7-dihydro-1*H*-indole-3-carboxylate (32)

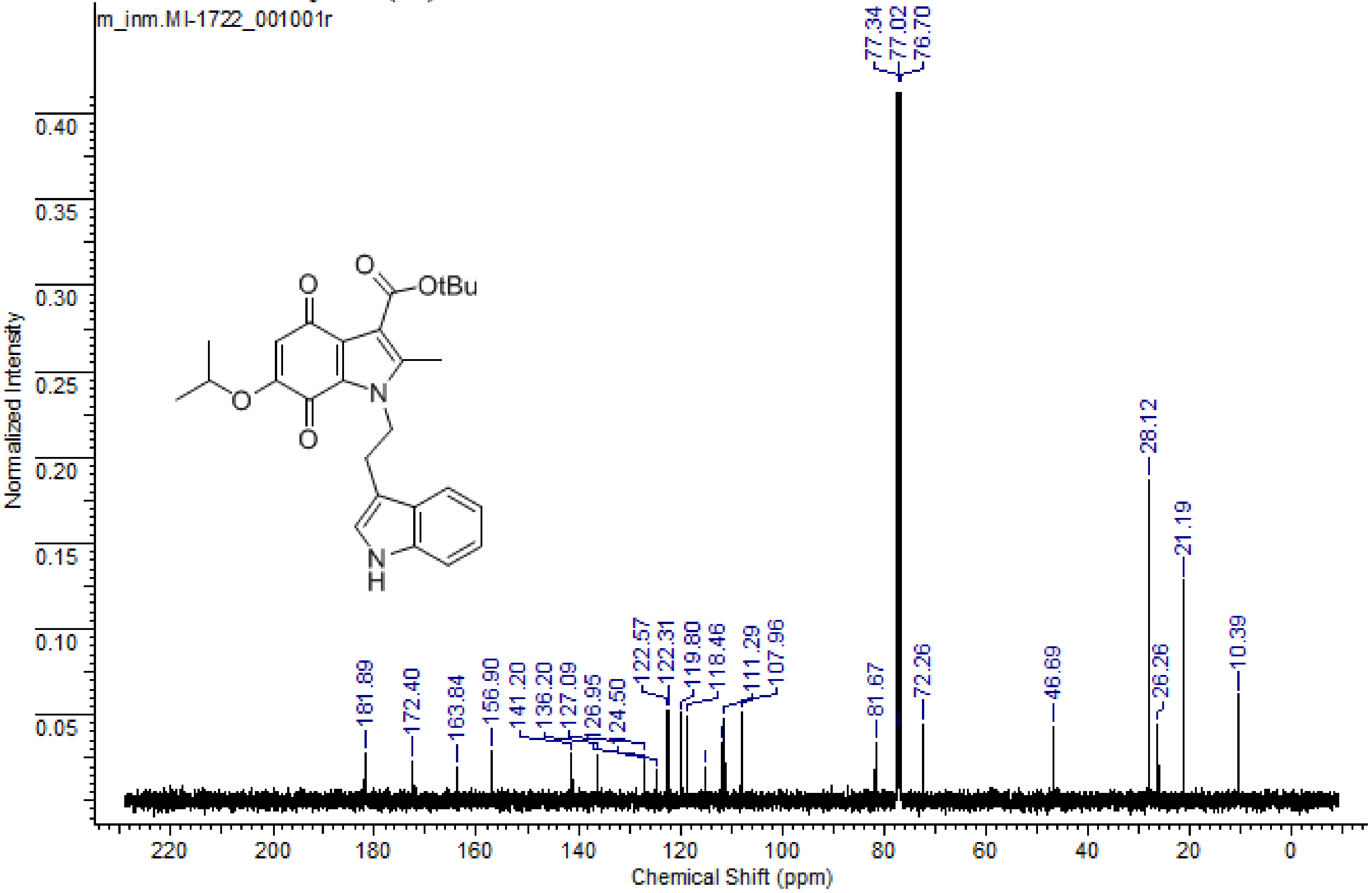

Methyl 1-(2-*tert*-butoxy-2-oxoethyl)-6-methoxy-2-methyl-4,7-dioxo-4,7-dihydro-1*H*-indole-3-carboxylate (33)

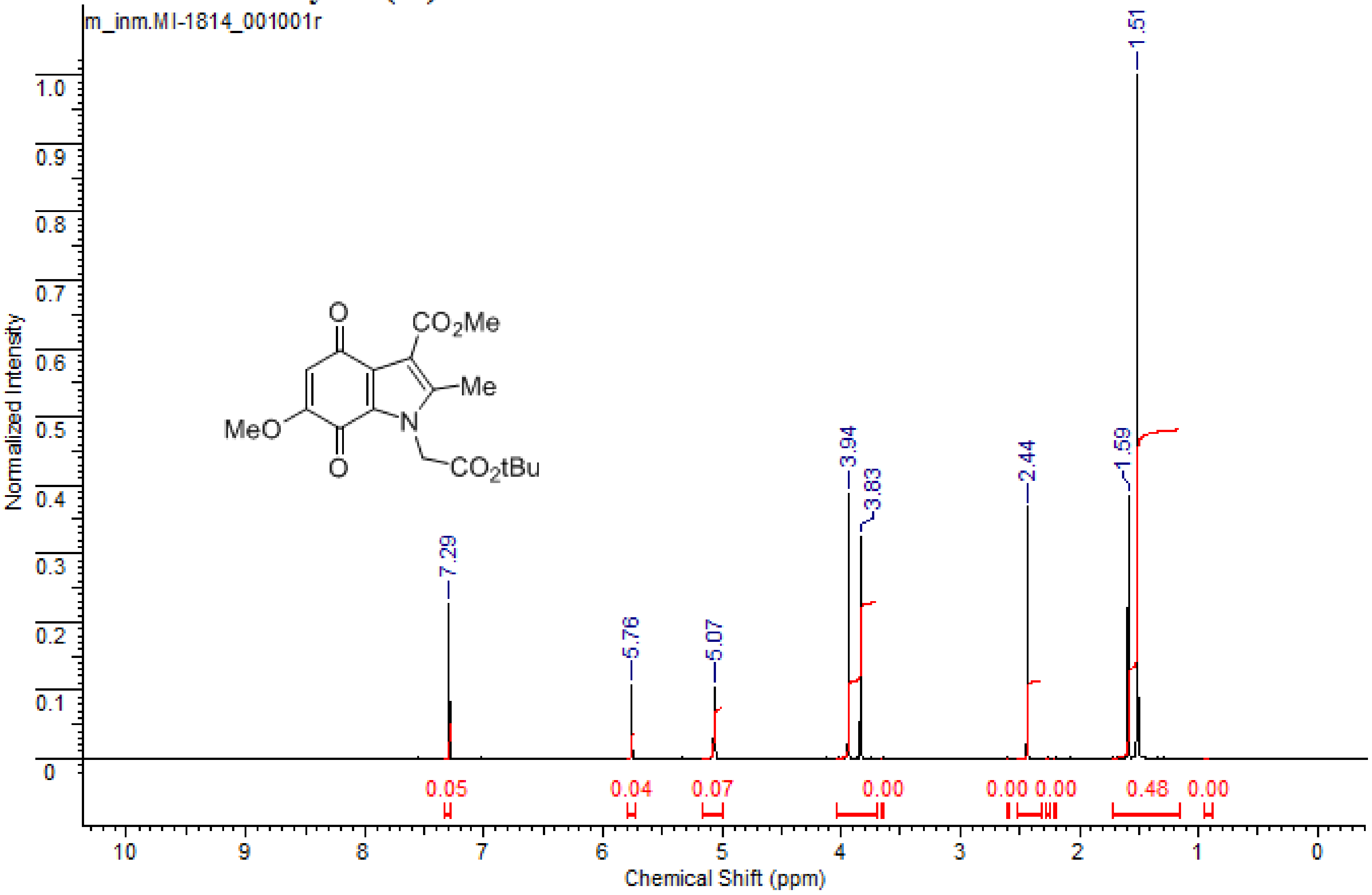

Methyl 1-(2-*tert*-butoxy-2-oxoethyl)-6-methoxy-2-methyl-4,7-dioxo-4,7-dihydro-1*H*-indole-3-carboxylate (33)

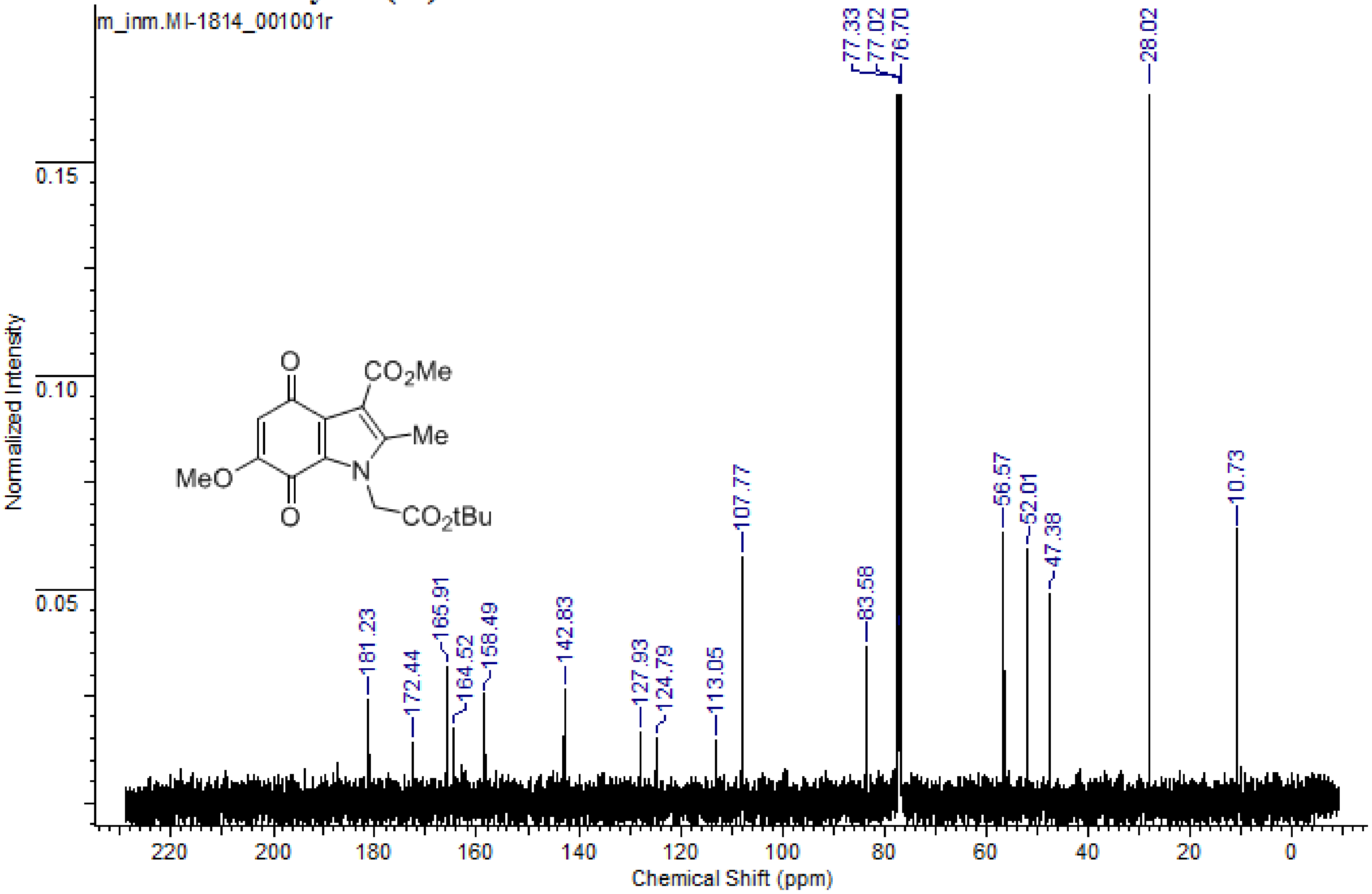

Methyl 1-cyclohexyl-2-ethyl-6-isopropoxy-4,7-dioxo-4,7-dihydro-1*H*-indole-3-carboxylate (34)

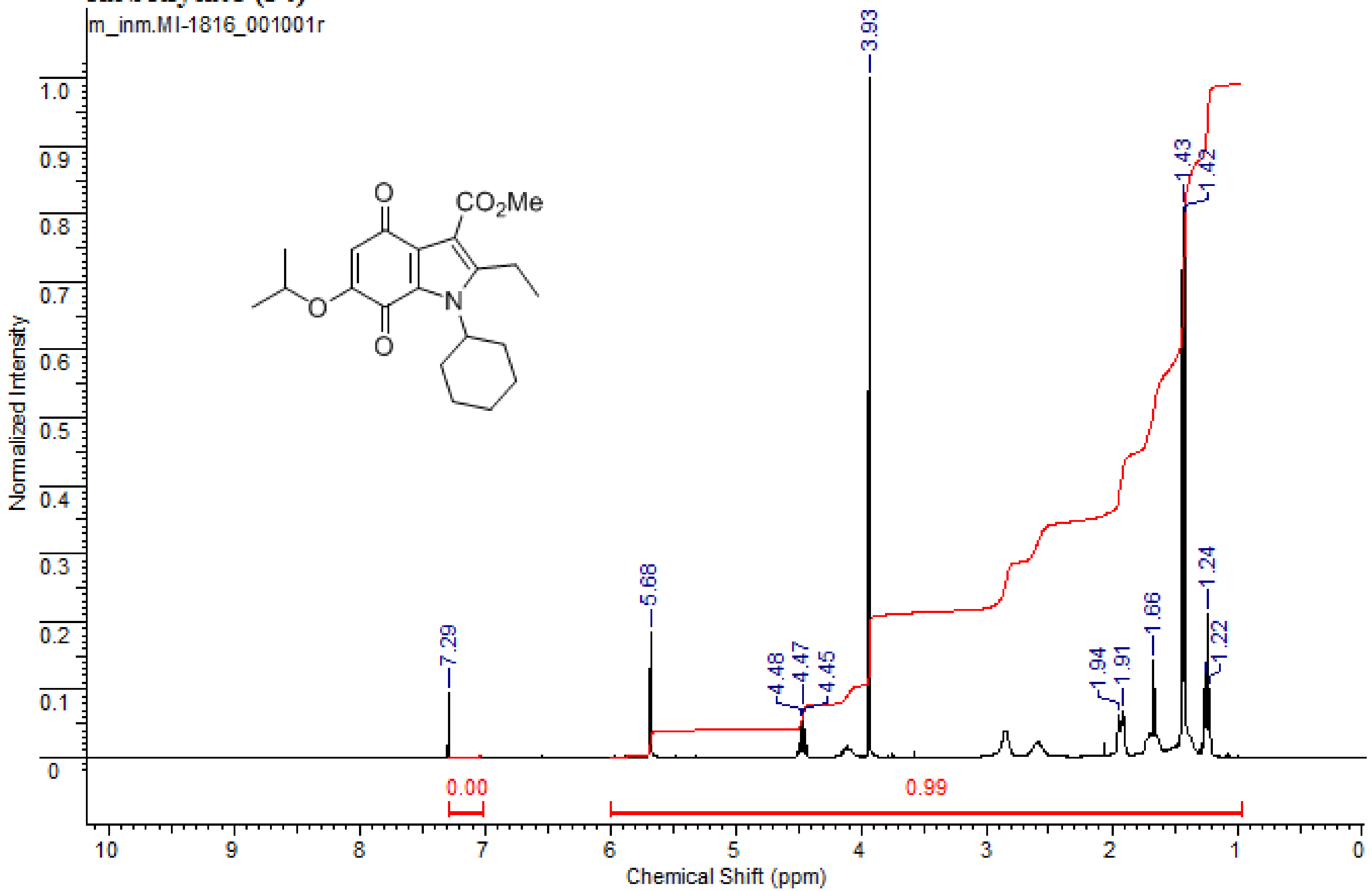

Methyl 1-cyclohexyl-2-ethyl-6-isopropoxy-4,7-dioxo-4,7-dihydro-1*H*-indole-3-carboxylate (34)

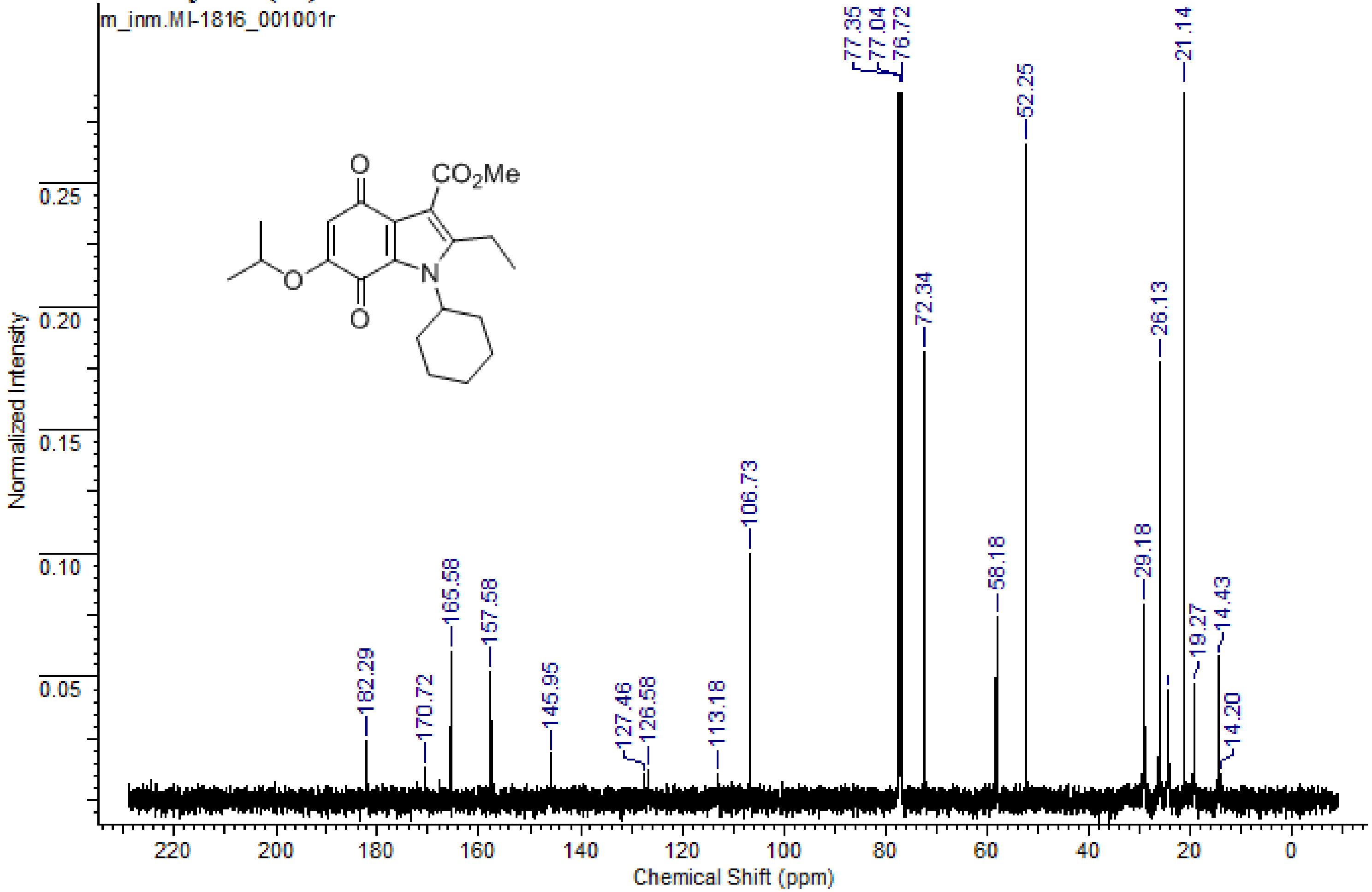

2-Methoxy-7,7-dimethyl-9-(4-nitrophenyl)-7,8-dihydro-1*H*-carbazole-1,4,5(6*H*,9*H*)-trione (35)

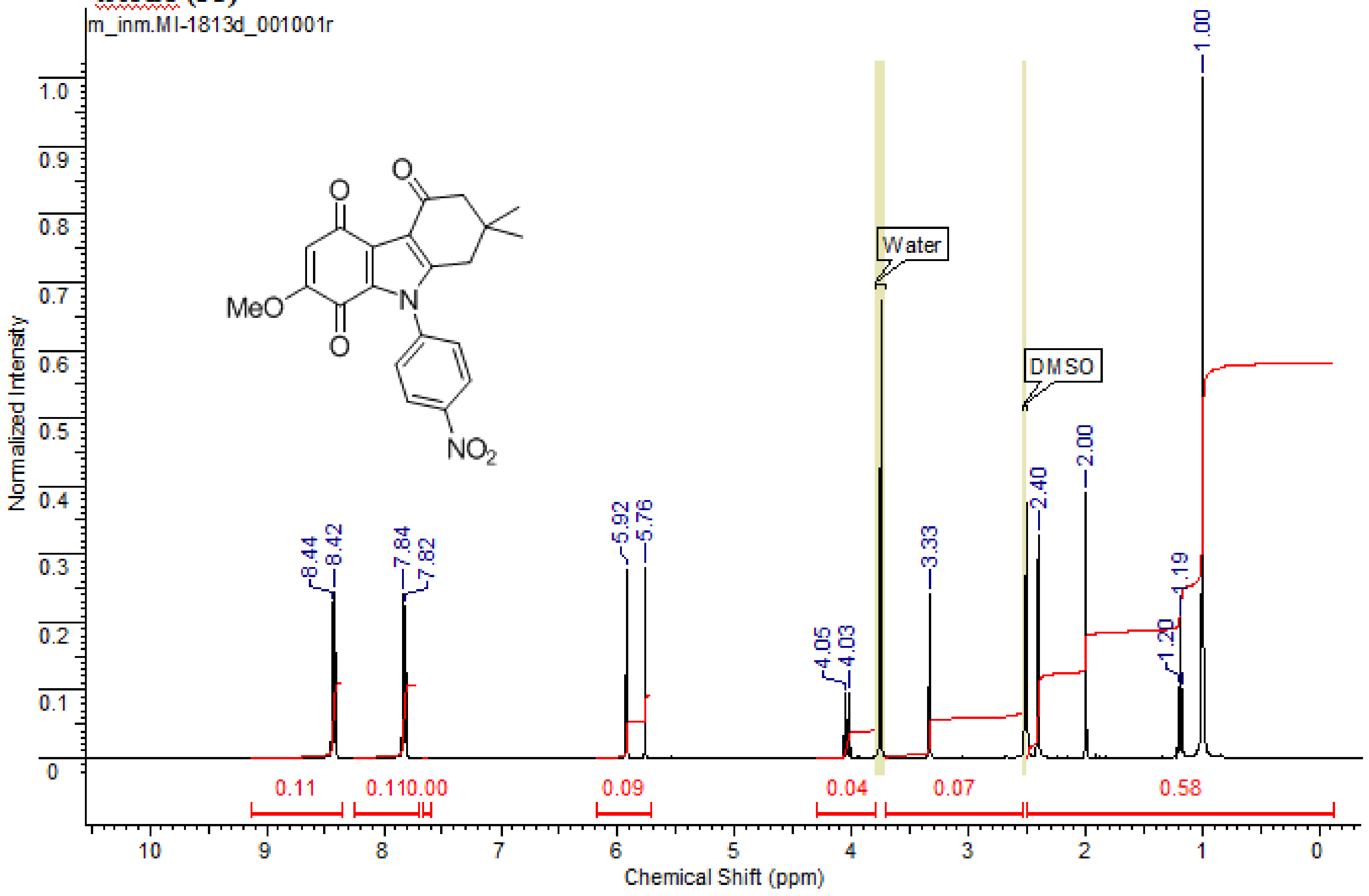

2-Methoxy-7,7-dimethyl-9-(4-nitrophenyl)-7,8-dihydro-1*H*-carbazole-1,4,5(6*H*,9*H*)-trione (35)

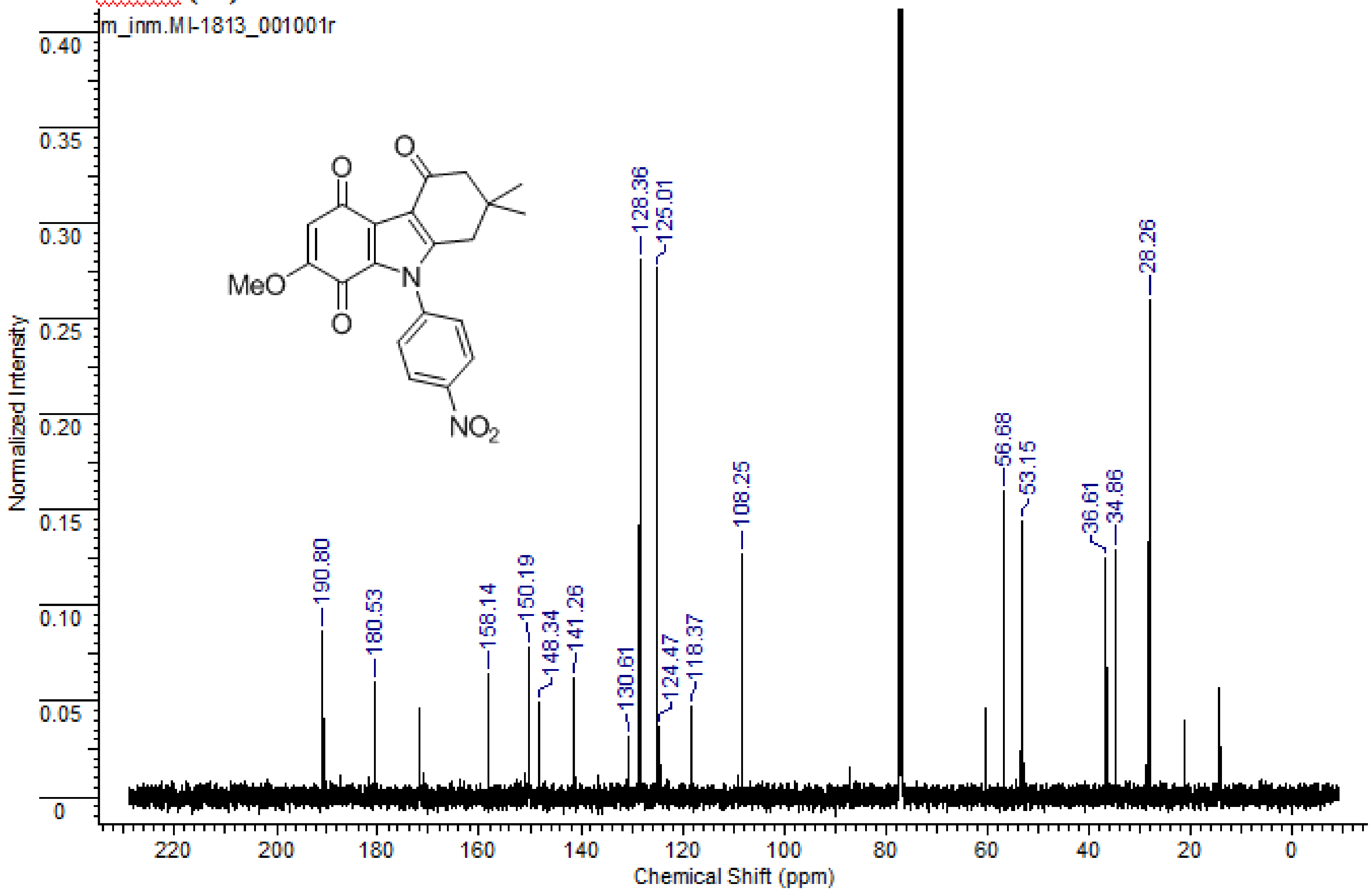

Cholesteryl 1-(2-(dimethylamino)ethyl)-6-methoxy-2-methyl-4,7-dioxo-4,7-dihydro-1*H*-indole-3-carboxylate (36)

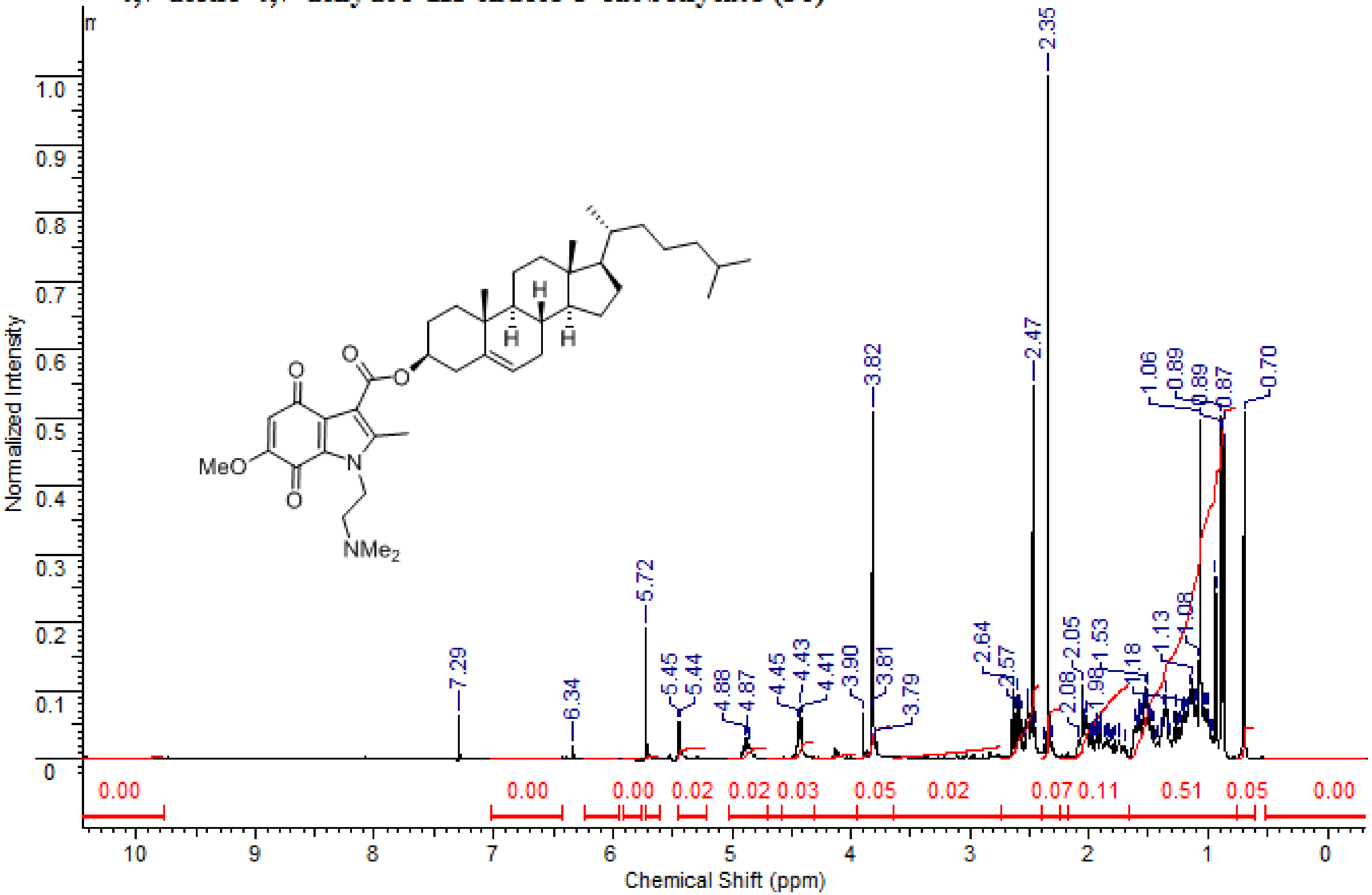

Cholesteryl 1-(2-(dimethylamino)ethyl)-6-methoxy-2-methyl-4,7-dioxo-4,7-dihydro-1*H*-indole-3-carboxylate (36)

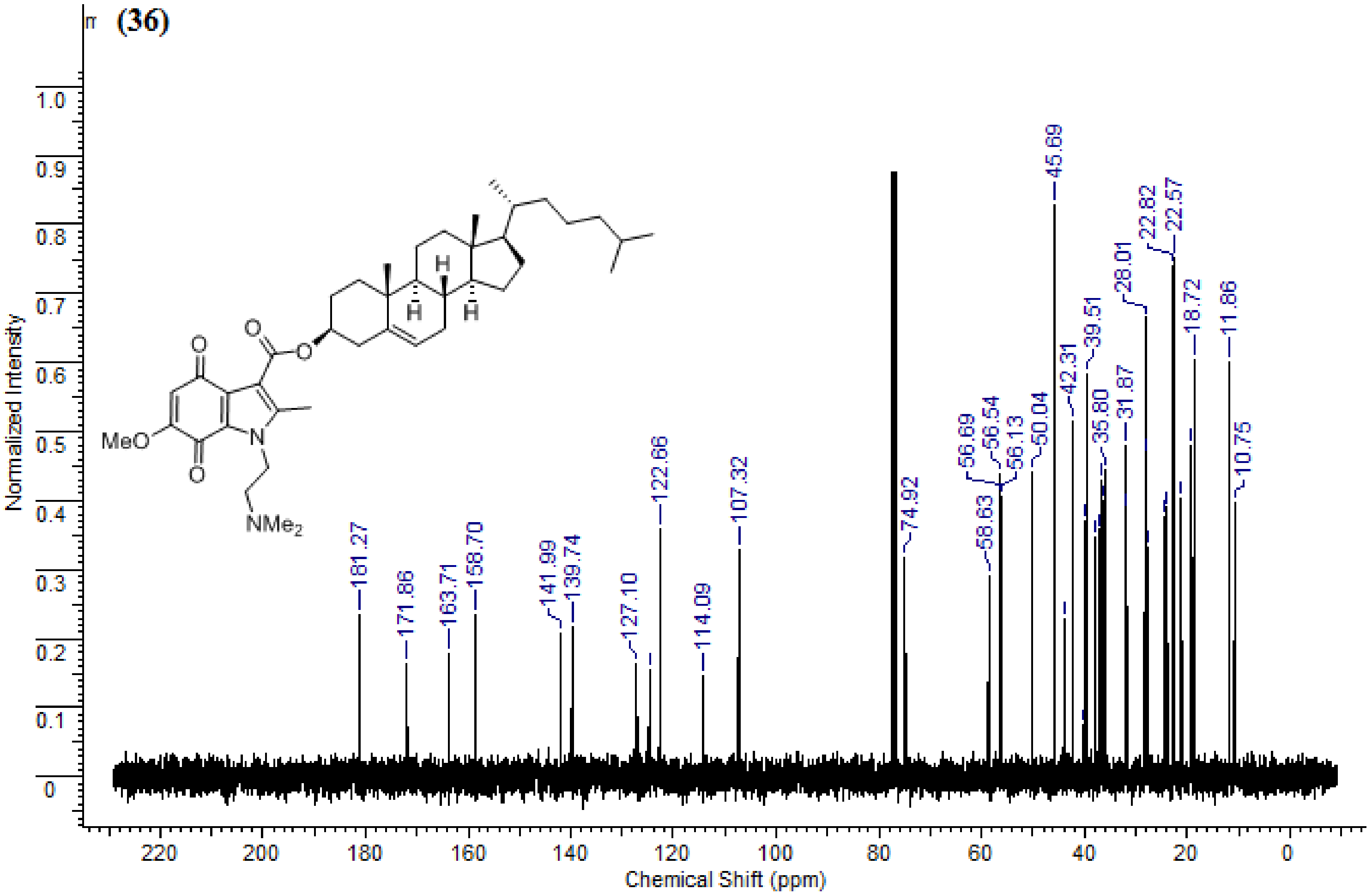

Dimethyl 1,2,6,7-tetramethyl-4,8-dioxo-1,4,7,8-tetrahydropyrrolo[3,2-*f*]indole-3,5-dicarboxylate (37)

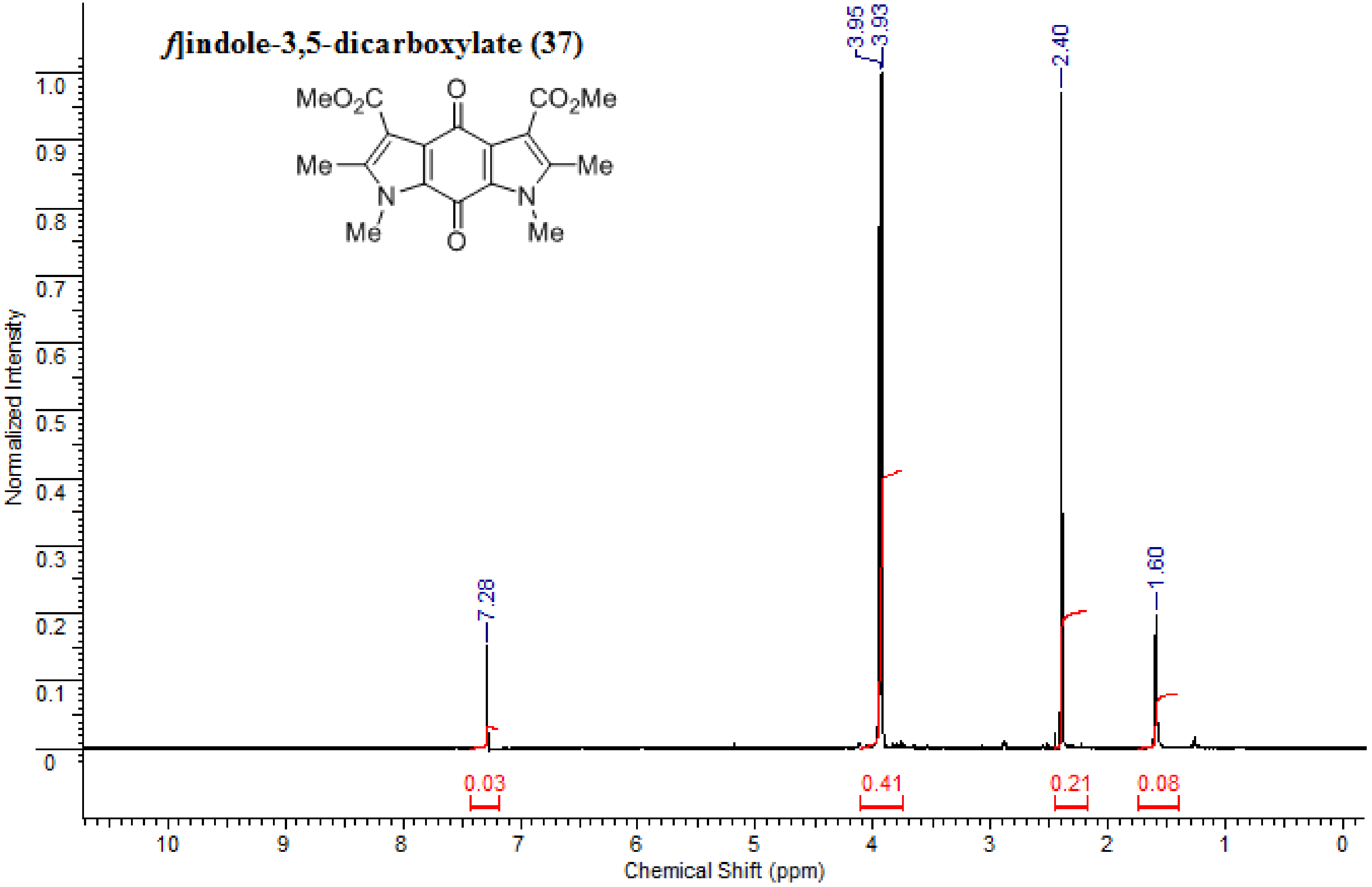

Dimethyl 1,2,6,7-tetramethyl-4,8-dioxo-1,4,7,8-tetrahydropyrrolo[3,2-*f*]indole-3,5-dicarboxylate (37)

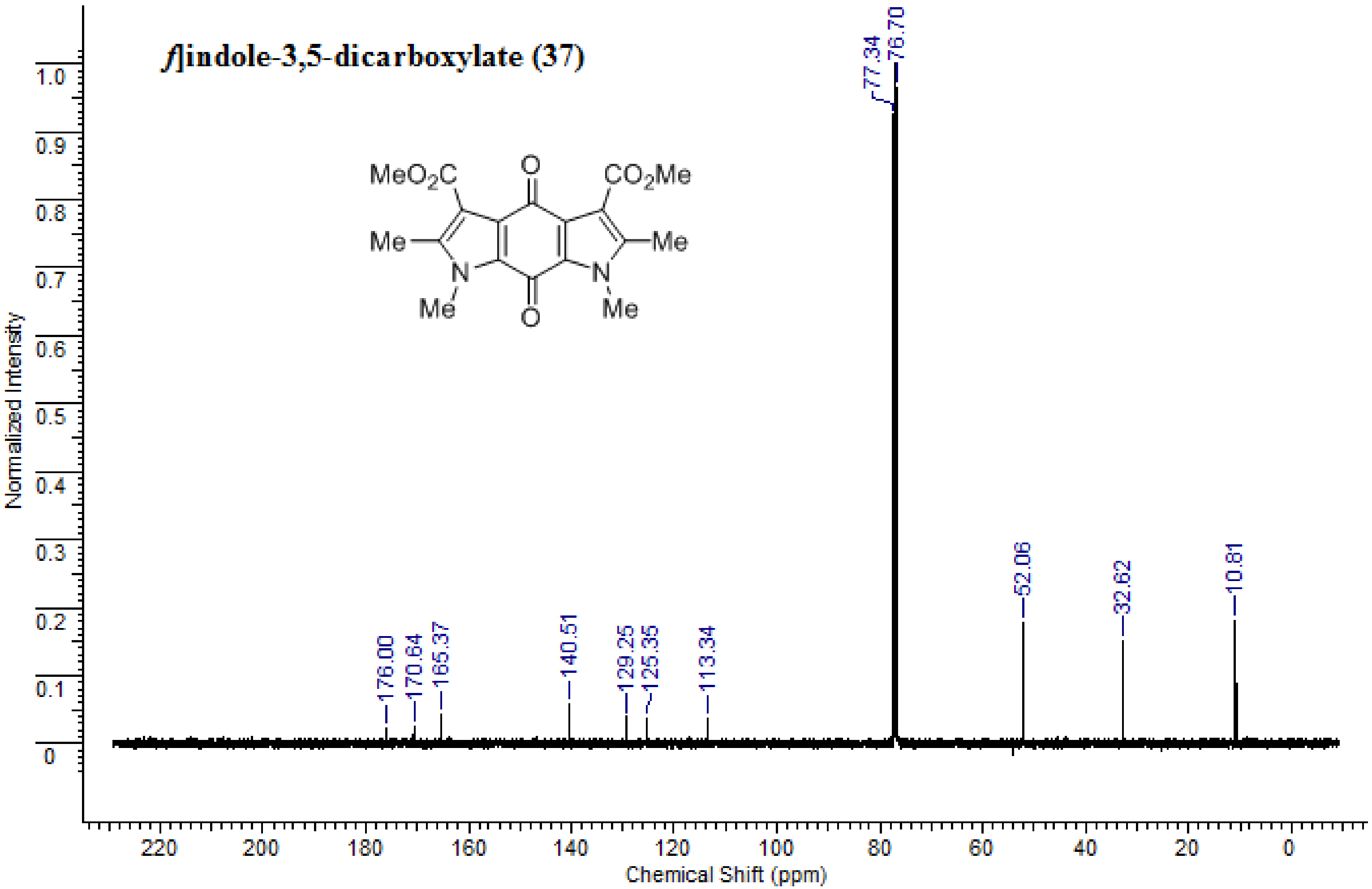

Methyl 6-bromo-1-(2-(*tert*-butyldiphenylsilyloxy)ethyl)-2-methyl-4,7-dioxo-4,7-dihydro-1*H*-indole-3-carboxylate (38)

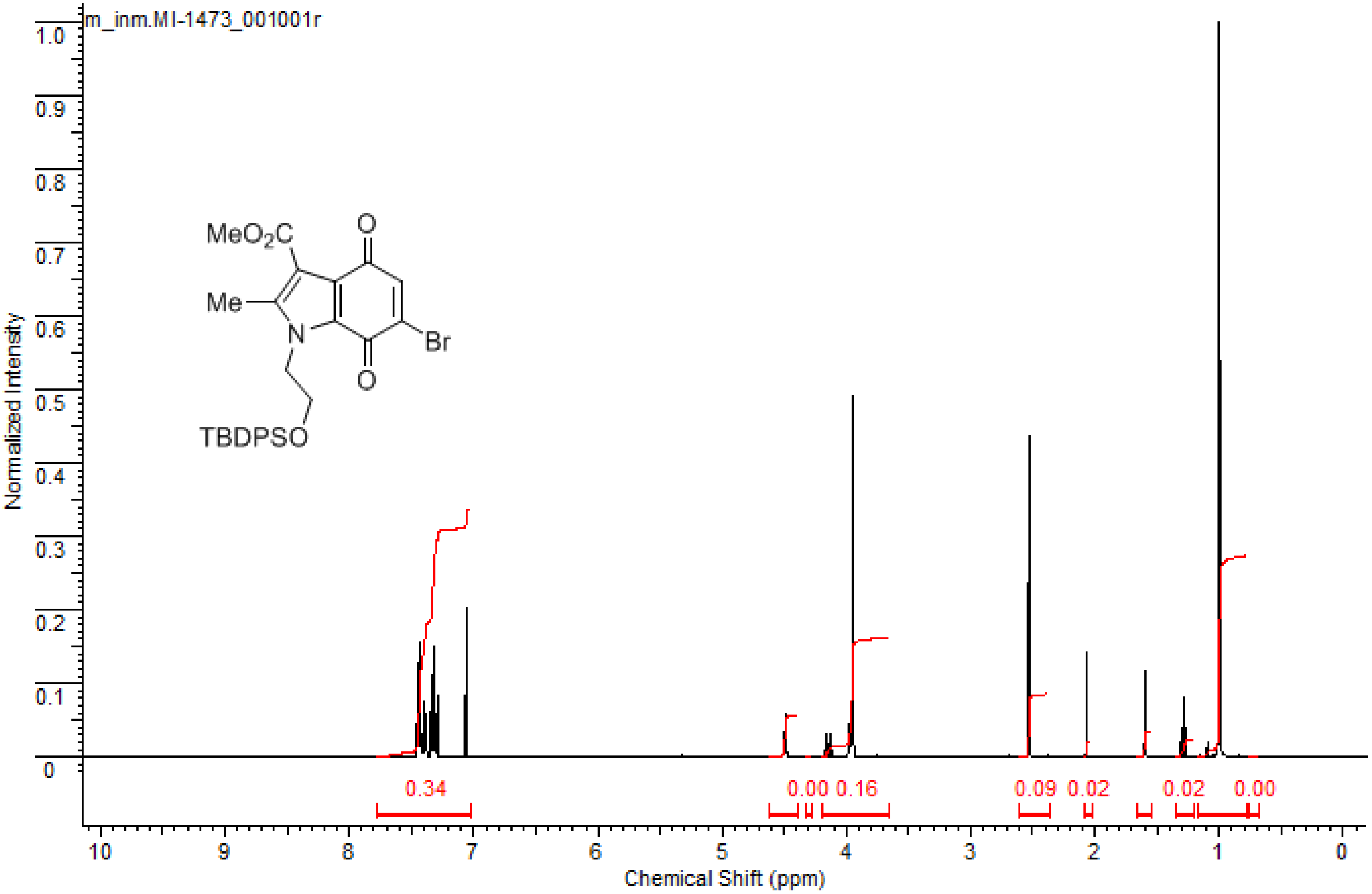

Methyl 6-bromo-1-(2-(*tert*-butyldiphenylsilyloxy)ethyl)-2-methyl-4,7-dioxo-4,7-dihydro-1*H*-indole-3-carboxylate (38)

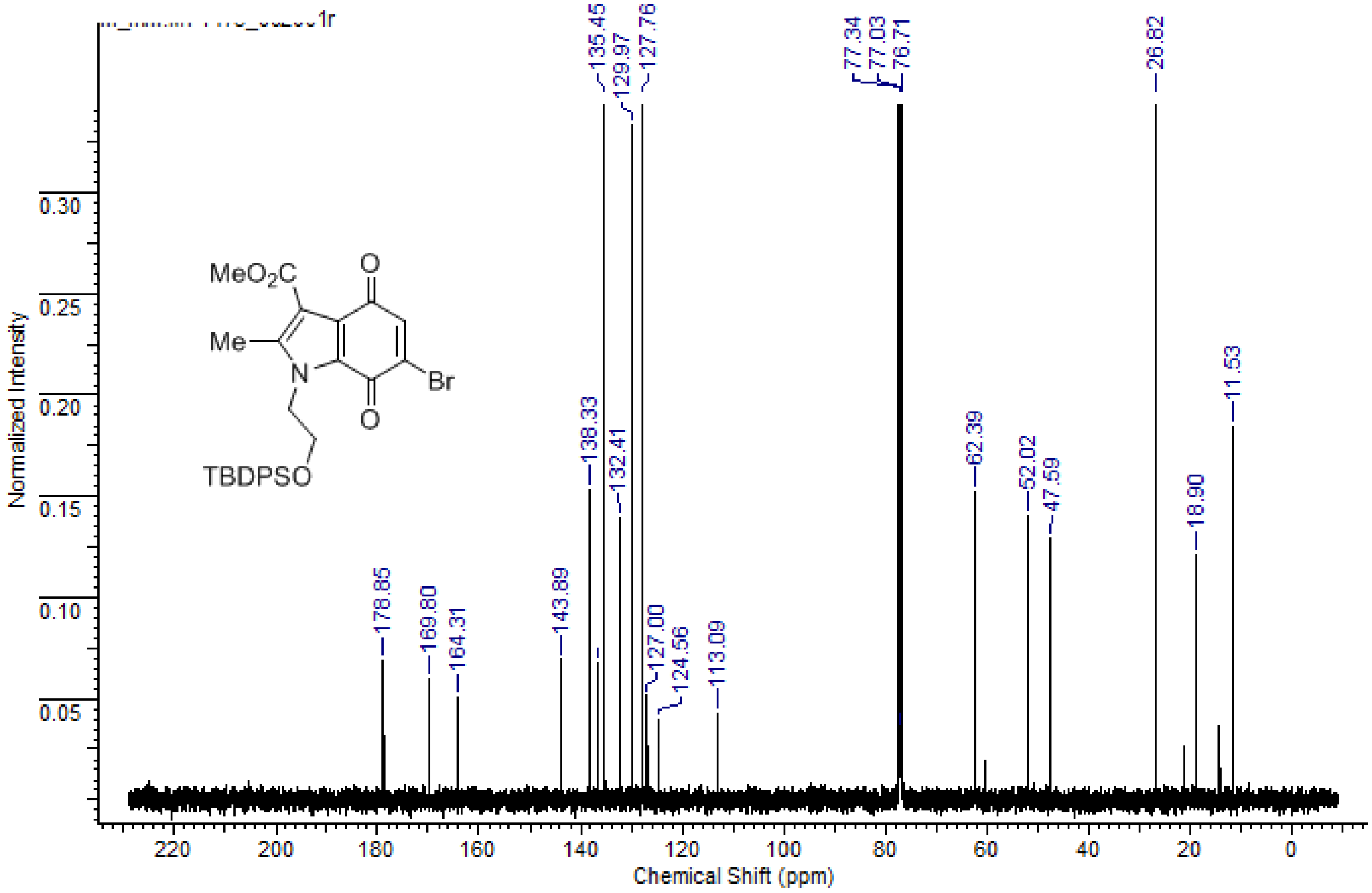

3-tert-Butyl 5-methyl 7-(2-(*tert*-butyldiphenylsilyloxy)ethyl)-1,2,6-trimethyl-4,8-dioxo-1,4,7,8-tetrahydropyrrolo[3,2-*f*]indole-3,5-dicarboxylate (39)

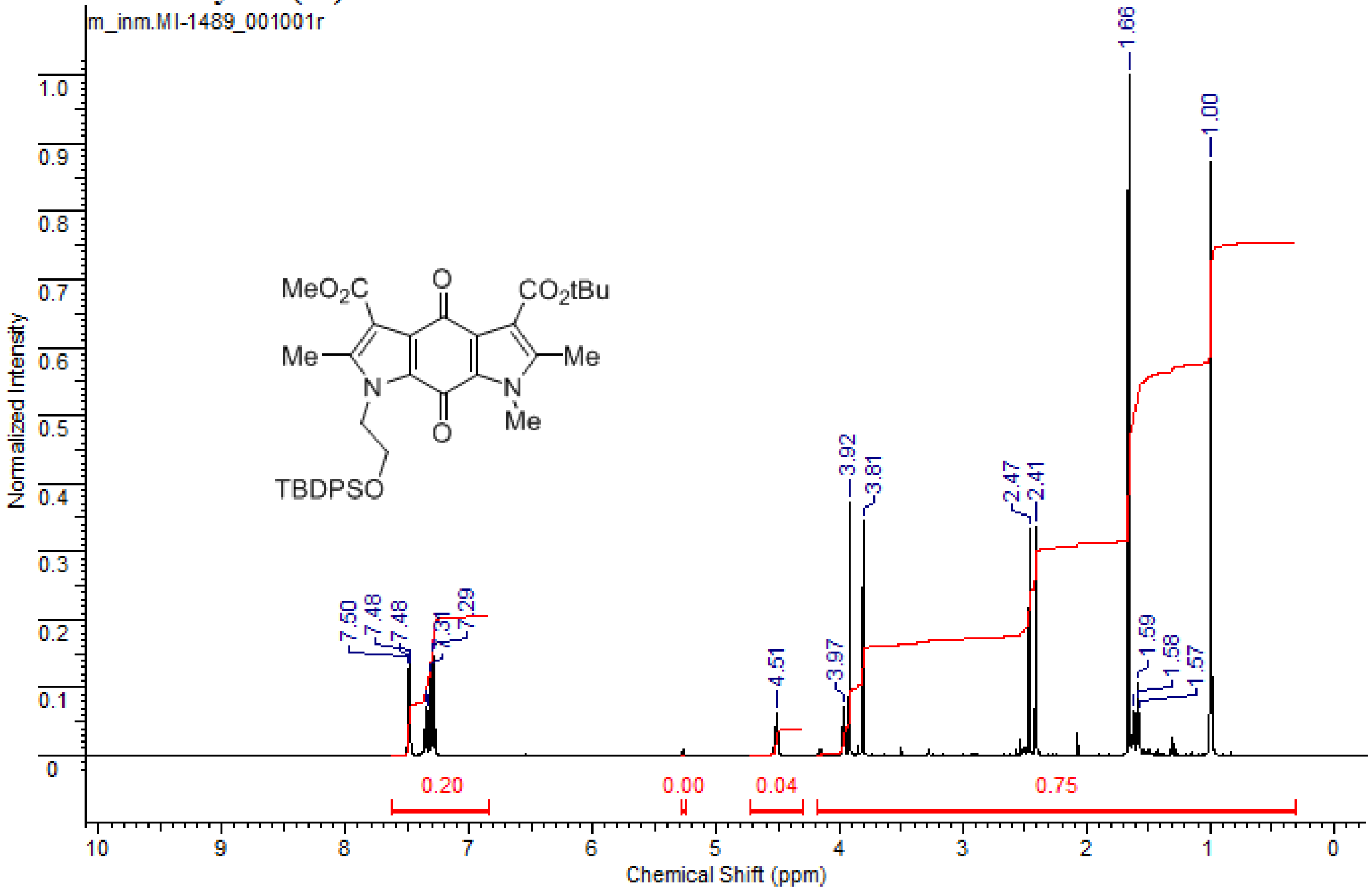

3-tert-Butyl 5-methyl 7-(2-(*tert*-butyldiphenylsilyloxy)ethyl)-1,2,6-trimethyl-4,8-dioxo-1,4,7,8-tetrahydropyrrolo[3,2-*f*]indole-3,5-dicarboxylate (39)

S

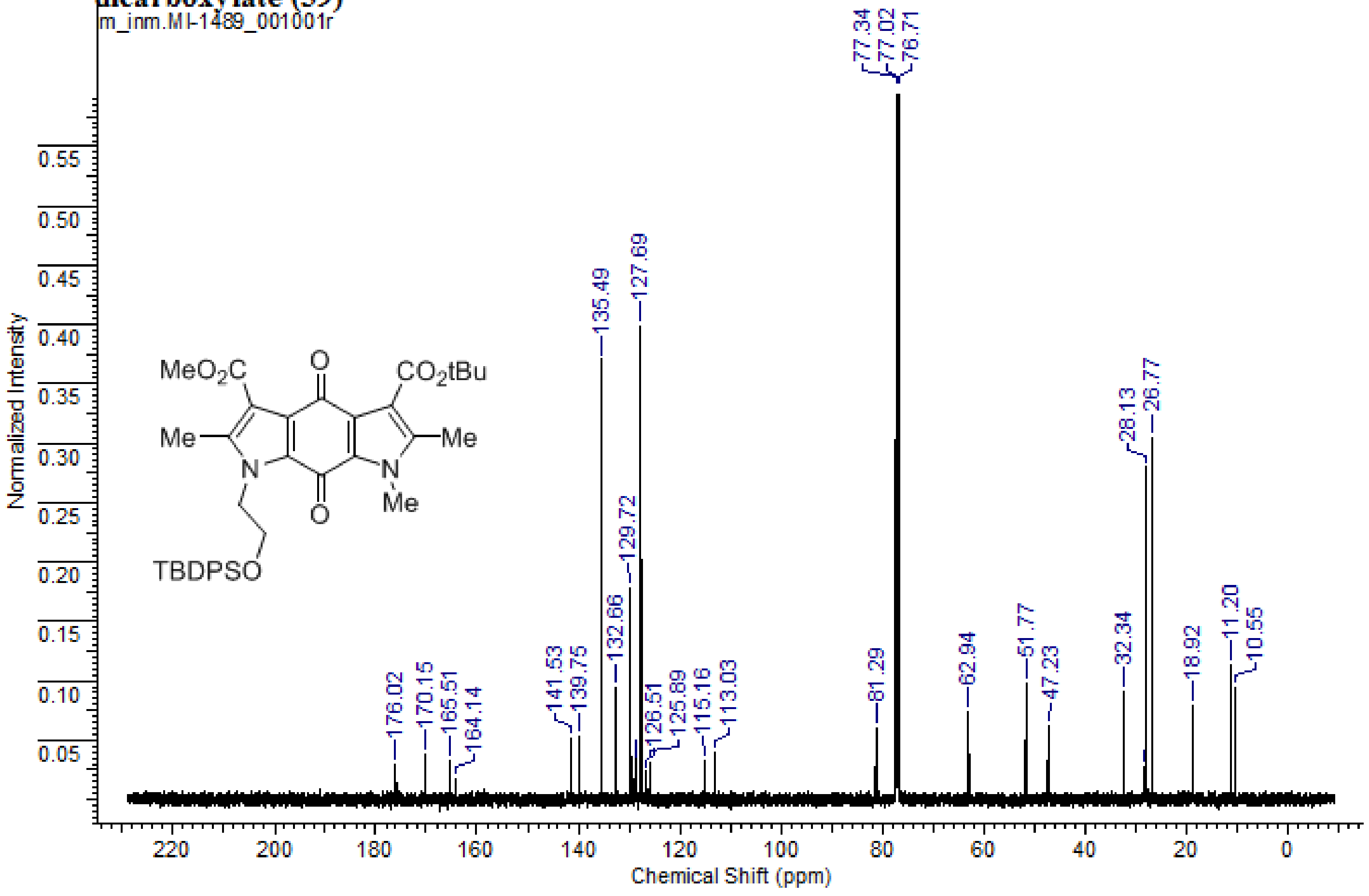

Dimethyl 1,2,5,6-tetramethyl-4,8-dioxo-1,4,5,8-tetrahydropyrrolo[2,3-*f*]indole-3,7-dicarboxylate (40)

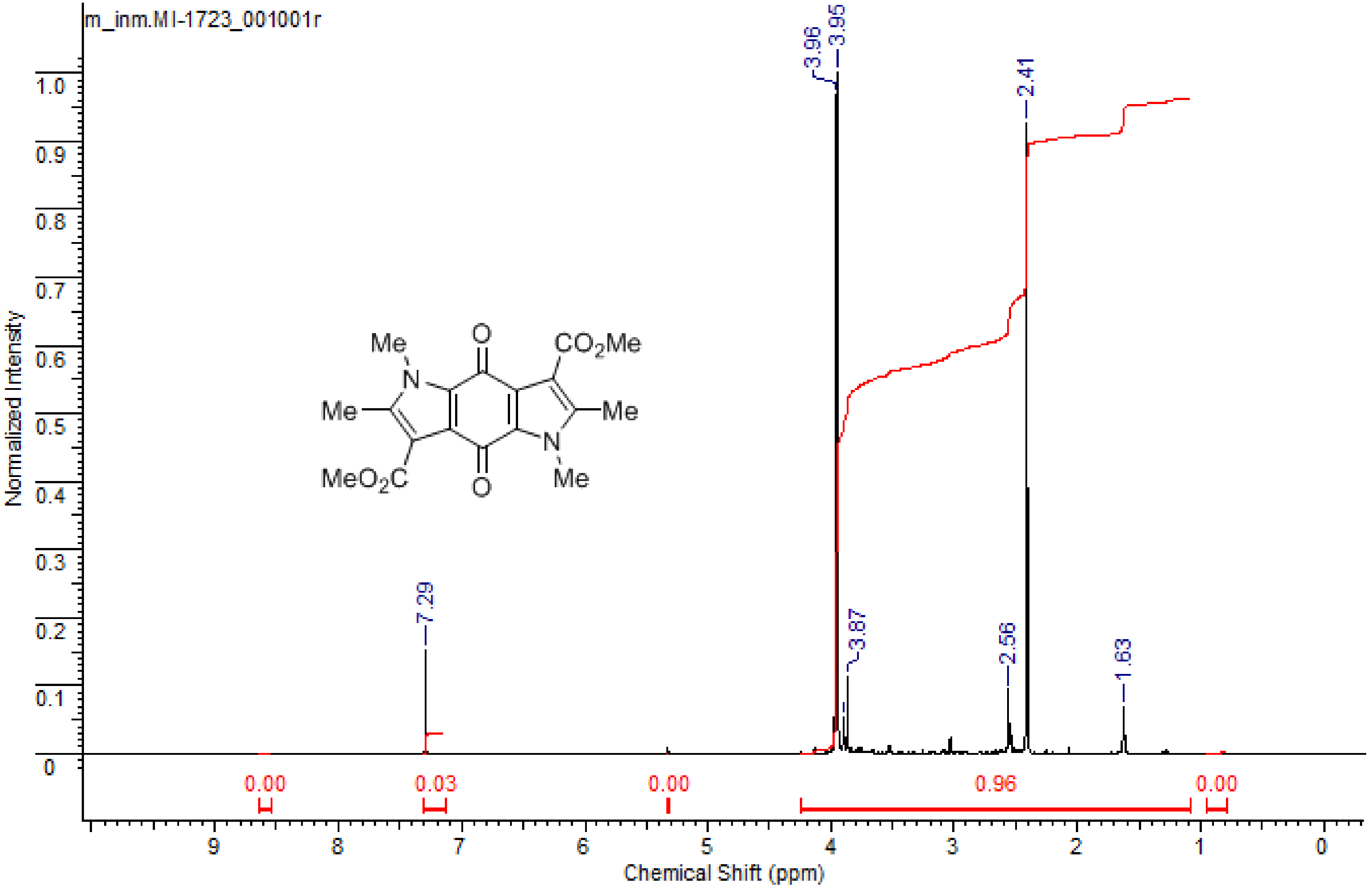

Dimethyl 1,2,5,6-tetramethyl-4,8-dioxo-1,4,5,8-tetrahydropyrrolo[2,3-*f*]indole-3,7-dicarboxylate (40)

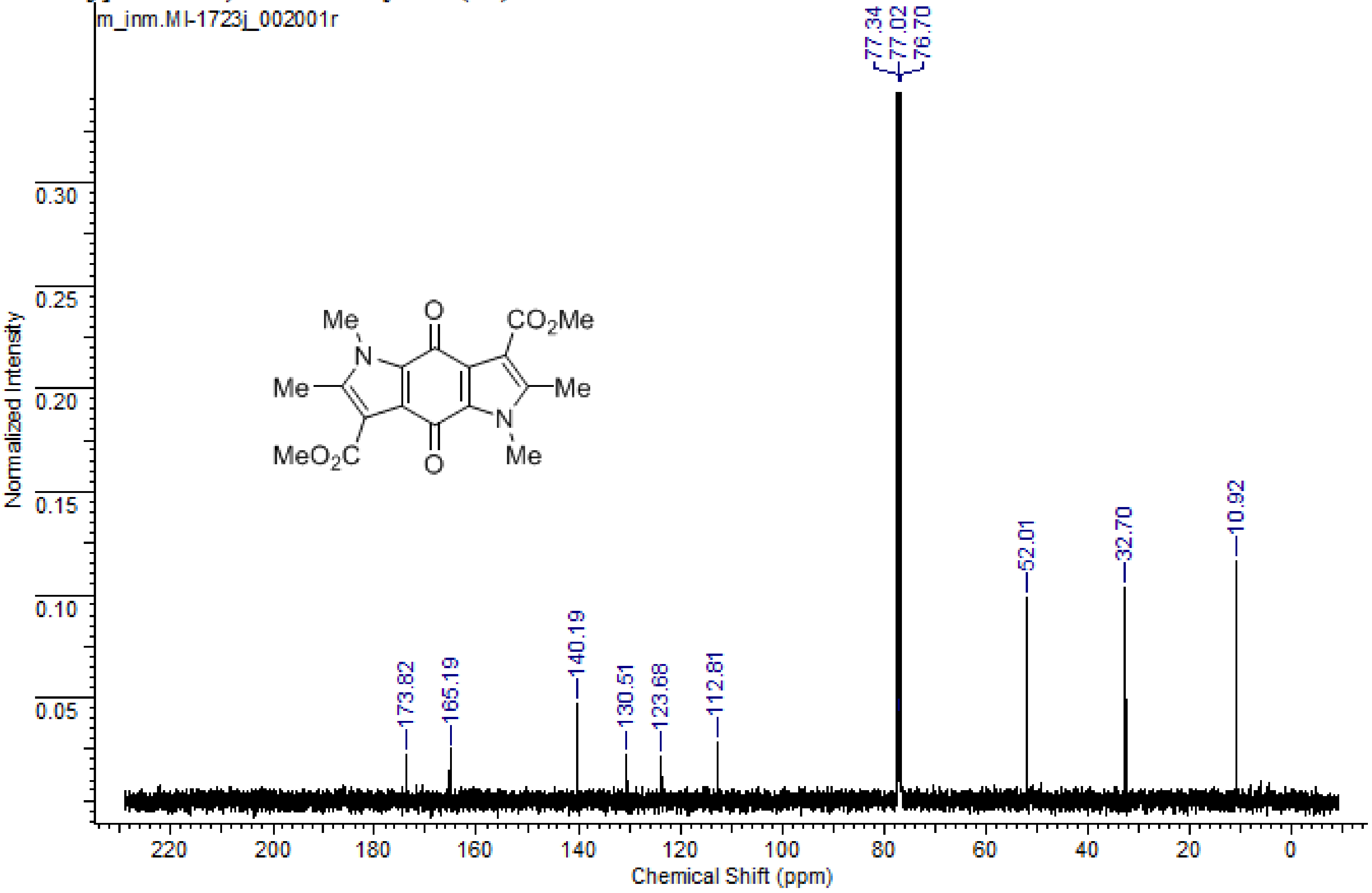

***tert*-Butyl 5-bromo-1,2-dimethyl-4,7-dioxo-4,7-dihydro-1*H*-indole-3-carboxylate (41)**

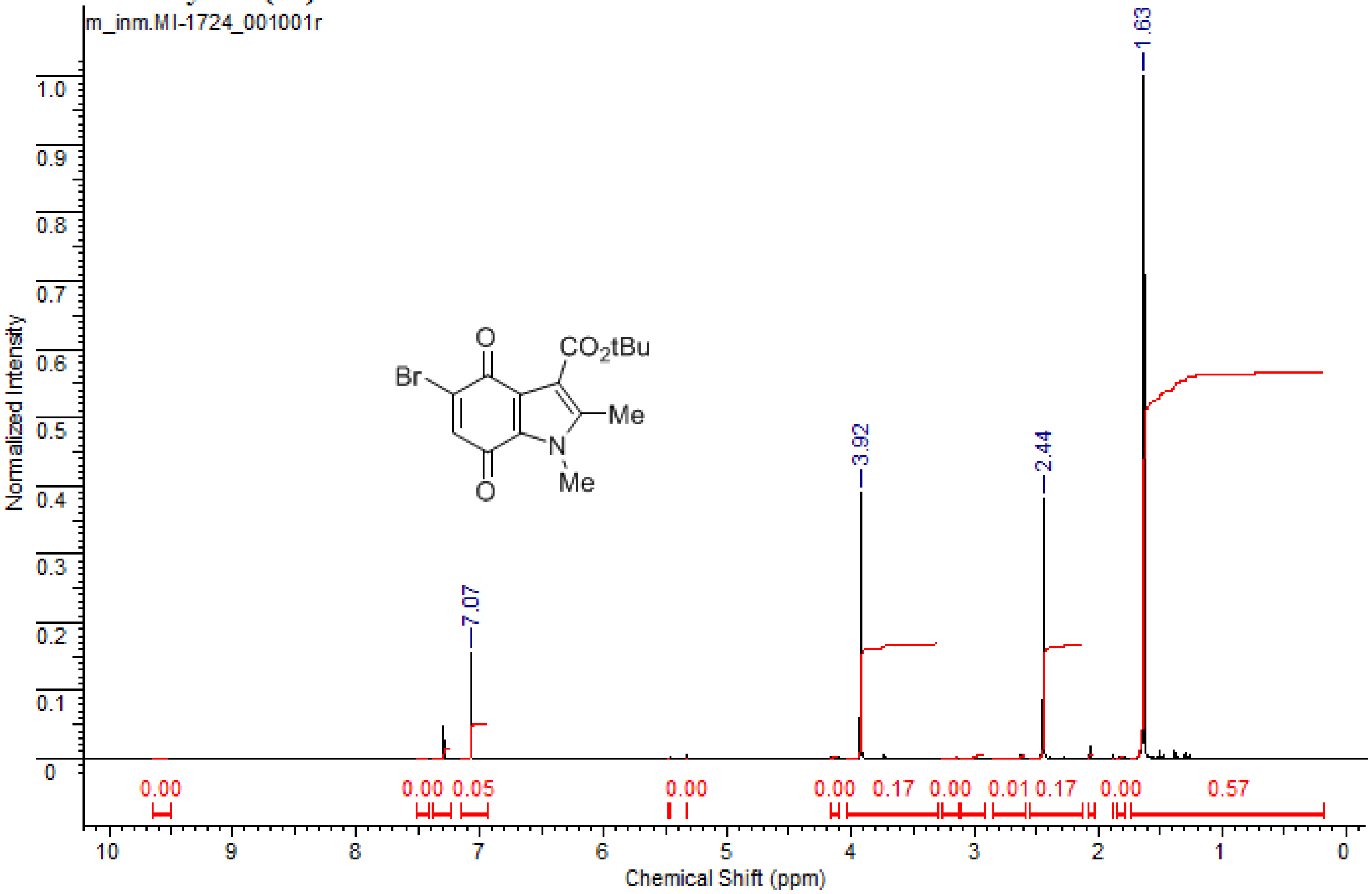

tert-Butyl 5-bromo-1,2-dimethyl-4,7-dioxo-4,7-dihydro-1H-indole-3-carboxylate (41)

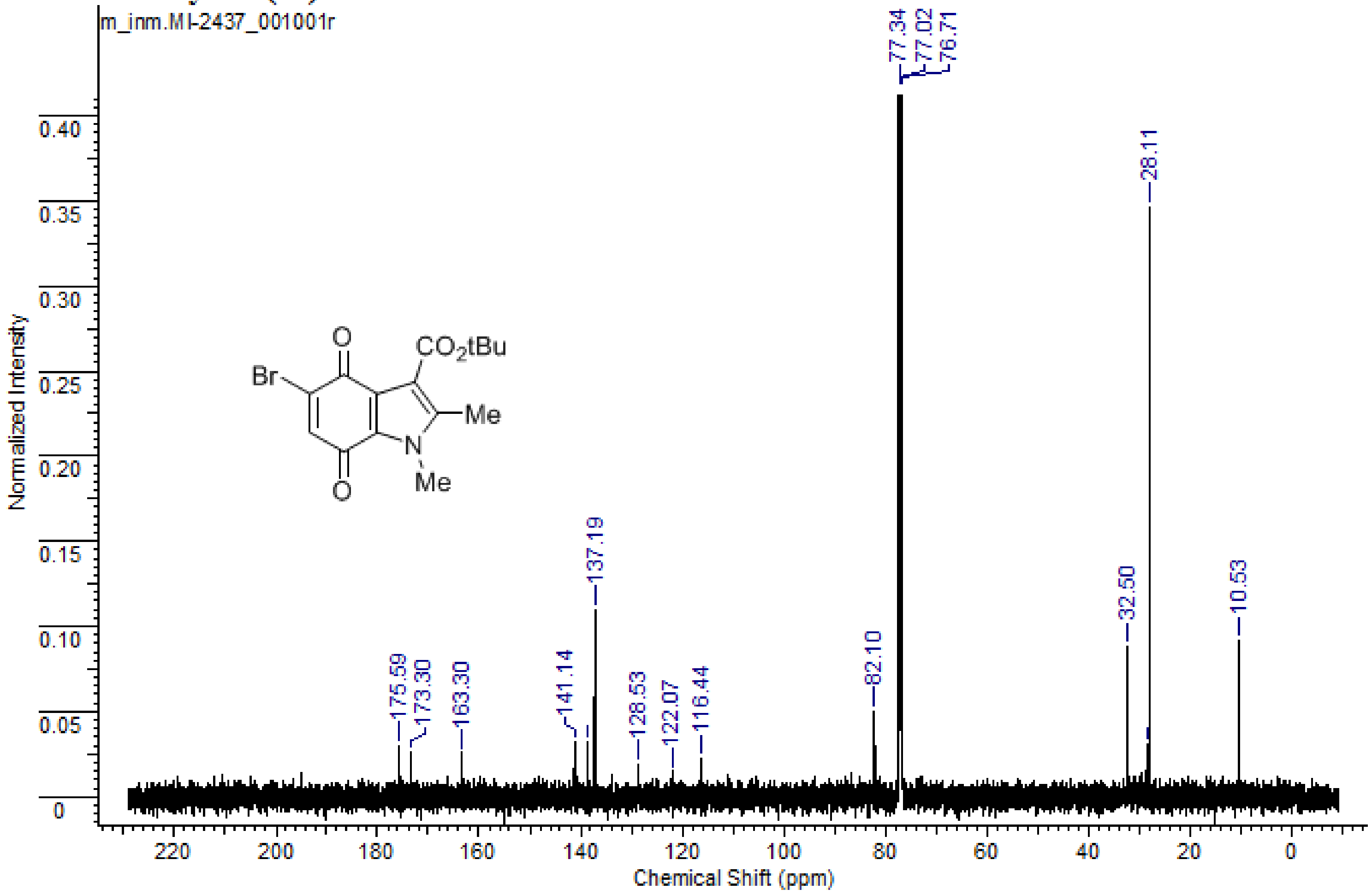

Methyl 6-methoxy-2-methyl-1-(4-methylpiperazin-1-yl)-4,7-dioxo-4,7-dihydro-1*H*-indole-3-carboxylate (44a)

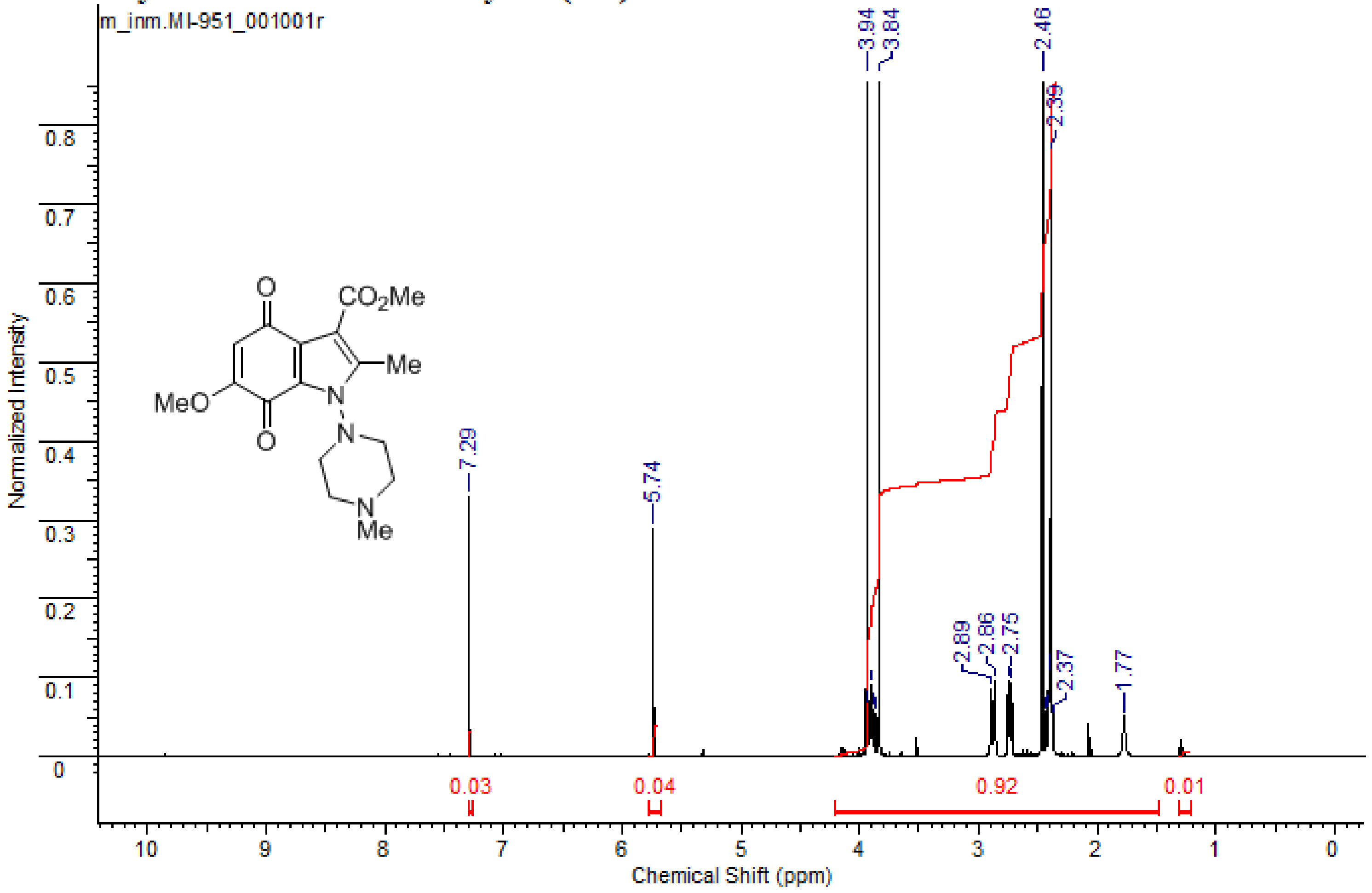

Methyl 6-methoxy-2-methyl-1-(4-methylpiperazin-1-yl)-4,7-dioxo-4,7-dihydro-1*H*-indole-3-carboxylate (44a)

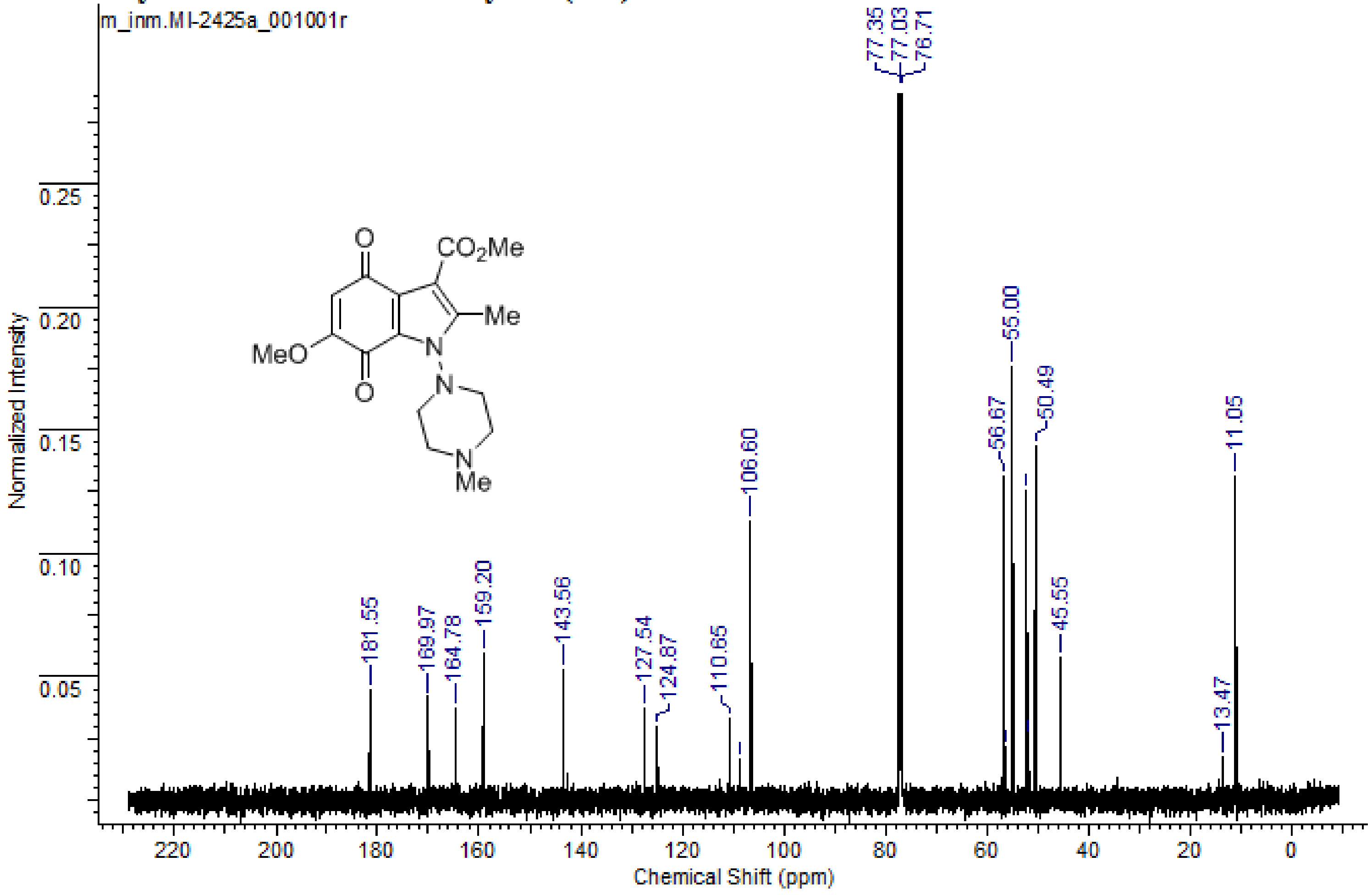

Methyl 6-methoxy-2-methyl-1-morpholino-4,7-dioxo-4,7-dihydro-1*H*-indole-3-carboxylate (44b)

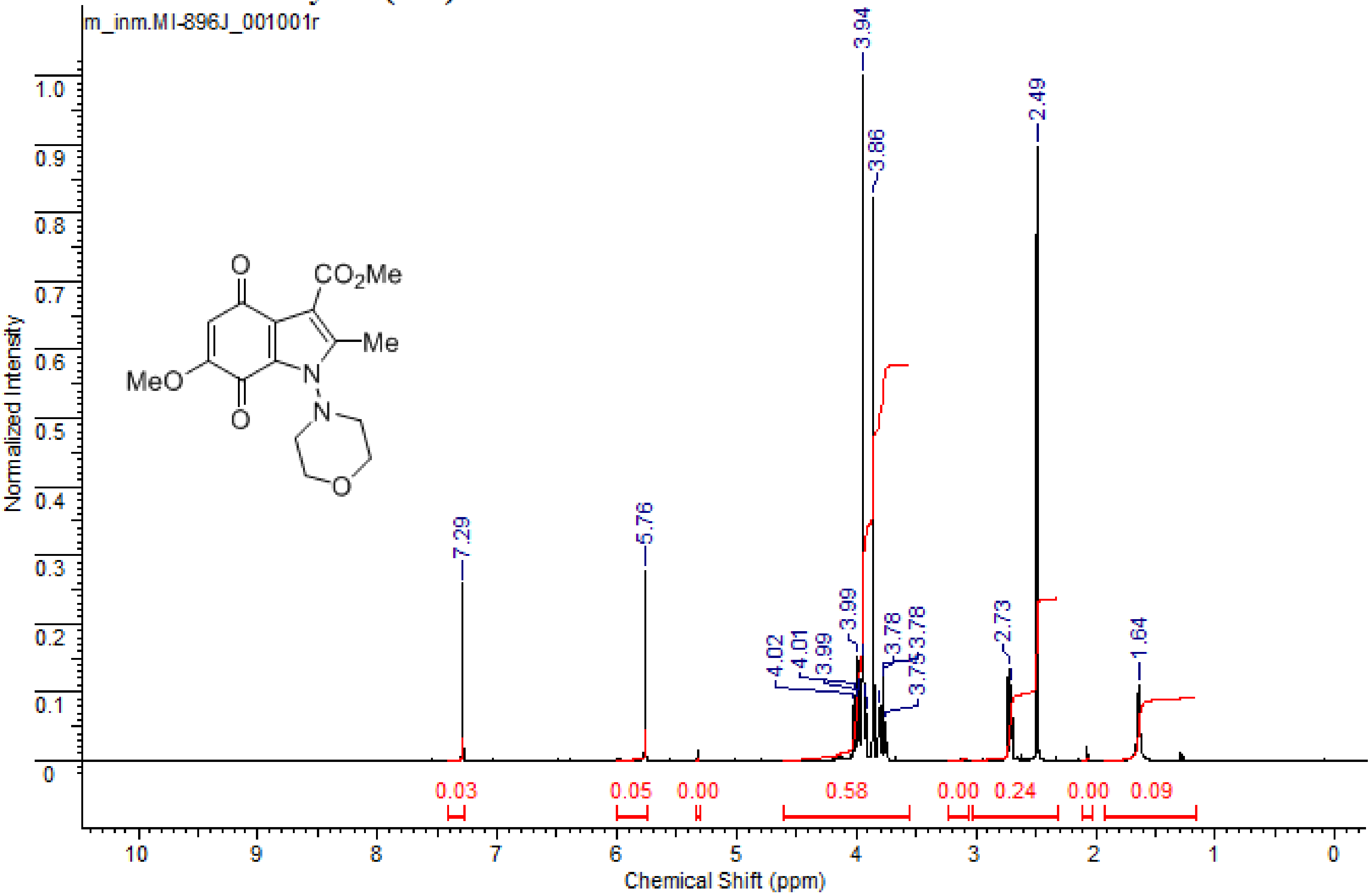

Methyl 6-methoxy-2-methyl-1-morpholino-4,7-dioxo-4,7-dihydro-1*H*-indole-3-carboxylate (44b)

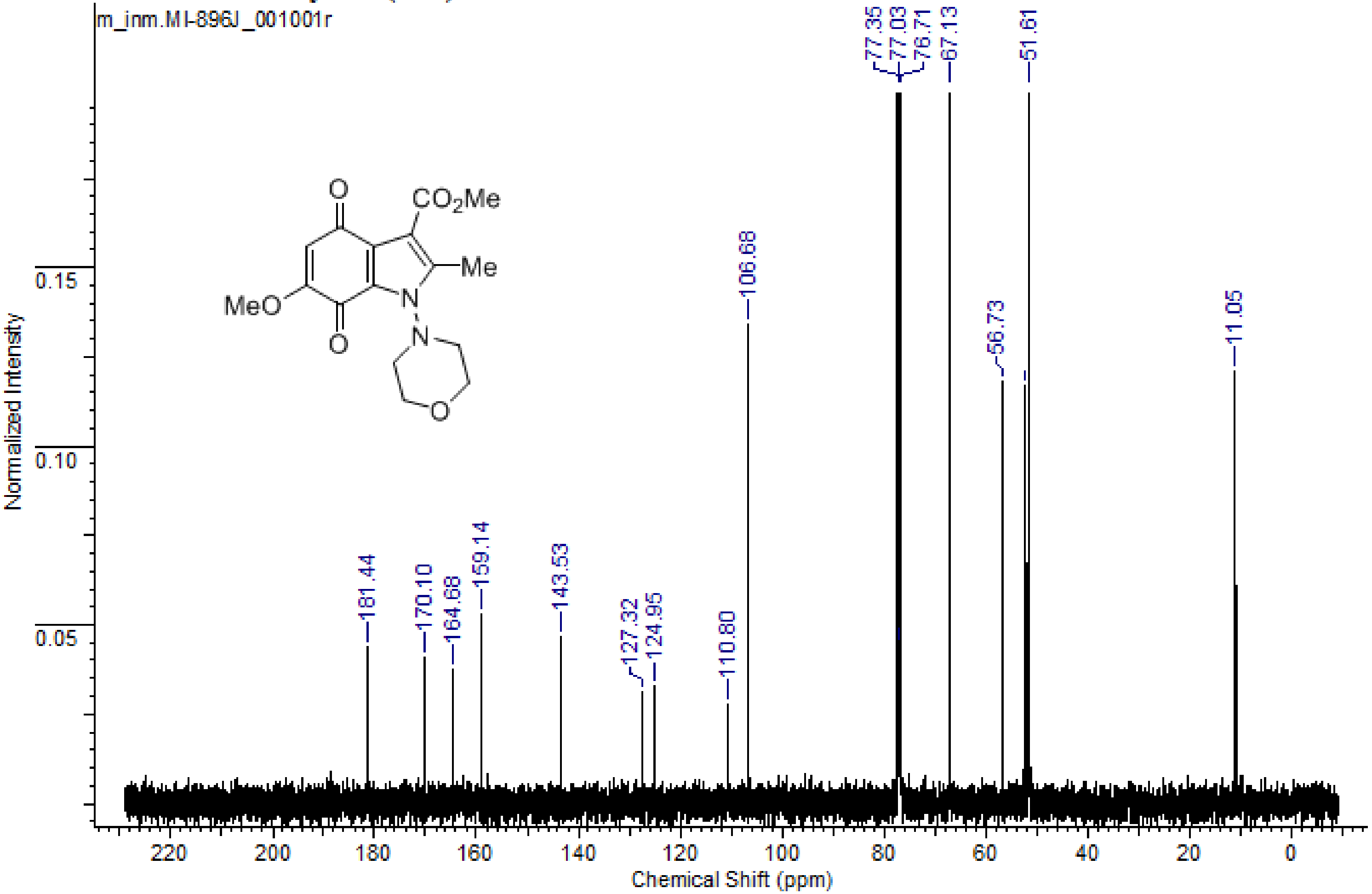

Methyl 1,6-dimethoxy-2-methyl-4,7-dioxo-4,7-dihydro-1*H*-indole-3-carboxylate (44c)

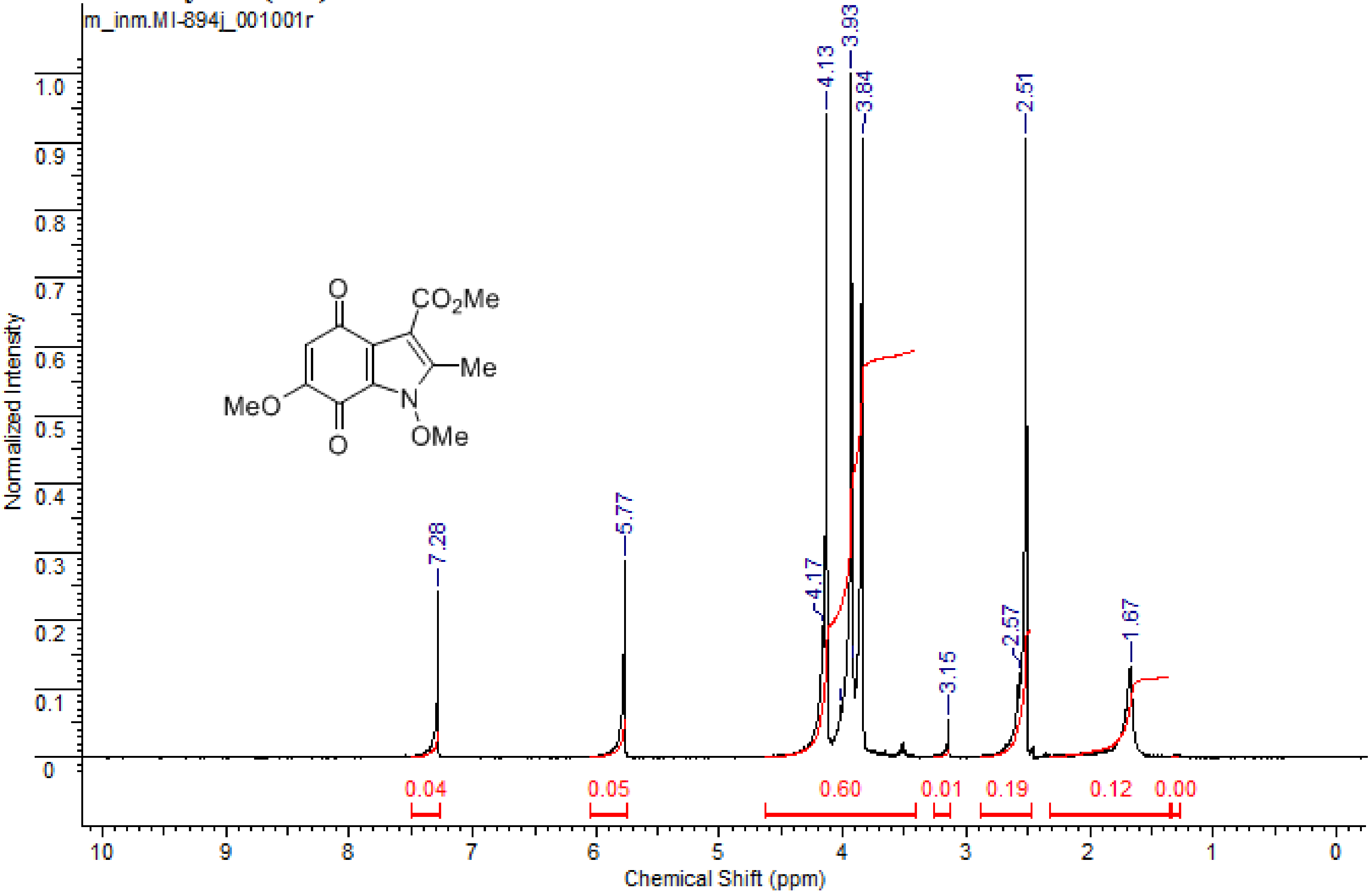

Methyl 1,6-dimethoxy-2-methyl-4,7-dioxo-4,7-dihydro-1*H*-indole-3-carboxylate (44c)

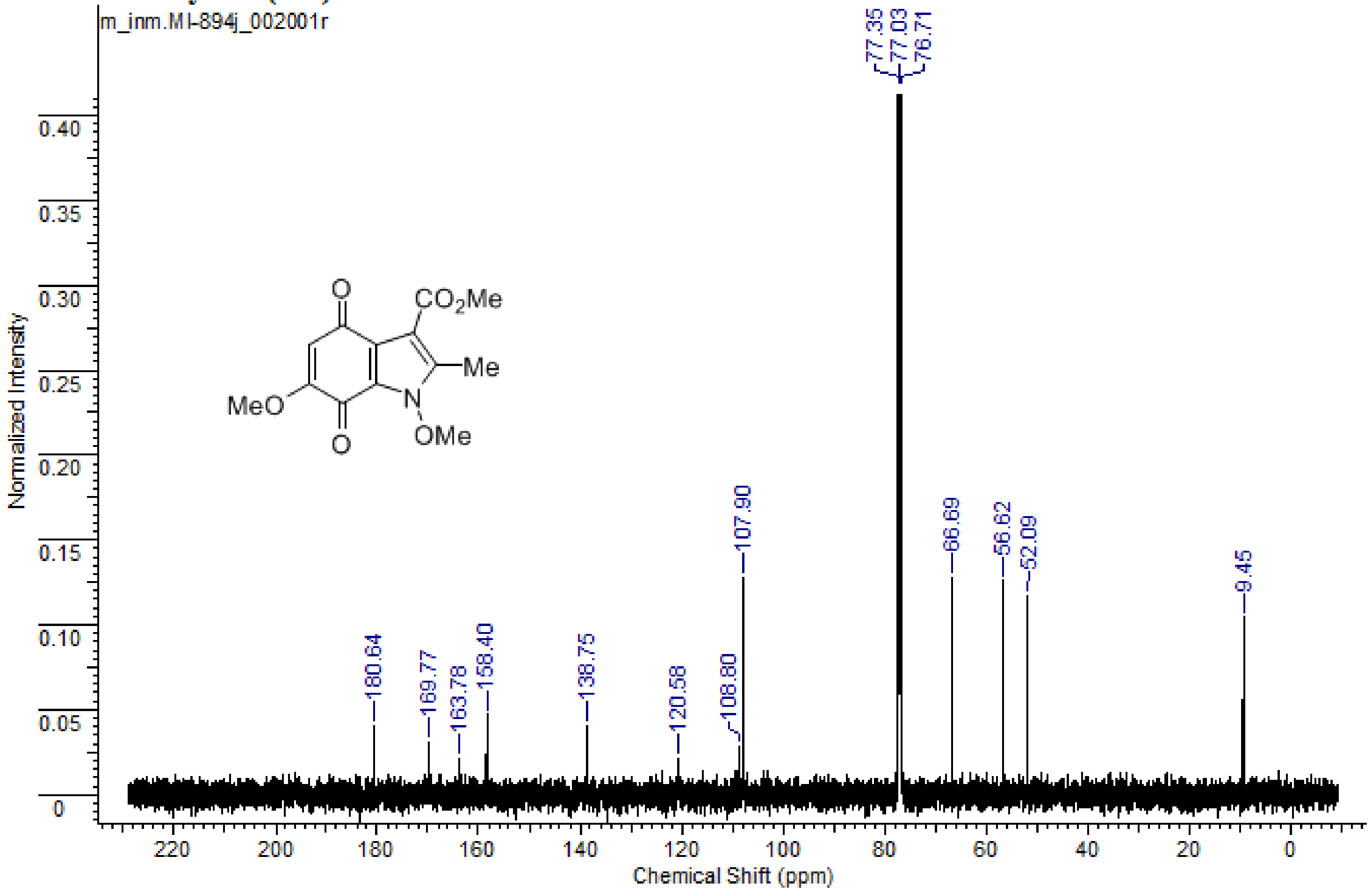

Supplement: Supplementary file 1 [file ejoc2013-2179-SD1.pdf]
